# Measurement properties of device-based physical activity instruments in ambulatory adults with physical disabilities and/or chronic diseases: a scoping review

**DOI:** 10.1186/s13102-023-00717-0

**Published:** 2023-09-21

**Authors:** Pim Brandenbarg, Femke Hoekstra, Ioulia Barakou, Bregje L. Seves, Florentina J. Hettinga, Trynke Hoekstra, Lucas H. V van der Woude, Rienk Dekker, Leonie A. Krops

**Affiliations:** 1 Department of Human Movement Sciences, University of Groningen, University Medical Center Groningen, Groningen, 9700 RB The Netherlands; 2 Department of Rehabilitation Medicine, University of Groningen, University Medical Center Groningen, Groningen, 9700 RB The Netherlands; 3 School of Health and Exercise Sciences, University of British Columbia Okanagan, Kelowna, BC V1V 1V7 Canada; 4 Department of Sport, Exercise and Rehabilitation, Northumbria University, Newcastle, NE1 8ST UK; 5 Department of Health Sciences and Amsterdam Public Health Research Institute, Vrije Universiteit Amsterdam, Amsterdam, 1081 BT The Netherlands

**Keywords:** Physical activity, Device-based instruments, Accelerometry, Measurement properties, Validity, Reliability, Responsiveness, Physical disability, Chronic disease, Scoping review

## Abstract

**Background:**

People with physical disabilities and/or chronic diseases tend to have an inactive lifestyle. Monitoring physical activity levels is important to provide insight on how much and what types of activities people with physical disabilities and/or chronic diseases engage in. This information can be used as input for interventions to promote a physically active lifestyle. Therefore, valid and reliable physical activity measurement instruments are needed. This scoping review aims 1) to provide a critical mapping of the existing literature and 2) directions for future research on measurement properties of device-based instruments assessing physical activity behavior in ambulant adults with physical disabilities and/or chronic diseases.

**Methods:**

Four databases (MEDLINE, CINAHL, Web of Science, Embase) were systematically searched from 2015 to April 16^th^ 2023 for articles investigating measurement properties of device-based instruments assessing physical activity in ambulatory adults with physical disabilities and/or chronic diseases. For the majority, screening and selection of eligible studies were done in duplicate. Extracted data were publication data, study data, study population, device, studied measurement properties and study outcome. Data were synthesized per device.

**Results:**

One hundred three of 21566 Studies were included. 55 Consumer-grade and 23 research-grade devices were studied on measurement properties, using 14 different physical activity outcomes, in 23 different physical disabilities and/or chronic diseases. ActiGraph (*n* = 28) and Fitbit (*n* = 39) devices were most frequently studied. Steps (*n* = 68) was the most common used physical activity outcome. 97 studies determined validity, 11 studies reliability and 6 studies responsiveness.

**Conclusion:**

This scoping review shows a large variability in research on measurement properties of device-based instruments in ambulatory adults with physical disabilities and/or chronic diseases. The variability highlights a need for standardization of and consensus on research in this field. The review provides directions for future research.

**Supplementary Information:**

The online version contains supplementary material available at 10.1186/s13102-023-00717-0.

## Background

Physical activity (PA), defined as “any bodily movement produced by skeletal muscles that result in energy expenditure “ [1], is a multidimensional construct with dimensions as setting (e.g. PA during leisure time, work), mode (e.g. walking, bicycling), frequency (e.g. times per week), duration (e.g. in hours) and intensity (e.g. light, moderate or vigorous) [2, 3]. PA has many health benefits across the lifespan, especially for people with physical disabilities and/or chronic diseases [4, 5]. Still, people with physical disabilities and/or chronic diseases tend to have an inactive lifestyle [6, 7]. Monitoring PA in this population is important, as it will provide insight in how much and what types of PA they engage in. Information on the amount and types of PA can help tailor PA promotion activities to individuals and uncover opportunities for improving PA for people with physical disabilities and/or chronic diseases. Furthermore, self-monitoring is one of the most effective behavior change techniques for improving PA, further stressing the importance of accurately measuring PA [8]. The need to measure and quantify PA in this varied population has also been emphasized by various research groups [9, 10], including the developers of the new World Health Organization’s PA guidelines [11].

A variety of instruments exist to measure PA in people with physical disabilities and/or chronic diseases. Instruments for PA measurement can be classified into two main categories: device-based instruments (e.g. accelerometers and pedometers; later also mentioned as devices) and self-report instruments (e.g. questionnaires and diaries). Both types of instruments have advantages and disadvantages [12] and are believed to measure different aspects of the PA construct [13]. Self-report instruments are assumed to capture the perceived PA behavior, whereas device-based instruments aim to capture the continuous acceleration of the body above a certain threshold [13]. The consensus is currently that both types of instruments have their own value and should be used complementary to one another, depending on the research questions or clinical and/or practical goals [14].

Device-based instruments collect raw movement data (e.g. acceleration) from a variety of locations on the human body. These data are converted into different PA outcomes (e.g. energy expenditure, steps) often using dedicated algorithms [15]. These algorithms are commonly developed for a general (non-disabled) population [9]. People with physical disabilities and/or chronic diseases such as those with stroke, Parkinson’s disease, and chronic obstructive pulmonary disorder, might have a different pattern of locomotion (e.g. slower and/or asymmetrical) [16–18]. Also, people with physical disabilities and/or chronic diseases could have a different energy expenditure during PA compared to people without physical disabilities and/or chronic diseases, due to a lower efficiency of walking or other motor actions in general [19–21] or due to an increased energy cost of daily activities [22]. This could be of influence on the validity of the algorithms used in device-based PA instruments when applied to people with physical disabilities and/or chronic diseases. Research already showed that slower walking speeds limit the validity of measuring steps using certain devices [23, 24]. Furthermore, energy expenditure estimations of devices had poor correlations with estimations of indirect calorimetry in people with stroke [25]. These findings warrant a critical mapping of the measurement properties of device-based instruments used to assess PA in people with physical disabilities and/or chronic diseases.

There have been reviews in the past on the measurement properties of device-based instruments in people with physical disabilities and/or chronic diseases. However, these are mostly either diagnosis- or PA-outcome specific [25–29]. Also, manual wheeled mobility involves a completely different class of bodily activities and their energetic consequences as opposed to individuals who walk. A recent systematic review gave an extensive overview of the measurement properties of device-based and self-reported instruments assessing PA in people using a wheelchair [30]. Therefore, the current review focused on the ambulatory population of adults with physical disabilities and/or chronic diseases.

This scoping review aims to provide a critical mapping of the existing literature on the measurement properties of device-based instruments assessing physical activity behavior in ambulant adults with various physical disabilities and/or chronic diseases. Using this critical mapping, we provide future directions to study the measurement properties of device-based instruments assessing PA in ambulatory adults with physical disabilities and/or chronic diseases.

## Methods

### Study design

This scoping review was guided by the methodological framework for scoping reviews [31, 32] and the Preferred Reporting Items for Systematic reviews and Meta-Analyses extension for Scoping Reviews (PRISMA-ScR) guideline [33]. A scoping review was chosen as it can be used to summarize research findings and potentially identify research gaps in the literature, which matches our aim. The study protocol is available at https://osf.io/c27xv/. During the review process, we deviated from the published protocol. In Supplementary file 1 we report the reason and the nature of these deviations. In short, we deviated from the protocol in three main ways: 1) because of the large amount of research, we changed the scope of the review from all literature on both device-based and self-reported instruments into only device-based instruments in a set time period; 2) we therefore changed the review question accordingly; and 3) we changed the method from a systematic into a scoping review.

Following the aim and scope of the original protocol, we defined the following PICO criteria: *(P)* Adults (≥ 18 years old) with physical disabilities and/or chronic diseases. *Physical disability* was defined as a congenital disease, acquired illness, or trauma that causes an impairment, activity limitation and participation restriction that lasts at least 1 year [34, 35]. *Chronic disease* was defined broadly as conditions that last 1 year or more and require ongoing medical attention or limit activities of daily living or both [36]. *(I)* Physical activity measurement instrument. *Physical activity measurement instrument* was defined as a device-based or self-report instrument that assesses any bodily movement produced by the muscles that results in increased energy expenditure [1] in the activity domain of the International Classification of Function, Disability and Health (ICF) model [35]. *(C)* We did not use a comparison group, since this is not relevant for studies on measurement properties. *(O)* Measurement properties (e.g. reliability, validity, responsiveness). Operationalization of *Measurement properties* followed the definitions of COSMIN [37].

### Search strategy and information sources

Together with an information specialist (KS), we combined the three different concepts of our PICO to create our search terms: physical activity measurement instrument, physical disability and/or chronic disease and measurement properties. We used a combination of both MeSH-terms and free text words for each concept, linked with Boolean operators. Literature was initially searched up to June 26^th^ 2019, with a first update of the search up to November 20^th^ 2020, and a second update of the search up to April 16^th^ 2023 in four databases: Medline, Cinahl, Web of Science and Embase. We adapted the search strategy for each database using the same keywords and, where possible, MeSH-terms. The full search strategies for each of the four databases can be found in Supplementary file 2.

### Eligibility criteria

Articles were eligible for inclusion in the scoping review when 1) included participants were 18 years or older and had a physical disability or chronic disease, with having the physical disability or chronic disease a primary reason for rehabilitation treatment; 2) PA was measured as an amount or energy cost using a self-reported or device-based instrument; 3) measurement properties were a (primary or secondary) outcome measure of the studies; 4) articles were published in peer-reviewed journals and involved primary research. Articles were excluded when 1) studies were not in humans; 2) participants had an intellectual-, sensory-, cognitive- or mental disability; 3) all included participants were wheelchair users; 4) PA was measured as a functional or a performance outcome; 5) articles were not in English or Dutch. We excluded literature studying participants with intellectual-, sensory-, cognitive- or mental disabilities, as these studies may require different approaches and interpretations compared to studies involving people with physical disabilities and/or chronic diseases. As the authors are knowledgeable in Dutch and English, we excluded all non-English/Dutch articles.

### Selection of sources of evidence

Before screening, duplicates were removed using Bramer et al.’s method [38] in EndNote. Two researchers independently screened titles (PB & LAK) and subsequently abstracts (PB & IB) on eligibility using custom Excel spreadsheets. Disagreement was resolved by including those articles to the next phase. For the title and abstract phase, pilot tested checklists with specific instructions for in- and exclusion were used. During the abstract screening phase, regular meetings were held to ensure equal interpretation of the abstracts between both researchers and to discuss uncertainties. Before full text screening, articles were removed that used self-reported PA instruments or were published before 2015. We did this due to the change of focus (on devise-based instruments only) of the review after the abstract phase (see Supplementary file 1).

Eligibility of full texts was screened by two researchers independently (PB & IB), using a checklist for full text eligibility and a custom Excel spreadsheet. Disagreements were discussed, and if necessary, a third assessor (LAK) was consulted. Cohen’s Kappa statistics were calculated to assess the agreement between the two screeners for the title, abstract and full text phase [39]. For feasibility reasons, the second update was performed by one researcher (PB) only. A second researcher (LAK) was consulted in case of questions and doubt with respect to the interpretation of the study. The PICO, in- and exclusion criteria and complete checklists per phase can be found in Supplementary file 3. The used custom Excel spreadsheets can be found on Open Science Framework (https://osf.io/c27xv/).

### Data charting process

The first author (PB) extracted data using an extraction form in Excel (available at Open Science Framework: https://osf.io/c27xv/). The data extraction form included the following information: 1) publication data (author, year of publication, land of origin); 2) study data (design, setting, sample size, and protocol tasks); 3) study population (diagnosis group(s), age, gender, and walking speed); 4) device (name, type, placement, unit of measurement, epoch length, sampling rate, and algorithm used); 5) studied measurement properties (validity, reliability, or responsiveness) and criterion measure (name, type, unit of measurement, algorithm used); and 6) study outcomes.

### Synthesis of results

We synthesized the data based on device. For each device, the available measurement properties were presented using the following ordering: 1) PA outcome; 2) diagnosis group; 3) study; 4) device placement; and 5) algorithm. We separated research-grade devices from consumer-grade devices.

## Results

**Fig. 1:**
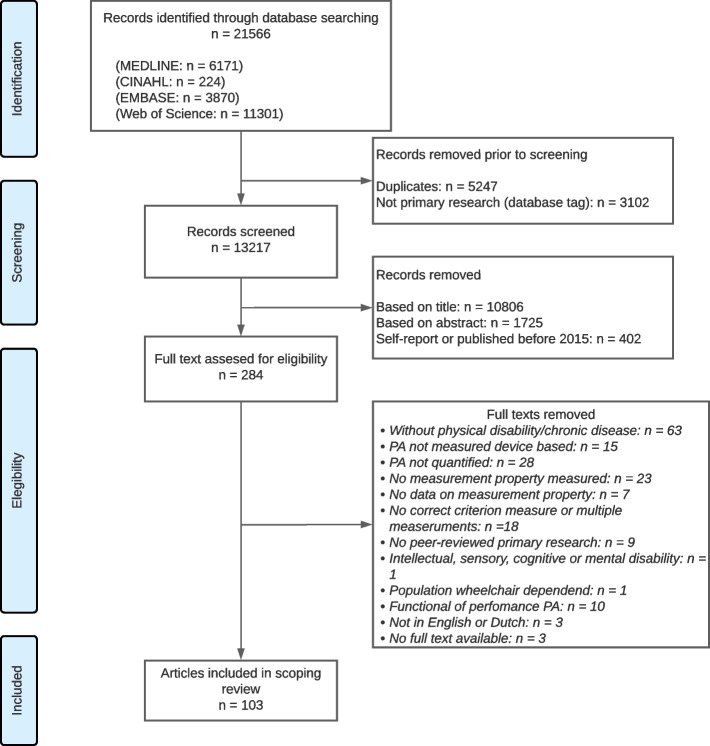
Flowchart of screening and review process of included studies on device-based instruments assessing physical activity. *n* = number of studies

Figure 1 shows a flowchart of the screening and review process. A total of 21566 records were identified through the search. After removing duplicates and publications categorized as non-primary research, 13219 records were screened on title. Based on title, we excluded 10752 records. We screened the remaining records on abstract, and excluded 1725 records. A further 403 records were excluded, as they were published before 2015 or used self-report measurement instruments for physical activity. The remaining 287 records were read in full. Of these, we excluded 184 records that did not meet the eligibility criteria, which resulted in a total of 103 studies included in this review. Agreement of the initial search and first update for title, abstract and full text screening was moderate (title phase: Cohen’s Kappa = 0.68, agreement = 78%; abstract phase: Cohen’s Kappa = 0.55, agreement = 82%; full text phase: Cohen’s Kappa = 0.57, agreement = 78%).

Characteristics of the included studies are shown in Table 1. In total, 23 different physical disabilities and/or chronic diseases were included in the studies. Most studies included people with stroke (*n* = 27) [40–66], chronic obstructive pulmonary disease (*n* = 11) [67–77] and multiple sclerosis (*n* = 10) [78–87]. Six studies included a mixed population of people with different physical disabilities and/or chronic diseases [23, 75, 77, 88–90]. Sample sizes ranged from 4 to 176, with a median of 28. The majority of studies were performed in Northern America (USA, *n* = 28 [51, 64, 69, 70, 72, 74, 76, 83–85, 91–107]; Canada, *n* = 10 [40, 47, 50, 52, 53, 89, 108–111]) and Western Europe (UK, *n* = 11 [78, 80, 82, 86, 112–118]; France, *n* = 8 [42–45, 55, 119]; the Netherlands, *n* = 6 [48, 75, 77, 120–122]; Germany, *n* = 4 [68, 87, 123, 124]; Switzerland, n = 4 [66, 81, 125, 126]; Denmark, *n* = 3 [127–129]; Belgium, *n* = 2 [67, 88]; Italy, *n* = 2 [56, 130]; Sweden, *n* = 2 [71, 79]; Ireland, *n* = 1 [131]; Portugal, *n* = 1 [132]). Only 14 studies were performed in other countries (Brazil, *n* = 6 [46, 49, 57, 62, 63, 133]; Japan, *n* = 4 [59, 73, 134, 135]; Australia, *n* = 3 [60, 90, 136]; Czech Republic, *n* = 1 [65]). Of the 103 included studies, 65 were performed in a laboratory setting with protocolled activities [23, 40–46, 49, 51–59, 61–66, 70, 72, 75, 78–80, 83, 86, 88–90, 92, 93, 95–97, 101, 103, 104, 107, 109, 111–115, 119, 120, 122, 123, 125, 126, 128–133, 137–139], 28 during free-living (activities of own choice) [50, 60, 67, 68, 71, 73, 76, 82, 87, 91, 94, 98–100, 102, 105, 106, 108, 110, 117, 121, 124, 127, 134–136, 140, 141], nine in a combined laboratory and free-living setting [47, 48, 69, 77, 81, 84, 85, 116, 118], and one in the home setting in which participants had to perform a set of protocolled activities [74]. Walking speed of the participants was on average slow, with speeds predominantly below 1.0 m/s. Supplementary file 4 provides an extended version of Table 1. This table provides extra information on important in- and exclusion criteria, the tasks performed, and criterion for valid measurement days and cases (for studies performed in a free-living setting).

**Table 1:** Descriptives of the 103 included studies

Author	Year	Country	Study design	Population	N	Study setting	% Male	Age (years)	Task	Walking speed (m/s)
Albaum et al. [108]	2019	Canada	Cross	iSCI	17	FL	76.5	62.0 (41.5—78.5)	Physical therapy & self-directed tasks	N.R
Alexander et al. [78]	2022	UK	Cross	MS	100	Lab	30.0	53.5 (47.8–58.0)	Circuit outdoors	Comfortable
Alharbi et al. [140]	2016	Australia	Cross	Coronary heart disease	28	FL	71.4	N.R	Free-living 4 days	N.R
Alothman et al. [91]	2020	USA	Long	DM2	30	FL	36.7	64.87 ± 5.99	Free-living 7 days (2x)	N.R
Anens et al. [79]	2023	Sweden	Cross	MS	30	Lab	30.0	49.2 ± 14.0	Circuit + sedentary activities	0.76 IQR 0.31 – 1.30 IQR 0.39
Arch et al. [92]	2018	USA	Cross	Amputation^a^	50	Lab	64–76	55.4 ± 10.1 – 58.6 ± 11.7	Circuit	0.95 ± 0.21—1.01 ± 0.19
Ata et al. [93]	2018	USA	Cross	Peripheral Aterial Disease	114	Lab	77.2	69.5 ± 13.1	6MWT	N.R
Balto et al. [83]	2016	USA	Cross	MS	45	Lab	N.R	46.7 ± 10.0	treatmill 500 steps	1.21 ± 0.27
Bianchini et al. [130]	2022	Italy	Cross	Parkinsons disease	47	Lab	67.0	66.3 ± 8.2	6MWT	Self-selected
Block et al. [84]	2017	USA	Cross	MS	82	Both	29.3	51.0 ± 13.7	2MWT and free-living 7 days	N.R
Block et al. [85]	2019	USA	Cohort	MS	61	Lab	28.0	50.0 ± 14.4	2 MWT	N.R
					31	FL	41.9	53.4 ± 11.7	Free-living 7 days	N.R
Blondeel et al. [67]	2020	Belgium	Cross	COPD	30	FL	61.0	66 ± 8	Free-living 14 days	N.R
Boeselt et al. [68]	2016	Germany	Cross	COPD	20	FL	85.0	66.4 ± 7.4	Free-living 3 days	N.R
Campos et al. [40]	2018	Canada	Cross	Stroke	33	Lab	69.7	64.9 ± 14.7	7 h on a single day	0.82 ± 0.27
Caron et al. [119]	2019	France	Cross	DM2	20	Lab	40.0	57.5 ± 8.4	Treadmill	0.50, 0.75, 1.00, 1.25 & 1.50
Cederberg et al. [104]	2021	USA	Cross	Parkinsons disease	29	lab	62.0	64.2 ± 6.4	6MWT + treadmill	1.03 ± 0.18
Chandrasekar et al. [112]	2018	UK	Cross	Polymyalgia rheumatica	27	Lab	11.0	69.2 ± 8.8	2 MWT & stairs test	1.19 (IQR 0.95–1.31)
Claridge et al. [120]	2019	Netherlands	Cross	Cerebral palsy	14	Lab	60.0	35.4 ± 13.1	Circuit	
Clay et al. [41]	2019	New Zealand	Cross	Stroke	19	Lab	42.0	65.6 ± 8.2	6MWT	Self-selected (0.97 ± 0.22)
Collins et al. [94]	2019	USA	Cross	Osteoarthritis (knee)	15	FL	33.0	68 ± 8	Free-living waking hours 28 days	N.R
Compagnat et al. [45]	2018	France	Corss	Stroke	35	Lab	N.R	64.6 ± 14.4	Circuit	0.6 ± 0.3
Compagnat et al. [44]	2019a	France	Cross	Stroke	35	Lab	N.R	64.6 ± 14.8	6MWT	Comfortable (0.56 ± 0.30)
Compagnat et al. [42]	2019b	France	Cross	Stroke	38	Lab	52.6	65.7 ± 13.5	Circuit	0.52 ± 0.28
Compagnat et al. [43]	2020	France	Cross	Stroke	26	Lab	N.R	64.6 (55.5–77.0)	6MWT	0.56 ± 0.3
Compagnat et al. [61]	2022	France	Cross	Stroke	26	Lab	61.5	63.5 (55.3—77.5)	6MWT	0.53 ± 0.30
Costa et al. [46]	2020	Brazil	Cross	Stroke	55	Lab	54.5	62.5 ± 14.9	2 MWT	0.7 ± 0.3
Coulter et al. [86]	2017	UK	Cross	MS	20	Lab	45.0	53.7 ± 7.4	Circuit	83.9 ± 25.1 steps/min
Daligadu et al. [109]	2018	Canada	Cross	Cardio-thorax surgery patients^b^	20	Lab	90.0	61.3 ± 10.2	6 MWT	0.7 ± 0.2
Daniel et al. [62]	2022	Brazil	Cross	Stroke	24	Lab	54.0	46.2 ± 12.0	Treadmill walking	0.22—0.89
Danilack et al. [69]	2015	USA	Cross	COPD	176	Both	99.0	72 ± 8	Circuit & free-living 14 days	0.97 ± 0.22
de Carvalho Lana et al. [133]	2021	Brazil	Cross	Parkinsons disease	34	Lab	76.5	66.8 ± 7.1	2MWT	Self-selected
Dhillon et al. [70]	2018	USA	Cross	Lung disease^c^	8	Lab	N.R	42.1 ± 17.1	Circuit	N.R
Douma et al. [121]	2018	Netherlands	Cross	Cancer	72	FL	63.0	63 ± 11.5	Free-living 14 days	N.R
Duclos et al. [47]	2019	Canada	Cross	Stroke	20	Both	65.0	53.9 ± 10.8	6MWT and Circuit at mall	1.02 ± 0.41 (6MWT) 0.86 ± 0.29 (Circuit)
Falter et al. [88]	2019	Belgium	Cross	Heart disease^d^, DM 1&2	40	Lab	80.0	61.9 ± 15.2	Cardiopulmonary exercise test, cycling ergometer	N.R
Fanchamps et al. [48]	2018	Netherlands	Cross	Stroke	25	Both	84.0	56 ± 12	Circuit	N.R
Faria et al. [49]	2019	Brazil	Cross	Stroke	30	Lab	70.0	62 ± 12	Circuit	Maximum speed 1.3 ± 1.0
Farmer et al. [90]	2022	Australia	Cross	Orthopedic, neurological and other	88	Lab	49.0	73 ± 11	Circuit indoors and outdoors	0.81 & 0.78 (in, outdoor)
Farooqi et al. [71]	2015	Swedem	Cross	COPD	19	FL	0.0	69.2 ± 6.0	Free-living 14 days	N.R
Femiano et al.[126]	2022	Switzerland	Cross	Cardiac rehabilitation patients^e^	22	Lab	N.R	56.6 ± 9.0	Physical therapy	N.R
Ferreira et al. [132]	2020	Portugal	Cross	Chronic pain	50	Lab	36.0	72.30 ± 6.76	Circuit	Self-selected & maximum walking speed
Garcia Oliveira et al. [63]	2021	Brazil	Cross	Stroke	50	Lab	64.0	62 (57—70)	10 mWT + TUG	0.88 (0.50—1.13)
Gustafsson et al. [128]	2022	Denmark	Cross	Lumbar spinal stenosis	30	Lab	63.0	76.2 ± 7.8	Circuit	Comfortable
Hei Chow et al. [60]	2023	Australia	Cross	Stroke	23	FL	65.0	74.8 ± 9.8	Free-living 7 days	N.R
Henderson et al. [64]	2021	USA	Cross	Stroke	21	Lab	48.0	64.0 ± 13.5	Physical therapy	0.33 (0.00—1.21)
					7	Lab	71.0	65.5 ± 8.3	Exercise training walking	0.49 (0.16–1.08)
Herkert et al. [122]	2019	Netherlands	Cross	Coronary artery disease	19	Lab	74.0	61.4 ± 6.9	Circuit and treadmill	N.R
				Heart failure	19	Lab	89.0	65.1 ± 6.6	Circuit and treadmill	N.R
Holubova et al. [65]	2022	Czech Republic	Cross	Stroke	24	Lab	62.5	58.95 ± 12.25	Circuit	N.R
Huber et al. [66]	2022	Switzerland	Cross	Stroke	20	Lab	65.0	63.1 ± 12.4	Circuit, outdoor	1.34 (0.77—1.47)
Hui et al. [50]	2018	Canada	RCT	Stroke	12	FL	58.0	62.6 ± 9.3	Free-living 3 days (fri-sun)	0.73 ± 0.27
Jao et al. [95]	2017	USA	Cross	DM (half with foot amputation)	31	Lab	N.R	56 ± 7.5	Circuit	Self-selected, 60 & 100 steps/min
Jayaraman et al. [96]	2016	USA	Cross	iSCI	8	Lab	87.5	48.5 ± 3.7	Circuit	N.R
Jayaraman et al. [51]	2018	USA	Cross	Stroke	8	Lab	60.0	55.6 ± 9.4	Circuit	N.R
				iSCI	10	Lab	87.5	48.5 ± 10.4	Circuit	N.R
Jimenez-Moreno et al. [113]	2019	UK	Cross	DM1	30	Lab	66.7	48 (25–72)	Circuit	N.R
Juen et al. [72]	2015	USA	Cross	pulmonary diseases^f^	28	Lab	42.8	N.R	6MWT	N.R
Klassen et al. [52]	2016	Canada	Cross	Stroke	43	Lab	70.0	65.0 ± 10.7	Circuit	Self-selected & 0.3–0.9 with increases of 0.1
Klassen et al. [53]	2017	Canada	Cross	Stroke	21	Lab	N.R	55 ± 10	Physical therapy	0.41 ± 0.27
Ladlow et al. [114]	2017	UK	Cross	Amputation^g^	20	Lab	N.R	32 ± 5 (unilateral) 29 ± 4 (bilateral)	Treadmill	0.48, 0.67, 0.89, 1.12, 1.34
Ladlow et al. [115]	2019	UK	Cross	Amputation^g^	19	Lab	100.0	30.4 ± 4.6	Treadmill	0.48, 0.67, 0.89, 1.12, 1.34
Lai et al. [97]	2020	USA	Cross	Parkinsons disease	31	Lab	N.R	64.3 ± 6.3	Circuit & treadmill	1.05 ± 0.16
Lamont et al. [137]	2018	Australia	Cross	Parkinsons disease	33	Lab	64.0	68.8 ± 8	Circuit	Self-selected & 60, 80, 100, 120, 140 steps/min
Larkin et al. [131]	2016	Ireland	Cross	Rheumatoid arthritis	20	Lab	15.0	55 ± 14	Circuit & treadmill	Self-selected pace
Lavelle et al. [80]	2021	UK	Cross	MS	19	Lab	31.6	52.1 ± 11.9	Circuit	N.R
Mahendran et al. [54]	2016	Australia	Cross	Stroke	15	Lab	53.3	63.4 ± 8.3	6MWT, circuit & treadmill	Slow (0.31 ± 0.11), comfortable (0.42 ± 0.17) & fast (0.54 ± 0.25)
Mandigout et al. [55]	2017	France	Cross	Stroke	24	Lab	62.5	68.2 ± 13.9	Circuit	N.R
McGinley et al. [110]	2015	Canada	Cross	DM2	35	FL	60.0	62.8 ± 7.8	Free-living 14 days	N.R
Miyamoto et al. [73]	2018	Japan	Cross	COPD	11	FL	91.6	76.6 ± 6.9	Free-living 7 days	N.R
Negrini et al. [56]	2020	Italy	Cross	Stroke	43	Lab	62.8	61.3 ± 14.95	Circuit	0.75 ± 0.32
Nishida et al. [134]	2020	Japan	Cross	DM2	51	FL	45.1	70 ± 5	Free-living 12–16 days	N.R
O'Brien et al. [116]	2020	UK	Cross	Rheumatoid arthritis	22	Lab	14.0	53.7 ± 12.5	Circuit	N.R
				Rheumatoid arthritis	100	FL	29.0	58.5 ± 12.1	Free-living 7 days	N.R
O'Neill et al. [117]	2017	UK	Cross	Bronchiectasis	55	FL	40.0	60 ± 10	Free-living 7 days	N.R
Pham et al. [123]	2017	Germany	Long	Parkinsons disease	20	Lab	52.4	66.4 ± 9.0	Circuit	N.R
Polese et al. [57]	2019	Brazil	Cross	Stroke	37	Lab	75.7	62 ± 11	Circuit	0.9 ± 0.3 Comfartable 1.3 ± 0.6 Fast
Polhemus et al. [81]	2023	Switzerland	Cross	MS	45	Both	35.6	46 (IQR 40—51)	Circuit + Free-living 14 days	109 (61–146) steps/min
Popp et al. [125]	2019	Switzerland	Cross	iSCI	30	Lab	70.0	54.1 ± 11.9	Circuit	N.R
Prieto-Centurion et al. [74]	2016	USA	Cross	COPD	4	Home	100.0	69 ± 10	6MWT	N.R
Roberts-Lewis et al. [118]	2022	UK	Long	Progressive muscle diseases	20	Lab	N.R	N.R	Circuit	N.R
					56	FL	44.6	44.7 ± 15.1	Free-living 7 days	N.R
Rockette-Wagner et al. [105]	2021	USA	Long	Inflammatory myopathy	50	FL	40.0	48.6 ± 15.4	Free-living 7 days	N.R
Rossi et al. [98]	2018	USA	Cross	Endometrial cancer	25	FL	0.0	62 ± 9	Free-living 30 days	N.R
Salih et al. [138]	2016	Australia	Cross	Amputation^h^	21	Lab	71.4	59.4 ± 11.5	Circuit	N.R
Saygin et al. [106]	2022	USA	Long	Myositis	24	FL	29.2	46.2 ± 14.4	Free-living 7 days	N.R
Schaffer et al. [58]	2017	USA	Cross	Stroke	24	Lab	58.3	54 ± 13.4	Circuit	0.72 ± 0.40
Semanik et al. [99]	2020	USA	RCT	Chronic knee symptoms	35	FL	31.0	52 ± N.R	Free-living 7 days	N.R
Shimizu et al. [59]	2018	Japan	Cross	Stroke	10	Lab	50.0	57.5 ± 16.2	circuit	0.98 ± 0.36
Shoemaker et al. [100]	2017	USA	Cohort	Heart failure	16	FL	56.3	64.9 ± 11.3	Free-living 7 days	N.R
Smith et al. [101]	2019	USA	Cross	Amputation^g^	32	Lab	66.0	49.7 ± 14.0		Self-selected
Smith & Guerra [107]	2021	USA	Cross	Amputation^g^	35	Lab	54.3	48.5 ± 14.8	2MWT	1.23 ± 0.22
Stuart et al. [82]	2020	UK	Long	MS	56	FL	52.0	53.6 ± 8.0	Free-living 2–7 days	N.R
Takasaki[135]	2017	Japan	Cross	Lower back pain	15	FL	40.0	22.1 ± 4.3	Free-living 14 days	N.R
Taoum et al. [139]	2020	France	Cross	Peripheral artery disease	23	Lab	N.R	60.0 ± 10.0	Circuit	Self-selected
Thorup et al. [127]	2017	Denmark	Cross	Heart disease^i^	24	FL	91.6	67.0 ± 10.0	Day at hospital and day at home	N.R
Treacy et al. [23]	2017	Australia	Cross	Diverse motor disabilities^j^	166	Lab	55.0	80 ± 11	6MWT	0.42 ± 0.22
Ummels et al. [75]	2018	Netherlands	Cross	CVD, cancer, COPD, osteoarthritis, chronic pain	130	Lab	43.6	61.5 ± 11.1	Circuit	1.3 ± 0.3
Van Blarigan et al. [102]	2017	USA	Cross	prostate cancer	22	FL	100.0	66 (56–83)	Free-living 7 days	N.R
Van der Weegen et al. [77]	2015	Netherlands	Cross	COPD & DM2	9	Lab	N.R	60.9 ± 7.1 (Lab)	Treadmill	0.56 + 0.14 every 3 min
				COPD & DM2	12	FL	N.R	61.6 ± 9.2 (free-living)	Free-living 6–7 consecutive days	N.R
Van Laerhoven et al. [124]	2022	Germany	Cross	DM	28	FL	65.0	74.8 ± 9.8	Free-living 2 days	N.R
Vetrovsky et al.[141]	2019	Australia	Cross	Heart failure	15	FL	60.0	65.5 ± 12.6	Free-living 3 days	N.R
Wagner et al. [105]	2022	Denmark	Cross	RA	30	Lab	17.0	61 (50–74)	Treadmill	0.69 – 1.39, increments of 0.14
Webber & John[89]	2016	Canada	Cross	Geriatric rehabilitation^k^	38	Lab	10.5	83.2 ± 7.1	Hallway walk	0.4 ± 0.2
Webster et al. [76]	2021	USA	Cross	COPD	59	FL	52.5	69.4 ± 7.8	Free-living 7 days	N.R
Wendel et al. [103]	2018	USA	Cross	Parkinsons disease	33	Lab	58.0	65.5 ± 9.4	Circuit	Comfertable and fast pace
Zbogar et al. [111]	2016	Canada	Cohort	SCI	35	Lab	70.0	48.9 ± 18.3	1 day of rehab	0.75 ± 0.39
Zhai et al. [87]	2020	Germany	Cross	MS	67	FL	37.3	42.9 ± 10.9	Free-living 7 days	N.R
Yu et al. [136]	2022	Australia	Long	OA	65	FL	54.0	61.3 ± 5.99	Free-living 7 days	N.R

^a^Lower limb, below knee, unilateral

^b^Post-coronay artery bypass graft surgery, aortic calve repair, mitral valve replacement

^c^COPD, interstitial lung disease, cystic fibrosis

^d^Ischemic heart disease, valvular heart disease

^e^coronary infarction, hypertensive cardiomyopathy, valvular cardiopathy, type-a aortic dissection

^f^COPD, congestive heart failure, other pulmonary diseases requiring pulmonary function test

^g^Lower limb, below and above knee, unilateral and bilateral

^h^Lower limb, below and above knee, unilateral

^i^Acute coronary syndrome, heart failure, coronary artery bypass grafting or valve surgery

^j^Fractured hip, pelvis, lower limb orthopedic surgery, stroke, TIA, neurological event, decreased mobility post medical or non-orthopedic surgical event, post fall with no lower limb fracture, other

^k^respiratory/infection, orthopedic, falls/decreased mobility, neurological, gastrointestinal, cancer, endocrine

*Cross* Cross sectional, *Long* Longitudinal, *RCT* Randomized controlled trial, *CAD* Coronary artery disease, *COPD* Chronic obstructive pulmonary disease, *CVD* Cardiovascular disease, *DM1* Diabetes mellitus type 1, *DM2* Diabetes mellitus type 2, *DM* Diabetes mellitus (further details unknown), *iSCI* Incomplete spinal cord injury, *MS* Multiple sclerosis, *PAD* Pulmonary artery disease, *SCI* Spinal cord injury, *Both* Both free-living and laboratory with protocolled activities, *FL* Free-living, *Home* Home situation with protocolled activities, *Lab* Laboratory setting with protocolled activities, *MWT* Minutes walking test, *mWT* Meter walking test, *TUG* Timed up and go-test, *N.R.* Not reported

In total, 78 different PA devices from 43 different companies were studied on their measurement properties. In 39 studies multiple devices were used and compared [23, 43, 44, 46, 49, 51, 54, 55, 57, 58, 63, 64, 67, 70, 75, 79–81, 83, 84, 89, 92–97, 101, 103, 107, 112, 115, 116, 118, 122, 132, 133, 137, 141]. Twenty-three devices were research-grade and 55 were consumer-grade. The most frequently studied research-grade devices were from the companies ActiGraph (*n* = 28 studies) [23, 40, 43–45, 49, 51, 55, 61, 64, 76, 79, 81, 84, 89, 93–96, 104, 105, 107, 108, 112, 114–116] and PAL technology (*n* = 8 studies) [23, 54, 86, 91, 95, 116, 131, 138]. The most frequently studied consumer-grade devices were from the companies Fitbit (*n* = 39 studies) [23, 41, 46, 47, 50, 52, 53, 58, 60, 64, 65, 67, 74, 75, 80, 81, 83–85, 90, 92, 94, 97–99, 101–103, 106, 109, 112, 118, 122, 127, 133, 136, 137, 140, 141] and Garmin (*n* = 10 studies) [23, 58, 66, 80, 97, 101, 107, 130, 137, 141].

With respect to measurement properties, 97 studies determined validity [23, 40–90, 92–110, 112, 114–129, 131–134, 136–138, 140, 141], 11 studies determined reliability [46, 54, 58, 66, 91, 105, 106, 111, 113, 118, 135] and six study determined responsiveness [82, 100, 105, 106, 118, 136]. The measurement properties of 14 different PA outcomes were studied. Step count was the most frequently studied PA outcome (*n* = 68) [23, 40, 41, 46, 47, 50, 52–54, 56–58, 63–69, 74, 75, 79–86, 89–98, 101–109, 111, 112, 116–118, 121, 123, 124, 126–133, 136, 137, 140, 141], followed by energy expenditure (*n* = 19) [42, 43, 45, 49, 51, 55, 61, 62, 70, 71, 82, 88, 96, 114, 115, 119, 122, 125, 134] and activity time (*n* = 15) [48, 54, 68, 80–82, 86, 91, 95, 100, 116, 117, 120, 131, 138]. In the majority of studies (*n* = 60), PA was measured by means of only walking tasks or by using walking-related PA outcomes (e.g. steps, walked distance) [23, 40, 41, 43, 44, 46, 47, 49, 52–54, 56–58, 61, 62, 64–67, 69, 72, 74, 75, 77, 78, 83–85, 89, 90, 92, 93, 97, 98, 101, 103, 104, 107–109, 112, 113, 115, 119, 121, 123, 126–130, 132, 133, 136–139, 141].

The proprietary algorithm of the instrument was most frequently used, or the algorithm used was not reported at all. A population-specific custom algorithm was used in three research-grade and three consumer-grade devices. Devices were positioned at 15 different body positions, with the positions at the ankle, thigh, waist and wrist as most common. One device (Medtronic ICD/CRT device) was a type of pacemaker, and was surgically implanted in patients with heart failure. Validity was measured using 21 different statistical methods, reliability with three different methods, and responsiveness with five methods.

Table 2 provides an overview of the measurement properties of the research-grade devices, per PA outcome, study population, device properties (placement of the device, used algorithms) and outcome (used statistical test, result). Table 3 provides the same overview for the consumer-grade devices. Supplementary files 5 and 6 contain a more in-depth version of both tables, with extra information such as epoch length, sampling rate and results per condition.

**Table 2:** Overview of research grade devices evaluated on their measurement properties in the 52 studies

								Result	
**Type**	**PA outcome**	**Population**	**Study**	**Measurement property**	**Criterion**	**Placement**	**Algorithm**	**Test**	**Outcome**
**ActiGraph**									
GT3	EE	Amputation	Ladlow [114]	CV	IC	Waist (SRL)	Cust	Pearson's r	r: 0.86—0.94
			Ladlow [115]	CV	IC	Waist (SRL)	Cust	Pearson's r	r: 0.92 – 0.96
		iSCI	Jayaraman [96]	CV	IC	Ankle	Prop (Freedson)	ANOVA	Sed: *p* < .05; low & high: *p* > .05
						U-arm	Prop (Freedson)	ANOVA	Sed & low: *p* < .05; high: *p* > .05
						Waist	Prop (Freedson)	ANOVA	Sed & low: *p* < .05; high: *p* > .05
			Jayaraman [51]	CV	IC	Ankle	Prop (Freedson)	Kruskal wallis	Lying, sitting & standing: *p* < 0.5
						U-arm	Prop (Freedson)	Kruskal wallis	Lying, sitting, standing & 50SWT: *p* < .05
						Waist	Prop (Freedson)	Kruskal wallis	All *p* < .05
		Stroke	Compagnat [45]	CV	IC	Ankle (ua)	N.R	Pearson's r	*r *= 0.41
						Waist	N.R	Pearson's r	*r* = 0.15
						Wrist (ua)	N.R	Pearson's r	*r* = 0.12
			Compagnat [43]	CV	IC	Waist	Prop	Pearson's r	*r* = 0.19
							Cust	Pearson's r	*r* = 0.44
			Compagnat [61]	CV	IC	Ankle	Cust (multiple)	LoA	-69.1 [-148.7; 10.5] -8.3 [-1.1; 17.8]
			Fairia [49]	CV	IC	Ankle (a)	Prop (work-energy teorem)	Pearson's r	*r* = 0.04
							Prop (Freedson)	Pearson's r	*r* = 0.04
							Prop (combined)	Pearson's r	*r* = 0.37
			Jayaraman [51]	CV	IC	Ankle (a)	Prop (Freedson)	Kruskal wallis	Lying, sitting, standing & STS: *p* < 0.5
						Ankle (ua)	Prop (Freedson)	Kruskal wallis	Lying, sitting, standing & STS: *p* < 0.5
						U-arm (a)	Prop (Freedson)	Kruskal wallis	Lying, sitting & standing: *p* < 0.5
						U-arm (ua)	Prop (Freedson)	Kruskal wallis	Lying, sitting & standing: *p* < 0.5
						Waist (a)	Prop (Freedson)	Kruskal wallis	Lying, sitting & standing: *p* < 0.5
						Waist (ua)	Prop (Freedson)	Kruskal wallis	Lying, sitting & standing: *p* < 0.5
			Mandigout [55]	CV	IC	Ankle (ua)	N.R	Spearman's rho	rho = 0.19
						Ankle (a)	N.R	Spearman's rho	rho = 0.21
						Waist	N.R	Spearman's rho	rho = 0.04
						Wrist (ua)	N.R	Spearman's rho	rho = 0.20
						Wrist (a)	N.R	Spearman's rho	rho = 0.08
	Steps	DM	Jao [95]	CV	DO	Waist	Prop	Accuracy (%)	43.0—81.4%
		Inflammatory myopathy	Rockette-Wagner [105]	Con V	F Tests & F SR	Waist	Prop	Pearson's r	r: -0.42—0.66
				TRT R		Waist	Prop	ICC	ICC = 0.92 (CI 0.86 – 0.96)
				Resp	SR	Waist	Prop	Pearson's r	*r* = 0.47 (CI 0.12—0.71)
		iSCI	Albaum [108]	CV	DO	Ankle (la)	Prop	ICC	ICC: 0.15 – 0.99
			Jayaraman [96]	CV	DO	Ankle	Prop (Freedson)	ANOVA	*p* > .05
						U-arm	Prop (Freedson)	ANOVA	*P* < .05
						Waist	Prop (Freedson)	ANOVA	*p* < .05
		MS	Block [84]	CV	DO	Waist	Prop	ICC	ICC = 0.76 (CI 0.63–0.85)
			Polhemus [81]	CV	DO	Waist	Prop	CCC	CCC = 0.68 (CI 0.37—0.82)
							Prop (+ LFE filter)	CCC	CCC = 0.73 (0.13—0.84)
		Multi	Treacy [23]	CV	DO	Waist	Prop	ICC	ICC = 0.123 (CI -0.071–0.355)
			Webber & John [89]	CV	DO	Ankle	Prop	ICC	ICC = 0.682 (CI -0.211–0.895)
							Prop (+ LFE filter)	ICC	ICC = 0.938 (CI 0.870–0.969)
						Waist	Prop	ICC	ICC = -0.051 (CI -0.191–0.153)
							Prop (+ LFE filter)	ICC	ICC = 0.829 (CI 0.329–0.936)
		Osteoarthritis	Collins [94]	CV	Acc	Wrist		ICC	ICC = 0.602
		PD	Cederberg [104]	CV	DO	Wrist (ua)	Prop	Mean difference	62—76 steps
						Wrist (a)	Prop	Mean difference	32—66 steps
		Polymyalgia rheumatica	Chandrasekar [112]	CV	DO	Waist	Prop	LoA	Walking: 141 + (0.5*mean count) [110 + (0.5*mean count)]
							Prop (+ LFE filter)	LoA	Walking: 20 [-40; 81]
							Prop	LoA	Stairs: 4 [-4; 12]
							Prop (+ LFE filter)	LoA	Stairs: 0 [-5; 5]
		RA	O'Brien [116]	CV	DO	Thigh	Prop	LoA	-30 [-116; 57]
		Stroke	Campos [40]	CV	Acc	Ankle	Prop	ICC	ICC = 0.80 (CI 0.63–0.90)
							Prop (+ LFE filter)	ICC	ICC = 0.76 (CI 0.56–0.87)
						Waist	Prop	ICC	ICC = 0.70 (CI 0.47–0.84)
							Prop (+ LFE filter)	ICC	ICC = 0.82 (CI 0.66–0.90)
			Henderson [64]	CV	DO	Ankle (a)	Prop	ICC	ICC: 0.57—0.81
							Prop (+ LFE filter)	ICC	ICC: 0.84—0.96
						Ankle (ua)	Prop	ICC	ICC: 0.84—0.86
							Prop (+ LFE filter)	ICC	ICC: 0.77—0.97)
	Intensity time	Osteoarthritis	Collins [94]	CV	Acc	Wrist	Cut off: counts < 200	% bias	Sed: -66%
							Cut off: 1924 counts/min, bouts of 10 min	Difference	MVPA: + 281 min
	Activity time	DM	Jao [95]	CV	DO	Waist	Prop	Accuracy (%)	41.8 – 100%
		RA	O'Brien [116]	CV	DO	Thigh	Prop	LoA	-0.3 [-1.2; 0.6] min—0.2 [-0.7; 1.1] min
	Distance walked	Stroke	Compagnat [44]	CV	DO	Ankle (ua)	Prop	Pearson's r	*r* = 0.95
						Ankle (a)	Prop	Pearson's r	*r* = 0.93
						Waist	Prop	Pearson's r	*r* = 0.86
						Wrist (ua)	Prop	Pearson's r	*r* = 0.79
						Wrist (a)	Prop	Pearson's r	*r* = 0.81
		Peripheral artery disease	Taoum [139]	CV	GPS	Hip	Cust	MAPE	11.9—18.8
	Counts	Amputation	Ladlow [114]	CV	IC	Waist (SRL)	Prop	Pearson's r	r: 0.82–0.92
						Waist (LRL)	Prop	Pearson's r	r: 0.76 – 0.80
						Waist (Sp)	Prop	Pearson's r	r: 0.68 – 0.80
	MET	Stroke	Jayaraman [51]	CV	IC	Ankle (a)	Prop (Freedson)	Kruskal wallis	STS: *p* < .05
						Ankle (ua)	Prop (Freedson)	Kruskal wallis	50SWT, 6MWT & STS: *p* < .05
						U-arm (a)	Prop (Freedson)	Kruskal wallis	All *p* > .05
						U-arm (ua)	Prop (Freedson)	Kruskal wallis	All *p* > 0.5
						Waist (a)	Prop (Freedson)	Kruskal wallis	All *p* > .05
						Waist (ua)	Prop (Freedson)	Kruskal wallis	All *p* > .05
		iSCI	Jayaraman [51]	CV	IC	Ankle	Prop (Freedson)	Kruskal wallis	Lying & STS *p* < .05
						U-arm	Prop (Freedson)	Kruskal wallis	50SWT *p* < .05
						Waist	Prop (Freedson)	Kruskal wallis	50SWT, 6MWT & STS *p* < .05
	Vector magnitude	Inflammatory myopathy	Rockette-Wagner [105]	Con V	F Tests & F SR	Waist	Prop	Pearson's r	r: -0.35—0.60
				TRT R		Waist	Prop	ICC	ICC = 0.80 (CI 0.62 – 0.89)
				Resp	SR	Waist	Prop	Pearson's r	*r* = 0.53 (CI 0.20—0.75)
GTX9	Steps	Amputation	Smith & Guerra [107]	CV	DO	Ankle	N.R	ICC	ICC = 0.111 (CI -0.202—0.418)
						Wrist	N.R	ICC	ICC = 0.005 (CI -0.256—0.299)
		MS	Anens [79]	CV	DO	Waist	Prop	Spearman's rho	rho: 0.74—0.79
							Prop (+ LFE filter)	Spearman's rho	rho: 0.85—0.93
		PAD	Ata [93]	CV	DO	Waist	Prop	% error	-3.1 ± 10.3%
	Sedentary time	COPD	Webster [76]	CV	Acc	Waist	Prop (multiple)	CCC	CCC: 0.614 -0.838
		MS	Anens [79]	CV	DO	Waist	Prop	Spearman's rho	rho: 0.18—0.39
							Prop (+ LFE filter)	Spearman's rho	rho: 0.16—0.38
**Pal Technologies**									
ActivPAL	Steps	DM	Alothman [91]	TRT R		Thigh	Prop	ICC	ICC = 0.91
			Jao [95]	CV	DO	Thigh	Prop	Accuracy (%)	90.7 – 98.5%
		Multi	Treacy [23]	CV	DO	Thigh	Prop	ICC	ICC = 0.781 [CI 0.231; 0.911]
		RA	Larkin [131]	CV	DO	Thigh	Prop	Pearson's r	r = 0.94 [CI 0.86; 0.98]
		Stroke	Mahendran [54]	CV	DO	Thigh	Prop	ICC	ICC: 0.72 – 0.99
				TRT R		Thigh	Prop	ICC	ICC: 0.66 – 0.98
	Activity time	Amputation	Salih [138]	CV	DO	Thigh (a)	N.R	LoA	Walking: 0.11 [-0.43; 0.66] sec (ue)
						Thigh (ua)	N.R	LoA	Walking: 0.004 [-0.09; 0.10] sec (ue)
		DM	Alothman [91]	TRT R		Thigh	Prop	ICC	ICC: 0.74 – 0.90
			Jao [95]	CV	DO	Thigh	Prop	Accuracy (%)	96.6 – 100%
		RA	Larkin [131]	CV	DO	Thigh	Prop	Pearson's r	r: 0.74 – 0.93
		Stroke	Mahendran [54]	CV	DO	Thigh	Prop	ICC	ICC = 0.99
								APE	0.3 – 3.2%
				TRT R		Thigh	Prop	ICC	ICC: 0.66 – 0.98
								APE	3.3 – 6.5%
	MET	Stroke	Mahendran [54]	TRT R		Thigh	Prop	ICC	ICC: 0.65 – 0.99
ActivPAL3	Steps	MS	Coulter [86]	CV	DO	Thigh	Prop	LoA	-4.7 [-22.88; 13.47] (ue)
		RA	O'Brien [116]	CV	DO	Thigh	Prop	LoA	-30 [-116; 57] (ue)
	Activity time	MS	Coulter [86]	CV	DO	Thigh	Prop	LoA	-4.6 [-26.1; 17.0] – 1.1 [-1.12; 3.34] sec
		RA	O'Brien [116]	CV	DO	Thigh	Prop	LoA	-0.3 [-1.2; 0.6] – 0.2 [-0.7; 1.1] min
**Modus Health**									
StepWatch 3	Steps	Amputation	Arch [92]	CV	DO	Ankle (a)	N.R	ICC	ICC: 0.90 – 0.99
		Multiple	Treacy [23]	CV	DO	Ankle	Prop	ICC	ICC = 0.982 [CI 0.975; 0.986]
			Webber & John [89]	CV	DO	Ankle	Prop	ICC	ICC = 0.960 [CI 0.924; 0.979]
		Stroke	Garcia Oliveira [63]	CV	DO	Ankle (la)	Prop	Spearman's rho	rho: 0.963—0.994
			Henderson [64]	CV	DO	Ankle (a)	Prop	ICC	ICC: 0.92—0.96
						Ankle (ua)	Prop	ICC	ICC: 0.97—0.97
	Distance walked	Peripheral artery disease	Taoum [139]	CV	GPS	Ankle	Cust	MAPE	16.7 ± 10.7
StepWatch 4	Steps	Amputation	Smith & Guerra [107]	CV	DO	Ankle	N.R	ICC	ICC = 0.967 (CI 0.929—0.984)
**Body Media**									
Sensewear armband	EE	Chronic lung disease	Dhillon [70]	CV	IC	U-arm	Prop	LoA	-1.26 [-4.71; 2.19] – 0.56 [-1.68–2.80]
		MS	Stuart [82]	Con V	F Tests & F SR	Arm	Prop	Spearman's rho	rho: -.0412—0.365
				Resp	F Tests & F SR	Arm	Prop	Spearman's rho	rho: -0.196—0.168
		Stroke	Compagnat [42]	CV	IC	U-arm (ua)	Prop	Pearson's r	r: 0.48 – 0.81
			Mandigout [55]			U-arm (a)	N.R	Spearman's rho	rho = 0.61
						U-arm (ua)	N.R	Spearman's rho	rho = 0.45
	Steps	MS	Stuart [82]	Con V	F Tests & F SR	Arm	Prop	Spearman's rho	rho: -0.325—0.305
				Resp	F Tests & F SR	Arm	Prop	Spearman's rho	rho: -0.170—0.250
	Activity time	MS	Stuart [82]	Con V	F Tests & F SR	Arm	Prop	Spearman's rho	rho: -0.640—0.493
				Resp	F Tests & F SR	Arm	Prop	Spearman's rho	rho: -0.128—0.272
	Distance walked	Stroke	Compagnat [44]	CV	DO	U-arm (a)	Prop	Pearson's r	*r* = 0.72
						U-arm (ua)	Prop	Pearson's r	*r* = 0.68
	MET	MS	Stuart [82]	Con V	F Tests & F SR	Arm	Prop	Spearman's rho	rho: -0.343—0.316
				Resp	F Tests & F SR	Arm	Prop	Spearman's rho	rho: -0.191—0.295
	PA composite score	MS	Stuart [82]	Con V	F Tests & F SR	Arm	Prop	Spearman's rho	rho: -0.444—0.376
				Resp	F Tests & F SR	Arm	Prop	Spearman's rho	rho: -0.110—0.356
Sensewear Pro2	Steps	Stroke	Mahendran [54]	CV	DO	U-arm (a)	N.R	APE	21.9 – 66.8%
				TRT R		U-arm (a)	N.R	APE	2.2 – 38.5%
	MET	Stroke	Mahendran [54]	TRT R		U-arm (a)	N.R	APE	17.8 – 26.8%
**Activ8**									
Activ8	Activity time	CP	Claridge [120]	CV	DO	Thigh (frontal) (la)	N.R	Spearman's rho	rho: -0.04 – 0.86
						Thigh (lateral 2 cm) (la)	N.R	Spearman's rho	rho: 0.49 – 0.99
						Pocket	N.R	Spearman's rho	rho: 0.14 – 0.79
		Stroke	Fanchamps [48]	CV	DO	Thigh (frontal)	N.R	% time difference	-3.8 – 6.5%
	Steps	Multi	Ummels [75]	CV	DO	Pocket	N.R	Pearson's r	r = 0.24
**Philips**									
Actical	EE	Chronic lung disease	Dhillon [70]	CV	IC	Wrist	Prop	LoA	-3.4 [-6.4; -0.4]—-0.8 [-1.6; 0.0]
		Stroke	Mandigout [55]	CV	IC	Ankle (a)	N.R	Spearman's rho	rho = 0.30
						Ankle (ua)	N.R	Spearman's rho	rho = 0.20
						Waist	N.R	Spearman's rho	rho = -0.01
						Wrist (a)	N.R	Spearman's rho	rho = -0.19
						Wrist (ua)	N.R	Spearman's rho	rho = -0.27
	Activity kilocounts	SCI	Zbogar [111]	TRT R		Wrist	N.R	Pearson's r	*r* = 0.74 [CI 0.54; 0.86]
	Steps	SCI	Zbogar [111]	TRT R		Waist	N.R	Pearson's r	*r* = 0.84 [CI 0.70; 0.92]
**Axivity**									
AX3/AX6	Steps	Cardiac rehab	Femiano [126]	CV	DO	Wrist	Cust	MAPE	4.1—143.0%
		Lumbar spinal stenosis	Gustafsson [128]	CV	DO	Lower back	Prop	ICC	ICC: -0.04—1.00
						Thigh	Prop	ICC	ICC: -0.16—1.00
						Waist	Prop	ICC	ICC: -0.10—1.00
						Wrist	Prop	ICC	ICC: -0.10—0.99
**McRoberts**									
Dynaport	EE	Stroke	Daniel [62]	CV	IC	Lower back	Prop	ICC	ICC: 0.77—0.94
Dynaport Hybrid	Steps	PD	Pham [123]	CV	DO	Lower back	Cust	Kappa	k: 0.70 – 0.71
**Vandrico Inc**									
Metria-IH1	EE	iSCI	Jayaraman [96]	CV	IC	U-arm	Prop	ANOVA	All *p* > .05
			Jayaraman [51]	CV	IC	U-arm	Prop	Kruskal wallis	All *p* > .05
		Stroke	Jayaraman [51]	CV	IC	U-arm (a)	Prop	Kruskal wallis	All *p* > .05
						U-arm (ua)	Prop	Kruskal wallis	STS: *p* < .05
	Steps	iSCI	Jayaraman [96]	CV	DO	U-arm	Prop	ANOVA	*p* < 0.05
	MET	iSCI	Jayaraman [51]	CV	IC	U-arm	Prop	Kruskal wallis	All *p* > .05
		Stroke	Jayaraman [51]	CV	IC	U-arm (a)	Prop	Kruskal wallis	50SWT & STS: *p* < .05
						U-arm (ua)	Prop	Kruskal wallis	STS: *p* < .05
**Activinsight**									
GENEactive	Raw acceleration (ENMO—mg)	Myotonic dystrophy type 1	Jimenez-Moreno et al. [113]	TRT R		Wrist & Ankle	N.A	ICC	0.86 [95% CI 0.74; 0.93] to 0.97 [95% CI 0.95; 0.99]
**CamNtech**									
Actiheart	EE	Amputation	Ladlow [115]	CV	IC	Chest	Branched-Model equation	Pearson's r	r 0.81 – 0.86
**Espruino**									
Bangle.js	Steps	DM	Van Laerhoven [124]	CV	Acc	Wrist	Custom (open source)	LoA	-566.7 [-4111.5—2978.0]—17.48 [-211.5—246.5]
**Garcia Oliveira et al**									
AMoR	Steps	Stroke	Garcia Oliveira [63]	CV	DO	Thigh	N.R	ICC	ICC: 0.999—0.999
					Acc	Thigh	N.R	ICC	ICC: 0.981—0.985
	Sedentary time	Stroke	Garcia Oliveira [63]	CV	DO	Thigh	N.R	ICC	ICC = 0.960 (CI 0.929—0.977)
**Maastricht Instruments BV**									
MOX (1.01)	Counts	Multi	Van der Weegen [77]	CV	Acc	Lower back		Pearson's r	*r* = 0.98 (range 0.95—1.00)
						Lower back		Spearman's rho	rho = 0.82 (range 0.60—0.94)
	Intensity time	Multi	Van der Weegen [77]	CV	Acc	Lower back		LoA	-2.3—-0.5
**Medtronic**									
ICD/CRT device	Activity time	Heart failure	Shoemaker [100]	CV	Acc	Chest (internal)	Prop	LoA	-0.77 [-2.71; 1.17] hours/day (ue)
				Resp	Acc	Chest (internal)	Prop	LoA	0.19 [-0.79; 1.17] hours/day (oe)
**StepsCounts**									
PiezoRX	Steps	MS	Anens [79]	CV	DO	N.R	N.R	Spearman's rho	rho: 0.82—0.99
**Xsens**									
MTw	EE	DM	Caron [119]	CV	IC	Lower back	Bouten's equation	LoA	-1.17 [-6.45; 4.14] W/kg (oe)
**ZurichMOVE**									
JUMP	EE	iSCI	Popp [125]	CV	IC	Multiple	Cust	Pearson's r	*r* = 0.92

Ordering on number of studies evaluating manufacturer. This is a condensed version of the more detailed table in Supplementary file 5

*EE* Energy expenditure, *MET* Metabolic equivalent, *PAL* Physical activity level, *CAD* Coronay artery disease, *COPD* Chronic obstructive pulmonary disease, *DM* Diabetes mellitus, *iSCI* incomplete spinal cord injury, *MS* Multiple sclerosis, *PAD* Pulmonary artery disease, *PD* Parkinson's disease, *RA* Rheumatoid arthritis, *SCI* Spinal cord injury, *CV* Criterion validity, *Con V* Construct validity, *Resp* Responsiveness, *TRT R* Test–retest reliability, *Acc* Accelerometer, *DLW* Doubly labelled water, *DO* Direct observation, *F Tests* Functional tests, *F SR* Functional Self-report, *IC* Indirect calorimetry, *SR* Self-report, *Q* questionnaire, *(a)* Affected side, *(b)* both affected and unaffected side, *(la)* Less affected side, *(LRL)* Longest residual limb, *(SRL)* shortest residual limb, *(ua)* unaffected side, *Cust* Custom algorithm, *LFE* Low frequency effect, *N.R.* Not reported, *Prop* Proprietary algorithm, *TEE* Total energy expenditure, *APE* Absolute percentage error, *CCC* Concordance correlation coefficients, *ICC* Intraclass correlation coefficient, LoA = limits of agreement, MAPE = mean absolute percentage error, *MPE* Mean percentage error, *[CI]* 95% confidence intervals, *(oe)* Over estimation, *(ue)* Under estimation, *MVPA* Moderate to vigorous physical activity, *MWT* Minutes walking test, *Sed* Sedentary, *STS* Sit-to-stand test, *SWT* Steps walk test

**Table 3:** Overview of consumer grade devices evaluated on their measurement properties in the 67 studies

								Result	
Type	PA outcome	Population	Study	Measurement property	Criterion	Placement	Algorithm	Test	Outcome
**Fitbit**									
Alta	Steps	Cancer	Rossi [98]	Con V	SR	N.R	N.R	CCC	CCC = 0.00005 [CI -0.22—0.22]
		COPD	Blondeel [67]	CV	Acc	Wrist	Prop	LoA	306 [-2068; 2680] (oe)
		MS	Lavelle [80]	CV	DO	Wrist	N.R	LoA	-302.8 [-1036.8; 431.1] (oe)
		Stroke	Holubova [65]	CV	DO	Upper limb (b)	Prop	MARD	3.05—85.67%
						Lower limb (b)	Prop	MARD	1.33—11.08%
						Waist	Prop	MARD	0.47—3.66%
	Activity time	MS	Lavelle [80]	CV	Acc	Wrist	N.R	% error	100% [range -38.7—100]
Charge	Steps	Amputation	Smith [101]	CV	DO	Wrist	N.R	ICC	ICC = 0.86
		Multi	Treacy [23]	CV	DO	Wrist	N.R	ICC	ICC = 0.399 [CI -0.026- 0.654]
		PDs	Lamont [137]	CV	Acc	Wrist	N.R	ICC	ICC: 0.18 – 0.94
Charge 2	EE	CAD	Herkert [122]	CV	IC	Wrist	Prop	ICC	ICC = 0.10
		Heart failure	Herkert [122]	CV	IC	Wrist	Prop	ICC	ICC = 0.42
	Steps	Heart failure	Vetrovsky [141]	CV	Acc	Wrist	N.R	CCC	CCC = 0.48 [CI 0.20—0.69]
		Osteoarthritis	Collins [94]	CV	Acc	Wrist	Prop	ICC	ICC = 0.602
		PD	Lai [97]	CV	DO	Wrist	N.R	ICC	ICC: 0.27 – 0.47
		Progressive muscle diseases	Roberts-Lewis et al. [118]	CV	DO	Wrist	N.R	Spearman's rho	rho = 0.97 [CI 0.96—0.98]
	Intensity time	Osteoarthritis	Collins [94]	CV	Acc	Wrist	Cust	% bias	-5 – 37%
Flex	Steps	Amputation	Smith [101]	CV	DO	Wrist	N.R	ICC	ICC = 0.843
		CAD	Alharbi [140]	CV	Acc	N.R	Prop	Pearson's r	*r* = 0.947
		Post heart operation	Daligadu [109]	CV	DO	Wrist	N.R	CCC	CCC = 0.43
		MS	Balto [83]	CV	DO	Wrist	N.R	MPE	12.4 – 13.8%
			Block [85]	CV	DO + ACC	Wrist	N.R	ICC	2MWT DO: ICC = 0.69
									2MWT ACC: ICC = 0.59
			Block [85]	CV	ACC	Wrist	N.R	ICC	ICC = 0.74
			Block [84]	CV	DO	Wrist	N.R	ICC	ICC = 0.69 [CI 0.53—0.80]
			Block [84]	CV	ACC	Wrist	N.R	ICC	ICC = 0.98 [CI 0.97—0.98]
		Multi	Ummels [75]	CV	DO	Wrist	N.R	Pearson's r	*r* = 0.31
	Intensity time	Chronic knee symptoms	Semanik [99]	CV	Acc	Wrist	Prop	Spearman's rho	rho: 0.25 – 0.73
		CAD	Alharbi [140]	CV	Acc	N.R	Prop	Pearson's r	r: 0.04 – 0.72
		Stroke	Hei Chow [60]	CV	Acc	Wrist	Prop	ICC	ICC: -0.236—0.884
	Distance walked	Post heart operation	Daligadu [109]	CV	DO	Wrist	N.R	CCC	CCC = 0.37
Flex 2	Steps	MS	Block [84]	CV	Acc	Wrist	N.R	ICC	ICC = 0.98 [CI 0.97—0.99]
		Osteoarthritis	Yu [136]	CV	SR	Wrist	N.R	Correlation	0.20—0.28
				Resp	SR & tests	Wrist	N.R	Correlation	-0.28—0.28
Inc	Steps	PD	de Carvalho Lana [133]	CV	DO	Waist	N.R	Pearson's r	*r* = 0.82
Inspire HR	Steps	MS	Polhemus [81]	CV	DO	Wrist	N.R	CCC	CCC = 0.66 (CI 0.14—0.80)
				CV	Acc	Wrist	N.R	CCC	CCC: 0.33—0.65
		Progressive muscle diseases	Roberts-Lewis [118]	CV	Acc	Wrist	N.R	Spearman's rho	rho = 0.76 (CI 0.60—0.87)
				TRT R	Acc	Wrist	N.R	ICC	ICC = 0.96 (CI 0.92—0.98)
				Resp	Acc	Wrist	N.R	AUC	AUC = 0.86 (CI 0.75—0.97)
	Activity time	MS	Polhemus [81]	CV	Acc	Wrist	N.R	CCC	CCC: 0.18—0.52
	Intensity time	MS	Polhemus [81]	CV	Acc	Wrist	N.R	CCC	CCC: 0.41—0.80
		Progressive muscle diseases	Roberts-Lewis et al. [118]	CV	Acc	Wrist	N.R	Spearman's rho	rho = 0.51 (CI 0.29—0.69)
				TRT R		Wrist	N.R	ICC	ICC = 0.78 (CI 0.63—0.87)
				Resp	Acc	Wrist	N.R	AUC	AUC = 0.72 (CI 0.56—0.88)
	MET	Progressive muscle diseases	Roberts-Lewis et al. [118]	CV	Acc	Wrist	N.R	Spearman's rho	rho = 0.63 (CI 0.47—0.74)
				TRT R		Wrist	N.R	ICC	ICC = 0.94 (CI 0.89—0.97)
				Resp	Acc	Wrist	N.R	AUC	AUC = 0.90 (CI 0.81—0.98)
One	Steps	Amputation	Arch [92]	CV	DO	Ankle (a)	N.R	ICC	ICC: 0.88 – 0.97
		Cancer	Van Blarigan [102]	CV	Acc	Waist	N.R	Pearson's r	*r* = 0.94
					Acc	Waist	N.R	Pearson's r	*r* = 0.67
		MS	Balto [83]	CV	DO	Waist	N.R	MPE	1.9%—1.9%
		Multi	Unmeis (2018)	CV	DO	Waist	N.R	Pearson's r	*r* = -0.15
			Treacy [23]	CV	DO	Ankle	N.R	ICC	ICC = 0.919 [CI 0.772—0.961]
						Waist	N.R	ICC	ICC = 0.397 [CI -0.087—0.689]
		Myositis	Saygin [106]	CV	Acc	Waist	Prop	ICC	ICC = 0.96 (CI 0.92—0.98)
				TRT R		Waist	Prop	ICC	ICC = 0.89 (CI 0.72—0.96)
				Resp	SR	Waist	Prop	Spearman's rho	rho = 0.63
		PDs	Lai [97]	CV	DO	Waist	N.R	ICC	ICC: 0.98 – 0.98
		Stroke	Duclos [47]	CV	DO	Ankle	Prop	% error	0.50 – 2.67%
			Henderson [64]	CV	DO	Ankle (a)	Prop	ICC	ICC: 0.71—0.92
						Ankle (ua)	Prop	ICC	ICC: 0.78—0.92
			Hui [50]	CV	Acc	Ankle (ua)	Prop	Regression r	r: 0.97 – 0.99
			Klassen [52]	CV	DO	Ankle (ua)	Prop	MPE	4.0 – 15.8%
						Waist	Prop	MPE	7.7 – 84.6%
			Klassen [53]	CV	Acc	Ankle (ua)	Prop	LoA	156.1 [-239.6; 551.9] (u)
	Intensity time	Cancer	Van Blarigan [102]	CV	Acc	Waist	Prop	Pearson's r	r: 0.65 – 0.85
		Myositis	Saygin [106]	CV	Acc	Waist	Prop	ICC	ICC: 0.59—0.96
		Stroke	Hui [50]	CV	Acc	Ankle (ua)	Prop	Regression r	r: 0.41 – 0.97
Surge	Steps	PD	Wendel [103]	CV	DO	Wrist (la)	Prop	ICC	ICC: -0.003 – 0.41
Ultra	Steps	Stroke	Costa [46]	CV	DO	Wrist (b)	N.R	Pearson's r	*r* = 0.67
Zip	Steps	COPD	Blondeel [67]	CV	Acc	Waist	Prop	LoA	-1055 [-2820; 589] (ue)
			Prieto-Centurion [74]	CV	DO	Waist	N.R	LoA	6 [-14; 25] (ue)
		Cardiac diseases	Thorup [127]	CV	Acc	Waist	Prop	ICC	ICC: 0.60 – 0.96
		MS	Lavelle [80]	CV	DO	Waist	N.R	LoA	-6.2 [-717.4; 705.0] (oe)
		Multi	Farmer [90]	CV	DO	Foot	Prop	ICC	ICC: 0.60—0.85
		PD	Wendel [103]	CV	DO	Waist	Prop	ICC	ICC: -0.03 – 0.98
		Polymyalgia rheumatica	Chandrasekar [112]	CV	DO	Waist	N.R	LoA	1 [-8;10] – 10 [-55; 74]
						Shirt, midline	N.R	LoA	-6 [-81; 68] – 12 [-58; 83]
		Stroke	Clay [41]			Waist	N.R	Kendall Tau-b	τ = 0.80
			Schaffer [58]	CV	DO	Waist	N.R	MAPE	-88.2 – 4.2%
				TRT R		Waist	N.R	ICC	ICC = 0.974
**Garmin**									
Forerunner 35	Steps	Stroke	Huber [66]	CV	Acc	Wrist (ua)	N.R	LoA	-1.6 [-86.9; 83.5]—5.0 [-63.7; 2689.5]
				TRT R		Wrist (ua)	N.R	ICC	ICC: 0.989—0.996
Vivofit	Steps	Amputation	Smith [101]	CV	DO	Wrist (b)	N.R	ICC	ICC = 0.86
		Heart failure	Vetrovsky [141]	CV	Acc	Wrist	N.R	CCC	CCC = 0.89 [CI 0.75; 0.96]
		Multi	Treacy [23]	CV	DO	Wrist	N.R	ICC	ICC = 0.259 [CI -0.071; 0.556]
		PD	Lamont [137]	CV	Acc	Wrist	N.R	ICC	ICC: 0.36 – 0.97
		Stroke	Schaffer [58]	CV		Wrist (ua)	N.R	MAPE	-90.1 – -16.0%
						Wrist (a)	N.R	MAPE	-68.2 – -4.0%
				TRT R		Wrist (ua)	N.R	ICC	ICC = 0.964 [CI 0.916; 0.984]
						Wrist (a)	N.R	ICC	ICC = 0.858 [CI 0.672; 0.939]
Vivofit 3	Steps	Amputation	Smith & Guerra [107]	CV	DO	Ankle	N.R	ICC	ICC = 0.122 (CI -0.141—0.398)
						Wrist	N.R	ICC	ICC = 0.895 (CI 0.802—0.945)
		Heart failure	Vetrovsky [141]	CV	Acc	Wrist	N.R	CCC	CCC = 0.92 [CI 0.78; 0.97]
Vivifit 4	Steps	MS	Lavelle [80]	CV	DO	Wrist	N.R	LoA	-251.05 [-717.4; 253.6] (oe)
	Activity time	MS	Lavelle [80]	CV	DO	Wrist	N.R	% error	100% [range 100—100]
Vivosmart 3	Steps	PD	Lai [97]	CV	DO	Wrist (la)	N.R	ICC	ICC: 0.67 – 0.97
Vivosmart 4	Steps	PD	Bianchini [130]	CV	DO	Wrist (b)	Prop	ICC	ICC = 0.66 (CI 0.31—0.83)
**Omron**									
Active Style Pro HJA-350	MET	Stroke	Shimizu [59]	CV	MET com	Waist	Prop	T-test (1-sample)	*P* < .05
Active Style Pro HJA-750c	EE	DM	Nishida [134]	CV	DLW	Waist	TEE = BMR (Ganpule's equation) * PAL	Pearson's r	TEE: *r* = 0.87
	PAL	DM	Nishida [134]	CV	DLW	Waist	PAL = ([BMR (Ganpule's equation) + AEE (prop)]*10/9)*BMR	Pearson's r	*r* = 0.71
	Intensity time	COPD	Miyamoto [73]		Acc	Waist	Cust	Pearson's r	r: 0.38 – 0.81
					Acc	Waist	Cust	Pearson's r	r: -0.05 – 0.83
HJ-113	Steps	Amputation	Smith [101]	CV	DO	Waist	N.R	ICC	ICC = 0.928
HJ-322U-E	Steps	Heart failure	Vetrovsky [141]	CV	Acc	Waist	N.R	CCC	CCC = 0.82 [CI 0.56; 0.93]
HJ-720ITC	Steps	COPD	Danilack [69]	CV	DO	Waist	N.R	LoA	34 [-186; 253]
Walking Style x	Steps	Multi	Ummels [75]	CV	DO	Waist	N.R	Pearson's r	*r* = 0.25
**Yamax**									
Digiwalker CW-700	Steps	Bronchiectasis	O'Neill [117]	CV	Acc	Waist	N.R	LoA	-167 [-3078; 2745] (oe)
		Multi	Ummels [75]	CV	DO	Wrist	N.R	Pearson's r	*r* = -0.33
	Activity time	Bronchiectasis	O'Neill [117]	CV	Acc	Waist	N.R	LoA	Daily activity time: 165 [62; 269] min
Digiwalker SW-200	Steps	MS	Anens [79]	CV	DO	N.R	N.R	Spearman's rho	rho: 0.64—0.97
			Balto [83]	CV	DO	Waist	N.R	MPE	8.5 – 9.7%
			Lavelle [80]	CV	DO	Waist	N.R	LoA	119.4 [-498.0; 736.8] (ue)
	EE	COPD	Farooqi [71]	CV	DLW	Waist	Harris-Benedict	ICC	ICC = 0.70 [CI 0.23; 0.89]
							Schofield	ICC	ICC = 0.71 [CI 0.21; 0.89]
							WHO	ICC	ICC = 0.74 [CI 0.33; 0.90]
							Moore	ICC	ICC = 0.69 [CI 0.21; 0.88]
							Nordic Nutrtion Recommendation	ICC	ICC = 0.70 [CI 0.17; 0.89]
							Nordenson	ICC	ICC = 0.40 [CI -0.16; 0.77]
	PAL	COPD	Farooqi [71]	CV	DLW + IC	Waist	Cust	ICC	ICC = 0.34
**Google**									
Fit	Steps	PD	de Carvalho Lana [133]	CV	DO	Waist	N.R	Pearson's r	*r* = 0.92
		Stroke	Costa [46]	CV	DO	Waist	N.R	Pearson's r	*r* = 0.66
				TRT R		Waist	N.R	ICC	ICC = 0.76
			Polese [57]	CV	DO	Front pocket (a)	N.R	ICC	ICC = 0.93 [CI 0.86; 0.96]
	EE	Stroke	Faria [49]	CV	IC	Front pocket (a)	N.R	Pearson's r	*r* = 0.30
Android stepcounter	Steps	RA	Wagner [129]	CV	DO	Waist	Prop	MAPE	1.0—19.3%
**Apple**									
Watch Sport	EE	Multi	Falter [88]	CV	IC	Wrist	Prop	ICC	ICC = 0.797
Health	Steps	MS	Balto [83]	CV	DO	Front pocket	N.R	MPE	2.7 – 2.9%
Iphone CMPedometer	Steps	PAD	Ata [93]	CV	DO	Hand/front pocket	N.R	% error	-7.2 ± 13.8%
Iphone SE	Steps	Cancer	Douma [121]	CV	Acc	Waist	N.R	ICC	ICC = 0.97 [CI 0.95; 0.98]
	Distance walked	Cancer	Douma [121]	CV	Acc	Waist	N.R	ICC	ICC = 0.47 [CI 0.21; 0.67]
**Geonaute**									
Onstep 400	EE	Stroke	Compagnat [43]	CV	IC	Waist	Prop	Pearson's r	TEE: *r* = 0.66
							Cust	Pearson's r	TEE: *r* = 0.87
			Mandigout [55]	CV	IC	Neck	N.R	Spearman's rho	rho = -0.16
						Waist	N.R	Spearman's rho	rho = -0.07
	Distance walked	Stroke	Compagnat [44]	CV	DO	Neck	Prop	Pearson's r	*r* = 0.91
						Waist	Prop	Pearson's r	*r* = 0.98
**JawBone**									
Up2	Steps	MS	Balto [83]	CV	DO	Wrist	N.R	MPE	1.9 – 3.9%
		PD	Wendel [103]	CV	DO	Wrist	Prop	ICC	ICC: -0.02 – 0.17
Up24	Steps	Multi	Ummels [75]	CV	DO	Wrist	N.R	Pearson's r	*r* = 0.09
Up Move	Steps	MS	Balto [83]	CV	DO	Waist	N.R	MPE	8.4 – 8.9%
		PD	Wendel [103]	CV	DO	Waist	Prop	ICC	ICC: -0.03 – 0.85
**Polar**									
A300	Steps	COPD	Boeselt [68]	CV	Acc	Wrist	Prop	ICC	ICC = 0.986
	Activity time	COPD	Boeselt [68]	CV	Acc	Wrist	Prop	ICC	Daily activity: ICC = 0.335
	MET	COPD	Boeselt [68]	CV	Acc	Wrist	Prop	ICC	ICC = 0.066
	Calories	COPD	Boeselt [68]	CV	Acc	Wrist	Prop	ICC	ICC = 0.829
Loop	Steps	Amputation	Smith [101]	CV	DO	Wrist	N.R	ICC	ICC = 0.723
T131	EE	Chronic lung disease	Dhillon [70]	CV	IC	N.R	Flex Heart Rate Method	LoA	-0.5 [-1.6; 0.7] – 0.4 [-0.3; 1.1]
**Samsung**									
Galaxy S4 mini	Mean vector magnitude	MS	Zhai [87]	CV	Acc	Habitual phone pos	N.R	Spearman's rho	rho: 0.06 – 0.33
	Variance vector magnitude	MS	Zhai [87]	CV	Acc	Habitual phone pos	N.R	Spearman's rho	rho: -0.13 – 0.29
Health	Steps	PD	de Carvalho Lana [133]	CV	DO	Waist	N.R	Pearson's r	*r* = 0.54
		Stroke	Costa [46]	CV	DO	Waist	N.R	Pearson's r	r: 0.18 – 0.19
				TRT R		Waist	N.R	ICC	ICC: -0.70 – 0.10
**Lumo**									
Lumoback	Steps	Multi	Ummels [75]	CV	DO	Lower back	N.R	Pearson's r	*r *= 0.19
	Intensity time	Lower back pain	Takasaki [135]	TRT R		Lower back	Prop	ICC	Sed.: ICC = 0.75 [CI 0.26; 0.91]
**Pacer Health**									
Pacer Pedometer	Steps	PD	de Carvalho Lana [133]	CV	DO	Waist	N.R	Pearson's r	*r* = 0.77
		Stroke	Costa [46]	CV	DO	Waist	N.R	Pearson's r	r: 0.68 – 0.80
				TRT R		Waist	N.R	ICC	r: 0.68 – 0.80
**Withings**									
Go	Steps	Heart failure	Vetrovsky [141]	CV	Acc	Wrist	N.R	CCC	CCC = 0.90 [CI 0.77–0.96]
Health Mate	Steps	MS	Balto [83]	CV	DO	Front pocket	N.R	MPE	1.5 – 3.5%
**Alexander et al**									
mSteps	Distance walked	MS	Alexander [78]	CV	DO	Arm	N.R	LoA	0.262 [-1.496; 2.020] m (oe)
**Corussen LLC**									
Accupedo	Steps	Multi	Ummels [75]	CV	DO	Waist	N.R	Pearson's r	*r* = 0.32
**DHS group**									
MOVEBAND	Steps	Amputation	Smith [101]	CV	DO	Wrist (b)	N.R	ICC	ICC = 0.897
**Juen**									
MoveSense	Distance walked	Pulmonary disease	Juen [72]	CV	DO	Lower back	Cust	LoA	-7.7 [CI -33.0; 17.6] meter (oe)
**Leap Fitness Group**									
Pedometro	Steps	Chronic pain	Ferreira [132]	CV	DO	Arm & waist	N.R	Pearson's r	For all tasks and placements: p ≥ 0.99
**Letscom**									
Letscom smartwatch	Steps	MS	Lavelle [80]	CV	DO	Wrist	N.R	LoA	-390.0 [-1006.7; 226.7] (oe)
	Activity time	MS	Lavelle [80]	CV	Acc	Wrist	N.R	% error	52.9% [range 5.6—65.1]
**Mario Herzberg**									
EasyFit pedometer	Steps	Chronic pain	Ferreira [132]	CV	DO	Arm & waist	N.R	Pearson's r	For all tasks and placements: p between -0.32 and 0.24
**Mio**									
Slice	EE	CAD	Herkert [122]	CV	IC	Wrist	Prop	ICC	ICC = 0.12
		Heart failure	Herkert [122]	CV	IC	Wrist	Prop	ICC	ICC = 0.11
**Nakosite**									
3D walking	Steps	Stroke	Negrini [56]	CV	DO	Ankle (a)	Prop	ICC	ICC: -0.20 – 0.70
						Ankle (ua)	Prop	ICC	ICC: -0.28 – 0.69
						Waist	Prop	ICC	ICC: -0.42 – 0.57
						Wrist (a)	Prop	ICC	ICC: -0.50 – 0.45
						Wrist (ua)	Prop	ICC	ICC: -0.41 – 0.45
**Pedometer Australia**									
G-Sensor 2026	Steps	Multi	Treacy [23]	CV	DO	Waist	N.R	ICC	ICC = 0.308 [CI -0.094; 0.604]
**ProtoGeo Oy**									
Moves	Steps	MS	Balto [83]	CV	DO	Front pocket	N.R	MPE	12.5 – 14.2%
**Technogym**									
MyWellnes Key	Intensity time	DM	McGinley [110]	CV	SR	Waist	Prop	Spearman's rho	rho = 0.81 [CI 0.76; 0.85]

Ordering on number of studies evaluating manufacturer. This is a condensed version of the more detailed table in Supplementary file 6

*EE* Energy expenditure, *MET* Metabolic equivalent, *PAL* Physical activity level, *CAD* Coronay artery disease, *COPD* Chronic obstructive pulmonary disease, *DM* Diabetes mellitus, *iSCI* incomplete spinal cord injury, *MS* Multiple sclerosis, *PAD* Pulmonary artery disease, *PD* Parkinson's disease, *RA* Rheumatoid arthritis, *SCI* Spinal cord injury, *CV* Criterion validity, *Con V* Construct validity, *Resp* Responsiveness, *TRT R* Test–retest reliability, *Acc* Accelerometer, *DLW* Doubly labelled water, *DO* Direct observation, *IC* Indirect calorimetry, *SR* Self-report, *(a)* Affected side, *(b)* Both affected and unaffected side, *(la)* Less affected side, *(LRL)* Longest residual limb, *(SRL)* Shortest residual limb, *(ua)* Unaffected side, *Cust* Custom algorithm, *LFE* Low frequency effect, *N.R.* Not reported, *Prop* Proprietary algorithm, *TEE* Total energy expenditure, *APE* Absolute percentage error, *CCC* Concordance correlation coefficients, *ICC* Intraclass correlation coefficient, *LoA* Limits of agreement, *MAPE* Mean absolute percentage error, *MARD* Mean absolute relative difference, *MPE* Mean percentage error, *[CI]* 95% confidence intervals, *(oe)* Over estimation, *(ue)* Under estimation, *MVPA* Moderate to vigorous physical activity, *MWT* Minutes walking test, *Sed* Sedentary, *STS* Sit-to-stand test, *SWT* Steps walk test

### Research-grade devices

#### ActiGraph

Measurement properties of a type of ActiGraph were determined in 28 studies, with 24 studies evaluating type GT3 [23, 40, 43–45, 49, 51, 55, 61, 64, 81, 84, 89, 94–96, 104, 105, 108, 112, 114–116, 139] and four studies evaluating type GT9 [76, 79, 93, 107] (Table 2). Only validity was measured in these 28 studies, with 27 determining criterion validity, and 1 construct validity [105]. For the GT3, the criterion validity of energy expenditure, steps, time spent in intensity zones, time in activities, distance walked, metabolic equivalent (MET) and activity counts and construct validity for steps and vector magnitude was measured in 12 unique diagnosis groups and one mixed group with variable diagnoses. Four studies applied custom-created algorithms [61, 114, 115, 139], two studies applied both a custom and a proprietary algorithm [43, 61], two studies did not report on used algorithms [45, 55] and the other studies used proprietary algorithms (*n* = 21), with Freedson [142] the most commonly reported. The GT3 was placed at five different body regions (ankle, upper arm, thigh, waist and wrist), at both the affected and unaffected side (for diagnosis groups that may suffer from unilateral impairment, e.g. stroke, unilateral amputation). The GT9 was studied on criterion validity of steps and sedentary time in 5 different diagnosis groups, placed on the ankle, waist or wrist. Three studies used one or more proprietary algorithms [76, 79, 93], and one study did not report on the used algorithm [107].The used epoch length of the instruments ranged from 0.033 s to 60 s, or it was not reported. Sampling rate was set at 10 Hz (1 study [45]), 30 Hz (14 studies (40, 44, 49, 51, 64, 76, 81, 84, 113, 115, 116, 117{Compagnat, 2022 #154, 140)}, 50 Hz (1 study [107]), 90 Hz (1 study [79], 100 Hz (2 studies [93, 104]), or it was not reported (9 studies [23, 43, 55, 89, 94–96, 105, 108]). The criterion validity was measured with 13 different statistical tests (among others: Pearson’s r, Spearman’s rho, intraclass correlation coefficient (ICC), Bland–Altman level of agreement, % accuracy). The results had a wide range of variation, with correlations between 0.004 to 0.97 and accuracy between 43.0% to 81.4%. This large variability was found among different PA outcomes, but also within PA outcomes.

#### PAL technologies

The devices of PAL technologies were evaluated in eight studies, six studies evaluating the ActivPAL [23, 54, 91, 95, 131, 138] and two studies evaluating the ActivPAL3 [86, 116] (Table 2). Criterion validity for steps, time spent in different activities or MET were measured in seven studies [23, 54, 86, 95, 116, 131, 138] in five unique diagnosis groups and one mixed group with variable diagnoses. Test–retest reliability was measured for steps, time spent in different activities and MET in two studies [54, 91] in two unique diagnosis groups. One study did not report the used algorithm [138], the other seven used proprietary algorithms. All studies placed the device on the thigh. The used epoch lengths were 0.1 s [91], 1 s [95] and 15 s [54, 131, 138]. Three studies did not report the epoch length [23, 86, 116]. Sampling rate was set at 10 [54, 91] or 20 Hz [86], or was not reported [23, 95, 116, 131, 138]. Test–retest reliability was measured as ICC, ranging from 0.654 to 0.997 and as absolute percentage error, ranging from 3.3% to 6.5%, depending on the PA outcome, diagnosis group and task. Criterion validity was measured as Pearson’s r, ICC, Bland–Altman level of agreement, percentage accuracy and percentage error, and varied with correlations between 0.65 and 0.99, accuracy between 90.7–100% and error between 0.3–3.1%, all depending on the PA outcome, diagnosis group and task.

### Consumer-grade devices

#### Fitbit

Eleven different types of Fitbits were evaluated: Alta (*n* = 4 studies) [65, 67, 80, 98], Charge (*n* = 3 studies) [23, 101, 137], Charge 2 (*n* = 5 studies) [94, 97, 118, 122, 141], Flex (*n* = 9 studies) [60, 75, 83–85, 99, 101, 109, 140], Flex 2 (*n* = 2 studies) [84, 136], Inc (*n* = 1 study) [133], One (*n* = 12 studies) [23, 47, 50, 52, 53, 64, 75, 83, 92, 97, 102, 106], Surge (*n* = 1 study) [103], Ultra (*n* = 1 study) [46] and Zip (*n* = 9 studies) [41, 58, 67, 74, 80, 90, 103, 112, 127] (Table 3). Criterion validity was measured for steps, energy expenditure, MET, time spent in different intensity zones, time spent in different activities and distance walked by 38 studies in 15 unique diagnosis groups, and three mixed groups with variable diagnoses. Convergence validity of the Alta was measured in one study for steps in cancer patients [98]. Test–retest reliability of the Inspire (*n* = 1 study) [118], One (*n* = 1 study) [106] and the Zip (*n* = 1 study) [58], for steps, MET and time spent in different intensity zones in patients with stroke, myositis or progressive muscle diseases. Responsiveness was measured for the Flex 2 (*n* = 1 study), Inspire (*n* = 1 study) and One (*n* = 1 study) for steps, MET and time spent in different intensity zones in patients with osteoarthritis, myositis or progressive muscle diseases. The Charge, Charge 2, Flex, Flex 2, Surge and Ultra were positioned at the wrist or it was not reported, the Alta at the lower limb, waist or wrist, the One at the ankle or waist, and the Zip at the foot, the waist or the midline of a shirt. Devices were placed at both the affected and unaffected side (for diagnosis groups that may suffer from unilateral impairment). One study used a custom algorithm [94], the other studies either used proprietary algorithms or did not report the used algorithm. Criterion validity of the Fitbits was measured with 13 different statistical tests, with correlations ranging from -0.236 to 0.99 and mean percentage errors ranging from 1.9 to 84.9%. Convergence validity, measured with concordance correlation coefficient, was smaller than 0.001 compared with a questionnaire. Test–retest reliability, measured with ICC, was 0.78—0.97. Responsiveness was measured with area under the curve (0.72 – 0.90) or correlation (-0.28 – 0.63).

#### Garmin

Six different types of Garmin devices were evaluated: Forerunner 35 (*n* = 1 study) [66], Vivofit (*n* = 5 studies) [23, 58, 101, 137, 141], Vivotfit 3 (*n* = 2 studies) [107, 141], Vivofit 4 (*n* = 1 study) [80], Vivosmart 3 (*n* = 1 study) [97] and Vivosmart 4 (*n* = 1 study) [130] (Table 3). Studies measured criterion validity for steps and time spent in different activities in five unique diagnosis groups and one mixed group with variable diagnoses. Test–retest reliability of the Forerunner 35 and Vivofit was measured for steps in a stroke population. All devices were worn on the wrist, with the Vivofit 3 also worn on the ankle in one study [107]. One study used the proprietary algorithm [130], the other studies did not report on the used algorithm. Sampling rate and epoch length were not reported for the devices. Criterion validity was measured using 5 different statistical tests (ICC, concordance correlation coefficient, Bland–Altman level of agreement, percentage error and mean absolute percentage error). Correlations ranged from 0.12 to 0.97, depending on the device, PA outcome and task. Test–retest reliability was measured using ICC, ranging from 0.86 to 0.99.

## Discussion

This scoping review provides a critical mapping of the research on measurement properties (validity, reliability and responsiveness) of device-based instruments assessing PA in ambulatory adults with disabilities and/or chronic diseases. The results show a large variability in research on the measurement properties of device-based instruments assessing PA in adults with physical disabilities and/or chronic diseases. Predominantly, different forms of validity are assessed in a total of 78 different research- and consumer-grade devices using 14 different PA outcomes in 23 different diagnosis groups. There is large variability in measurement properties within and between instruments and studies. The ActiGraph devices are the most frequently studied research-grade devices, and the Fitbit devices are the most frequently studied consumer-grade devices.

### PA outcomes

PA behavior is assessed with a variety of different PA outcomes. The most commonly used PA outcome is step count, comparable to previous reviews on the use of device-based PA instruments [143–145]. However, step count informs only about walking and walking-related tasks and does not give information on the intensity and duration of PA behavior from a broader perspective. Even when step count is not used as the PA outcome, we have found that studies mostly use walking-related tasks to study the measurement properties. This results in device-based PA instruments only applicable for valid and reliable measurement of walking, and thereby excluding valid and reliable measurement of other modes of PA behavior such as cycling and swimming.

The importance of frequency, intensity and duration of PA is stressed by the guidelines for PA, which typically include statements on the frequency and duration in certain intensities needed for achieving optimal health benefits [146, 147]. Energy expenditure and intensity time are PA outcomes that take two of these dimensions into account (i.e. intensity and duration). However, the trend visible in this scoping review is that incorporating intensity in the PA outcome results in lower validity outcomes. As intensity depends on the used cut-off points and algorithms [148], given the fact that these are mostly developed for a general population [9], this finding is not surprising. Custom-made disease-specific algorithms could be a solution to increase validity outcomes. In the eight studies using custom algorithms in five different instruments, generally moderate to good values of validity are found [43, 61, 73, 94, 114, 115, 125, 134]. However, just two of these studies compare a custom disease-specific algorithm with a proprietary algorithm, reporting increased validity for the custom algorithm [43, 61]. More research needs to compare custom disease-specific algorithms with proprietary algorithms.

When using intensity time and energy expenditure as PA outcomes only, information on how and where PA is being performed is not acquired. This information can be of importance for rehabilitation specialists and policymakers to identify possibilities to improve PA behavior in people with physical disabilities and/or chronic diseases. The how (or mode) of PA can be measured using activity time. This outcome is used by 15 studies, with a variety of outcomes on measurement properties [48, 54, 68, 80–82, 86, 91, 95, 100, 116, 117, 120, 131, 138]. As device-based PA instruments only capture movement or acceleration of the body, the where (or context) of PA cannot be measured with these instruments [15]. Self-report instruments can fill this gap, hence the consensus that both self-report and device-based PA instruments should be used in complement to each other [12, 14]. In conclusion, we can say that different PA outcomes have different advantages and disadvantages, but none of the device-based PA outcomes is able to capture the complete construct of PA (i.e. setting, mode, intensity, duration, frequency). This requires future research consideration.

### Population

Most of the studies on measurement properties of device-based PA instruments are conducted in diagnosis-specific populations, and only six studies concerned a mixed population including people with different physical disabilities and/or chronic diseases [23, 75, 77, 88–90]. People with different diagnoses may suffer from different walking-related complications [19–22], which could have an effect on measurement properties of device-based PA instruments (e.g. frequency spectrum of accelerations, energetic cost and efficiency of movement/activities). Thus, a diagnosis-specific approach in these studies seems logical. However, this diagnosis-specific focus does have the drawback that it lacks generalizability to other types of physical disabilities and/or chronic diseases. It might be of interest to conduct studies using a functioning-specific focus, in line with the ICF model [35]. Functional limitations may differ between individuals within diagnosis groups, and different diagnoses might share problems with functioning, such as slower and asymmetrical gait [16–18], which can influence the measurement properties of PA devices [24]. Studies using this functioning-specific approach can give insight in PA devices with good measurement properties for multiple physical disabilities and/or chronic diseases. This is of relevance as monitoring and measuring PA is important for all physical disabilities and/or chronic diseases. As self-monitoring is an important behavior change technique [8], a PA device that is valid and reliable for a variety of people with physical disabilities and/or chronic diseases might increase feasibility of PA promoting interventions for people with physical disabilities and/or chronic diseases. The same can be suggested for the rehabilitation setting, in which a variety of patient groups are treated. Correct measurement and monitoring of PA in the rehabilitation setting can lead to a more tailored approach to improve PA behavior, which ultimately may improve health and functioning [149].

### Measurement properties and statistics

The criterion validity of the device-based PA instruments is the most common studied measurement property. Besides criterion validity, only 11 studies on (test–retest) reliability [46, 54, 58, 66, 91, 105, 106, 111, 113, 118, 135] and six studies on responsiveness are included [82, 100, 105, 106, 118, 136]. Good reliability of a device-based PA instrument is needed for suitable clinical application to ensure that a change in PA behavior over time is related to an actual change instead of measurement error. Good responsiveness is needed as a prerequisite for measuring effectiveness of PA promotion in clinical care. During our search, we found studies that investigated the number of days needed for reliable measurement of PA using devices in free-living settings [150–153]. Although this is important information, it is not considered a measurement property since it does not provide information on the measurement error and the extent to which repeated measurement outcomes are the same for people who have not changed [37].

There is a large variety of statistical methods used to study the measurement properties of the different devices, which makes it difficult to compare the different studies. Most studies included in this review assessed criterion validity and test–retest reliability, for which methods of correlational nature are recommended [154]. The use of techniques comparing means (e.g. t-test and analysis of variance) is irrelevant in studies on measurement properties, since these pretend to measure a difference (from a criterion measure or between two measurements), instead of an agreement [37]. Still, a number of included studies did not use the appropriate statistical methods according to the international standards of the COSMIN group.

### Technical decisions

Using device-based PA instruments in research or clinical practice, numerous choices about data collection and data processing need to be made. All these choices could influence the measurement properties. First, one needs to think about the placement of the device on the body. Multiple studies showed the influence of placement of the device on measurement properties [23, 40, 44, 45, 51, 53, 55, 56, 58, 65, 89, 96, 107, 112, 114, 120, 128], with no clear advantage to a single location. Algorithms and cut-off points are developed with a certain placement in mind, and are not interchangeable between placements [149, 155], explaining at least part of the influence of placement on measurement properties. Secondly, epoch length and sampling rate should be considered when using PA measurement devices. Previous studies have shown that different epoch lengths result in differences in PA outcomes [15, 156]. However, none of the reviewed studies have looked at the influence of epoch length on measurement properties. Furthermore, in a large number of studies (*n* = 25 in research-grade devices, *n* = 59 in consumer-grade devices) the used epoch length is not reported. The same is found for sampling rate, which is also not always reported. Therefore, we cannot make recommendations on the optimal epoch length and sampling rate. However, for the use of device-based instruments in practice, one needs to critically assess considerations such as accuracy versus storage capacity. Thirdly, another important choice is the algorithm used to convert the measured accelerations of movement into interpretable PA outcomes. Applying different general algorithms could lead to differences in measurement properties, which is shown by the three studies that compared multiple algorithms [49, 71, 76]. And as mentioned previously, custom-made disease-specific algorithms could influence the measurement properties when using intensity-based PA outcomes [43]. For research and clinical use, we suggest applying an algorithm that is evaluated for the specific population and activity level. However, based on our findings we cannot recommend certain algorithms, as this is beyond the scope of this review. Considering the effect of these technical choices on PA outcomes and the measurement properties of the device-based instruments, Burchartz et al. already stated in their state of science paper on device-based PA instruments that all important technical decisions (such as placement on the body, the used epoch length, sampling rate and algorithm) should be reported in studies on measurement properties [15]. As it is apparent from this review that reporting the technical decisions is not common practice in studies on measurement properties, we wholeheartedly support this recommendation.

### Strengths and limitations

The main strength of this scoping review is the detailed and extensive mapping of studies using a broad range of methodological approaches and in a diverse group of ambulatory people with physical disabilities and/or chronic diseases. Furthermore, we used a systematic process in this scoping review, with the screening and selection process for the majority done in duplicate using information from four major databases. Another strength is the transparency and openness of the current scoping review. We provided additional information on the screening and analysis processes in the supplements and on Open Science Framework, which greatly improves the reproducibility of our scoping review. Lastly, we provided detailed information on decisions made in the included studies, which has not been reported in such detail in previous reviews on this topic. The Supplementary files add an extra layer of information for the interested reader, and provide extra emphasis on the large variability of the studies (e.g. the variety in what is considered a valid day/case among the studies).

However, some limitations of this scoping review should be acknowledged. One of the limitations is related to the search strategy. Although we carefully developed our search strategy, together with an information specialist, it is possible that we missed important search terms (e.g. specific wearables, specific disease groups), which could have resulted in missed relevant studies. Also, the inclusion of some search terms could have led to a relative overrepresentation of certain studies or devices used in the studies. As an example, ‘ActiGraph’ was included as a search term in our search strategy, which we found as the most used research-grade device in the literature. However, a previous review of device-based PA instruments in cardiovascular patients also found the ActiGraph as most frequently used instrument [145]. We did not apply the search filter for measurement properties developed by the COSMIN group [157], as this increased our search results exponentially.

Another limitation is our Dutch view on the rehabilitation setting. One of our inclusion criteria was that the physical disability or chronic disease of the participants must be a primary reason for rehabilitation. However, rehabilitation might not be organized the same across countries. This may have resulted in us excluding certain diagnosis groups that would be included by researchers of other countries, and vice versa, using the same in- and exclusion criteria.

In the current scoping review, we did not differentiate the overview of the measurement properties to the used setting (i.e. laboratory setting vs free-living setting) of the studies, which can be considered a limitation. The difference in setting might influence the measurement properties, and thus entail different concepts. We reported the used setting of the studies in the description table (Table 1) so that readers who are interested in these concepts can find this information in the current scoping review. However, future reviews could put more in-depth focus on the differences in setting and their effect on measurement properties.

A limitation inherent to research on device-based PA instruments is the rapidly changing field with regard to the technology. The technology of these devices develops rapidly, leading to newer models to hit the market before previous models have been properly studied. This is especially true for the consumer-grade instruments, which illustrates a commercialky-driven approach to the development of new technology, not necessarily leading to a quality-driven market. For research purposes, there is more need for valid and reliable instruments.

### Future directions

Considering the importance of PA in people with physical disabilities and/or chronic diseases, and the need to measure and quantify PA in this population as stated by different research agenda’s [9–11], instruments with good measurement properties are vital. Due to the large variability in measurement devices and the methods used to evaluate these, we were unfortunately unable to make concrete recommendations for specific devices and settings based on this review. However, this review provides an overview of detailed information per measurement device, which we use to provide directions for research on measurement properties of device-based instruments assessing PA in people with physical disabilities and/or chronic diseases.

- The focus of research on measurement properties of device-based PA instruments in people with physical disabilities and/or chronic diseases needs to be less on step count as a PA outcome, as it provides a very narrow view of PA behavior. Energy expenditure and intensity time seem important, but the validity of these outcomes needs to be improved. More research is needed on the measurement properties when using activity time since this can be important information for rehabilitation purposes. To better measure the multidimensionality of PA, the use of device-based PA instruments can be supplemented by the simultaneous application of self-report instruments.
- Studies on measurement properties of device-based instruments should inform readers of important technical decisions made for data collection and data processing. Especially the placement of the device on the body, the epoch length, sampling rate, and the used algorithm in full detail should be reported, as these are known to influence PA measurement. This information will help with data comparison between studies, but will also inform in detail in which situation a device-based instrument should or could be used.
- Future research should investigate the influence of disease-specific versus general algorithms on the measurement properties (in this case mainly validity) of device-based PA instruments. Intensity is an important aspect of PA, as evidenced by the focus of PA guidelines on moderate to vigorous PA [146, 147]. The use of custom disease-specific algorithms could improve the ability of device-based instruments to capture intensity.
- More research on the measurement properties of device-based PA instruments should be conducted in populations consisting of people with different physical disabilities and/or chronic diseases, for example by using a functioning-specific approach. It would be beneficial to have a single device-based PA instrument with good measurement properties available for different diagnosis groups. This will improve the ease of use in a rehabilitation setting where different diagnosis groups are treated.
- Raw data from device-based instruments should be used, instead of using PA outcomes processed by proprietary algorithms. In this way, the measurement properties of the device-based instruments when using raw data can be studied in a diverse population, and this raw data can subsequently be processed into PA outcomes using disease-specific or even individualized algorithms. Important to note, is that these algorithms should also be validated. The use of raw data has also been recommended by previous studies [15, 149].
- Reliability and responsiveness of device-based instruments should be studied more often. These measurement properties are especially important when device-based PA instruments are used to study changes in PA behavior over time. And although there has been an increase in studies on these measurement properties (especially responsiveness) in the last two to three years, they are still underrepresented in the literature of this scoping review.
- The methodologically correct statistical methods should be used while studying measurement properties of device-based instruments. This will help with comparing different studies and will result in better informed researchers and health professionals when selecting device-based instruments.

## Conclusion

There is a large variability in research on the measurement properties of device-based instruments assessing PA in ambulatory adults with physical disabilities and/or chronic diseases. This variability shows a need for more standardization of and consensus on research in this field. Based on this scoping review, the results could provide researchers and health professionals with some directions for selecting a device-based PA instrument that suits their need. Finally, to improve research and bridge knowledge gaps, we provide future directions for researchers interested in studying the measurement properties of device-based instruments assessing PA in ambulatory adults with physical disabilities and/or chronic diseases.

### Supplementary Information

**Additional file 1:** **Supplementary file 1.** Protocol deviations. A files containing details of the deviations we made to the protocol.

**Additional file 2:** **Supplementary file 2.** Full search strategy for each database. A file containing the search strategy used for each database.

**Additional file 3:** **Supplementary file 3.** PICO, Selection criteria and Checklists. A file containing the PICO and the selection criteria used for the scoping review. Als contains the checklist that were used during the screening process.

**Additional file 4:** **Supplementary file 4.** Expanded description of the 103 studies included in the scoping review. A file containing the description of the included studies in more detail. Extra information on in- and exclusion criteria, the used task in the study and criteria for valid days and cases.

**Additional file 5:** **Supplementary file 5.** Expanded overview of research-grade devices evaluated on their measurement properties in 52 studies. An expanded overview of the research-grade devices evaluated on their measurement properties. Extra information on epoch length, sampling rate and results per condition.

**Additional file 6:** **Supplementary file 6.** Expanded overview of consumer-grade devices evaluated on their measurement properties in 74 studies. An expanded overview of the consumer-grade devices evaluated on their measurement properties. Extra information on epoch length, sampling rate and results per condition.

## Data Availability

Additional information can be found on Open Science Framework (https://osf.io/c27xv/). This includes information on the original protocol, the complete list of search results, an overview of the screening process (number of search results, duplications removed per deduplication step according to the method of Bramer et al., included and excluded per phase), the used checklists for the screening, the filled-in checklists by the screeners with results, and the data extracting tool (both the filled in version and a clean version).

## Abbreviations

PA: Physical activity
MET: Metabolic equivalent
ICC: Intraclass correlation coefficient

## References

[1] Caspersen CJ, Powell KE, Christenson GM (1985). Physical activity, exercise, and physical fitness: definitions and distinctions for health-related research. Public Health Rep.

[2] Mahar M, Rowe D (2002). Construct validity in physical activity research.

[3] Strath SJ, Kaminsky LA, Ainsworth BE, Ekelund U, Freedson PS, Gary RA (2013). Guide to the assessment of physical activity: Clinical and research applications: a scientific statement from the American Heart Association. Circulation.

[4] Haskell WL, Lee IM, Pate RR, Powell KE, Blair SN, Franklin BA (2007). Physical activity and public health: updated recommendation for adults from the American College of Sports Medicine and the American Heart Association. Med Sci Sports Exerc.

[5] Martin JJ (2013). Benefits and barriers to physical activity for individuals with disabilities: a social-relational model of disability perspective. Disabil Rehabil.

[6] Hollis ND, Zhang QC, Cyrus AC, Courtney-Long E, Watson K, Carroll DD (2020). Physical activity types among US adults with mobility disability, Behavioral Risk Factor Surveillance System, 2017. Disabil Health J.

[7] Martin Ginis KA, van der Ploeg HP, Foster C, Lai B, McBride CB, Ng K (2021). Participation of people living with disabilities in physical activity: a global perspective. Lancet.

[8] Michie S, Abraham C, Whittington C, McAteer J, Gupta S (2009). Effective techniques in healthy eating and physical activity interventions: a meta-regression. Health Psychol.

[9] Rosenberg DE, Bombardier CH, Hoffman JM, Belza B (2011). Physical activity among persons aging with mobility disabilities: shaping a research agenda. J Aging Res.

[10] van der Woude LHV, Houdijk HJP, Janssen TWJ, Seves B, Schelhaas R, Plaggenmarsch C (2021). Rehabilitation: mobility, exercise & sports; a critical position stand on current and future research perspectives. Disabil Rehabil.

[11] DiPietro L, Al-Ansari SS, Biddle SJH, Borodulin K, Bull FC, Buman MP (2020). Advancing the global physical activity agenda: recommendations for future research by the 2020 WHO physical activity and sedentary behavior guidelines development group. Int J Behav Nutr Phys Act.

[12] Nigg CR, Woll A. Best practices and future research directions: Consensus from the 2 International Workshop of the Center for the Assessment of Physical Activity (CAPA). Psychol Sport Exerc. 2020;50:101734.

[13] Troiano RP, McClain JJ, Brychta RJ, Chen KY (2014). Evolution of accelerometer methods for physical activity research. Br J Sports Med.

[14] Troiano RP, Stamatakis E, Bull FC (2020). How can global physical activity surveillance adapt to evolving physical activity guidelines? Needs, challenges and future directions. Br J Sports Med.

[15] Burchartz A, Anedda B, Auerswald T, Giurgiu M, Hill H, Ketelhut S, et al. Assessing physical behavior through accelerometry – State of the science, best practices and future directions. Psychol Sport Exerc. 2020;49:101703. 10.1016/j.psychsport.2020.101742 PMC7430559 32831643

[16] Morlino P, Balbi B, Guglielmetti S, Giardini M, Grasso M, Giordano C (2017). Gait abnormalities of COPD are not directly related to respiratory function. Gait Posture.

[17] Balaban B, Tok F (2014). Gait disturbances in patients with stroke. PM R.

[18] Moon Y, Sung J, An R, Hernandez ME, Sosnoff JJ (2016). Gait variability in people with neurological disorders: a systematic review and meta-analysis. Hum Mov Sci.

[19] van Schaik L, Geertzen JHB, Dijkstra PU, Dekker R (2019). Metabolic costs of activities of daily living in persons with a lower limb amputation: a systematic review and meta-analysis. PLoS One.

[20] Houdijk H, Blokland IJ, Nazier SA, Castenmiller SV, van den Heuvel I, Ijmker T (2021). Effects of handrail and cane support on energy cost of walking in people with different levels and causes of lower limb amputation. Arch Phys Med Rehabil.

[21] Buoite Stella A, Morelli ME, Giudici F, Sartori A, Manganotti P, di Prampero PE (2020). Comfortable walking speed and energy cost of locomotion in patients with multiple sclerosis. Eur J Appl Physiol.

[22] Blokland IJ, Ijmker T, Houdijk H. Aerobic Capacity and Aerobic Load of Activities of Daily Living After Stroke. Handbook of Human Motion. Cham: Springer International Publishing; 2018;2-3:863–84.

[23] Treacy D, Hassett L, Schurr K, Chagpar S, Paul SS, Sherrington C (2017). Validity of different activity monitors to count steps in an inpatient rehabilitation setting. Phys Ther.

[24] Svarre FR, Jensen MM, Nielsen J, Villumsen M (2020). The validity of activity trackers is affected by walking speed: the criterion validity of Garmin Vivosmart(®) HR and StepWatch(™) 3 for measuring steps at various walking speeds under controlled conditions. PeerJ.

[25] Cabot M, Daviet JC, Duclos N, Bernikier D, Salle JY, Compagnat M. First systematic review and meta-analysis of the validity and test retest reliability of physical activity monitors for estimating energy expenditure during walking in individuals with stroke. Archives of Phys Med Rehabil. 2022;103(11):2245–55. 10.1016/j.apmr.2022.03.020 35443210

[26] Gore S, Blackwood J, Guyette M, Alsalaheen B (2018). Validity and reliability of accelerometers in patients With COPD: a systematic review. J Cardiopulm Rehabil Prev.

[27] Rabinovich RA, Louvaris Z, Raste Y, Langer D, Van Remoortel H, Giavedoni S (2013). Validity of physical activity monitors during daily life in patients with COPD. Eur Respir J.

[28] Gebruers N, Vanroy C, Truijen S, Engelborghs S, De Deyn PP (2010). Monitoring of physical activity after stroke: a systematic review of accelerometry-based measures. Arch Phys Med Rehabil.

[29] Alharbi M, Bauman A, Neubeck L, Gallagher R (2017). Measuring overall physical activity for cardiac rehabilitation participants: a review of the literature. Heart Lung Circ.

[30] Lankhorst K, Oerbekke M, van den Berg-Emons R, Takken T, de Groot J (2020). Instruments measuring physical activity in individuals who use a wheelchair: a systematic review of measurement properties. Arch Phys Med Rehabil.

[31] Arksey H, O'Malley L (2005). Scoping studies: towards a methodological framework. Int J Soc Res Methodol.

[32] Levac D, Colquhoun H, O'Brien KK (2010). Scoping studies: advancing the methodology. Implement Sci.

[33] Tricco AC, Lillie E, Zarin W, O'Brien KK, Colquhoun H, Levac D (2018). PRISMA Extension for Scoping Reviews (PRISMA-ScR): checklist and explanation. Ann Intern Med.

[34] Liou TH, Pi-Sunyer FX, Laferrère B (2005). Physical disability and obesity. Nutr Rev.

[35] World Health Organization (2001). International classification of functioning, disability and health : ICF.

[36] National Center for Chronic Disease Prevention and Health Promotion (2022). About Chronic Diseases.

[37] Mokkink LB, Terwee CB, Patrick DL, Alonso J, Stratford PW, Knol DL (2010). The COSMIN study reached international consensus on taxonomy, terminology, and definitions of measurement properties for health-related patient-reported outcomes. J Clin Epidemiol.

[38] Bramer WM, Giustini D, de Jonge GB, Holland L, Bekhuis T (2016). De-duplication of database search results for systematic reviews in EndNote. J Med Libr Assoc.

[39] Cohen J (1968). Weighted kappa: nominal scale agreement with provision for scaled disagreement or partial credit. Psychol Bull.

[40] Campos C, DePaul VJ, Knorr S, Wong JS, Mansfield A, Patterson KK (2018). Validity of the ActiGraph activity monitor for individuals who walk slowly post-stroke. Top Stroke Rehabil.

[41] Clay LWM, Hargest C, Adhia DB. Gait quality and velocity influences activity tracker accuracy in individuals post-stroke. Top Stroke Rehabil. 2019;26(4):412–7. 10.1080/10749357.2019.1623474 31141461

[42] Compagnat M, Daviet JC, Batcho CS, David R, Salle JY, Mandigout S. Quantification of energy expenditure during daily living activities after stroke by multi-sensor. Brain Inj. 2019b;33(10):1341–6. 10.1080/02699052.2019.1641840 31309843

[43] Compagnat M, Mandigout S, Batcho CS, Vuillerme N, Salle JY, David R (2020). Validity of wearable actimeter computation of total energy expenditure during walking in post-stroke individuals. Ann Phys Rehabil Med.

[44] Compagnat M, Batcho CS, David R, Vuillerme N, Salle JY, Daviet JC, Mandigout S. Validity of the Walked Distance Estimated by Wearable Devices in Stroke Individuals. Sensors (Basel). 2019a;19(11):2497. 10.3390/s19112497. 10.3390/s19112497 PMC6604102 31159246

[45] Compagnat MMS, Chaparro D, Daviet JC, Salle JY (2018). Validity of the Actigraph GT3x and influence of the sensor positioning for the assessment of active energy expenditure during four activities of daily living in stroke subjects. Clin Rehabil.

[46] Costa PHV, de Jesus TPD, Winstein C, Torriani-Pasin C, Polese JC (2020). An investigation into the validity and reliability of mHealth devices for counting steps in chronic stroke survivors. Clin Rehabil.

[47] Duclos NC, Aguiar LT, Aissaoui R, Faria C, Nadeau S, Duclos C (2019). Activity monitor placed at the nonparetic ankle is accurate in measuring step counts during community walking in poststroke individuals: a validation study. Pm r.

[48] Fanchamps MHJ, Horemans HLD, Ribbers GM, Stam HJ, Bussmann JBJ. The Accuracy of the Detection of Body Postures and Movements Using a Physical Activity Monitor in People after a Stroke. Sensors. 2018;18(7):2167. 10.3390/s18072167. 10.3390/s18072167 PMC6069255 29976900

[49] Faria GS, Polese JC, Ribeiro-Samora GA, Scianni AA, Faria CD, Teixeira-Salmela LF. Validity of the accelerometer and smartphone application in estimating energy expenditure in individuals with chronic stroke. Braz J Phys Ther. 2018;23(3):236–43. 10.1016/j.bjpt.2018.08.003 PMC6531636 30143357

[50] Hui J, Heyden R, Bao T, Accettone N, McBay C, Richardson J, Tang A (2018). Validity of the fitbit one for measuring activity in community-dwelling stroke survivors. Physiother Can.

[51] Jayaraman C, Mummidisetty CK, Mannix-Slobig A, McGee Koch L, Jayaraman A (2018). Variables influencing wearable sensor outcome estimates in individuals with stroke and incomplete spinal cord injury: a pilot investigation validating two research grade sensors. J Neuroeng Rehabil.

[52] Klassen TD, Simpson LA, Lim SB, Louie DR, Parappilly B, Sakakibara BM, Zbogar D, Eng JJ (2016). "Stepping Up" Activity poststroke: ankle-positioned accelerometer can accurately record steps during slow walking. Phys Ther.

[53] Klassen TD, Semrau JA, Dukelow SP, Bayley MT, Hill MD, Eng JJ (2017). Consumer-based physical activity monitor as a practical way to measure walking intensity during inpatient stroke rehabilitation. Stroke.

[54] Mahendran N, Kuys SS, Downie E, Ng P, Brauer SG (2016). Are Accelerometers and GPS Devices Valid, Reliable and Feasible Tools for Measurement of Community Ambulation after Stroke?. Brain Impairment.

[55] Mandigout S, Lacroix J, Ferry B, Vuillerme N, Compagnat M, Daviet JC (2017). Can energy expenditure be accurately assessed using accelerometry-based wearable motion detectors for physical activity monitoring in post-stroke patients in the subacute phase?. Eur J Prev Cardiol.

[56] Negrini F, Gasperini G, Guanziroli E, Vitale JA, Banfi G, Molteni F. Using an Accelerometer-Based Step Counter in Post-Stroke Patients: Validation of a Low-Cost Tool. Int J Environ Res Public Health. 2020;17(9):3177. 10.3390/ijerph17093177 PMC7246942 32370210

[57] Polese JC, e Faria GS, Ribeiro-Samora GA, Lima LP, Coelho de Morais Faria CD, Scianni AA, Teixeira-Salmela LF. Google fit smartphone application or Gt3X Actigraph: Which is better for detecting the stepping activity of individuals with stroke? A validity study. J Bodyw Mov Ther. 2019;23(3):461–5. 10.1016/j.jbmt.2019.01.011 31563356

[58] Schaffer SD, Holzapfel SD, Fulk G, Bosch PR (2017). Step count accuracy and reliability of two activity tracking devices in people after stroke. Physiother Theory Pract.

[59] Shimizu N, Hashidate H, Ota T, Saito A (2018). The known-groups validity of intensity-based physical activity measurement using an accelerometer in people with subacute stroke. J Phys Ther Sci.

[60] Hei Chow C, Fraysse F, Hillier S (2023). The relationship between sleep and physical activity in an in-patient rehabilitation stroke setting: a cross-sectional study. Top Stroke Rehabil.

[61] Compagnat M, Salle JY, Vinti M, Joste R, Daviet JC (2022). The Best Choice of Oxygen Cost Prediction Equation for Computing Post-Stroke Walking Energy Expenditure Using an Accelerometer. Neurorehabil Neural Repair.

[62] Daniel CR, Yazbek P, Santos ACA, Battistella LR. Validity study of a triaxial accelerometer for measuring energy expenditure in stroke inpatients of a physical medicine and rehabilitation center. Top Stroke Rehabil. 2022;30(4):402–9. 10.1080/10749357.2022.2058292 35383539

[63] Garcia Oliveira S, Lourenço Nogueira S, Alex Matos Ribeiro J, Carnaz L, Regina Rocha Urruchia V, Alcantara CC, et al. Concurrent validity and reliability of an activity monitoring for rehabilitation (AMoR) platform for step counting and sitting/lying time in post-stroke individuals. Top Stroke Rehabil. 2021;29(2):103–13. 10.1080/10749357.2021.1886639 33605190

[64] Henderson CE, Toth L, Kaplan A, Hornby TG. Step Monitor Accuracy During PostStroke Physical Therapy and Simulated Activities. Transl J Am Coll Sports Med. 2021;7(1):e000186. 10.1249/tjx.0000000000000186 PMC9004549 35425853

[65] Holubová A, Malá E, Hoidekrová K, Pětioký J, Ďuriš A, Mužík J. The Accuracy of Commercially Available Fitness Trackers in Patients after Stroke. Sensors (Basel). 2022;22(19):7392. 10.3390/s22197392 PMC9573007 36236491

[66] Huber SK, Knols RH, Held JPO, Christen T, de Bruin ED (2022). Agreement, reliability, and concurrent validity of an outdoor, wearable-based walk ratio assessment in healthy adults and chronic stroke survivors. Front Physiol.

[67] Blondeel A, Demeyer H, Janssens W, Troosters T (2020). Accuracy of consumer-based activity trackers as measuring tool and coaching device in patients with COPD and healthy controls. PLoS One.

[68] Boeselt T, Spielmanns M, Nell C, Storre JH, Windisch W, Magerhans L, Beutel B, Kenn K, Greulich T, Alter P, Vogelmeier C (2016). Validity and Usability of Physical Activity Monitoring in Patients with Chronic Obstructive Pulmonary Disease (COPD). PLoS One.

[69] Danilack VA, Okunbor O, Richardson CR, Teylan M, Moy ML (2015). Performance of a pedometer to measure physical activity in a U.S. cohort with chronic obstructive pulmonary disease. J Rehabil Res Dev..

[70] Dhillon SS, Levy RD, Wilcox PG, Guenette JA, Quon BS, Ryerson CJ, Camp PG (2018). Physical activity measurement accuracy in advanced chronic lung disease. Can J Respir Crit Care Sleep Med.

[71] Farooqi N, Slinde F, Carlsson M, Håglin L, Sandström T (2015). Predicting energy requirement with pedometer-determined physical-activity level in women with chronic obstructive pulmonary disease. Int J Chron Obstruct Pulmon Dis.

[72] Juen J, Cheng Q, Schatz B (2015). A natural walking monitor for pulmonary patients using mobile phones. IEEE J Biomed Health Inform.

[73] Miyamoto S, Minakata Y, Azuma Y, Kawabe K, Ono H, Yanagimoto R, Suruda T (2018). Verification of a motion sensor for evaluating physical activity in COPD patients. Can Respir J.

[74] Prieto-Centurion V, Bracken N, Norwick L, Zaidi F, Mutso AA, Morken V, Coultas DB, Rand CS, Marquez DX, Krishnan JA (2016). Can commercially available pedometers be used for physical activity monitoring in patients with COPD following exacerbations?. Chronic Obstr Pulm Dis.

[75] Ummels D, Beekman E, Theunissen K, Braun S, Beurskens AJ (2018). Counting steps in activities of daily living in people with a chronic disease using nine commercially available fitness trackers: cross-sectional validity study. JMIR Mhealth Uhealth..

[76] Webster KE, Colabianchi N, Ploutz-Snyder R, Gothe N, Smith EL, Larson JL. Comparative assessment of ActiGraph data processing techniques for measuring sedentary behavior in adults with COPD. Physiol Meas. 2021;42(8):085006. 10.1088/1361-6579/ac18fe PMC8812274 34325404

[77] van der Weegen S, Essers H, Spreeuwenberg M, Verwey R, Tange H, de Witte L, Meijer K (2015). Concurrent validity of the MOX activity monitor compared to the ActiGraph GT3X. Telemed J E Health.

[78] Alexander S, Braisher M, Tur C, Chataway J (2022). The mSteps pilot study: Analysis of the distance walked using a novel smartphone application in multiple sclerosis. Mult Scler.

[79] Anens E, Ahlström I, Emtner M, Zetterberg L, Nilsagård Y, Hellström K (2023). Validity and reliability of physical activity measures in multiple sclerosis. Physiother Theory Pract.

[80] Lavelle G, Norris M, Flemming J, Harper J, Bradley J, Johnston H (2021). Validity and acceptability of wearable devices for monitoring step-count and activity minutes among people with multiple sclerosis. Front Rehabil Sci.

[81] Polhemus A, Sieber C, Haag C, Sylvester R, Kool J, Gonzenbach R (2023). Non-equivalent, but still valid: Establishing the construct validity of a consumer fitness tracker in persons with multiple sclerosis. PLOS Digit Health.

[82] Stuart CM, Varatharaj A, Domjan J, Philip S, Galea I (2020). Physical activity monitoring to assess disability progression in multiple sclerosis. Mult Scler J Exp Transl Clin.

[83] Balto JM, Kinnett-Hopkins DL, Motl RW (2016). Accuracy and precision of smartphone applications and commercially available motion sensors in multiple sclerosis. Mult Scler J Exp Transl Clin.

[84] Block VJ, Zhao C, Hollenbach JA, Olgin JE, Marcus GM, Pletcher MJ (2019). Validation of a consumer-grade activity monitor for continuous daily activity monitoring in individuals with multiple sclerosis. Mult Scler J Exp Transl Clin.

[85] Block VJL A, Crabtree-Hartman E, Bevan CJ, Graves JS, Bove R, Green AJ, Nourbakhsh B, Tremblay M, Gourraud PA, Ng MY, Pletcher MJ, Olgin JE, Marcus GM, Allen DD, Cree BA, Gelfand JM (2017). Continuous daily assessment of multiple sclerosis disability using remote step count monitoring. J Neurol.

[86] Coulter EH, Miller L, McCorkell S, McGuire C, Algie K, Freeman J, Weller B, Mattison PG, McConnachie A, Wu O, Paul L (2017). Validity of the activPAL3 activity monitor in people moderately affected by Multiple Sclerosis. Med Eng Phys.

[87] Zhai Y, Nasseri N, Pöttgen J, Gezhelbash E, Heesen C, Stellmann JP (2020). Smartphone accelerometry: a smart and reliable measurement of real-life physical activity in multiple sclerosis and healthy individuals. Front Neurol.

[88] Falter M, Budts W, Goetschalckx K, Cornelissen V, Buys R (2019). Accuracy of apple watch measurements for heart rate and energy expenditure in patients with cardiovascular disease: cross-sectional study. JMIR Mhealth Uhealth.

[89] Webber SC, John PD (2016). Comparison of ActiGraph GT3X+ and StepWatch Step Count Accuracy in Geriatric Rehabilitation Patients. J Aging Phys Act.

[90] Farmer C, van den Berg ME, Vuu S, Barr CJ (2022). A study of the accuracy of the Fitbit Zip in measuring steps both indoors and outdoors in a mixed rehabilitation population. Clin Rehabil.

[91] Alothman S, Hoover JC, Alshehri MM, Alenazi AM, Wick J, LeMaster J, et al. Test-Retest Reliability of activPAL in Measuring Sedentary Behavior and Physical Activity in People With Type 2 Diabetes. J Phys Activity Health. 2020;17(11):1134–9. 10.1123/jpah.2019-0506 32971519

[92] Arch ES, Sions JM, Horne J, Bodt BA (2018). Step count accuracy of StepWatch and FitBit One among individuals with a unilateral transtibial amputation. Prosthet Orthot Int.

[93] Ata RGN, Rasmussen H, El-Gabalawy O, Gutierrez S, Ahmad A, Suresh S, Ravi R, Rothenberg K, Aalami O. Clinical validation of smartphone-based activity tracking in peripheral artery disease patients. NPJ Digit Med. 2018;1(1):66. 10.1038/s41746-018-0073-x PMC6550212 31304343

[94] Collins JE, Yang HY, Trentadue TP, Gong Y, Losina E (2019). Validation of the Fitbit Charge 2 compared to the ActiGraph GT3X+in older adults with knee osteoarthritis in free-living conditions. Plos One.

[95] Jao YL, Gardner SE, Carr LJ (2017). Measuring weight-bearing activities in patients with previous diabetic foot ulcers. J Wound Ostomy Continence Nurs.

[96] Jayaraman C, Mummidisetty CK, Jayaraman A (2016). Effect of wearable sensor dynamics on physical activity estimates: a comparison between SCI vs. healthy individuals. Conf Proc IEEE Eng Med Biol Soc..

[97] Lai B, Sasaki JE, Jeng B, Cederberg KL, Bamman MM, Motl RW (2020). Accuracy and precision of three consumer-grade motion sensors during overground and treadmill walking in people with parkinson disease: cross-sectional comparative study. JMIR Rehabil Assistive Technol.

[98] Rossi A, Frechette L, Miller D, Miller E, Friel C, Van Arsdale A, Lin J, Shankar V, Kuo DY, Nevadunsky NS (2018). Acceptability and feasibility of a Fitbit physical activity monitor for endometrial cancer survivors. Gynecol Oncol.

[99] Semanik P, Lee J, Pellegrini CA, Song J, Dunlop DD, Chang RW (2020). Comparison of physical activity measures derived from the fitbit flex and the ActiGraph GT3X+ in an employee population with chronic knee symptoms. ACR Open Rheumatol.

[100] Shoemaker MJ, Cartwright K, Hanson K, Serba D, Dickinson MG, Kowalk A (2017). Concurrent validity of daily activity data from medtronic ICD/CRT devices and the actigraph GT3X triaxial accelerometer: a pilot study. Cardiopulm Phys Ther J.

[101] Smith JDGG, Burkholder BG. The validity and accuracy of wrist-worn activity monitors in lower-limb prosthesis users. Disabil Rehabil. 2019;42(22):3182–8. 10.1080/09638288.2019.1587792 30978125

[102] Van Blarigan EL, Kenfield SA, Tantum L, Cadmus-Bertram LA, Carroll PR, Chan JM (2017). The fitbit one physical activity tracker in men with prostate cancer: validation study. JMIR Cancer.

[103] Wendel N, Macpherson CE, Webber K, Hendron K, DeAngelis T, Colon-Semenza C, Ellis T (2018). Accuracy of activity trackers in parkinson disease: should we prescribe them?. Phys Ther.

[104] Cederberg KLJ, Jeng B, Sasaki JE, Lai B, Bamman M, Motl RW (2021). Accuracy and precision of wrist-worn actigraphy for measuring steps taken during over-ground and treadmill walking in adults with Parkinson's disease. Parkinsonism Relat Disord.

[105] Rockette-Wagner B, Saygin D, Moghadam-Kia S, Oddis C, Landon-Cardinal O, Allenbach Y (2021). Reliability, validity and responsiveness of physical activity monitors in patients with inflammatory myopathy. Rheumatology (Oxford).

[106] Saygin D, Rockette-Wagner B, Oddis C, Neiman N, Koontz D, Moghadam-Kia S (2022). Consumer-based activity trackers in evaluation of physical activity in myositis patients. Rheumatology (Oxford).

[107] Smith JD, Guerra G. Quantifying step count and oxygen consumption with portable technology during the 2-min walk test in people with lower limb amputation. Sensors (Basel). 2021;21(6):2080. 10.3390/s21062080 PMC7999204 33809581

[108] Albaum E, Quinn E, Sedaghatkish S, Singh P, Watkins A, Musselman K, Williams J. Accuracy of the Actigraph wGT3x-BT for step counting during inpatient spinal cord rehabilitation. Spinal Cord. 2019;57(7):571–8. 10.1038/s41393-019-0254-8. 10.1038/s41393-019-0254-8 30737452

[109] Daligadu J, Pollock CL, Carlaw K, Chin M, Haynes A, Thevaraajah Kopal T, Tahsinul A, Walters K, Colella TJ (2018). Validation of the fitbit flex in an acute post-cardiac surgery patient population. Physiother Can..

[110] McGinley SK, Armstrong MJ, Khandwala F, Zanuso S, Sigal RJ (2015). Assessment of the MyWellness Key accelerometer in people with type 2 diabetes. Appl Physiol Nutr Metab.

[111] Zbogar D, Eng JJ, Miller WC, Krassioukov AV, Verrier MC (2016). Reliability and validity of daily physical activity measures during inpatient spinal cord injury rehabilitation. SAGE Open Med.

[112] Chandrasekar A, Hensor EM, Mackie SL, Backhouse MR, Harris E (2018). Preliminary concurrent validity of the Fitbit-Zip and ActiGraph activity monitors for measuring steps in people with polymyalgia rheumatica. Gait Posture.

[113] Jimenez-Moreno AC, Charman SJ, Nikolenko N, Larweh M, Turner C, Gorman G (2019). Analyzing walking speeds with ankle and wrist worn accelerometers in a cohort with myotonic dystrophy. Disabil Rehabil.

[114] Ladlow P, Nightingale TE, McGuigan MP, Bennett AN, Phillip R, Bilzon JL (2017). Impact of anatomical placement of an accelerometer on prediction of physical activity energy expenditure in lower-limb amputees. PLoS One.

[115] Ladlow P, Nightingale TE, McGuigan MP, Bennett AN, Phillip RD, Bilzon JL (2019). Predicting ambulatory energy expenditure in lower limb amputees using multi-sensor methods. PLoS One..

[116] O'Brien CM, Duda JL, Kitas GD, Veldhuijzen van Zanten J, Metsios GS, Fenton SAM (2020). Measurement of sedentary time and physical activity in rheumatoid arthritis: an ActiGraph and activPAL™ validation study. Rheumatol Int.

[117] O'Neill BMSM, Wilson JJ, Bradbury I, Hayes K, Kirk A, Kent L, Cosgrove D, Bradley JM, Tully MA. Comparing accelerometer, pedometer and a questionnaire for measuring physical activity in bronchiectasis: a validity and feasibility study. Respir Res. 2017;18(1):16. 10.1186/s12931-016-0497-2 PMC5237513 28088206

[118] Roberts-Lewis SF, White CM, Ashworth M, Rose MR (2022). Validity of Fitbit activity monitoring for adults with progressive muscle diseases. Disabil Rehabil.

[119] Caron NPN, Caderby T, Verkindt C, Dalleau G. Accelerometry-based method for assessing energy expenditure in patients with diabetes during walking. J Hum Nutr Diet. 2019;32(4):531–4. 10.1111/jhn.12642 30916423

[120] Claridge EA, van den Berg-Emons RJG, Horemans HLD, van der Slot WMA, van der Stam N, Tang A (2019). Detection of body postures and movements in ambulatory adults with cerebral palsy: a novel and valid measure of physical behaviour. J Neuroeng Rehabil.

[121] Douma JAJVHMW, Buffart LM. Feasibility, validity and reliability of objective smartphone measurements of physical activity and fitness in patients with cancer. BMC Cancer. 2018;18(1):1052. 10.1186/s12885-018-4983-4 PMC6206914 30373549

[122] Herkert C, Kraal JJ, van Loon EMA, van Hooff M, Kemps HMC (2019). Usefulness of modern activity trackers for monitoring exercise behavior in chronic cardiac patients: validation study. JMIR Mhealth Uhealth.

[123] Pham MHEM, Haertner L, Deldin S, Srulijes K, Heger T, Synofzik M, Hobert MA, Faber GS, Hansen C, Salkovic D, Ferreira JJ, Berg D, Sanchez-Ferro A, van Dieen JH, Becker C, Rochester L, Schmidt G, Maetzler W (2017). Validation of a Step Detection Algorithm during Straight Walking and Turning in Patients with Parkinson's Disease and Older Adults Using an Inertial Measurement Unit at the Lower Back. Front Neurol..

[124] Van Laerhoven K, Hoelzemann A, Pahmeier I, Teti A, Gabrys L (2022). Validation of an open-source ambulatory assessment system in support of replicable activity studies. German J Exerc Sport Res.

[125] Popp WL, Schneider S, Bär J, Bösch P, Spengler CM, Gassert R (2019). Wearable sensors in ambulatory individuals with a spinal cord injury: from energy expenditure estimation to activity recommendations. Front Neurol.

[126] Femiano R, Werner C, Wilhelm M, Eser P (2022). Validation of open-source step-counting algorithms for wrist-worn tri-axial accelerometers in cardiovascular patients. Gait Posture.

[127] Thorup CB, Andreasen JJ, Sørensen EE, Grønkjær M, Dinesen BI, Hansen J (2017). Accuracy of a step counter during treadmill and daily life walking by healthy adults and patients with cardiac disease. BMJ Open.

[128] Gustafsson ME, Schiøttz-Christensen B, Wedderkopp N, Brønd JC (2022). Step count in patients with lumbar spinal stenosis: accuracy during walking and nonwalking activities. Spine (Phila Pa 1976)..

[129] Wagner SR, Gregersen RR, Henriksen L, Hauge EM, Keller KK. Smartphone Pedometer Sensor Application for Evaluating Disease Activity and Predicting Comorbidities in Patients with Rheumatoid Arthritis: a validation study. Sensors (Basel). 2022;22(23):9396. 10.3390/s22239396 PMC9735816 36502098

[130] Bianchini E, Caliò B, Alborghetti M, Rinaldi D, Hansen C, Vuillerme N, et al. Step-Counting Accuracy of a Commercial Smartwatch in Mild-to-Moderate PD Patients and Effect of Spatiotemporal Gait Parameters, Laterality of Symptoms, Pharmacological State, and Clinical Variables. Sensors (Basel). 2022;23(1):214. 10.3390/s23010214 PMC9823757 36616812

[131] Larkin L, Nordgren B, Purtill H, Brand C, Fraser A, Kennedy N (2016). Criterion validity of the activpal activity monitor for sedentary and physical activity patterns in people who have rheumatoid arthritis. Phys Ther.

[132] Ferreira J, Queirós A, Silva AG. Criterion validity of two mobile applications to count the number of steps in older adults with chronic pain. Euro J Physiother. 2020;23(5):325–30.

[133] de Carvalho Lana R, de Paula AR, Silva AF, Costa PH, Polese JC (2021). Validity of mHealth devices for counting steps in individuals with Parkinson's disease. J Bodyw Mov Ther.

[134] Nishida Y, Tanaka S, Nakae S, Yamada Y, Morino K, Kondo K (2020). Validity of the use of a triaxial accelerometer and a physical activity questionnaire for estimating total energy expenditure and physical activity level among elderly patients with type 2 diabetes mellitus: CLEVER-DM Study. Ann Nutr Metab.

[135] Takasaki H. Habitual pelvic posture and time spent sitting: Measurement test-retest reliability for the LUMOback device and preliminary evidence for slouched posture in individuals with low back pain. Sage Open Med. 2017;5:2050312117731251. 10.1177/2050312117731251 PMC5606340 28951781

[136] Yu SP, Ferreira ML, Duong V, Caroupapoullé J, Arden NK, Bennell KL (2022). Responsiveness of an activity tracker as a measurement tool in a knee osteoarthritis clinical trial (ACTIVe-OA study). Ann Phys Rehabil Med.

[137] Lamont RM, Daniel HL, Payne CL, Brauer SG (2018). Accuracy of wearable physical activity trackers in people with Parkinson's disease. Gait Posture..

[138] Salih SA, Peel NM, Burgess K (2016). Monitoring activity of inpatient lower limb prosthetic users in rehabilitation using accelerometry: validation study. J Rehabil Assist Technol Eng.

[139] Taoum A, Chaudru S, de Müllenheim PY, Congnard F, Emily M, Noury-Desvaux B (2021). Comparison of activity monitors accuracy in assessing intermittent outdoor walking. Med Sci Sports Exerc.

[140] Alharbi M, Bauman A, Neubeck L, Gallagher R (2016). Validation of Fitbit-Flex as a measure of free-living physical activity in a community-based phase III cardiac rehabilitation population. Eur J Prev Cardiol.

[141] Vetrovsky T, Siranec M, Marencakova J, Tufano JJ, Capek V, Bunc V (2019). Validity of six consumer-level activity monitors for measuring steps in patients with chronic heart failure. PLoS One.

[142] Freedson PS, Melanson E, Sirard J (1998). Calibration of the Computer Science and Applications Inc accelerometer. Med Sci Sports Exerc.

[143] Fuller D, Colwell E, Low J, Orychock K, Tobin MA, Simango B (2020). Reliability and validity of commercially available wearable devices for measuring steps, energy expenditure, and heart rate: systematic review. JMIR Mhealth Uhealth.

[144] Straiton N, Alharbi M, Bauman A, Neubeck L, Gullick J, Bhindi R (2018). The validity and reliability of consumer-grade activity trackers in older, community-dwelling adults: a systematic review. Maturitas.

[145] Vetrovsky T, Clark CCT, Bisi MC, Siranec M, Linhart A, Tufano JJ (2020). Advances in accelerometry for cardiovascular patients: a systematic review with practical recommendations. ESC Heart Fail.

[146] Carty C, van der Ploeg HP, Biddle SJH, Bull F, Willumsen J, Lee L (2021). The first global physical activity and sedentary behavior guidelines for people living with disability. J Phys Act Health.

[147] Bull FC, Al-Ansari SS, Biddle S, Borodulin K, Buman MP, Cardon G (2020). World Health Organization 2020 guidelines on physical activity and sedentary behaviour. Br J Sports Med.

[148] Migueles JH, Cadenas-Sanchez C, Ekelund U, Delisle Nystrom C, Mora-Gonzalez J, Lof M (2017). Accelerometer data collection and processing criteria to assess physical activity and other outcomes: a systematic review and practical considerations. Sports Med.

[149] Arvidsson D, Fridolfsson J, Borjesson M (2019). Measurement of physical activity in clinical practice using accelerometers. J Intern Med.

[150] Fini NA, Burge AT, Bernhardt J, Holland AE (2019). Two days of measurement provides reliable estimates of physical activity poststroke: an observational study. Arch Phys Med Rehabil..

[151] Shimizu N, Hashidate H, Ota T, Saito A (2018). Reliability of intensity-based physical activity measurement using an activity monitor in people with subacute stroke in the hospital setting: a cross-sectional study. Top Stroke Rehabil.

[152] Young LHM, Barnason S. Feasibility of Using Accelerometer Measurements to Assess Habitual Physical Activity in Rural Heart Failure Patients. Geriatrics (Basel). 2017;2(3):23. 10.3390/geriatrics2030023 PMC6371161 31011033

[153] DasMahapatra P, Chiauzzi E, Bhalerao R, Rhodes J (2018). Free-living physical activity monitoring in Adult US Patients with multiple sclerosis using a consumer wearable device. Digit Biomark.

[154] de Vet HCW, Terwee CB, Mokkink LB, Knol DL (2011). Measurement in medicine: a practical guide.

[155] Gao Z, Liu W, McDonough DJ, Zeng N, Lee JE. The dilemma of analyzing physical activity and sedentary behavior with wrist accelerometer data: challenges and opportunities. J Clin Med. 2021;10(24):5951. 10.3390/jcm10245951 PMC8706489 34945247

[156] Akkerman M, Mouton LJ, Disseldorp LM, Niemeijer AS, van Brussel M, van der Woude LHV (2018). Physical activity and sedentary behavior following pediatric burns - a preliminary investigation using objective activity monitoring. BMC Sports Sci Med Rehabil.

[157] Terwee CB, Jansma EP, Riphagen II, de Vet HCW (2009). Development of a methodological PubMed search filter for finding studies on measurement properties of measurement instruments. Qual Life Res.

