# Supplementary material for: Measurement properties of device-based physical activity instruments in ambulatory adults with physical disabilities and/or chronic diseases: a scoping review
Source: BMC Sports Sci Med Rehabil. 2023 Sep 21;15:115. doi: 10.1186/s13102-023-00717-0 (PMC10512652; doi:10.1186/s13102-023-00717-0)
Supplement: Supplementary file 1 — Additional file 1: Supplementary file 1. Protocol deviations. A files containing details of the deviations we made to the protocol. [file 13102_2023_717_MOESM1_ESM.docx]

# Supplementary file 1 – Protocol deviations

We originally planned to conduct a systematic review and the protocol has been created as such. However, during the review process we deviated from the protocol on a couple of points specified below. Firstly, the scope of the review was changed. Initially, the scope of the review was to include both self-reported and device-based instruments that assess PA in people with physical disabilities/chronic diseases. Due to the large number of studies on measurement properties in either type of instrument, we decided to shift the focus only on device-based instruments after the abstract screening phase. Furthermore, we decided to only include studies published from 2015 till date of search (20-11-2020). These decisions were mainly made for feasibility reasons. Secondly, we adjusted our research question from “what are the measurement properties” into “what is known about the measurement properties”. The reason for this adjustment was the large variability in studies, making it impossible to answer the original research question adequately. Consequently, we changed the method from a systematic review into a scoping review. A scoping review is more in line with the adjusted research question.
