# Supplementary material for: Measurement properties of device-based physical activity instruments in ambulatory adults with physical disabilities and/or chronic diseases: a scoping review
Source: BMC Sports Sci Med Rehabil. 2023 Sep 21;15:115. doi: 10.1186/s13102-023-00717-0 (PMC10512652; doi:10.1186/s13102-023-00717-0)
Supplement: Supplementary file 2 — Additional file 2: Supplementary file 2. Full search strategy for each database. A file containing the search strategy used for each database. [file 13102_2023_717_MOESM2_ESM.docx]

# Supplementary file 2 – Full search strategy for each database

**Search strategy**

A search will be performed in 4 databases: MEDLINE, EMBASE, CINAHL and Web of Science. Concepts used to formulate a search strategy are “Chronic disease and physical disability”, “physical activity measurement instruments” and “validity, reliability and responsiveness”.

**Search strategy for MEDLINE:**

(“Diabetes Mellitus”[mesh] OR diabet* [tiab]

OR

“Cardiovascular Diseases”[mesh] OR cardiac arrest [tiab] OR heart attack [tiab] OR heart failure[tiab] OR cardiomyopathy [tiab] OR cardiovascular disease* [tiab] OR claudication [tiab] OR arteriosclerosis [tiab] OR heart diseas* [tiab]

OR

“Neoplasms”[Mesh] OR cancer* [tiab]

OR

“Lung Diseases, Obstructive"[Mesh] OR asthma[tiab] OR COPD [tiab] OR obstructive pulmonary disease* [tiab] OR chronic bronchitis [tiab] OR emphysema [tiab]

OR

"Musculoskeletal Diseases"[Mesh] OR arthritis [tiab] OR amput*[tiab] OR rheuma*[tiab] OR lower back pain[tiab] OR low back pain[tiab] OR lower back dis*[tiab] OR osteoporos*[tiab] OR fibromy* [tiab] OR chronic pain*[tiab] OR Spondylarthritis[tiab] OR Amyotrophic Lateral Sclerosis [tiab] OR spinal muscular atrophy[tiab] OR musculoskeletal dis*[tiab]

OR

“Movement Disorders”[mesh] OR “Neuromuscular Diseases”[mesh] OR "Spinal Cord Injuries"[Mesh] OR spinal cord injur*[tiab] OR Paraplegi*[tiab] OR Quadriplegi*[tiab] OR Tetraplegi*[tiab] OR spine injur*[tiab] OR Parkinson*[tiab] OR cerebro vascular accident[tiab] OR cva[tiab] OR stroke[tiab] OR multiple sclerosis[tiab] OR spina bifida[tiab] OR myalgic encephalomyelitis[tiab] OR chronic fatigue syndrome[tiab] OR cerebral pals*[tiab] OR brain injur*[tiab]

OR

“Chronic Disease”[Mesh] OR “Wheelchairs”[mesh] OR chronic dis*[tiab] OR wheelchair*[tiab] OR disab*[tiab] OR rehabilitation[tiab] OR mobility impair*[tiab])

**AND**

(("Fitness Trackers"[Mesh] OR "Accelerometry"[Mesh] OR fitness track*[tiab] OR activity track*[tiab] OR wrist-mounted devic*[tiab] OR actigraph*[tiab] OR acceleromet*[tiab] OR pedomet*[tiab])

OR ((step-count*[tiab] OR physical activ*[tiab] OR energy expenditure[tiab]) AND (tracker*[tiab] OR tracking[tiab] OR monitor*[tiab]))

OR

(("Surveys and Questionnaires"[Mesh] OR Questionnair* [tiab] OR survey* [tiab] OR self report* [tiab] OR diary[tiab]) AND (physical activit* [tiab])))

**AND**( "Reproducibility of Results"[Mesh] OR reproducib*[tiab] OR valid*[tiab] OR reliab*[tiab] OR test-retest[tiab] OR accura*[tiab] OR “validation studies”[pt] OR responsiv*[tiab] OR psychometric*[tiab] OR “Psychometrics”[mesh])

**Search strategy for CINAHL**

**S1 =**

((MH “Diabetes Mellitus+”) OR TI”diabet*” OR AB” diabet*”

OR

(MH ”Cardiovascular Diseases+”) OR TI”cardiac arrest” OR AB”cardiac arrest” OR TI”heart attact” OR AB” heart attact” OR TI”heart failure” OR AB”heart failure” OR TI”cardiomyopathy” OR AB”cardiomyopathy” OR TI”cardiovascular disease*” OR AB”cardiovascular disease*” OR TI”claudication” OR AB”claudication” OR TI”arteriosclerosis” OR AB”arteriosclerosis” OR TI”heart diseas*” OR AB” heart diseas*”

OR

(MH ”Neoplasms+”) OR TI”cancer*” OR AB”cancer*”

OR

(MH “Lung Diseases, Obstructive+” OR TI”asthma” OR AB”asthma” OR TI”COPD” OR AB”COPD” OR TI”obstructive pulmonary disease*” OR AB”obstructive pulmonary disease*” OR TI”chronic bronchitis” OR AB”chronic bronchitis” OR TI”emphysema” OR AB”emphysema”

OR

(MH "Musculoskeletal Diseases+") OR TI”arthritis” OR AB” arthritis” OR TI”amput*” OR AB”amput*” OR TI”rheuma*” OR AB”rheuma*” OR TI”lower back pain” OR AB”lower back pain” OR TI”low back pain” OR AB”low back pain” OR TI”lower back dis*” OR AB”lower back dis*” OR TI”osteoporos*” OR AB”osteoporos*” OR TI”fibromy*” OR AB”fibromy*” OR TI”chronic pain*” OR AB”chronic pain*” OR TI”spondylarthritis” OR AB”spondylarthritis” OR TI”amyotrophic Lateral Sclerosis” OR AB”Amyotrophic Lateral Sclerosis” OR TI”spinal muscular atrophy” OR AB”spinal muscular atrophy” OR TI”musculoskeletal dis*” OR AB”musculoskeletal dis*”

OR

(MH ”Movement Disorders+”) OR (MH “Neuromuscular Diseases+”) OR (MH ”Spinal Cord Injuries+”) OR TI”spinal cord injur*” OR AB”spinal cord injur*” OR TI”paraplegi*” OR AB”paraplegi*” OR TI”quadriplegi*” OR AB”quadriplegi*” OR TI”tetraplegi*” OR AB”tetraplegi*” OR TI”spine injur*” OR AB”spine injur*” OR TI”Parkinson*” OR AB”Parkinson*” OR TI”cerebro vascular accident” OR AB”cerebro vascular accident” OR TI”cva” OR AB”cva” OR TI”stroke” OR AB”stroke” OR TI”multiple sclerosis” OR AB”multiple sclerosis” OR TI”spina bifida” OR AB”spina bifida” OR TI”myalgic encephalomyelitis” OR AB”myalgic encephalomyelitis” OR TI”chronic fatigue syndrome” OR AB”chronic fatigue syndrome” OR TI”cerebral pals*” OR AB”cerebral pals*” OR TI”brain injur*” OR AB”brain injur*”

OR

(MH “Chronic Disease+”) OR (MH “Wheelchair+”) OR TI”chronic dis*” OR AB”chronic dis*” OR TI”wheelchair*” OR AB”wheelchair*” OR TI”disab*” OR AB”disab*” OR TI”rehabilitation” OR AB”rehabilitation” OR TI”mobility impair*” OR AB”mobility impair*” )

**S2=**

(MH “Fitness Trackers+”) OR (MH “Accelerometry+”) OR TI”fitness track*” OR AB”fitness track*” OR TI”activity track*” OR AB”activity track*” OR TI”wrist-mounted devic*” OR AB”wrist-mounted devic*” OR TI”actigraph*” OR AB”actigraph*” OR TI”acceleromet*” OR AB”acceleromet*” OR TI”pedomet*” OR AB”pedomet*”

**S3=**

TI”step-count*” OR AB”step-count*” OR TI”physical activ*” OR AB”physical activ*” OR TI”energy expenditure” OR AB”energy expenditure”

**S4=**

TI”tracker*” OR AB”tracker*” OR TI”tracking” OR AB”tracking” OR TI”monitor*” OR AB”monitor*”

**S5=**

(MH “Surveys+”) OR (MH Questionnaires+”) OR TI”questionnaire*” OR AB”questionnaire*” OR TI”survey*” OR AB”survey*” OR TI”self report*” OR AB”self report*” OR TI”diary” OR AB”diary”

**S6=**

TI”physical activit*” OR AB”physical activit*”

**S7=**

(MH “Reproducibility of Results+”) OR TI”reproducib*” OR AB”reproducib*” OR TI”valid*” OR AB”valid*” OR TI”reliab*” OR AB”reliab*” OR TI”test-retest*” OR AB”test-retest*” OR TI”accura*” OR AB”accura*” OR PT”validation studies” OR TI”responsiv*” OR AB”responsiv*” OR TI”psychometric*” OR AB”psychometric*” OR (MH “Psychometrics+”) OR (MH “[Reliability and Validity](http://web.a.ebscohost.com/ehost/mesh/tree?term=Reliability%20and%20Validity&sid=7b3a2845-a16d-4f3c-a5ab-706fef9a3480%40sdc-v-sessmgr05&vid=76)+”)

1. Each searchterm (S1 till S7) separately
2. S3 AND S4 --> S8
3. S5 AND S6 --> S9
4. S2 OR S8 OR S9 --> S10
5. S1 AND S7 AND S10

**Search strategy Web of Science**

(TS=”diabetes mellitus” OR TS=diabet*

OR

TS=”Cardiovascular Diseases” OR TS=”cardiac arrest” OR TS=”heart attack” OR TS=”heart failure” OR TS=cardiomyopathy OR TS=”cardiovascular disease*” OR TS=claudication OR TS=arteriosclerosis OR TS=”heart diseas*”

OR

TS=neoplasms OR TS=cancer*

OR

TS=”obstructive pulmonary disease*” OR TS=asthma OR TS= COPD OR TS=chronic bronchitis OR TS=emphysema

OR

TS=”musculoskeletal dis*” OR TS=arthritis OR TS=amput* OR TS=rheuma* OR TS=”low* back pain” OR TS=”low* back dis*” OR TS=osteoporos* OR TS=fibromy* OR TS=”chronic pain*” OR TS=spondylarthritis OR TS=”amyotrophic lateral sclerosis” OR TS=”spinal muscular atrophy”

OR

TS = “movement dis*” OR TS=”neuromuscular dis*” OR TS=”spinal cord injur*” OR TS=paraplegi* OR TS=quadriplegi* OR TS=tetraplegi* OR TS=”spine injur*” OR TS=Parkinson* OR TS=”cerebro vascular accident” OR TS=CVA OR TS=stroke OR TS=”multiple sclerosis” OR TS=”spina bifida” OR TS=”myalgic encephalomyelitis” OR TS=”chronic fatigue syndrome” OR TS=”cerebral pals*” OR TS=”brain injur*”

OR

TS=”chronic dis*” OR TS=wheelchair* OR TS=disab* OR TS=rehabilitation OR TS=”mobility impair*”)

**AND**

((TS=”fitness track*” OR TS=acceleromet* OR TS=”activity track*” OR TS=”wrist-mounted divic*” OR ((TS=”step-count*” OR TS=”physical activ*”) AND (TS=tracker* OR TS=tracking OR TS=monitor*)) OR TS=actigraph* OR TS=acceleromet* OR TS=pedomet*)

OR

((TS=servey* OR TS=questionnaire* OR TS=”self report*” OR TS=diary) AND (TS=”physical activit*”)))

**AND**

(TS=”reproducibility of result*” OR TS=reproducib* OR TS=valid* OR TS=reliab* OR TS=”test-retest” OR TS=accura* OR TS=responsive* OR TS=psychometric*)

**Search strategy Embase**

('diabetes mellitus'/exp OR diabet*:ab,ti,kw

OR

'cardiovascular disease'/exp OR 'cardiac arrest':ab,ti,kw OR 'heart attack':ab,ti,kw OR 'heart failure':ab,ti,kw OR cardiomyopathy:ab,ti,kw OR 'cardiovascular disease*':ab,ti,kw OR claudication:ab,ti,kw OR arteriosclerosis:ab,ti,kw OR 'heart diseas*':ab,ti,kw

OR

'neoplasm'/exp OR cancer:ab,ti,kw

OR

'chronic obstructive lung disease'/exp OR asthma:ab,ti,kw OR COPD:ab,ti,kw OR ‘obstructive pulmonary disease*’:ab,ti,kw OR ‘chronic bronchitis’:ab,ti,kw OR emphysema:ab,ti,kw

OR

'musculoskeletal disease'/exp OR arthritis:ab,ti,kw OR amput*:ab,ti,kw OR rheuma*:ab,ti,kw OR ‘lower back pain’:ab,ti,kw OR ‘low back pain’:ab,ti,kw OR ‘lower back dis*’:ab,ti,kw OR osteoporos*:ab,ti,kw OR fibromy*:ab,ti,kw OR ‘chronic pain*’:ab,ti,kw OR Spondylarthritis:ab,ti,kw OR ‘Amyotrophic Lateral Sclerosis’:ab,ti,kw OR ‘spinal muscular atrophy’:ab,ti,kw OR ‘musculoskeletal dis*’:ab,ti,kw

OR

'motor dysfunction'/exp OR 'neuromuscular disease'/exp OR 'spinal cord injury'/exp OR ‘spinal cord injur*’:ab,ti,kw OR Paraplegi*:ab,ti,kw OR Quadriplegi*:ab,ti,kw OR Tetraplegi*:ab,ti,kw OR ‘spine injur*’:ab,ti,kw OR Parkinson*:ab,ti,kw OR ‘cerebro vascular accident’:ab,ti,kw OR cva:ab,ti,kw OR stroke:ab,ti,kw OR ‘multiple sclerosis’:ab,ti,kw OR ‘spina bifida’:ab,ti,kw OR ‘myalgic encephalomyelitis’:ab,ti,kw OR ‘chronic fatigue syndrome’:ab,ti,kw OR ‘cerebral pals*’:ab,ti,kw OR ‘brain injur*’:ab,ti,kw

OR

'chronic disease'/exp OR 'wheelchair'/exp OR ‘chronic dis*’:ab,ti,kw OR wheelchair*:ab,ti,kw OR disab*:ab,ti,kw OR rehabilitation:ab,ti,kw OR ‘mobility impair*’:ab,ti,kw)

**AND**

('activity tracker'/exp OR 'accelerometry'/exp OR 'fitness track*':ab,ti,kw OR 'activity track*':ab,ti,kw OR 'wrist-mounted devic*':ab,ti,kw

OR

(('step-count*':ab,ti,kw OR 'physical activ*':ab,ti,kw) AND (tracker*:ab,ti,kw OR tracking:ab,ti,kw OR monitor*:ab,ti,kw OR actigraph*:ab,ti,kw OR acceleromet*:ab,ti,kw OR pedomet*:ab,ti,kw))

OR

(('questionnaire'/exp OR Questionnair*:ab,ti,kw OR survey*:ab,ti,kw OR ‘self report*’:ab,ti,kw OR diary:ab,ti,kw) AND (‘physical activit*’:ab,ti,kw)))

**AND**

('reproducibility'/exp OR reproducib*:ab,ti,kw OR valid*:ab,ti,kw OR reliab*:ab,ti,kw OR ‘test-retest’:ab,ti,kw OR accura*:ab,ti,kw OR 'validation study'/exp OR responsiv*:ab,ti,kw OR psychometric*:ab,ti,kw)

**AND**

[embase]/lim NOT [medline]/lim
