# Supplementary material for: Measurement properties of device-based physical activity instruments in ambulatory adults with physical disabilities and/or chronic diseases: a scoping review
Source: BMC Sports Sci Med Rehabil. 2023 Sep 21;15:115. doi: 10.1186/s13102-023-00717-0 (PMC10512652; doi:10.1186/s13102-023-00717-0)
Supplement: Supplementary file 3 — Additional file 3: Supplementary file 3. PICO, Selection criteria and Checklists. A file containing the PICO and the selection criteria used for the scoping review. Als contains the checklist that were used during the screening process. [file 13102_2023_717_MOESM3_ESM.docx]

# Supplementary file 3 – PICO, Selection criteria and Checklists

**PICO**

**P =** Adults (≥18y) with physical disabilities and/or chronic diseases

- ***Physical disability****: a congenital disease, acquired illness, or trauma that causes an impairment, activity limitation and participation restriction that lasts at least 1 year (Liou et al. 2005 combined with ICF model)*
- ***Chronic disease****: chronic diseases are defined broadly as conditions that last 1 year or more and require ongoing medical attention or limit activities of daily living or both (NCCDPHP)*
  - A cardiovascular disease, such as arteriosclerosis, claudication, cardiomyopathy, heart failure, heart attack
  - Cancer
  - A respiratory disease such as Asthma, COPD, chronic bronchitis, emphysema
  - A musculoskeletal disease/disability such as arthritis, amputation, fibromyalgia, osteoporosis, lower back pain, spondylarthritis, ALS, SMA
  - Neurological or neuromuscular disease such as SCI, Parkinson’s disease, CVA, stroke, MS, spina bifida, CP, chronic fatigue syndrome
- The physical disability or chronic disease must be a primary reason for rehabilitation treatment

**I** = Objective and subjective measurement instrument assessing the construct physical activity.

- ***Physical activity****: any bodily movement produced by the muscles that results in increased energy expenditure (Caspersen et al. 1985) in the activity domain of the ICF model*
- ***Objective measurement instrument****: accelerometer, pedometer, multisensory devices (e.g. accelerometer combined with heartrate)* *measuring* *steps, counts, energy expenditure, MET, time spent physically active, time spent in different activity modalities etc.*
- ***Subjective measurement instrument****: Questionnaire, diary measuring energy expenditure, MET, time spent physically active, time spent in different activity modalities etc.*

**C** = NA

**O** = Measurement properties

- **Validity**: Content validity, criterion validity (e.g. sensitivity and specificity, ROC, Kappa, Spearman’s or Pearson’s r, Bland-Altman limits of agreement, ICC), construct validity (e.g. factor analysis).
- **Reliability**: e.g. Cohen’s kappa, ICC, Cronbach’s alpha, Bland-Altman plots
- **Responsiveness**: e.g. e.g. sensitivity and specificity, ROC, Kappa, Spearman’s or Pearson’s r, Bland-Altman limits of agreement, ICC

**Type of studies to include**: Cross-sectional, Cohort (baseline measurement), Intervention studies (RCT, quasi-RCT, before-after studies)

**Type of publication to include**: Peer-reviewed articles describing primary research

**Language restrictions (only with full text screening)**: English and Dutch

**Selection criteria**

***Overall selection criteria:***

**Inclusion criteria:**

1. Participants are 18 years or older
2. Participants have a physical disability or chronic disease which is a primary reason for rehabilitation treatment. See list for physical disabilities and chronic diseases which can be included (but not limited to)
   - A cardiovascular disease, such as arteriosclerosis, claudication, cardiomyopathy, heart failure, heart attack
   - Cancer
   - A respiratory disease such as Asthma, COPD, chronic bronchitis, emphysema
   - A musculoskeletal disease/disability such as arthritis, amputation, fibromyalgia, osteoporosis, lower back pain, spondylarthritis, ALS, SMA
   - Neurological or neuromuscular disease such as SCI, Parkinson’s disease, CVA, stroke, MS, spina bifida, CP, chronic fatigue syndrome
3. Physical activity is measured
   1. The measured physical activity should be an amount or energy cost (e.g. step-count, counts, energy expenditure, MET, time spent physically active, time spent in different activity modalities)
4. Validity, reliability or responsiveness study, meaning that
   1. Data on one of these constructs should be available
   2. A comparison made with a criterion measure or gold standard for criterion validity outcomes
   3. A comparison with itself for reliability outcomes
   4. A comparison of the change score with a criterion measure or gold standard for responsiveness outcomes
5. Study type: peer-reviewed articles describing primary research
6. Full text available

**Exclusion criteria:**

1. Studies not in humans
2. People with an intellectual disability, sensory disability (e.g. visual or auditory), cognitive disability (e.g. Alzheimer’s disease), mental disability (e.g. depression)
3. Total population is wheelchair dependend
4. Studies measuring functional or performance outcomes (e.g. gait characteristics, walking ability)
5. Case studies, protocol articles, intervention studies not considering validity, reliability or responsiveness
6. Articles not in English or Dutch

**Study selection**

**Title screening:**

Because of the broadness of the search strategy and large number of results, we will conduct a first screening based on title alone. Based on the selection criteria mentioned above, a checklist with broadly defined inclusion criteria and precise exclusion criteria is developed for the title screening phase. Two assessors piloted the checklist, and uncertainties were discussed and straighten out. Using the improved checklist (see below), the same two assessors will independently screen the titles to identify studies that potentially meet the inclusion criteria. Titles on which disagreement exits will be included for the abstract screening phase.

*Title screening checklist:*

| **Title screening** |  |  |  |  |
| --- | --- | --- | --- | --- |
| **Assessor** | **Inclusion criteria number** | **Yes** | **No** | **Unclear** |
| **Patient/participants:**  Where patients/participants aged ≥ 18 years? | **1** |  | Only **no** when term children or age range <18 |  |
| Do the patients/participants have a chronic disease/physical disability? (see list) | **2** |  | Only **no** when population specified is healthy/able bodied. |  |
| **Method:**  Is physical activity measured? | **3** |  | Only **no** when PA or related terms (e.g. energy expenditure, active lifestyle) are not mentioned |  |
| Is it a validity, reliability or responsiveness study? | **4** |  |  |  |
| Is it a peer-reviewed article describing primary research? | **5** |  | Only **no** when term review is in title |  |
|  | **Exclusion criteria number** | **Yes** | **No** | **Unclear** |
| **Patients/participants:**  Is the study done in anything other than humans? | **1** | Only **Yes** when explicitly stated animal study or computer model without participants |  |  |
| Do the patients/participants have an intellectual, sensory, cognitive or mental disability | **2** | Only **Yes** when explicitly stated that patients have such a disability |  |  |
| **Method:**  Is physical activity measured as a functional or performance outcome? | **3** | Performance e.g. 10 meter walking test, timed up and go  Functional e.g. gait analysis, range of motion |  |  |
| **Study type:**  Is it a case study, protocol article or an intervention study not considering validity, reliability or responsiveness? | **4** |  |  |  |

**If no no’s on inclusion criteria and no yes’s on exclusion criteria, include for abstract screening. Unclears will be taken to abstract screening**

**Abstract screening:**

All records identified in the in title screening will be assessed on abstract for inclusion for full text screening. This will be done by two assessors independently, of which at least one was involved in the title screening. They will use a more detailed checklist (see below). Before the start of screening a calibration session will be held on how to use and interpret the checklist. Intra-rater agreement for title/abstract screening will be calculated as % overall agreement and Cohen’s Kappa (K: 0.00 – 0.20 = slight; 0.21 – 0.40 = fair; 0.41 – 0.60 = moderate; 0.61 – 0.80 = good; 0.81 – 1.00 = very good (13)). Studies on which disagreement exist, will be discussed in a consensus meeting. If disagreement remains, include for the next phase.

*Abstract screening checklist:*

| **Abstract screening** |  |  |  |  |
| --- | --- | --- | --- | --- |
| **Assessor** | **Inclusion criteria number** | Yes | No | Unclear |
| **Patient/participants:**  Are patients/participants aged ≥ 18 years? | **1** | Adults, age range > 18 years | Children, adolescents, age range includes < 18 | When age range is not mentioned |
| Do the patients/participants have a chronic disease (CD)/physical disability (PD)?^1^ | **2** |  | If no mention of CD/PD/patients at all | If mention of CD/PD/patients in intro, but not specified in method part. |
| **Method:**  Is physical activity measured? | **3** | Specified activities, such as mode of travel (biking, swimming etc.), mode of posture (standing, sitting, lying) also included |  | if PA could be a subscale of the used measurement instrument, such as total lifestyle questionnaire |
| Is physical activity measured as an amount or energy cost (quantified)? (e.g. step-count, counts, METs, energy expenditure, time spent physically active etc.) | **3.a** | If it is measured as an amount | If it is measured as a classification (i.e. active/not active), or if it is predicted | If it is unclear how PA is measured. |
| Is the instrument being tested on validity, reliability or responsiveness | **4** |  | If no mention of validity, reliability or responsiveness (clinometric aspects) at all | The term accuracy can be used for validity or reliability, if not sure how it is used put unclear |
| **Outcome:**  Is there data available on the validity, reliability or responsiveness of the instrument | **4.a** |  |  | If validity, reliability or responsiveness is mentioned, but no clear data is given |
| **Study type:**  Is it a peer-reviewed article describing primary research | **5** |  | if it is clear the article is not presenting original data (e.g. reviews, conference abstracts) |  |
|  | **Exclusion criteria number** | Yes | No | Unclear |
| **Patients/participants:**  Is the study done in anything other than humans? | **1** | If stated no participants/ computer simulation/ animals |  |  |
| Do the patients/participants have an intellectual, sensory, cognitive or mental disability | **2** | If explicitly stated |  |  |
| **Method:**  Is physical activity measured as a functional or performance outcome? (e.g. gait characteristics, walking ability, range of motion) | **4** |  |  |  |
| **Study type:**  Is it a case study, protocol article or a RCT not considering validity, reliability or responsiveness? | **5** | If stated it is a case study/ protocol article |  |  |
| **Abstract not in Dutch or English** | **6** |  |  |  |

^1^ Definitions of physical disability and chronic disease:

*Physical disability: a congenital disease, acquired illness, or trauma that causes an impairment, activity limitation and participation restriction that lasts at least 1 year (Liou et al. 2005 combined with ICF model)*

*Chronic disease: chronic diseases are defined broadly as conditions that last 1 year or more and require ongoing medical attention or limit activities of daily living or both (NCCDPHP)*

List of included chronic diseases/physical disabilities

- A cardiovascular disease, such as arteriosclerosis, claudication, cardiomyopathy, heart failure, heart attack
- Cancer
- A respiratory disease such as Asthma, COPD, chronic bronchitis, emphysema
- A musculoskeletal disease/disability such as arthritis, amputation, fibromyalgia, osteoporosis, lower back pain, spondylarthritis, ALS, SMA
- Neurological or neuromuscular disease such as SCI, Parkinson’s disease, CVA, stroke, MS, spina bifida, CP, chronic fatigue syndrome
- Wheelchair dependent
- Diabetes

**If there are no No’s in the inclusion criteria, and no Yes’s in the exclusion criteria, include article for full text phase.**

**Full text screening:**

Studies on which disagreement exist, will be discussed in a consensus meeting. A third assessor will be consulted in case no consensus was obtained after the consensus meeting.

*Checklist full-text screening*

| **Fulltext screening** |  |  |  |
| --- | --- | --- | --- |
| **Assessor** | **Inclusion criteria number** |  |  |
| **Patient/participants:**  Where patients/participants aged ≥ 18 years? | **1** | Yes | No |
| Do the patients/participants have a chronic disease/physical disability? (see list) | **2** | Yes | No |
| **Method:**  Is physical activity measured? | **3** | Yes | No |
| Is physical activity measured as an amount or energy cost? (e.g. step-count, counts, METs, energy expenditure, time spent physically active etc) | **3.a** | Yes | No |
| Is the instrument being tested on validity, reliability or responsiveness? | **4** | Yes | No |
| **Outcome:**  Is there data available on the validity, reliability or responsiveness of the instrument? | **4.a** | Yes | No |
| *Criterion validity (leave empty if not measured):* Comparison with a gold standard or criterion measure? | **4.b** | Yes | No |
| *Reliability (leave empty if not measured):* Comparison with itself? | **4.c** | Yes | No |
| *Responsiveness (leave empty if not measured):* comparison of change score with a gold standard or criterion measure? | **4.d** | Yes | No |
| **Study type:**  Is it a peer-reviewed article describing primary research? | **5** | Yes | No |
| Is full text available | **6** | Yes | No |
|  | **Exclusion criteria number** |  |  |
| **Patients/participants:**  Is the study done in anything other than humans? | **1** | Yes | No |
| Do the patients/participants have an intellectual, sensory, cognitive or mental disability | **2** | Yes | No |
| **Method:**  Is physical activity measured as a functional or performance outcome? | **3** | Yes | No |
| **Study type:**  Is it a case study, protocol article or a RCT not considering validity, reliability or responsiveness? | **4** | Yes | No |
| Is the article not in English or Dutch | **5** | Yes | No |

List of included chronic diseases/physical disabilities

- A cardiovascular disease, such as arteriosclerosis, claudication, cardiomyopathy, heart failure, heart attack
- Cancer
- A respiratory disease such as Asthma, COPD, chronic bronchitis, emphysema
- A musculoskeletal disease/disability such as arthritis, amputation, fibromyalgia, osteoporosis, lower back pain, spondylarthritis, ALS, SMA
- Neurological or neuromuscular disease such as SCI, Parkinson’s disease, CVA, stroke, MS, spina bifida, CP, chronic fatigue syndrome

**If an article scores a No on the inclusion criteria, it is excluded in the full text phase. If it scores a Yes on the exclusion criteria, it is also excluded in the full text phase.**
