# Supplementary material for: Measurement properties of device-based physical activity instruments in ambulatory adults with physical disabilities and/or chronic diseases: a scoping review
Source: BMC Sports Sci Med Rehabil. 2023 Sep 21;15:115. doi: 10.1186/s13102-023-00717-0 (PMC10512652; doi:10.1186/s13102-023-00717-0)
Supplement: Supplementary file 4 — Additional file 4: Supplementary file 4. Expanded description of the 103 studies included in the scoping review. A file containing the description of the included studies in more detail. Extra information on in- and exclusion criteria, the used task in the study and criteria for valid days and cases. [file 13102_2023_717_MOESM4_ESM.docx]

# Supplementary file 4 – Expanded description of the 103 studies included in the scoping review

| Author | Year | Country | Study design | Population | Sample size | Study setting | In- & exclusion criteria | % Male | Age (mean ± sd or median (range)) | Task | Walking speed (mean ± sd or median (range)) | Valid days + cases |
| --- | --- | --- | --- | --- | --- | --- | --- | --- | --- | --- | --- | --- |
| Albaum et al. | 2019 | Canada | Cross sectional | Incomplete SCI | 17 | Free-living | 1) sustained motor iSCI < 6months 2) 18 y or older | 76.5% | 62.0 (41.5 - 78.5) | Physical therapy session & self-directed tasks | N.R. | N.R. |
| Alexander et al. | 2022 | UK | Cross sectional | MS | 100 | Lab | In  1) Confirmed MS diagnosis  2) >= 18 yrs | 30.0% | 53.5 (47.8-58.0) | Outdoors circuit | Comfortable | N.A. |
| Alharbi et al. | 2016 | Australia | Cross sectional | Coronary heart disease | 28 | Free-living | In: 1) CHD and completed phase II cardiac rehab program | 71.4% | N.R. for only CHD patients | 4 days free-living activities | N.R. | N.R. |
| Alothman et al. | 2020 | USA | Longitudinal | Diabetes mellitus (type 2) | 30 | Free-living | In: 1) English speakers between 50-75 yrs old 2) Self-reported diagnosis DM type 2 3) Able to ambulate independently without assistive device for at least 50m | 36.7% | 64.87 ± 5.99 | 7 days free-living activities (twice) | N.R. | ≥10 hour for ≥4 days |
| Anens et al. | 2023 | Sweden | Cross sectional | MS | 30 | Lab | In  1) MS  2) able to walk 10 min with or without aids  Ex  1) Pregnancy  2) Relapse MS <2months  3) severe cognitive problems  4) serious comorbidity affecting walking ability | 30.0% | 49.2 ± 14.0 | Circuit + sedentary activities | Slow (0.76 IQR 0.31) comfortable (1.02 IQR 0.30)  fast (1.30 IQR 0.39) m/s | N.A. |
| Arch et al. | 2018 | USA | Cross sectional | Amputation (lower limb (transtibial), unilateral) | 50 | Lab | In: 1) Prosthetic users 2) Aged 18-85 years 3) Unilateral transtibial amputation | Differs per task: 64% - 76% | Differs per task: 55.4 ± 10.1 – 58.6 ± 11.7 | Circuit:  - 6 MWT  - Four-square step test & Figure-of-8 walk test | 0.95 ± 0.21 - 1.01 ± 0.19 | N.A. |
| Ata et al. | 2018 | USA | Cross sectional | Peripheral Aterial Disease | 114 | Lab | In: 2) 18 yrs or older | 77.2% | 69.5 ± 13.1 | 6MWT | N.R. | N.A. |
| Balto et al. | 2016 | USA | Cross sectional | MS | 45 | Lab | 1) Neurologist confirmed MS 2) age 18-64 4) Ability to walk 500 steps without assistive device | N.R. | 46.7 ± 10.0 | treadmill 500 steps | 1.21 ± 0.27 m/s | N.A. |
| Bianchini et al. | 2022 | Italy | Cross sectional | PD | 47 | Lab | in  1) >=18 yrs  2) ability to walk  4) idiopathic PD  ex  1) presence of FOG" | 67.0% | 66.3 ± 8.2 | 6MWT | N.R. | N.A. |
| Block et al. | 2017 | USA | Cross sectional | MS | 82 | Lab + Free-living | In: 1) MS 2) 18 yrs or older 3) able to walk at least 2 min with or without assistive device | 29.3% | 51.0 ± 13.7 | 2MWT and 7 days free-living activities | N.R. | >300 steps for ≥1 day |
| Block et al. | 2019 | USA | Cohort (part of) | MS | 61 | Lab | In: 1) Definite diagnosis of MS 2) 18 yrs or older 3) Ability to walk at least 2 min with or without assistive devices | 28.0% | 50.0 ± 14.4 | 2 MWT | N.R. | ≥10 hour for ≥3 days |
| Block et al. | 2019 |  |  |  | 31 | Free-living |  | 41.9% | 53.4 ± 11.7 | 7 days free-living activities | N.R. | N.A. |
| Blondeel et al. | 2020 | Belgium | Cross sectional | COPD | 30 | Free-living | In: 1) Diagnosis of COPD confirmed by spirometry 2) Older than 40 yrs |  |  | 14 days free-living activities | N.R. | ≥8 hour for ≥3 matching days of all instruments |
| Boeselt et al. | 2016 | Germany | Cross sectional | COPD | 20 | Free-living | In: 1) COPD stage I to IV 2) Ability to walk 3) Age 40 to 90 | 85.0% | 66.4 ± 7.4 | 3 days free-living activities | N.R. | N.R. |
| Campos et al. | 2018 | Canada | Cross sectional | Stroke | 33 | Lab | 1) Subacute stroke in inpatient stroke rehab program 2) able to walk without physical assistance from another person | 69.7% | 64.9 ± 14.7 | 7 hours on a single day | 0.82 ± 0.27 | N.A. |
| Caron et al. | 2019 | France | cross sectional | Diabetes mellitus (type 2) | 20 | Lab | N.R. | 40.0% | 57.5 ± 8.4 | Treadmill | 0.50, 0.75, 1.00, 1.25 & 1.50 m/s | N.A. |
| Cederberg et al. | 2021 | USA | Cross sectional | PD | 29 | lab | In  1) aged 50-74  2) confirmed idiopathic PD  3) ambulatory without assistance  ex  1) motor symptoms due to neuroleptic medication  2) stroke  3) non responsive to dopaminergic medication | 62.0% | 64.2 ± 6.4 | 6MWT circuit + treadmill | 1.03 ± 0.18 m/s | N.A. |
| Chandrasekar et al. | 2018 | UK | Cross sectional | Polymyalgia rheumatica | 27 | Lab | 1) Confirmed diagnosis of Polymyalgia rheumatica 2) Treated with systemic glucocorticoids at least 1 month | 11.0% | 69.2 ± 8.8 | 2 MWT & stairs test | 1.19 (IQR 0.95-1.31) | N.A. |
| Claridge et al. | 2019 | Netherlands | Cross sectional | Cerebral palsy | 14 | Lab | In: 1) 18 yrs or older 2) Diagnosis of spastic CP 3) Ambulatory, with or without use of assistive devices | 60.0% | 35.4 ± 13.1 | Circuit (meaningful activities of daily life in individuals with CP | N.R. | N.A. |
| Clay et al. | 2019 | New Zealand | Cross sectional | Stroke | 19 | Lab | In: 1) Clinical diagnosis of stroke 3) Independently ambulant with or without assistive devices | 42.0% | 65.6 ± 8.2 | 6MWT | Self-selected (0.97 ± 0.22) | N.A. |
| Collins et al. | 2019 | USA | cross sectional | Osteoarthritis (knee) | 15 | Free-living | Inclusion 1) age between 50 and 85 y 2) primary diagnosis of knee OA | 33.0% | 68 ± 8 | Waking hours | N.R. | ≥10 hour for ≥4 days |
| Compagnat et al. | 2018 | France | Cross sectional | Stroke | 35 | Lab | Inclusion 1) Stroke survivors (ischemic or hemorrhagic) 2) ≥18y 3) move alone with or without ambulatory aid | N.R. | 64.4 ± 14.4 | 4 tasks:  1) combination of transfers 2) manual task 3) moving during 6 min 4) walk up and down stairs | 0.6 ± 0.3 | N.A. |
| Compagnat et al. | 2019a | France | Cross sectional | Stroke | 35 | Lab | In: 1) Single stroke confirmed with brain imaging 2) Ability to walk continuously for 6 min without human assistance | N.R. | 64.6 ± 14.8 | Circuit (6MWT) | Comfortable (0.56 ± 0.30 m/s) | N.A. |
| Compagnat et al. | 2019b | France | Cross sectional | Stroke | 38 | Lab | In: 1) Brain imaging confirmed single stroke survivor 2) able to walk 6 min with or without mobility aids | 52.6% | 65.7 ± 13.5 | 4 tasks:  1) combination of transfers 2) manual task 3) moving during 6 min 4) walk up and down stairs | 0.52 ± 0.28 m/s | N.A. |
| Compagnat et al. | 2020 | France | Cross sectional | Stroke | 26 | Lab | In 1) Single stroke confirmed by brain imaging 2) Ability to walk continuously for 6 min with or without mobility aid | N.R. | 64.6 (55.5-77.0) | Circuit (6MWT) | 0.56 ± 0.3 | N.A. |
| Compagnat et al. | 2022 | France | Cross sectional | Stroke | 26 | Lab | In  1) Single stroke not in cerebellum  2) Ability to walk continuously for 6 min without human assistance | 61.5% | 63.5 (55.3 - 77.5) | 6MWT | 0.53 ± 0.30 m/s | N.A. |
| Costa | 2020 | Brazil | Observational study | Stroke | 55 | Lab | In: 1) At least 6 months after stroke 2) Older than 18 yrs 3) >=18 on Mini Mental State Examination 4) Walking speed at least 0.1 m/s during 10meter walk test 5) Able to walk independently without assistive device | 54.5% | 62.5 ± 14.9 | Circuit (2 MWT) | 0.7 ± 0.3 m/s | N.A. |
| Coulter et al. | 2017 | UK | Cross sectional | MS | 20 | Lab | In: 1) People with MS 2) EDSS score of 4.0-6.5 3) able to walk independently with or without aids | 45.0% | 53.7 ± 7.4 | Circuit - Sitting, walking and standing | 83.9 ± 25.1 steps/min | N.A. |
| Daligadu et al. | 2018 | Canada | Cross sectional | Post-coronay artery bypass graft surgery, aortic calve repair, mitral valve replacement | 20 | Lab | In 1) aged 35 yrs or older 2) Able to ambulate safely and independently without assistive device for at least 6 min | 90.0% | 61.3 ± 10.2 | Circuit (6 MWT) | 0.7 ± 0.2 m/s | N.A. |
| Daniel et al. | 2022 | Brazil | Cross sectional | Stroke | 24 | Lab | In  1) Single episode stroke  2) Hemiplegia  3) Gait independence with or without orthosis  ex  1) Musculoskeletal deformities  2) Pain hindering participation  3) history of CVD surgeries or interventions | 54.0% | 46.2 ± 12.0 | Treadmill walking | 0.22 - 0.89 m/s | N.A. |
| Danilack et al. | 2015 | USA | Cross sectional | COPD | 176 | Lab + Free-living | In: 1) > 40 yrs old 2) Clinical diagnosis of COPD 3) able to ambulate (with or without walking aid) | 99.0% | 72 ± 8 | Circuit (walking on level course of 244m) & 14 days free-living activities | 0.97 ± 0.22 | ≥200 steps for ≥4 days |
| de Carvalho Lana et al. | 2021 | Brazil | Cross | PD | 34 | Lab | In  1) Idiopathic PD  2) ability to walk independently  3) >50 yrs  4) levodopo medication | 76.5% | 66.8 ± 7.1 | 2MWT | Self-selected | N.A. |
| Dhillon et al. | 2018 | USA | Cross sectional | COPD, interstitial lung disease, cystic fibrosis | 8 | Lab | 1) Adults between 35 and 65 yrs old with clinically diagnosed COPD or interstitial lung disease or  2) 19 yrs and older with clinically diagnosed cystic fibrosis | N.R. | 42.1 ± 17.1 | Standardized activity protocol 1) Walking on flat 2) walking on incline 3) Rising from chair, sitting in other chair 4) Moving 1kg weight between 3 heights (shoulder, waist, floor) + Cycling 1) 10% peak 2) 25% peak 3) 50% peak 4) 60% peak | N.R. | N.A. |
| Douma et al. | 2018 | Netherlands | Cross sectional | Cancer | 72 | Free-living | Inclusion 1)  ≥18 y 2) Diagnosed advanced cancer or received adjuvant therapy for localized cancer | 63.0% | 63 ± 11.5 | 14 days free-living activities | N.R. | ≥8 hour for ≥4 days per week |
| Duclos et al. | 2019 | Canada | Cross sectional | Stroke | 20 | Lab + Free-living | In 1) At least 6 months post stroke 2) Able to walk independently and safely in community (with or without walking aids) 3) Presence of residual sensorimotor deficits at paretic lower limb | 65.0% | 53.9 ± 10.8 | 6MWT and Circuit at mall | 1.02 ± 0.41 (6MWT) 0.86 ± 0.29 (Circuit) | N.A. |
| Falter et al. | 2019 | Belgium | cross sectional | Multi (ischemic heart disease, valvular heart disease, Type 1 and Type 2 diabetes mellitus) | 40 | Lab | Patients scheduled for a CPET | 80.0% | 61.9 ± 15.2 | Cardiopulmonary exercise test, cycling ergometer | N.R. |  |
| Fanchamps et al. | 2018 | Netherlands | Cross sectional | Stroke | 25 | Lab + Free-living | Inclusion 1) aged 18-75y 3) Mobility problems cause by stroke Exclusion 3) severe mobility problems preventing safe participation (functional ambulation category score <3) | 84.0% | 56 ± 12 | Basic activities (e.g. Lying, sitting, standing, walking and cycling) Daily-life activities (e.g. Hanging laundry, packing bag, personal care activities, vacuuming, sitting and standing with upper limb activity) | N.R. | N.A. |
| Faria et al. | 2019 | Brazil | Cross sectional | Stroke | 30 | Lab | Inclusion 1) >20 y 2) stroke > 6 months 3) walk independently (with or without aids) 4) Hemiparesis | 70.0% | 62 ± 12 | Circuit (10m flat straight hallway, 5 min) | Maximum speed 1.3 ± 1.0 | N.A. |
| Farmer et al. | 2022 | Australia | Cross sectional | Orthopedic, neurological and other | 88 | Lab | In  1) admitted to rehab following hospitalization  2) able to walk 2 min with or without gait aids  3) completed 10 mWT speeds between 0.5 and 1.0 m/s | 49.0% | 73 ± 11 | Circuit indoors and outdoors | 0.81 & 0.78 m/s (in and outdoor) | N.A. |
| Farooqi et al. | 2015 | Swedem | Cross sectional | COPD | 19 | Free-living | In 1) Clinically stable COPD (GOLD stages II and III) 3) Being a woman | 0.0% | 69.2 ± 6.0 | 14 days free-living | N.R. | ≥12 days (valid days N.R.) |
| Femiano et al. | 2022 | Switzerland | Cross sectional | Cardiac rehabiliation patients (coronary infarction, hypertensive cardiomyopathy, valvular cariopathy, type a aortic dissection) | 22 | Lab | In  1) Carciac rehabilitations patients  2) Without neurological impairment  3) participated in PT sessions | N.R. | 56.6 ± 9.0 | outdoor PT session (walking, nordic walking, stairs, arm movements while walking, arm movements without walking and jogging) | N.R. | N.A. |
| Ferreira et al. | 2020 | Portugal | Cross sectional | Chronic pain | 50 | Lab | In: 1) 65 yrs or older 2) Report chronic pain associated with musculoskeletal pathology 4) Walk independently with or without assistive devices | 36.0% | 72.30 ± 6.76 | Circuit:  1) Straight path 2) Curvy path 3) Stairs | Self-selected pace & as fast as possible still walking | N.A. |
| Garcia Oliveira et al. | 2021 | Brazil | Cross sectional | Stroke | 50 | Lab | In  1) age between 20 and 80  2) diagnosed with stroke  3) walk with or without walking aids  ex  1) cognitive deficits | 64.0% | 62 (57 - 70) | 10 mWT + TUG | 0.88 (0.50 - 1.13) | N.A. |
| Gustafsson et al. | 2022 | Denmark | Cross sectional | Lumbar spinal stenosis | 30 | Lab | In  1) LSS diagnosis  2) Neurogenic claudication  3) >=60 yrs  ex  1) other diseases limiting walking  2) Impaired vision | 63.0% | 76.2 ± 7.8 | Circuit:  With and without walking aids, and intermittent | Comfortable | N.A. |
| Hei Chow et al. | 2023 | Australia | Cross sectional | Stroke | 23 | Free living | In  1) diagnosed with stroke  2) receiving in-patient rehab  3) >18 yrs  ex  1) allergies to plastic or metal | 65.0% | 74.8 ± 9.8 | 7 days of free-living | N.R. | >10 hour for ≥4 days |
| Henderson et al. | 2021 | USA | Cross sectional | Stroke | 21 | Lab | Clinical PT  1) Subacute stroke (<6 months)  2) 18-89 yrs old  3) ability to walk >50m before recent stroke  4) no significant lower extremity orthopedic impairment | 48.0% | 64.0 ± 13.5 | PT session | 0.33 (0.00 - 1.21) m/s | N.A. |
|  |  |  |  |  | 7 | Lab | Research trial  1) Chronic stroke (>6 months)  2) 18-85 yrs  3) ability to walk >10m with assistive device at speeds <1m/s  4) no medical history limiting walking capacity | 71.0% | 65.5 ± 8.3 | Exercise training practicing walking | 0.49 (0.16–1.08) m/s | N.A. |
| Herkert et al. | 2019 | Netherlands | Cross sectional | Coronary artery disease | 19 | Lab | In: 1) 18 yrs or older  2) Stable coronary artery disease with preserved left ventricular ejection fraction | 74.0% | 61.4 ± 6.9 | Circuit and treadmill: 14 low-to-moderate intensity activities - sedentary activities - household activities - treadmill walking (3 speeds) - cycling ergometer (3 loads) - stair walking (up and down) | N.R. | N.A. |
|  |  |  |  | Heart failure | 19 | Lab | In: 1) 18 yrs or older  2) Stable heart failure with reduced ejection fraction | 89.0% | 65.1 ± 6.6 | Circuit and treadmill: 14 low-to-moderate intensity activities - sedentary activities - household activities - treadmill walking (3 speeds) - cycling ergometer (3 loads) | N.R. | N.A. |
| Holubova et al. | 2022 | Czech Republic | Cross sectional | Stroke | 24 | Lab | In  1) Acute or chronic stroke  2) aged 18-70 yrs  3) gait disorder  Ex  2) trials below 2 km/h | 62.5% | 58.95 ± 12.25 | Circuit | N.R. | N.A. |
| Huber et al. | 2022 | Switzerland | Cross sectional | Stroke | 20 | Lab | In  1) >= 6 months post stroke  2) >=30 yrs  ex  1) Needing a walker  2) SR fall risk, or fall previous year  3) leg prosthesis  4) Cognitive impairment | 65.0% | 63.1 ± 12.4 | Outdoor walking | 1.34 (0.77 - 1.47) m/s | N.A. |
| Hui et al. | 2018 | Canada | RCT | Stroke | 12 | Free-living | 1) Stroke, not actively engaged in rehab 2) 18 years or older 4) able to walk 10 meter or more independently, with or without assistive device | 58.0% | 62.6 ± 9.3 | 3 days free-living activities (fri-sun) | 0.73 ± 0.27 | N.R. |
| Jao et al. | 2017 | USA | Cross sectional | Diabetes mellitus (half with foot amputation) | 31 | Lab | In 1) 18 yrs or older 2) Diabetes mellitus 3) neuropathic nonischemic diabetic foot ulcer 4) Able to engage in activities for at least 5 min without assistance from others Ex: 2) Amputation of the study foot | N.R. | 56 ± 7.5 | 14 structured activities 1 static weight-bearing 5 dynamic weight-bearing 8 non-weight-bearing | Self-selected, 60 & 100 steps/min | N.A. |
| Jayaraman et al. | 2016 | USA | Cross sectional | incomplete SCI | 8 | Lab | In:  1) Incomplete SCI 3) able to walk at least with an assistive device | 87.5% | 48.5 ± 3.7 | Activity protocol 1) sedentary; Lying down on treatment table 2) Moderate: walk 50 steps 3) high: 6 MWT | N.R. | N.A. |
| Jayaraman et al. | 2018 |  |  | Incomplete SCI | 10 | Lab | iSCI and able to ambulate with or without assistive device | 87.5% | 48.5 ± 10.4 | Circuit - Sedentary activities (Lying, sitting & standing) - Low intensity activity (50 step walk test) - High intensity activity (6MWT, multi sit to stand) | N.R. | N.A. |
| Jayaraman et al. | 2018 | USA | Cross sectional | Stroke | 8 | Lab | Stroke and able to ambulate with or without assistive device | 60.0% | 55.6 ± 9.4 | Circuit - Sedentary activities (Lying, sitting & standing) - Low intensity activity (50 step walk test) - High intensity activity (6MWT, multi sit to stand) | N.R. | N.A. |
| Jiminez-Moreno et al. | 2019 | UK | Cross sectional | Diabetes mellitus (type 1) | 30 | Lab | N.R. | 66.7% | 48 (25-72) | Circuit - 6MWT - 10mWT - 10mWRT | N.R. | N.A. |
| Juen et al. | 2015 | USA | Cross sectional | COPD, CHF, other pulmonary diseases | 28 | Lab | Patients diagnosed with pulmonary disease, such as COPD, congestive heart failure and other conditions requiring a pulmonary function test | 42.8% | N.R. | Circuit (6MWT) | N.R. | N.A. |
| Klassen et al | 2016 | Canada | Cross sectional | Stroke | 43 | Lab | In 1) At least 3 months poststroke 2) Able to walk independently for 30m with or without assistive devices | 70% | 65 ± 10.66 | Circuit 8 trials of 15m walking | Self-selected and 0.3-0.9 m/s with increases of 0.1m/s | N.A. |
| Klassen et al. | 2017 | Canada | Cross sectional | Stroke | 21 | Lab | In: 1) within 6 weeks poststroke 2) Hemiparesis 3) Ability to ambulate at least 5 m with max 1 person assisting | N.R. | 55 ± 10 | Physical therapy sessions | 0.41 ± 0.27 | N.A. |
| Ladlow et al. | 2017 | UK | Cross sectional | Amputation (lower limb) | 20 | Lab | In: 1) Traumatic amputation 2) Previously received at least 3 4week admissions of intensive exercise rehab | N.R. | 32 ± 5 (unilateral) 29 ± 4 (bilateral) | Treadmill: 5 speeds (0.48, 0.67, 0.89, 1.12, 1.34 m/s) 2 gradients at 0.98 m/s (3% and 5%) Arm crank ergometry (50, 70, 90 rmp at 55 W) | 0.48, 0.67, 0.89, 1.12, 1.34 m/s | N.A. |
| Ladlow et al. | 2019 | UK | cross sectional | Amputation (lower limb, unilateral and bilateral) | 19 | Lab | Inclusion: 1) traumatic amputation 2) previously al least 4 weeks of exercise rehab | 100.0% | 30.4 ± 4.6 | Treadmill: 5 speeds (0.48, 0.67, 0.89, 1.12, 1.34 m/s) 2 gradients at 0.98 m/s (3% and 5%) | 0.48, 0.67, 0.89, 1.12, 1.34 m/s | N.A. |
| Lai et al. | 2020 | USA | Cross sectional | Parkinson’s disease | 31 | Lab | In: 1) Neurologist-confirmed idiopathic Parkinson’s disease 2) Age between 50 and 74 yrs 3) Physically independent with bilateral symptoms (Hoehn & Yahr stage 2 or 3) confirmed by neurologist and self-reported 4) Ability to walk for 6 min (with or without assistive device) | N.R. | 64.3 ± 6.3 | Both: - 6 min over ground - 6 min treadmill | 1.05 ± 0.16 m/s | N.A. |
| Lamont et al. | 2018 | Australia | Cross sectional | Parkinsons disease | 33 | Lab | 1) confirmed idiopathic PD 2) ability to walk at least 2 min without stopping | 64.0% | 68.8 ± 8 | Circuit  - 44m indoor  - 500m outdoor with slopes, grass and stairs | Self-selected and  60, 80, 100, 120 and 140 steps/min | N.A. |
| Larkin et al. | 2016 | Ireland | Cross sectional | Rheumatoid arthritis | 20 | Lab | 1) Confirmed rheumatoid arthritis 2) 18-80 yrs old 3) Walk without assistive aid | 15.0% | 55 ± 14 | Treadmill & ADL activities | Self-selected pace (further details N.R.) | N.A. |
| Lavelle et al. | 2021 | UK | Cross sectional | MS | 19 | Lab | In  1) Self-Reported MS  2) >= 18 yrs  3) Relapse free >3 months  4) able to walk independently with or without aids  ex  1) Pregnant | 31.6% | 52.1 ± 11.9 | Circuit:  Deskwork, elevator, washing and drying dishes, indoor walking, walking with obstacles, outdoor walking, stationary cycling, driving | N.R. | N.A. |
| Mahendran et al. | 2016 | Australia | Cross sectional | Stroke | 15 | Lab | In: 1) Unilateral stroke at least 6 months prior 2) Independently mobile 3) Community-dwelling | 53.3% | 63.4 ± 8.3 | 6 MWT, treadmill task, 200 m outdoor circuit | Slow (0.31 ± 0.11 m/s), comfortable (0.42 ± 0.17) and fast (0.54 ± 0.25) | N.A. |
| Mandigout et al. | 2017 | France | Cross sectional | Stroke | 24 | Lab | In: 1) 18 yrs or older 2) Able to perform transfer and displacement on their own 3) Ischemic or hemorrhagic stroke within 6 months | 62.5% | 68.2 ± 13.9 | Scenario of everyday tasks (transfers, walking, up and down stairs & tidying | N.R. | N.A. |
| McGinley et al. | 2015 | Canada | Cross sectional | Diabetes mellitus (type 2) | 35 | Free-living | 1) Physician diagnosed type 2 diabetes 2) At least 35 yrs old 3) Walk without impairment on treadmill at moderate pace for 20 min | 60.0% | 62.8 ± 7.8 | 14 days free-living activities, 3x/wk 30 min walking or jogging | N.R. | N.R. |
| Miyamoto et al. | 2018 | Japan | Cross sectional | COPD | 11 | Free-living | Inclusion 1) Stable COPD 2) aged 40 or older | 91.6% | 76.6 ± 6.9 | Free-living, at least 8 hours available | N.R. | ≥8 hour for ≥3 days |
| Negrini et al. | 2020 | Italy | Cross sectional | Stroke | 43 | Lab | In: 1) Hemiparetic post-stroke 2) ability to perform timed up an go without assistance of operator | 62.8% | 61.3 ± 14.95 | Circuit: 1) 10m walking test 2) 50m walking test 3) 6 MWT 4) Timed up and go test | 0.75 ± 0.32 m/s | N.A. |
| Nishida et al. | 2020 | Japan | Cross sectional | Diabetes mellitus (type 2) | 51 | Free-living | In: 1) Outpatient with T2DM 2) Aged 6--79 yrs | 45.1% | 70 ± 5 | 12 to 16 days free-living activities | N.R. | ≥10 hour (valid case N.R.) |
| O'Brien et al. | 2020 | UK | Cross sectional | Rheumatoid arthritis | 22 | Lab | In: 1) Clinical diagnosis of RA 2) 18 yrs or older 3) ambulate independently | 14.0% | 53.7 ± 12.5 | Circuit 11 activities: -6 standardized -5 ADLs | N.R. | N.A. |
| O'Brien et al. | 2020 |  |  | Rheumatoid arthritis | 100 | Free-living | In: 1) Clinical diagnosis of RA 2) 18 yrs or older 3) ambulate independently, with or without assistive device | 29.0% | 58.5 ± 12.1 | 7 days free-living activities | N.R. | ≥10 hour for ≥4 days (incl ≥1 weekend day) |
| O'Neill et al. | 2017 | UK | Cross sectional | Bronchiectasis | 55 | Free-living | In: 1) 18 yrs or older 2) Diagnosis of bronchiectasis confirmed by HRCT or CT | 40.0% | 60 ± 10 | 7 days of free-living activities | N.R. | Between 100 – 50.000 steps for ≥5 days (incl ≥1 weekend day) |
| Pham et al. | 2017 | Germany | Prospective observational study | Parkinsons disease | 20 | Lab | In: 1) Professionally diagnosed Parkinson’s disease Ex: 1) Deep brain stimulation 2) Hoehn and Yahr score >3 3) Mini Mental Stat Examination score <24 | 52.4% | 66.4 ± 9.0 | Circuit  daily-life activities i.e moving around labs and corridors, walking backwards, climbing stairs, performing transfers, making coffee, brushing teeth and ironing clothes | N.R. | N.A. |
| Polese et al. | 2019 | Brazil | Cross sectional | Stroke | 37 | Lab | In: 1) Stroke, at least 6 months since onset 2) 20 yrs or older 3) Able to walk independently with or without assistive devices | 75.7% | 62 ± 11 | Circuit: 10 m flat and straight hallway, 5 minutes at maximus speed | 0.9 ± 0.3 Comfortable 1.3 ± 0.6 Fast | N.A. |
| Polhemus et al. | 2023 | Switzerland | Cross sectional | MS | 45 | Lab + FL | In  1) confirmed MS diagnosis  2) >= 18 yrs  3) reduced, but independent, walking ability with or without assistive device | 35.6% | 46 (IQR 40 - 51) | 6MWT  Circuit:  - Walking with postural transitions  - Simulated cleaning  - Sit to stand  - Wheelchair push  - Stair climb and descent  Free living 14 days | 109 (61-146) steps/min | ≥10 hour for ≥2 days |
| Popp et al. | 2019 | Switzerland | Cross sectional | Incomplet SCI | 30 | Lab | In: 1) >18 yrs 2) >1 yrs post injury 3) Able to walk at least 100m without supervision | 70.0% | 54.1 ± 11.9 | Circuit (34 activities of daily living divided in the following categories) - Rest - Leisure time - Housework - Office work - Sport and fitness - Locomotion | N.R. | N.A. |
| Prieto-Centurion et al. | 2016 | USA | Cross sectional | COPD | 4 | Home situation | 1) Physician diagnosed COPD exacerbation 2) Walk unaided | 100.0% | 69 ± 10 | 6MWT | N.R. | N.A. |
| Roberts-Lewis et al. | 2022 | UK | longitudinal | Progressive muscle diseases (body myositis, myotonic dystrophy, or muscular dystrophy) | 20 | Lab | In  1) Confirmed diagnosis of inclusion body myositis, myotonic dystrophy, or muscular dystrophy  Ex  1) Cognitively impaired  2) aged <18yrs  3) muscle weakness from other nervous system dysfunction | N.R. | N.R. | Circuit (Sitting, Lying, walking, cycling) | N.R. | N.A. |
|  |  |  |  |  | 56 | Free living | See above | 44.6% | 44.7 ± 15.1 | 7 days of free living | N.R. | ≥23 hour (valid case N.R.) |
| Rockette-Wagner et al. | 2021 | USA | Longitudinal | Inflammatory myopathy | 50 | Free-living | In  1) Patients with inflammatory myopathy  ex  1) other conditions interfering with physical functioning | 40.0% | 48.6 ± 15.4 | 7 days of free-living | N.R. | ≥10 hour for ≥4 days |
| Rossi et al. | 2018 | USA | Cross sectional | Endometrial cancer | 25 | Free-living | 1) Adult (ages 18-90 years) 2) Endometrial cancer survivor 3) No diagnosed signs of disease within 6 months | 0.0% | 62 ± 9 | 30 days free-living activities | N.R. | N.R. |
| Salih et al. | 2016 | Australia | Cross sectional | Amputation (lower limb) | 21 | Lab | 1) Unilateral lower limb amputee patients 2) Able to walk with or without walking aids, using prosthesis | 71.4% | 59.4 ± 11.5 | Activity routine: 1) 5 min walking 2) 3 min self-propelling wheelchair 3) 3 min being pushed in wheelchair | N.R. | N.A. |
| Saygin et al. | 2022 | USA | Longitudinal | Myositis | 24 | Free living | In  1) patients with myositis | 29.2% | 46.2 ± 14.4 | 7 days of free living | N.R. | ≥500 steps for ≥3  days |
| Schaffer et al. | 2017 | USA | Cross sectional | Stroke | 24 | Lab | 1) Stroke >6 months 2) 18 yrs or older 4) Able to walk with no more than minimal assistance (with or without assistive devices) | 58.3% | 54 ± 13.4 | validity: 6 MWT Reliability 50 step test | 0.72 ± 0.40 | N.A. |
| Semanik et al. | 2020 | USA | RCT (part of) | Chronic knee symptoms | 35 | Free-living | In: 2) 18 yrs or older 3) Have chronic knee symptoms 4) Able to ambulate at least 15.24 m Ex: 2) Total joint replacement occurred or planning within year | 31.0% | 52 ± N.R. | 7 days free-living activities | N.R. | ≥10 hour (valid case N.R.) |
| Shimizu et al. | 2018 | Japan | Cross sectional | Stroke | 10 | Lab | 1) First stroke 2) One month after stroke 4) able to walk independently with or without aids | 50.0% | 57.5 ± 16.2 | circuit: Sitting, lying, standing, sitting with reaching, standing with reaching | 0.98 ± 0.36 m/s | N.A. |
| Shoemaker et al. | 2017 | USA | Cohort | Heart failure | 16 | Free-living | In: 1) 40 yrs or older 2) Diagnosis of HF 3) implementation of a Medtronic ICD or CRT device at least 6 months before study enrollment 4) NYHA-FC II-III symptoms | 56.3% | 64.9 ± 11.3 | 7 days free-living activities | N.R. | N.R. |
| Smith et al. | 2019 | USA | Cross sectional | Amputation (lower limb) | 32 | Lab | In: 1) Transtibial to hip disarticulation prosthesis wearers on one or both sides 2) Able to walk without assistive devices | 66.0% | 49.7 ± 14.0 | Circuit: 140m flat indoor path at self-selected speed | Self-selected (no details reported) | N.A. |
| Smith & Guerra | 2021 | USA | Cross sectional | Amputation (lower limb) | 35 | lab | In  1) amputation at tibial or femoral level on one or both sides  Ex  1) relied on assistive walking devices | 54.3% | 48.5 ± 14.8 | 2MWT | 1.23 ± 0.22 m/s | N.A. |
| Stuart et al. | 2020 | UK | Longitudinal | MS | 56 | Free-living | In 1) diagnosis of progressive MS 2) aged =< 70 yrs 3) EDSS =< 6.5 Ex 1) relapse in last year 2) disease-modifying or immunosuppressive treatment last 6 months 3) comorbidities contributing to neurological disability | 52.0% | 53.6 ± 8.0 | 2-7 days of free-living | N.R. | ≥10 hour for ≥3 days |
| Takasaki | 2017 | Japan | Cross sectional | Lower back pain | 15 | Free-living | In: 1) age between 20 and 60 yrs 2) Persistent lower back pain for more than 3 months | 40.0% | 22.1 ± 4.3 | 14 days free-living activities | N.R. | N.A. |
| Taoum et al. | 2020 | France | Cross sectional | peripheral artery disease | 23 | LAB | In  1) Having Peripheral artery disease | N.R. | 60 ± 10 | Outdoor walking 45 - 60 min | Self-selected | N.A. |
| Thorup et al. | 2017 | Denmark | Cross sectional | Heart disease (acute coronary syndrome, heart failure, coronary artery bypass grafting or valve surgery) | 24 | Free-living | In: 1) 18 yrs or older 2) hospitalized with acute coronary syndrome, heart failure, coronary artery bypass grafting or valve surgery | 91.6% | 67.0 ± 10.0 | Day at hospital and day at home & known period of 3min walking for both | N.R. | N.A. |
| Treacy et al. | 2017 | Australia | Cross sectional | Multi (Fractured hip, pelvis, lower limb orthopedic surgery, stroke, TIA, neurological event, decreased mobility post medical or non-orthopedic surgical event, post fall with no lower limb fracture, other) | 166 | Lab | in: 1) Gait speed <1.2 m/s or gait abnormality likely impacting accuracy of AM 2) able to perform 6MWT | 55.0% | 80 ± 11 | 6MWT | 0.42 ± 0.22 m/s | N.A. |
| Ummels et al. | 2018 | Netherlands | Cross sectional | Multi (CVD, cancer, COPD, osteoarthritis, chronic pain) | 130 | Lab | In: 1) 18 years or older 2) Diagnosed with at least 1 of the following chronic diseases (CVD, COPD, DM, Chronic pain, cancer, osteoarthritis) Ex: 2) Use of walking aid | 43.6% | 61.5 ± 11.1 | Circuit (activity protocol representing activities of daily living) | 1.3 ± 0.3 | N.A. |
| Van Blarigan et al. | 2017 | USA | Cross sectional | prostate cancer | 22 | Free-living | 1) Diagnosed with adenocarcinoma of prostate | 100.0% | 66 (56-83) | 7 days of free-living | N.R. | N.A. |
| Van der Weegen et al. | 2015 | Netherlands | Cross sectional | Multi (COPD (N=4), type II diabetes (N=5)) | 13 | Lab | 1) COPD or T2 Diabetes 2) 40 yrs or older 3) able to walk independently | N.R. | 60.9 ± 7.1 (Lab) | Treadmill | 0.56 + 0.14 every 3 min | N.A. |
|  |  |  |  | Multi (COPD (N=5), type II diabetes (N=7)) | 13 | Free-living |  | N.R. | 61.6 ± 9.2 (free-living) | 6-7 consecutive days free-living activities | N.R. | N.R. |
| Van Laerhoven et al. | 2022 | Germany | Cross sectional | Diabetes | 28 | Free living | In  1) diabetes or CVD | 65.0% | 74.8 ± 9.8 | 2 days of free-living | N.R. | No limitations |
| Vetrovsky et al | 2019 | Australia | Cross sectional | Heart failure | 15 | Free-living | In: 1) Diagnosis of heart failure (NYHA class II or III) | 60.0% | 65.5 ± 12.6 | 3 days free-living activities | N.R. | No limitations |
| Wagner et al. | 2022 | Denmark | Cross sectional | Rheumatoid arthritis | 30 | Lab | In  1) Diagnosis of RA  2) >= 18 yrs | 17.0% | 61 (50-74) | Treadmill | 2.5 – 5 km/h, increments of 0.5 km/h | N.A. |
| Webber & John | 2016 | Canada | Cross sectional | Multi (respiratory/infection, orthopedic, falls/decreased mobility, neurological, gastrointestinal, cancer, endocrine) | 38 | Lab | In: 1) 65 yrs or older 2) Ambulating without assistance of others 3) ≥25/30 MMSE | 10.5% | 83.2 ± 7.1 | Hallway walk | 0.4 ± 0.2 | N.A. |
| Webster et al. | 2021 | USA | Cross sectional | COPD | 59 | Free living | In  1) >= 50 yrs  2) stable COPD (moderate to severe)  Ex  1) participating in pulmonary rehab previous year  2) major health problems limiting PA | 52.5% | 69.4 ± 7.8 | 7 days of free living | N.R. | ≥10 hour for ≥4 days |
| Wendel et al. | 2018 | USA | Cross sectional | Parkinson’s disease | 33 | Lab | 1) Idiopathic PD 2) ≥22 on Montreal Cognitive Assessment 3) ability to walk pain free 10 min independently without aids | 58.0% | 65.5 ± 9.4 | Circuit: Continuous   - 2 MWT Discontinuous  - Simulated home environment   - negotiating obstacles | Comfortable and fast pace | N.A. |
| Zbogar | 2016 | Canada | Cohort | SCI | 35 | Lab (in clinic) | In: 1) Nontraumatic SCI | 70.0% | 48.9 ± 18.3 | 1 day of rehabilitation | 0.75 ± 0.39 | N.A. |
| Zhai et al. | 2020 | Germany | Cross sectional | MS | 67 | Free-living | In: 1) Age 18-65 2) confirmed diagnosis of MS 3) Extended Disability Status Scale score below 6.5 | 37.3% | 42.9 ± 10.9 | 7 days free-living activities | N.R. | N.R. |
| Yu et al. | 2022 | Australia | Longitudinal | Osteoarthritis | 65 | Free living | In  1) aged >= 50 yrs  2) knee pain most days last months  3) medial tibiofemoral Kellgren and Lawrence grade 2-3 | 54.0% | 61.3 ± 5.99 | Free living 7 days | N.R. | N.R. |

RCT = randomized controlled trial

CAD = coronary artery disease, COPD = chronic obstructive pulmonary disease, CVD = cardiovascular disease, DM = diabetes mellitus, DM1 = diabetes mellitus type 1, DM2 = diabetes mellitus type 2, iSCI = incomplete spinal cord injury, MS = multiple sclerosis, PAD = pulmonary artery disease, PD = Parkinson’s disease, SCI = spinal cord injury

Both = both free-living and laboratory with protocolled activities, FL = Free-living, Home = home situation with protocolled activities, Lab = laboratory setting with protocolled activities

Yrs = years

MWT = minutes walking test

N.R. = Not reported, N.A. = Not applicable
