# Supplementary material for: Measurement properties of device-based physical activity instruments in ambulatory adults with physical disabilities and/or chronic diseases: a scoping review
Source: BMC Sports Sci Med Rehabil. 2023 Sep 21;15:115. doi: 10.1186/s13102-023-00717-0 (PMC10512652; doi:10.1186/s13102-023-00717-0)
Supplement: Supplementary file 5 — Additional file 5: Supplementary file 5. Expanded overview of research-grade devices evaluated on their measurement properties in 52 studies. An expanded overview of the research-grade devices evaluated on their measurement properties. Extra information on epoch length, sampling rate and results per condition. [file 13102_2023_717_MOESM5_ESM.docx]

# Supplementary file 5 – Expanded overview of research-grade devices evaluated on their measurement properties in 52 studies. Ordering on number of studies evaluating manufacturer.

|  | |  |  | |  | | |  | |  | |  |  | |  | | |  | |  | | Result |  |  |  |
| --- | --- | --- | --- | --- | --- | --- | --- | --- | --- | --- | --- | --- | --- | --- | --- | --- | --- | --- | --- | --- | --- | --- | --- | --- | --- |
| Type | | Device type | PA outcome | | population | | | Study | | Measurement property | | Criterion | Placement | | Algorithm | | | Epoch length | | Sampling rate | | Test | Outcome |  |  |
| **ActiGraph** | | | | | | | | | | | | | | | | | | | | | | | | |  |
| GT3 | | Accelerometer | EE | | Amputation | | | Ladlow et al. (2017) | | CV | | IC | Waist (SRL) | | Cust | | | N.R. | | 30 Hz | | Pearson’s r | Unilateral: r = 0.86 |  |  |
|  |  |  |  |  |  |  |  |  |  |  |  |  |  |  |  |  |  |  |  |  |  |  | Bilateral: r = 0.94 |  |  |
|  | |  |  | |  | | | Ladlow et al. (2019) | | CV | | IC | Waist (SRL) | | Cust | | | N.R. | | 30 Hz | | Pearson’s r | Unilateral: r = 0.92 |  |  |
|  |  |  |  |  |  |  |  |  |  |  |  |  |  |  |  |  |  |  |  |  |  |  | Bilateral: r = 0.96 |  |  |
|  | |  |  | | iSCI | | | Jayaraman et al (2016) | | CV | | IC | Ankle | | Prop (Freedson + Harris Benedict BMR) | | | 10 sec | | N.R. | | ANOVA (one way) | Sed: p<.05 |  |  |
|  |  |  |  |  |  |  |  |  |  |  |  |  |  |  |  |  |  |  |  |  |  |  | Low: p>.05 |  |  |
|  |  |  |  |  |  |  |  |  |  |  |  |  |  |  |  |  |  |  |  |  |  |  | High: p>.05 |  |  |
|  | |  |  | |  | | |  | |  | |  | U-arm | | Prop (Freedson + Harris Benedict BMR) | | | 10 sec | | N.R. | | ANOVA (one way) | Sed: p<.05 |  |  |
|  |  |  |  |  |  |  |  |  |  |  |  |  |  |  |  |  |  |  |  |  |  |  | Low: p<.05 |  |  |
|  |  |  |  |  |  |  |  |  |  |  |  |  |  |  |  |  |  |  |  |  |  |  | High: p>.05 |  |  |
|  | |  |  | |  | | |  | |  | |  | Waist | | Prop (Freedson + Harris Benedict BMR) | | | 10 sec | | N.R. | | ANOVA (one way) | Sed: p<.05 |  |  |
|  |  |  |  |  |  |  |  |  |  |  |  |  |  |  |  |  |  |  |  |  |  |  | Low: p<.05 |  |  |
|  |  |  |  |  |  |  |  |  |  |  |  |  |  |  |  |  |  |  |  |  |  |  | High: p>.05 |  |  |
|  | |  |  | |  | | | Jayaraman et al (2018) | | CV | | IC | Ankle | | Prop (Freedson + Harris Benedict BMR | | | N.R. | | 30 Hz | | Kruskal wallis (Games-Howell post hoc) | Lying: mean diff: 1.58, p < .001 |  |  |
|  |  |  |  |  |  |  |  |  |  |  |  |  |  |  |  |  |  |  |  |  |  |  | Sitting: mean diff = 1.47, p < .001 |  |  |
|  |  |  |  |  |  |  |  |  |  |  |  |  |  |  |  |  |  |  |  |  |  |  | Standing: mean diff = 1.73, p < .001 |  |  |
|  |  |  |  |  |  |  |  |  |  |  |  |  |  |  |  |  |  |  |  |  |  |  | 50 SWT: mean diff = 0.81, p = .942 |  |  |
|  |  |  |  |  |  |  |  |  |  |  |  |  |  |  |  |  |  |  |  |  |  |  | 6 MWT: mean diff = 2.88, p = .064 |  |  |
|  |  |  |  |  |  |  |  |  |  |  |  |  |  |  |  |  |  |  |  |  |  |  | Sit-to-stand: mean diff = 3.77, p < .001 |  |  |
|  | |  |  | |  | | |  | |  | |  | U arm | | Prop (Freedson + Harris Benedict BMR | | | N.R. | | 30 Hz | | Kruskal wallis (Games-Howell post hoc) | Lying: mean diff: 1.58, p < .001 |  |  |
|  |  |  |  |  |  |  |  |  |  |  |  |  |  |  |  |  |  |  |  |  |  |  | Sitting: mean diff = 1.43, p < .001 |  |  |
|  |  |  |  |  |  |  |  |  |  |  |  |  |  |  |  |  |  |  |  |  |  |  | Standing: mean diff = 1.71, p < .001 |  |  |
|  |  |  |  |  |  |  |  |  |  |  |  |  |  |  |  |  |  |  |  |  |  |  | 50 SWT: mean diff = 2.25, p = .007 |  |  |
|  |  |  |  |  |  |  |  |  |  |  |  |  |  |  |  |  |  |  |  |  |  |  | 6 MWT: mean diff = 2.87, p = .067 |  |  |
|  |  |  |  |  |  |  |  |  |  |  |  |  |  |  |  |  |  |  |  |  |  |  | Sit-to-stand: mean diff = -0.82, p = .912 |  |  |
|  | |  |  | |  | | |  | |  | |  | Waist | | Prop (Freedson + Harris Benedict BMR | | | N.R. | | 30 Hz | | Kruskal wallis (Games-Howell post hoc) | Lying: mean diff: 1.58, p < .001 |  |  |
|  |  |  |  |  |  |  |  |  |  |  |  |  |  |  |  |  |  |  |  |  |  |  | Sitting: mean diff = 1.47, p < .001 |  |  |
|  |  |  |  |  |  |  |  |  |  |  |  |  |  |  |  |  |  |  |  |  |  |  | Standing: mean diff = 1.73, p < .001 |  |  |
|  |  |  |  |  |  |  |  |  |  |  |  |  |  |  |  |  |  |  |  |  |  |  | 50 SWT: mean diff = 2.88, p = .001 |  |  |
|  |  |  |  |  |  |  |  |  |  |  |  |  |  |  |  |  |  |  |  |  |  |  | 6 MWT: mean diff = 4.15, p = .002 |  |  |
|  |  |  |  |  |  |  |  |  |  |  |  |  |  |  |  |  |  |  |  |  |  |  | Sit-to-stand: mean diff = 2.29, p = .002 |  |  |
|  | |  |  | | Stroke | | | Compagnat et al. (2018) | | CV | | IC | Ankle (h) | | N.R. | | | 1 sec | | 10 Hz | | Pearson’s r | r = 0.41 |  |  |
|  | |  |  | |  | | |  | |  | |  | Waist | | N.R. | | | 1 sec | | 10 Hz | | Pearson’s r | r = 0.15 |  |  |
|  | |  |  | |  | | |  | |  | |  | Wrist (h) | | N.R. | | | 1 sec | | 10 Hz | | Pearson’s r | r = 0.12 |  |  |
|  | |  |  | |  | | | Compagnat et al. (2020) | | CV | | IC | Waist | | Prop | | | N.R. | | N.R. | | Pearson’s r | r = 0.19 |  |  |
|  | |  |  | |  | | |  | |  | |  |  | | Cust | | | N.R. | | N.R. | | Pearson’s r | r = 0.44 |  |  |
|  | |  |  | |  | | | Compagnat et al. (2022) | | CV | | IC | Ankle (ua) | | TEE = distance * Energy cost walking | | | 1 sec | | 30 Hz | | Bland-Altman LoA | Diff. equations for energy cost of walking: |  |  |
|  |  |  |  |  |  |  |  |  |  |  |  |  |  |  |  |  |  |  |  |  |  |  | Compagnat: 1.2 [-12.0; 14.3] |  |  |
|  |  |  |  |  |  |  |  |  |  |  |  |  |  |  |  |  |  |  |  |  |  |  | Polese: 3.5 [-9.2; 16.1] |  |  |
|  |  |  |  |  |  |  |  |  |  |  |  |  |  |  |  |  |  |  |  |  |  |  | Zamparo: 8.3 [-1.1; 17.8] |  |  |
|  |  |  |  |  |  |  |  |  |  |  |  |  |  |  |  |  |  |  |  |  |  |  | Tesio: -69.1 [-148.7; 10.5] |  |  |
|  |  |  |  |  |  |  |  |  |  |  |  |  |  |  |  |  |  |  |  |  |  |  | Prop: -15.0 [-52.9; 22.8] |  |  |
|  | |  |  | |  | | | Faria et al. (2018) | | CV | | IC | Ankle (a) | | Prop (work-energy teorem) | | | 60 sec | | 30 Hz | | Pearson’s r | r = 0.04 |  |  |
|  | |  |  | |  | | |  | |  | |  |  | | Prop (Freedson) | | | 60 sec | | 30 Hz | | Pearson’s r | r = 0.04 |  |  |
|  | |  |  | |  | | |  | |  | |  |  | | Prop (combined) | | | 60 sec | | 30 Hz | | Pearson’s r | r = 0.37 |  |  |
|  | |  |  | |  | | | Jayaraman et al (2018) | | CV | | IC | Ankle (a) | | Prop (Freedson + Harris Benedict BMR | | | N.R. | | 30 Hz | | Kruskal wallis (Games-Howell post hoc) | Lying: mean diff = 1.65, p = .016 |  |  |
|  |  |  |  |  |  |  |  |  |  |  |  |  |  |  |  |  |  |  |  |  |  |  | Sitting: mean diff = 1.19, p = .002 |  |  |
|  |  |  |  |  |  |  |  |  |  |  |  |  |  |  |  |  |  |  |  |  |  |  | Standing: mean diff = 1.42, p = .020 |  |  |
|  |  |  |  |  |  |  |  |  |  |  |  |  |  |  |  |  |  |  |  |  |  |  | 50 SWT: mean diff = -0.23, p = .999 |  |  |
|  |  |  |  |  |  |  |  |  |  |  |  |  |  |  |  |  |  |  |  |  |  |  | 6 MWT: mean diff = -0.49, p = .989 |  |  |
|  |  |  |  |  |  |  |  |  |  |  |  |  |  |  |  |  |  |  |  |  |  |  | Sit-to-stand: mean diff = 4.54, p = .004 |  |  |
|  | |  |  | |  | | |  | |  | |  | Ankle (ua) | | Prop (Freedson + Harris Benedict BMR | | | N.R. | | 30 Hz | | Kruskal wallis (Games-Howell post hoc) | Lying: mean diff = 1.57, p = .001 |  |  |
|  |  |  |  |  |  |  |  |  |  |  |  |  |  |  |  |  |  |  |  |  |  |  | Sitting: mean diff = 1.28, p < .001 |  |  |
|  |  |  |  |  |  |  |  |  |  |  |  |  |  |  |  |  |  |  |  |  |  |  | Standing: mean diff = 1.53, p = .001 |  |  |
|  |  |  |  |  |  |  |  |  |  |  |  |  |  |  |  |  |  |  |  |  |  |  | 50 SWT: mean diff = -2.96, p = .110 |  |  |
|  |  |  |  |  |  |  |  |  |  |  |  |  |  |  |  |  |  |  |  |  |  |  | 6 MWT: mean diff = -3.72, p = .528 |  |  |
|  |  |  |  |  |  |  |  |  |  |  |  |  |  |  |  |  |  |  |  |  |  |  | Sit-to-stand: mean diff = 4.84, p = .015 |  |  |
|  | |  |  | |  | | |  | |  | |  | U arm (a) | | Prop (Freedson + Harris Benedict BMR | | | N.R. | | 30 Hz | | Kruskal wallis (Games-Howell post hoc) | Lying: mean diff = 1.65, p = .016 |  |  |
|  |  |  |  |  |  |  |  |  |  |  |  |  |  |  |  |  |  |  |  |  |  |  | Sitting: mean diff = 1.19, p = .002 |  |  |
|  |  |  |  |  |  |  |  |  |  |  |  |  |  |  |  |  |  |  |  |  |  |  | Standing: mean diff = 1.42, p = .020 |  |  |
|  |  |  |  |  |  |  |  |  |  |  |  |  |  |  |  |  |  |  |  |  |  |  | 50 SWT: mean diff = -0.08, p = 1.00 |  |  |
|  |  |  |  |  |  |  |  |  |  |  |  |  |  |  |  |  |  |  |  |  |  |  | 6 MWT: mean diff = -0.69, p = .964 |  |  |
|  |  |  |  |  |  |  |  |  |  |  |  |  |  |  |  |  |  |  |  |  |  |  | Sit-to-stand: mean diff = 1.86, p = .348 |  |  |
|  | |  |  | |  | | |  | |  | |  | U arm (ua) | | Prop (Freedson + Harris Benedict BMR | | | N.R. | | 30 Hz | | Kruskal wallis (Games-Howell post hoc) | Lying: mean diff = 1.58, p = .001 |  |  |
|  |  |  |  |  |  |  |  |  |  |  |  |  |  |  |  |  |  |  |  |  |  |  | Sitting: mean diff = 1.25, p < .001 |  |  |
|  |  |  |  |  |  |  |  |  |  |  |  |  |  |  |  |  |  |  |  |  |  |  | Standing: mean diff = 1.39, p = .001 |  |  |
|  |  |  |  |  |  |  |  |  |  |  |  |  |  |  |  |  |  |  |  |  |  |  | 50 SWT: mean diff = 0.14, p =1.000 |  |  |
|  |  |  |  |  |  |  |  |  |  |  |  |  |  |  |  |  |  |  |  |  |  |  | 6 MWT: mean diff = -0.30, p = 1.000 |  |  |
|  |  |  |  |  |  |  |  |  |  |  |  |  |  |  |  |  |  |  |  |  |  |  | Sit-to-stand: mean diff = -1.61, p =.842 |  |  |
|  | |  |  | |  | | |  | |  | |  | Waist (a) | | Prop (Freedson + Harris Benedict BMR | | | N.R. | | 30 Hz | | Kruskal wallis (Games-Howell post hoc) | Lying: mean diff = 1.65, p = .016 |  |  |
|  |  |  |  |  |  |  |  |  |  |  |  |  |  |  |  |  |  |  |  |  |  |  | Sitting: mean diff = 1.19, p = .002 |  |  |
|  |  |  |  |  |  |  |  |  |  |  |  |  |  |  |  |  |  |  |  |  |  |  | Standing: mean diff = 1.42, p = .020 |  |  |
|  |  |  |  |  |  |  |  |  |  |  |  |  |  |  |  |  |  |  |  |  |  |  | 50 SWT: mean diff = 0.74, p = .543 |  |  |
|  |  |  |  |  |  |  |  |  |  |  |  |  |  |  |  |  |  |  |  |  |  |  | 6 MWT: mean diff = 0.17, p = 1.000 |  |  |
|  |  |  |  |  |  |  |  |  |  |  |  |  |  |  |  |  |  |  |  |  |  |  | Sit-to-stand: mean diff = -0.61, p = .966 |  |  |
|  | |  |  | |  | | |  | |  | |  | Waist (ua) | | Prop (Freedson + Harris Benedict BMR | | | N.R. | | 30 Hz | | Kruskal wallis (Games-Howell post hoc) | Lying: mean diff = 1.427, p < .001 |  |  |
|  |  |  |  |  |  |  |  |  |  |  |  |  |  |  |  |  |  |  |  |  |  |  | Sitting: mean diff = 1.25, p < .001 |  |  |
|  |  |  |  |  |  |  |  |  |  |  |  |  |  |  |  |  |  |  |  |  |  |  | Standing: mean diff = 1.54, p = .001 |  |  |
|  |  |  |  |  |  |  |  |  |  |  |  |  |  |  |  |  |  |  |  |  |  |  | 50 SWT: mean diff = 1.11, p = .763 |  |  |
|  |  |  |  |  |  |  |  |  |  |  |  |  |  |  |  |  |  |  |  |  |  |  | 6 MWT: mean diff = 1.12, p = .993 |  |  |
|  |  |  |  |  |  |  |  |  |  |  |  |  |  |  |  |  |  |  |  |  |  |  | Sit-to-stand: mean diff = -0.50, p = .998 |  |  |
|  | |  |  | |  | | | Mandigout et al. (2017) | | CV | | IC | Ankle (h) | | N.R. | | | N.R. | | N.R. | | Spearman’s rho | r = 0.19 |  |  |
|  | |  |  | |  | | |  | |  | |  | Ankle (a) | | N.R. | | | N.R. | | N.R. | | Spearman’s rho | r = 0.21 |  |  |
|  | |  |  | |  | | |  | |  | |  | Waist | | N.R. | | | N.R. | | N.R. | | Spearman’s rho | r = 0.04 |  |  |
|  | |  |  | |  | | |  | |  | |  | Wrist (h) | | N.R. | | | N.R. | | N.R. | | Spearman’s rho | r = 0.20 |  |  |
|  | |  |  | |  | | |  | |  | |  | Wrist (a) | | N.R. | | | N.R. | | N.R. | | Spearman’s rho | r = 0.08 |  |  |
|  | |  | Steps | | Diabetes mellitus | | | Jao et al. (2017) | | CV | | DO | Waist | | Prop | | | 15 sec | | N.R. | | Accuracy (%) | Self-paced: 79.0 ± 22.2% |  |  |
|  |  |  |  |  |  |  |  |  |  |  |  |  |  |  |  |  |  |  |  |  |  |  | 60 steps/min: 43.0 ± 26.2% |  |  |
|  |  |  |  |  |  |  |  |  |  |  |  |  |  |  |  |  |  |  |  |  |  |  | 100 steps/min: 81.4 ± 21.4% |  |  |
|  |  |  |  |  |  |  |  |  |  |  |  |  |  |  |  |  |  |  |  |  |  |  | Stair self-paced: 69.0 ± 15.1% |  |  |
|  |  |  |  |  |  |  |  |  |  |  |  |  |  |  |  |  |  |  |  |  |  |  | Stair 60 steps/min: 70.3 ± 15.4% |  |  |
|  | |  |  | | Inflammatory myopathy | | | Rockette-Wagner et al. (2021) | | Con V, conv | | Functional test, TUG | Waist | | Prop | | | 1 sec | | N.R. | | Pearson's r | r = -0.42 (-0.64 - -0.12) |  |  |
|  | |  |  | |  | | |  | |  | | Functional test, 30STS | Waist | | Prop | | | 2 sec | | N.R. | | Pearson's r | r = 0.52 (0.26 - 0.70) |  |  |
|  | |  |  | |  | | |  | |  | | Test, 6MWT | Waist | | Prop | | | 3 sec | | N.R. | | Pearson's r | r = 0.66 (0.45 - 0.80) |  |  |
|  | |  |  | |  | | |  | |  | | Functional SR - SF36 PF | Waist | | Prop | | | 4 sec | | N.R. | | Pearson's r | r = 0.54 (0.27 - 0.72) |  |  |
|  | |  |  | |  | | |  | |  | | Funcitonal SR - PROMIS PF-20 | Waist | | Prop | | | 5 sec | | N.R. | | Pearson's r | r = 0.57 (0.32 - 0.73) |  |  |
|  | |  |  | |  | | |  | | Con V, div | | Funcitonal - Cutaneous disease activity | Waist | | Prop | | | 6 sec | | N.R. | | Pearson's r | r = -0.07 (-0.35 - 0.23) |  |  |
|  | |  |  | |  | | |  | |  | | Funcitonal - Pulmonary disease activity | Waist | | Prop | | | 7 sec | | N.R. | | Pearson's r | r = -0.09 (-0.37 - 0.21) |  |  |
|  | |  |  | |  | | |  | |  | | Functional - Extramuscular disease activity | Waist | | Prop | | | 8 sec | | N.R. | | Pearson's r | r = -0.24 (-0.49 - 0.06) |  |  |
|  | |  |  | |  | | |  | | TRT R | |  | Waist | | Prop | | | 1 sec | | N.R. | | ICC | ICC = 0.92 (CI 0.86 – 0.96) |  |  |
|  | |  |  | |  | | |  | | Resp | | SR | Waist | | Prop | | | 1 sec | | N.R. | | Pearson's r | r = 0.47 (CI 0.12 - 0.71) |  |  |
|  | |  |  | | iSCI | | | Albaum et al. (2019) | | CV | | DO | Ankle (la) | | Prop | | | 1 sec | | N.R. | | ICC | PT total: ICC = 0.86 (95% CI 0.49-0.95) |  |  |
|  |  |  |  |  |  |  |  |  |  |  |  |  |  |  |  |  |  |  |  |  |  |  | PT walking: ICC = 0.99 (95% CI 0.96-1.00) |  |  |
|  |  |  |  |  |  |  |  |  |  |  |  |  |  |  |  |  |  |  |  |  |  |  | Self-directed total: ICC = 0.15 (95% CI -0.18-0.55) |  |  |
|  |  |  |  |  |  |  |  |  |  |  |  |  |  |  |  |  |  |  |  |  |  |  | Self-directed walking: ICC = 0.99 (95% ci 0.95-1.00) |  |  |
|  | |  |  | |  | | | Jayaraman et al (2016) | | CV | | DO | Ankle | | Prop (Freedson) | | | 10 sec | | N.R. | | ANOVA (one way) | p>.05 |  |  |
|  | |  |  | |  | | |  | |  | |  | U-arm | | Prop (Freedson) | | | 10 sec | | N.R. | | ANOVA (one way) | P<.05 |  |  |
|  | |  |  | |  | | |  | |  | |  | Waist | | Prop (Freedson) | | | 10 sec | | N.R. | | ANOVA (one way) | p<.05 |  |  |
|  | |  |  | | MS | | | Block et al. (2019) | | CV | | DO | Waist | | Prop | | | 10 sec | | 30 Hz | | ICC | ICC = 0.76 (95% CI 0.63-0.85) |  |  |
|  | |  |  | |  | | | Polhemus (2023) | | CV | | DO | Waist | | Prop | | | N.R. | | 30 Hz | | CCC | CCC = 0.68 (CI 0.37 - 0.82) |  |  |
|  | |  |  | |  | | |  | |  | |  |  | | Prop (+ LFE filter) | | | N.R. | | 30 Hz | | CCC | CCC = 0.73 (0.13 - 0.84) |  |  |
|  | |  |  | | Multiple (cardiovascular, musculoskeletal, neurological, cancer and others) | | | Treacy et al. (2017) | | CV | | DO | Waist | | Prop | | | 1 sec | | N.R. | | ICC | ICC = 0.123 (95% CI -0.071-0.355) |  |  |
|  | |  |  | |  | | | Webber & John (2016) | | CV | | DO | Ankle | | Prop | | | 1 sec | | N.R. | | ICC | ICC = 0.682 (95% CI -0.211-0.895) |  |  |
|  | |  |  | |  | | |  | |  | |  |  | | Prop (+ LFE filter) | | | 1 sec | | N.R. | | ICC | ICC = 0.938 (95% CI 0.870-0.969) |  |  |
|  | |  |  | |  | | |  | |  | |  | Waist | | Prop | | | 1 sec | | N.R. | | ICC | ICC = -0.051 (95% CI -0.191-0.153) |  |  |
|  | |  |  | |  | | |  | |  | |  |  | | Prop (+ LFE filter) | | | 1 sec | | N.R. | | ICC | ICC = 0.829 (95% CI 0.329-0.936) |  |  |
|  | |  |  | | Osteoarthritis | | | Collins et al. (2019) | | CV | | Acc (AG GT3 waist) | Wrist | | Prop | | | 60 sec | | N.R. | | ICC | ICC = 0.602 |  |  |
|  | |  |  | | Parkinson's disease | | | Cederberg et al. (2021) | | CV | | DO | Wrist (ua) | | Prop | | | 60 sec | | 100 Hz | | Mean difference | Overground: 62 ± 114 |  |  |
|  |  |  |  |  |  |  |  |  |  |  |  |  |  |  |  |  |  |  |  |  |  |  | Treadmill: 76 ± 86 |  |  |
|  | |  |  | |  | | |  | |  | |  | Wrist (a) | | Prop | | | 60 sec | | 100 Hz | | Mean difference | Overground: 32 ± 68 |  |  |
|  |  |  |  |  |  |  |  |  |  |  |  |  |  |  |  |  |  |  |  |  |  |  | Treadmill: 66 ± 66 |  |  |
|  | |  |  | | Polymyalgia rheumatica | | | Chandrasekar et al. (2018) | | CV | | DO | Waist | | Prop | | | 60 sec | | 30 Hz | | Bland-Altman LoA | Walking: 141 + (0.5*mean count) [110+(0.5*mean count)] |  |  |
|  | |  |  | |  | | |  | |  | |  |  | | Prop (+ LFE filter) | | | 60 sec | | 30 Hz | | Bland-Altman LoA | Walking: 20 [-40; 81] |  |  |
|  | |  |  | |  | | |  | |  | |  |  | | Prop | | | 60 sec | | 30 Hz | | Bland-Altman LoA | Stairs: 4 [-4; 12] |  |  |
|  | |  |  | |  | | |  | |  | |  |  | | Prop (+ LFE filter) | | | 60 sec | | 30 Hz | | Bland-Altman LoA | Stairs: 0 [-5; 5] |  |  |
|  | |  |  | | Rheumatoid arthritis | | | O'Brien et al. (2020) | | CV | | DO | Thigh | | Prop | | | 1 sec | | 30 Hz | | Bland-Altman LoA | -30 [-116; 57] |  |  |
|  | |  |  | | Stroke | | | Campos et al. (2018) | | CV | | Acc (M X6-2 mini) | Ankle | | Prop | | | 60 sec | | 30 Hz | | ICC | ICC = 0.80 (95% CI 0.63-0.90) |  |  |
|  | |  |  | |  | | |  | |  | |  |  | | Prop (+ LFE filter) | | | 60 sec | | 30 Hz | | ICC | ICC = 0.76 (95% CI 0.56-0.87) |  |  |
|  | |  |  | |  | | |  | |  | |  | Waist | | Prop | | | 60 sec | | 30 Hz | | ICC | ICC = 0.70 (95% CI 0.47-0.84) |  |  |
|  | |  |  | |  | | |  | |  | |  |  | | Prop (+ LFE filter) | | | 60 sec | | 30 Hz | | ICC | ICC = 0.82 (95% CI 0.66-0.90) |  |  |
|  | |  |  | |  | | | Henderson et al. (2021) | | CV | | DO | Ankle (a) | | Prop | | | 60 sec | | 30 Hz | | ICC | PT session: ICC = 0.57 [CI 0.03; 0.80] |  |  |
|  |  |  |  |  |  |  |  |  |  |  |  |  |  |  |  |  |  |  |  |  |  |  | Walk training: ICC = 0.81 [CI -0.04; 0.95] |  |  |
|  | |  |  | |  | | |  | |  | |  |  | | Prop (+ LFE filter) | | | 60 sec | | 30 Hz | | ICC | PT session: ICC = 0.84 [CI 0.69; 0.92] |  |  |
|  |  |  |  |  |  |  |  |  |  |  |  |  |  |  |  |  |  |  |  |  |  |  | Walk training: ICC = 0.96 [CI 0.93; 0.98] |  |  |
|  | |  |  | |  | | |  | |  | |  | Ankle (ua) | | Prop | | | 60 sec | | 30 Hz | | ICC | PT session: ICC = 0.62 [CI 0.16; 0.82] |  |  |
|  |  |  |  |  |  |  |  |  |  |  |  |  |  |  |  |  |  |  |  |  |  |  | Walk training: ICC = 0.86 [CI 0.07; 0.96] |  |  |
|  | |  |  | |  | | |  | |  | |  |  | | Prop (+ LFE filter) | | | 60 sec | | 30 Hz | | ICC | PT session: ICC = 0.77 [CI 0.59; 0.87] |  |  |
|  |  |  |  |  |  |  |  |  |  |  |  |  |  |  |  |  |  |  |  |  |  |  | Walk training: ICC = 0.97 [CI 0.94; 0.98] |  |  |
|  | |  | Intensity time | | Osteoarthritis | | | Collins et al. (2019) | | CV | | Acc (AG GT3 waist) | Wrist | | Cut off: counts < 200 | | | 60 sec | | N.R. | | % bias | Sed: -66% |  |  |
|  | |  |  | |  | | |  | |  | |  |  | | Cut off: 1924 counts/min, bouts of 10 min | | | 60 sec | | N.R. | | Difference | MVPA: +281 min |  |  |
|  | |  | Activity time | | Diabetes mellitus | | | Jao et al. (2017) | | CV | | DO | Waist | | Prop | | | 15 sec | | N.R. | | Accuracy (%) | Standing still: 50.4 ± 48.0% |  |  |
|  |  |  |  |  |  |  |  |  |  |  |  |  |  |  |  |  |  |  |  |  |  |  | Walking (self-paced): 100 ± 0% |  |  |
|  |  |  |  |  |  |  |  |  |  |  |  |  |  |  |  |  |  |  |  |  |  |  | Walking (60 steps/min): 73.3 ± 39.6% |  |  |
|  |  |  |  |  |  |  |  |  |  |  |  |  |  |  |  |  |  |  |  |  |  |  | Walking (100 steps/min): 98.3 ± 9.3% |  |  |
|  |  |  |  |  |  |  |  |  |  |  |  |  |  |  |  |  |  |  |  |  |  |  | Stair climbing (self-paced): 99.2 ± 4.6% |  |  |
|  |  |  |  |  |  |  |  |  |  |  |  |  |  |  |  |  |  |  |  |  |  |  | Stair climbing (60 steps/min): 98.3 ± 5.5% |  |  |
|  |  |  |  |  |  |  |  |  |  |  |  |  |  |  |  |  |  |  |  |  |  |  | sitting 90◦: 80.0 ± 39.1% |  |  |
|  |  |  |  |  |  |  |  |  |  |  |  |  |  |  |  |  |  |  |  |  |  |  | sitting+pedaling 90◦: 46.3 ± 49.3% |  |  |
|  |  |  |  |  |  |  |  |  |  |  |  |  |  |  |  |  |  |  |  |  |  |  | sitting 60◦: 94.8 ± 20.5% |  |  |
|  |  |  |  |  |  |  |  |  |  |  |  |  |  |  |  |  |  |  |  |  |  |  | sitting 45◦: 100 ± 0% |  |  |
|  |  |  |  |  |  |  |  |  |  |  |  |  |  |  |  |  |  |  |  |  |  |  | Lying 30◦: 100 ± 0% |  |  |
|  |  |  |  |  |  |  |  |  |  |  |  |  |  |  |  |  |  |  |  |  |  |  | Lying flat: 100 ± 0% |  |  |
|  |  |  |  |  |  |  |  |  |  |  |  |  |  |  |  |  |  |  |  |  |  |  | Lying left: 41.8 ± 49.8% |  |  |
|  |  |  |  |  |  |  |  |  |  |  |  |  |  |  |  |  |  |  |  |  |  |  | Lying Right: 43.5 ± 49.6% |  |  |
|  | |  |  | | Rheumatoid arthritis | | | O'Brien et al. (2020) | | CV | | DO | Thigh | | Prop | | | 1 sec | | 30 Hz | | Bland-Altman LoA | Sed: 0.1 [-0.1; 0.2] min |  |  |
|  |  |  |  |  |  |  |  |  |  |  |  |  |  |  |  |  |  |  |  |  |  |  | Standing: 0.2 [-0.7; 1.1] min |  |  |
|  |  |  |  |  |  |  |  |  |  |  |  |  |  |  |  |  |  |  |  |  |  |  | Walking: -0.3 [-1.2; 0.6] min |  |  |
|  | |  | Distance walked | | Stroke | | | Compagnat et al. (2019a) | | CV | | DO | Ankle (h) | | Prop | | | 1 sec | | 30 Hz | | Pearson’s r | r = 0.95 |  |  |
|  | |  |  | |  | | |  | |  | |  | Ankle (a) | | Prop | | | 1 sec | | 30 Hz | | Pearson’s r | r = 0.93 |  |  |
|  | |  |  | |  | | |  | |  | |  | Waist | | Prop | | | 1 sec | | 30 Hz | | Pearson’s r | r = 0.86 |  |  |
|  | |  |  | |  | | |  | |  | |  | Wrist (h) | | Prop | | | 1 sec | | 30 Hz | | Pearson’s r | r = 0.79 |  |  |
|  | |  |  | |  | | |  | |  | |  | Wrist (a) | | Prop | | | 1 sec | | 30 Hz | | Pearson’s r | r = 0.81 |  |  |
|  | |  |  | | Peripheral artery disease | | | Taoum et al. (2020) | | CV | | GPS | Hip | | Cust | | | 0.033 sec | | 30 Hz | | MAPE | Using raw accelerations: 12.5 ± 7.9 |  |  |
|  | |  |  | |  | | |  | |  | |  |  | |  | | | 1 sec | | 30 Hz | | MAPE | Using VM - normal filter: 12.5 ± 8.5 |  |  |
|  |  |  |  |  |  |  |  |  |  |  |  |  |  |  |  |  |  |  |  |  |  |  | Using VM - LFE filter: 11.9 ± 7.4 |  |  |
|  |  |  |  |  |  |  |  |  |  |  |  |  |  |  |  |  |  |  |  |  |  |  | Using steps - nomral filter: 17.4 ± 9.7 |  |  |
|  |  |  |  |  |  |  |  |  |  |  |  |  |  |  |  |  |  |  |  |  |  |  | Using steps - LFE filter: 18.8 ± 10.3 |  |  |
|  | |  | Counts | | Amputation | | | Ladlow et al. (2017) | | CV | | IC | Waist (SRL) | | Prop | | | N.R. | | 30 Hz | | Pearson’s r | Unilateral: r = 0.82 |  |  |
|  |  |  |  |  |  |  |  |  |  |  |  |  |  |  |  |  |  |  |  |  |  |  | Bilateral: r = 0.92 |  |  |
|  | |  |  | |  | | |  | |  | |  | Waist (LRL) | | Prop | | | N.R. | | 30 Hz | | Pearson’s r | Unilateral: r = 0.76 |  |  |
|  |  |  |  |  |  |  |  |  |  |  |  |  |  |  |  |  |  |  |  |  |  |  | Bilateral: r = 0.80 |  |  |
|  | |  |  | |  | | |  | |  | |  | Waist (Sp) | | Prop | | | N.R. | | 30 Hz | | Pearson’s r | Unilateral: r = 0.68 |  |  |
|  |  |  |  |  |  |  |  |  |  |  |  |  |  |  |  |  |  |  |  |  |  |  | Bilateral: r = 0.80 |  |  |
|  | |  | MET | | Stroke | | | Jayaraman et al (2018) | | CV | | IC | Ankle (a) | | Prop (Freedson + Harris Benedict BMR | | | N.R. | | 30 Hz | | Kruskal wallis (Games-Howell post hoc) | Lying: mean diff = 0.17, p = .71 |  |  |
|  |  |  |  |  |  |  |  |  |  |  |  |  |  |  |  |  |  |  |  |  |  |  | Sitting: mean diff = -0.14, p = .39 |  |  |
|  |  |  |  |  |  |  |  |  |  |  |  |  |  |  |  |  |  |  |  |  |  |  | Standing: mean diff = 0.01, p = 1.00 |  |  |
|  |  |  |  |  |  |  |  |  |  |  |  |  |  |  |  |  |  |  |  |  |  |  | 50 SWT: mean diff = -0.47, p = .92 |  |  |
|  |  |  |  |  |  |  |  |  |  |  |  |  |  |  |  |  |  |  |  |  |  |  | 6 MWT: mean diff = -0.60, p = .70 |  |  |
|  |  |  |  |  |  |  |  |  |  |  |  |  |  |  |  |  |  |  |  |  |  |  | Sit-to-stand: mean diff = 2.29, p = .003 |  |  |
|  | |  |  | |  | | |  | |  | |  | Ankle (ua) | | Prop (Freedson + Harris Benedict BMR | | | N.R. | | 30 Hz | | Kruskal wallis (Games-Howell post hoc) | Lying: mean diff = 0.07, p = .11 |  |  |
|  |  |  |  |  |  |  |  |  |  |  |  |  |  |  |  |  |  |  |  |  |  |  | Sitting: mean diff = -0.11, p = .27 |  |  |
|  |  |  |  |  |  |  |  |  |  |  |  |  |  |  |  |  |  |  |  |  |  |  | Standing: mean diff = 0.04, p = .99 |  |  |
|  |  |  |  |  |  |  |  |  |  |  |  |  |  |  |  |  |  |  |  |  |  |  | 50 SWT: mean diff = -2.18, p = .001 |  |  |
|  |  |  |  |  |  |  |  |  |  |  |  |  |  |  |  |  |  |  |  |  |  |  | 6 MWT: mean diff = -2.70, p = .04 |  |  |
|  |  |  |  |  |  |  |  |  |  |  |  |  |  |  |  |  |  |  |  |  |  |  | Sit-to-stand: mean diff = 2.16, p = .01 |  |  |
|  | |  |  | |  | | |  | |  | |  | U arm (a) | | Prop (Freedson + Harris Benedict BMR | | | N.R. | | 30 Hz | | Kruskal wallis (Games-Howell post hoc) | Lying: mean diff = 0.17, p = .71 |  |  |
|  |  |  |  |  |  |  |  |  |  |  |  |  |  |  |  |  |  |  |  |  |  |  | Sitting: mean diff = -0.14, p = .39 |  |  |
|  |  |  |  |  |  |  |  |  |  |  |  |  |  |  |  |  |  |  |  |  |  |  | Standing: mean diff = 0.01, p = 1.00 |  |  |
|  |  |  |  |  |  |  |  |  |  |  |  |  |  |  |  |  |  |  |  |  |  |  | 50 SWT: mean diff = -0.34, p = .92 |  |  |
|  |  |  |  |  |  |  |  |  |  |  |  |  |  |  |  |  |  |  |  |  |  |  | 6 MWT: mean diff = -0.73, p = .52 |  |  |
|  |  |  |  |  |  |  |  |  |  |  |  |  |  |  |  |  |  |  |  |  |  |  | Sit-to-stand: mean diff = 1.23, p = .32 |  |  |
|  | |  |  | |  | | |  | |  | |  | U arm (ua) | | Prop (Freedson + Harris Benedict BMR | | | N.R. | | 30 Hz | | Kruskal wallis (Games-Howell post hoc) | Lying: mean diff = 0.07, p = .11 |  |  |
|  |  |  |  |  |  |  |  |  |  |  |  |  |  |  |  |  |  |  |  |  |  |  | Sitting: mean diff = -0.12, p = .24 |  |  |
|  |  |  |  |  |  |  |  |  |  |  |  |  |  |  |  |  |  |  |  |  |  |  | Standing: mean diff =-0.02, p = 1.00 |  |  |
|  |  |  |  |  |  |  |  |  |  |  |  |  |  |  |  |  |  |  |  |  |  |  | 50 SWT: mean diff = -0.04, p = 1.00 |  |  |
|  |  |  |  |  |  |  |  |  |  |  |  |  |  |  |  |  |  |  |  |  |  |  | 6 MWT: mean diff = -0.35, p = 1.00 |  |  |
|  |  |  |  |  |  |  |  |  |  |  |  |  |  |  |  |  |  |  |  |  |  |  | Sit-to-stand: mean diff = -1.14, p = .41 |  |  |
|  | |  |  | |  | | |  | |  | |  | Waist (a) | | Prop (Freedson + Harris Benedict BMR | | | N.R. | | 30 Hz | | Kruskal wallis (Games-Howell post hoc) | Lying: mean diff = 0.17, p = .71 |  |  |
|  |  |  |  |  |  |  |  |  |  |  |  |  |  |  |  |  |  |  |  |  |  |  | Sitting: mean diff = -0.14, p = .39 |  |  |
|  |  |  |  |  |  |  |  |  |  |  |  |  |  |  |  |  |  |  |  |  |  |  | Standing: mean diff = 0.01, p = 1.00 |  |  |
|  |  |  |  |  |  |  |  |  |  |  |  |  |  |  |  |  |  |  |  |  |  |  | 50 SWT: mean diff = 0.18, p = .99 |  |  |
|  |  |  |  |  |  |  |  |  |  |  |  |  |  |  |  |  |  |  |  |  |  |  | 6 MWT: mean diff = -0.15, p = .99 |  |  |
|  |  |  |  |  |  |  |  |  |  |  |  |  |  |  |  |  |  |  |  |  |  |  | Sit-to-stand: mean diff = -0.56, p = .84 |  |  |
|  | |  |  | |  | | |  | |  | |  | Waist (ua) | | Prop (Freedson + Harris Benedict BMR | | | N.R. | | 30 Hz | | Kruskal wallis (Games-Howell post hoc) | Lying: mean diff = 0.05, p = .45 |  |  |
|  |  |  |  |  |  |  |  |  |  |  |  |  |  |  |  |  |  |  |  |  |  |  | Sitting: mean diff = -0.11, p = .27 |  |  |
|  |  |  |  |  |  |  |  |  |  |  |  |  |  |  |  |  |  |  |  |  |  |  | Standing: mean diff = 0.04, p = .99 |  |  |
|  |  |  |  |  |  |  |  |  |  |  |  |  |  |  |  |  |  |  |  |  |  |  | 50 SWT: mean diff = 0.28, p = .94 |  |  |
|  |  |  |  |  |  |  |  |  |  |  |  |  |  |  |  |  |  |  |  |  |  |  | 6 MWT: mean diff = 0.70, p = .98 |  |  |
|  |  |  |  |  |  |  |  |  |  |  |  |  |  |  |  |  |  |  |  |  |  |  | Sit-to-stand: mean diff = -0.33, p = .99 |  |  |
|  | |  |  | | iSCI | | | Jayaraman et al (2018) | | CV | | IC | Ankle | | Prop (Freedson + Harris Benedict BMR | | | N.R. | | 30 Hz | | Kruskal wallis (Games-Howell post hoc) | Lying: mean diff: 1.58, p < .001 |  |  |
|  |  |  |  |  |  |  |  |  |  |  |  |  |  |  |  |  |  |  |  |  |  |  | Sitting: mean diff: 0.12, p < .547 |  |  |
|  |  |  |  |  |  |  |  |  |  |  |  |  |  |  |  |  |  |  |  |  |  |  | Standing: mean diff = 0.04, p = .960 |  |  |
|  |  |  |  |  |  |  |  |  |  |  |  |  |  |  |  |  |  |  |  |  |  |  | 50 SWT: mean diff = 0.34, p = .965 |  |  |
|  |  |  |  |  |  |  |  |  |  |  |  |  |  |  |  |  |  |  |  |  |  |  | 6 MWT: mean diff = 0.57, p = .917 |  |  |
|  |  |  |  |  |  |  |  |  |  |  |  |  |  |  |  |  |  |  |  |  |  |  | Sit-to-stand: mean diff = 1.68, p < .001 |  |  |
|  | |  |  | |  | | |  | |  | |  | U arm | | Prop (Freedson + Harris Benedict BMR | | | N.R. | | 30 Hz | | Kruskal wallis (Games-Howell post hoc) | Lying: mean diff: 0.12, p < .547 |  |  |
|  |  |  |  |  |  |  |  |  |  |  |  |  |  |  |  |  |  |  |  |  |  |  | Sitting: mean diff = 0.04, p = .960 |  |  |
|  |  |  |  |  |  |  |  |  |  |  |  |  |  |  |  |  |  |  |  |  |  |  | Standing: mean diff = 0.22, p = .108 |  |  |
|  |  |  |  |  |  |  |  |  |  |  |  |  |  |  |  |  |  |  |  |  |  |  | 50 SWT: mean diff = 1.22, p = .001 |  |  |
|  |  |  |  |  |  |  |  |  |  |  |  |  |  |  |  |  |  |  |  |  |  |  | 6 MWT: mean diff = 1.48, p = .0.84 |  |  |
|  |  |  |  |  |  |  |  |  |  |  |  |  |  |  |  |  |  |  |  |  |  |  | Sit-to-stand: mean diff = -1.20, p = .341 |  |  |
|  | |  |  | |  | | |  | |  | |  | Waist | | Prop (Freedson + Harris Benedict BMR | | | N.R. | | 30 Hz | | Kruskal wallis (Games-Howell post hoc) | Lying: mean diff: 0.12, p < .547 |  |  |
|  |  |  |  |  |  |  |  |  |  |  |  |  |  |  |  |  |  |  |  |  |  |  | Sitting: mean diff = 0.04, p = .960 |  |  |
|  |  |  |  |  |  |  |  |  |  |  |  |  |  |  |  |  |  |  |  |  |  |  | Standing: mean diff = 0.22, p = .108 |  |  |
|  |  |  |  |  |  |  |  |  |  |  |  |  |  |  |  |  |  |  |  |  |  |  | 50 SWT: mean diff = 1.40, p = .001 |  |  |
|  |  |  |  |  |  |  |  |  |  |  |  |  |  |  |  |  |  |  |  |  |  |  | 6 MWT: mean diff = 2.18, p = .002 |  |  |
|  |  |  |  |  |  |  |  |  |  |  |  |  |  |  |  |  |  |  |  |  |  |  | Sit-to-stand: mean diff = 1.44, p < .001 |  |  |
|  | |  | Vector magnitude | | Inflammatory myopathy | | | Rockette-Wagner et al. (2021) | | Con V, conv | | Functional test, TUG | Waist | | Prop | | | 1 sec | | N.R. | | Pearson's r | r = -0.35 (-0.59 - -0.04) |  |  |
|  | |  |  | |  | | |  | |  | | Functional test, 30STS | Waist | | Prop | | | 2 sec | | N.R. | | Pearson's r | r = 0.49 (0.22 - 0.68) |  |  |
|  | |  |  | |  | | |  | |  | | Test, 6MWT | Waist | | Prop | | | 3 sec | | N.R. | | Pearson's r | r = 0.60 (0.36 - 0.76) |  |  |
|  | |  |  | |  | | |  | |  | | Functional SR - SF36 PF | Waist | | Prop | | | 4 sec | | N.R. | | Pearson's r | r = 0.47 (0.18 - 0.67) |  |  |
|  | |  |  | |  | | |  | |  | | Funcitonal SR - PROMIS PF-20 | Waist | | Prop | | | 5 sec | | N.R. | | Pearson's r | r = 0.48 (0.20 - 0.67) |  |  |
|  | |  |  | |  | | |  | | Con V, div | | Funcitonal - Cutaneous disease activity | Waist | | Prop | | | 6 sec | | N.R. | | Pearson's r | r = -0.05 (-0.34 - 0.25) |  |  |
|  | |  |  | |  | | |  | |  | | Funcitonal - Pulmonary disease activity | Waist | | Prop | | | 7 sec | | N.R. | | Pearson's r | r = -0.07 (-0.36 - 0.22) |  |  |
|  | |  |  | |  | | |  | |  | | Functional - Extramuscular disease activity | Waist | | Prop | | | 8 sec | | N.R. | | Pearson's r | r = -0.07 (-0.52 - 0.02) |  |  |
|  | |  |  | |  | | |  | | TRT R | |  | Waist | | Prop | | | 1 sec | | N.R. | | ICC | ICC = 0.80 (CI 0.62 – 0.89) |  |  |
|  | |  |  | |  | | |  | | Resp | | SR - total improvement score | Waist | | Prop | | | 1 sec | | N.R. | | Pearson's r | r = 0.53 (CI 0.20 - 0.75) |  |  |
| GTX9 | | Accelerometer | Steps | | Amputation | | | Smith & Guerra (2021) | | CV | | DO | Wrist | | N.R. | | | N.R. | | 50 Hz | | ICC | ICC = 0.005 (CI -0.256 - 0.299) |  |  |
|  | |  |  | |  | | |  | |  | |  | Ankle | | N.R. | | | N.R. | | 50 Hz | | ICC | ICC = 0.111 (CI -0.202 - 0.418) |  |  |
|  | |  |  | | MS | | | Anens et al. (2023) | | CV | | DO | Waist | | Prop | | | 1 sec | | 90 Hz | | Spearman's rho | Comfortable: rho = 0.79 |  |  |
|  |  |  |  |  |  |  |  |  |  |  |  |  |  |  |  |  |  |  |  |  |  |  | Fast: rho = 0.75 |  |  |
|  |  |  |  |  |  |  |  |  |  |  |  |  |  |  |  |  |  |  |  |  |  |  | Slow: rho = 0.75 |  |  |
|  |  |  |  |  |  |  |  |  |  |  |  |  |  |  |  |  |  |  |  |  |  |  | Total: rho = 0.74 |  |  |
|  | |  |  | |  | | |  | |  | |  |  | | Prop (+LFE filter) | | | 1 sec | | 90 Hz | | Spearman's rho | Comfortable: rho = 0.93 |  |  |
|  |  |  |  |  |  |  |  |  |  |  |  |  |  |  |  |  |  |  |  |  |  |  | Fast: rho = 0.92 |  |  |
|  |  |  |  |  |  |  |  |  |  |  |  |  |  |  |  |  |  |  |  |  |  |  | Slow: rho = 0.85 |  |  |
|  |  |  |  |  |  |  |  |  |  |  |  |  |  |  |  |  |  |  |  |  |  |  | Total: rho = 0.89 |  |  |
|  | |  |  | | Peripheral artery disease | | | Ata et al. (2018) | | CV | | DO | Waist | | Prop | | | 1 sec | | 100 Hz | | % error | -3.1 ± 10.3% |  |  |
|  | |  | Sedentary time | | COPD | | | Webster et al. (2021) | | CV | | Acc (GENEActive - thigh) | Waist | | Prop (multiple) | | | N.R. | | 30 Hz | | CCC | Vertical Axis or Vector Magnitude, filter, non-wear time, sedentary cut point |  |  |
|  |  |  |  |  |  |  |  |  |  |  |  |  |  |  |  |  |  |  |  |  |  |  | VA, normal, 60min, <1c/15s: ccc = 0.729 (0.661, 0.796) |  |  |
|  |  |  |  |  |  |  |  |  |  |  |  |  |  |  |  |  |  |  |  |  |  |  | VA, normal, 60min, 10c/15s: ccc = 0.680 (0.598, 0.762) |  |  |
|  |  |  |  |  |  |  |  |  |  |  |  |  |  |  |  |  |  |  |  |  |  |  | VA, normal, 90min, 1c/15s: ccc = 0.753 (0.686, 0.821) |  |  |
|  |  |  |  |  |  |  |  |  |  |  |  |  |  |  |  |  |  |  |  |  |  |  | VA, normal, 90min, 10c/15s: ccc = 0.679 (0.598, 0.760) |  |  |
|  |  |  |  |  |  |  |  |  |  |  |  |  |  |  |  |  |  |  |  |  |  |  | VA, normal, 120min, 1c/15s: ccc = 0.774 (0.709, 0.839) |  |  |
|  |  |  |  |  |  |  |  |  |  |  |  |  |  |  |  |  |  |  |  |  |  |  | VA, normal, 120min, 10c/15s: ccc = 0.689 (0.606, 0.772) |  |  |
|  |  |  |  |  |  |  |  |  |  |  |  |  |  |  |  |  |  |  |  |  |  |  | VA, LFE, 60min, <1c/15s: ccc = 0.614 (0.529, 0.699) |  |  |
|  |  |  |  |  |  |  |  |  |  |  |  |  |  |  |  |  |  |  |  |  |  |  | VA, LFE, 60min, 10c/15s: ccc = 0.766 (0.701, 0.831) |  |  |
|  |  |  |  |  |  |  |  |  |  |  |  |  |  |  |  |  |  |  |  |  |  |  | VA, LFE, 90min, 1c/15s: ccc = 0.643 (0.558, 0.728) |  |  |
|  |  |  |  |  |  |  |  |  |  |  |  |  |  |  |  |  |  |  |  |  |  |  | VA, LFE, 90min, 10c/15s: ccc = 0.771 (0.703, 0.839) |  |  |
|  |  |  |  |  |  |  |  |  |  |  |  |  |  |  |  |  |  |  |  |  |  |  | VA, LFE, 120min, 1c/15s: ccc = 0.664 (0.581, 0.748) |  |  |
|  |  |  |  |  |  |  |  |  |  |  |  |  |  |  |  |  |  |  |  |  |  |  | VA, LFE, 120min, 10c/15s: ccc = 0.782 (0.720, 0.844) |  |  |
|  |  |  |  |  |  |  |  |  |  |  |  |  |  |  |  |  |  |  |  |  |  |  | VM, normal, 60min, 19c/15s: ccc = 0.780 (0.716, 0.844) |  |  |
|  |  |  |  |  |  |  |  |  |  |  |  |  |  |  |  |  |  |  |  |  |  |  | VM, normal, 60min, 20c/15s: ccc = 0.784 (0.721, 0.846) |  |  |
|  |  |  |  |  |  |  |  |  |  |  |  |  |  |  |  |  |  |  |  |  |  |  | VM, normal, 60min, 70c/15s: ccc = 0.761 (0.694, 0.828) |  |  |
|  |  |  |  |  |  |  |  |  |  |  |  |  |  |  |  |  |  |  |  |  |  |  | VM, normal, 90min, 19c/15s: ccc = 0.807 (0.750, 0.864) |  |  |
|  |  |  |  |  |  |  |  |  |  |  |  |  |  |  |  |  |  |  |  |  |  |  | VM, normal, 90min, 20c/15s: ccc = 0.810 (0.752, 0.868) |  |  |
|  |  |  |  |  |  |  |  |  |  |  |  |  |  |  |  |  |  |  |  |  |  |  | VM, normal, 90min, 70c/15s: ccc = 0.763 (0.698, 0.827) |  |  |
|  |  |  |  |  |  |  |  |  |  |  |  |  |  |  |  |  |  |  |  |  |  |  | VM, normal, 120min, 19c/15s: ccc = 0.828 (0.773, 0.884) |  |  |
|  |  |  |  |  |  |  |  |  |  |  |  |  |  |  |  |  |  |  |  |  |  |  | VM, normal, 120min, 20c/15s: ccc = 0.831 (0.778, 0.883) |  |  |
|  |  |  |  |  |  |  |  |  |  |  |  |  |  |  |  |  |  |  |  |  |  |  | VM, normal, 120min, 70c/15s: ccc = 0.770 (0.703, 0.837) |  |  |
|  |  |  |  |  |  |  |  |  |  |  |  |  |  |  |  |  |  |  |  |  |  |  | VM, LFE, 60min, 19c/15s: ccc = 0.736 (0.658, 0.815) |  |  |
|  |  |  |  |  |  |  |  |  |  |  |  |  |  |  |  |  |  |  |  |  |  |  | VM, LFE, 60min, 20c/15s: ccc = 0.814 (0.756, 0.872) |  |  |
|  |  |  |  |  |  |  |  |  |  |  |  |  |  |  |  |  |  |  |  |  |  |  | VM, LFE, 60min, 70c/15s: ccc = 0.811 (0.753, 0.869) |  |  |
|  |  |  |  |  |  |  |  |  |  |  |  |  |  |  |  |  |  |  |  |  |  |  | VM, LFE, 90min, 19c/15s: ccc = 0.756 (0.682, 0.831) |  |  |
|  |  |  |  |  |  |  |  |  |  |  |  |  |  |  |  |  |  |  |  |  |  |  | VM, LFE, 90min, 20c/15s: ccc = 0.828 (0.773, 0.883) |  |  |
|  |  |  |  |  |  |  |  |  |  |  |  |  |  |  |  |  |  |  |  |  |  |  | VM, LFE, 90min, 70c/15s: ccc = 0.816 (0.760, 0.873) |  |  |
|  |  |  |  |  |  |  |  |  |  |  |  |  |  |  |  |  |  |  |  |  |  |  | VM, LFE, 120min, 19c/15s: ccc = 0.770 (0.701, 0.839) |  |  |
|  |  |  |  |  |  |  |  |  |  |  |  |  |  |  |  |  |  |  |  |  |  |  | VM, LFE, 120min, 20c/15s: ccc = 0.838 (0.787, 0.889) |  |  |
|  |  |  |  |  |  |  |  |  |  |  |  |  |  |  |  |  |  |  |  |  |  |  | VM, LFE, 120min, 70c/15s: ccc = 0.821 (0.766, 0.876) |  |  |
|  | |  |  | | MS | | | Anens et al. (2023) | | CV | | DO | Waist | | Prop | | | 1 sec | | 90 Hz | | Spearman's rho | Sitting: rho = 0.18 |  |  |
|  |  |  |  |  |  |  |  |  |  |  |  |  |  |  |  |  |  |  |  |  |  |  | Lying: rho = 0.22 |  |  |
|  |  |  |  |  |  |  |  |  |  |  |  |  |  |  |  |  |  |  |  |  |  |  | Lying side: rho = 0.39 |  |  |
|  | |  |  | |  | | |  | |  | |  |  | | Prop (+LFE filter) | | | 1 sec | | 90 Hz | | Spearman's rho | Sitting: rho = 0.16 |  |  |
|  |  |  |  |  |  |  |  |  |  |  |  |  |  |  |  |  |  |  |  |  |  |  | Lying: rho = 0.22 |  |  |
|  |  |  |  |  |  |  |  |  |  |  |  |  |  |  |  |  |  |  |  |  |  |  | Lying side: rho = 0.38 |  |  |
| **Pal Technologies** | | | | | | | | | | | | | | | | | | | | | | | | |  |
| ActivPAL | | Accelerometer | Steps | | Diabetes mellitus | | | Alothman et al. (2020) | | TRT R | |  | Thight | | Prop | | | 0.1 sec | | 10 Hz | | ICC | ICC = 0.91 |  |  |
|  | |  |  | |  | | | Jao et al. (2017) | | CV | | DO | Thight | | Prop | | | 1 sec | | N.R. | | Accuracy (%) | Self paced: 98.3 ± 2.5% |  |  |
|  |  |  |  |  |  |  |  |  |  |  |  |  |  |  |  |  |  |  |  |  |  |  | 60 steps/min: 90.7 ± 10.0% |  |  |
|  |  |  |  |  |  |  |  |  |  |  |  |  |  |  |  |  |  |  |  |  |  |  | 100 steps/min: 98.5 ± 2.1% |  |  |
|  |  |  |  |  |  |  |  |  |  |  |  |  |  |  |  |  |  |  |  |  |  |  | Stair self paced: 91.2 ± 8.1% |  |  |
|  |  |  |  |  |  |  |  |  |  |  |  |  |  |  |  |  |  |  |  |  |  |  | Stair 60 steps/min: 91.7 ± 7.2% |  |  |
|  | |  |  | | Multi (musculoskeletal, neurological and other) | | | Treacy et al. (2017) | | CV | | DO | Thight | | Prop | | | N.R. | | N.R. | | ICC | ICC = 0.781 [CI 0.231; 0.911] |  |  |
|  | |  |  | | Rheumatoid arthritis | | | Larkin et al. (2016) | | CV | | DO | Thight | | Prop | | | 15 sec | | N.R. | | Pearson’s r | r = 0.94 [CI 0.86; 0.98] |  |  |
|  | |  |  | | Stroke | | | Mahendran et al. (2016) | | CV | | DO | Thight | | Prop | | | 15 sec | | 10 Hz | | ICC | T slow: ICC = 0.718 [CI 0.343; 0.865] |  |  |
|  |  |  |  |  |  |  |  |  |  |  |  |  |  |  |  |  |  |  |  |  |  |  | T comf: ICC = 0.758 [CI 0.419; 0.912] |  |  |
|  |  |  |  |  |  |  |  |  |  |  |  |  |  |  |  |  |  |  |  |  |  |  | T fast: ICC = 0.855 [CI 0.622; 0.949] |  |  |
|  |  |  |  |  |  |  |  |  |  |  |  |  |  |  |  |  |  |  |  |  |  |  | 6MWT: ICC = 0.994 [CI 0.982; 0.998] |  |  |
|  |  |  |  |  |  |  |  |  |  |  |  |  |  |  |  |  |  |  |  |  |  |  | Circuit: ICC = 0.992 [CI 0.976; 0.997] |  |  |
|  | |  |  | |  | | |  | | TRT R | |  | Thight | | Prop | | | 15 sec | | 10 Hz | | ICC | T slow: ICC = 0.894 [CI 0.703; 0.965] |  |  |
|  |  |  |  |  |  |  |  |  |  |  |  |  |  |  |  |  |  |  |  |  |  |  | T comf: ICC = 0.659 [CI 0.194; 0.882] |  |  |
|  |  |  |  |  |  |  |  |  |  |  |  |  |  |  |  |  |  |  |  |  |  |  | T fast: ICC = 0.902 [CI 0.712; 0.969] |  |  |
|  |  |  |  |  |  |  |  |  |  |  |  |  |  |  |  |  |  |  |  |  |  |  | 6MWT: ICC = 0.977 [CI 0.930; 0.991] |  |  |
|  |  |  |  |  |  |  |  |  |  |  |  |  |  |  |  |  |  |  |  |  |  |  | Circuit: ICC = 0.960 [CI 0.881; 0.987] |  |  |
|  | |  | Activity time | | Amputation | | | Salih et al. (2016) | | CV | | DO | Thight (a) | | N.R. | | | 15 sec | | N.R. | | Bland-Altman LoA | Walking: 0.11 [-0.43; 0.66] sec (ue) |  |  |
|  | |  |  | |  | | |  | |  | |  | Thight (ua) | | N.R. | | | 15 sec | | N.R. | | Bland-Altman LoA | Walking: 0.004 [-0.09; 0.10] sec (ue) |  |  |
|  | |  |  | | Diabetes mellitus | | | Alothman et al. (2020) | | TRT R | |  | Thight | | Prop | | | 0.1 sec | | 10 Hz | | ICC | Sed: ICC = 0.79 |  |  |
|  |  |  |  |  |  |  |  |  |  |  |  |  |  |  |  |  |  |  |  |  |  |  | Standing: ICC = 0.74 |  |  |
|  |  |  |  |  |  |  |  |  |  |  |  |  |  |  |  |  |  |  |  |  |  |  | Walking: ICC = 0.90 |  |  |
|  |  |  |  |  |  |  |  |  |  |  |  |  |  |  |  |  |  |  |  |  |  |  | Sit to stand: ICC = 0.90 |  |  |
|  | |  |  | |  | | | Jao et al. (2017) | | CV | | DO | Thight | | Prop | | | 1 sec | | N.R. | | Accuracy (%) | Standing still: 100 ± 0% |  |  |
|  |  |  |  |  |  |  |  |  |  |  |  |  |  |  |  |  |  |  |  |  |  |  | Walking (self-paced): 100 ± 0% |  |  |
|  |  |  |  |  |  |  |  |  |  |  |  |  |  |  |  |  |  |  |  |  |  |  | Walking (60 steps/min): 98.0 ± 6.0% |  |  |
|  |  |  |  |  |  |  |  |  |  |  |  |  |  |  |  |  |  |  |  |  |  |  | Walking (100 steps/min): 100 ± 0% |  |  |
|  |  |  |  |  |  |  |  |  |  |  |  |  |  |  |  |  |  |  |  |  |  |  | Stair climbing (self-paced): 97.6 ± 4.7% |  |  |
|  |  |  |  |  |  |  |  |  |  |  |  |  |  |  |  |  |  |  |  |  |  |  | Stair climbing (60 steps/min): 98.5 ± 5.5% |  |  |
|  |  |  |  |  |  |  |  |  |  |  |  |  |  |  |  |  |  |  |  |  |  |  | sitting 90◦: 96.7 ± 18.3% |  |  |
|  |  |  |  |  |  |  |  |  |  |  |  |  |  |  |  |  |  |  |  |  |  |  | sitting+pedaling 90◦: 100 ± 0% |  |  |
|  |  |  |  |  |  |  |  |  |  |  |  |  |  |  |  |  |  |  |  |  |  |  | sitting 60◦: 96.6 ± 18.6% |  |  |
|  |  |  |  |  |  |  |  |  |  |  |  |  |  |  |  |  |  |  |  |  |  |  | sitting 45◦: 96.7 ± 18.3% |  |  |
|  |  |  |  |  |  |  |  |  |  |  |  |  |  |  |  |  |  |  |  |  |  |  | Lying 30◦: 96.7 ± 18.3% |  |  |
|  |  |  |  |  |  |  |  |  |  |  |  |  |  |  |  |  |  |  |  |  |  |  | Lying flat: 100 ± 0% |  |  |
|  |  |  |  |  |  |  |  |  |  |  |  |  |  |  |  |  |  |  |  |  |  |  | Lying left: 100 ± 0% |  |  |
|  |  |  |  |  |  |  |  |  |  |  |  |  |  |  |  |  |  |  |  |  |  |  | Lying Right: 100 ± 0% |  |  |
|  | |  |  | | Rheumatoid arthritis | | | Larkin et al. (2016) | | CV | | DO | Thight | | Prop | | | 15 sec | | N.R. | | Pearson’s r | Sed: r = 0.74 [CI 0.44; 0.89] |  |  |
|  |  |  |  |  |  |  |  |  |  |  |  |  |  |  |  |  |  |  |  |  |  |  | Standing and light activity: r = 0.86 [CI 0.74; 0.96] |  |  |
|  |  |  |  |  |  |  |  |  |  |  |  |  |  |  |  |  |  |  |  |  |  |  | Walking: r = 0.93 [CI 0.83; 0.97] |  |  |
|  | |  |  | | Stroke | | | Mahendran et al. (2016) | | CV | | DO | Thight | | Prop | | | 15 sec | | 10 Hz | | ICC | Walking Circuit: ICC = 0.997 [CI 0.990; 0.999] |  |  |
|  | |  |  | |  | | |  | |  | |  |  | |  | | |  | |  | | Absolute % error | Walking t slow: 1.1% |  |  |
|  |  |  |  |  |  |  |  |  |  |  |  |  |  |  |  |  |  |  |  |  |  |  | Walking t comf: 3.2% |  |  |
|  |  |  |  |  |  |  |  |  |  |  |  |  |  |  |  |  |  |  |  |  |  |  | Walking t fast: 3.1% |  |  |
|  |  |  |  |  |  |  |  |  |  |  |  |  |  |  |  |  |  |  |  |  |  |  | Walking 6MWT: 0.3% |  |  |
|  | |  |  | |  | | |  | | TRT R | |  | Thight | | Prop | | | 15 sec | | 10 Hz | | ICC | Walking t slow: ICC = 0.659 [CI -0.193; 0.736] |  |  |
|  |  |  |  |  |  |  |  |  |  |  |  |  |  |  |  |  |  |  |  |  |  |  | Walking 6MWT: ICC = 0.981 [CI 0.942; 0.994] |  |  |
|  |  |  |  |  |  |  |  |  |  |  |  |  |  |  |  |  |  |  |  |  |  |  | Walking Circuit: ICC = 0.828 [CI 0.527; 0.944] |  |  |
|  | |  |  | |  | | |  | |  | |  |  | |  | | |  | |  | | Absolute % error | Walking t comf: 6.5% |  |  |
|  |  |  |  |  |  |  |  |  |  |  |  |  |  |  |  |  |  |  |  |  |  |  | Walking t fast: 3.3% |  |  |
|  | |  | MET | | Stroke | | | Mahendran et al. (2016) | | TRT R | |  | Thight | | Prop | | | 15 sec | | 10 Hz | | ICC | T slow: ICC = 0.821 [CI 0.530; 0.939] |  |  |
|  |  |  |  |  |  |  |  |  |  |  |  |  |  |  |  |  |  |  |  |  |  |  | T comf: ICC = 0.654 [CI 0.187; 0.880] |  |  |
|  |  |  |  |  |  |  |  |  |  |  |  |  |  |  |  |  |  |  |  |  |  |  | T fast: ICC = 0.912 [CI 0.737; 0.972] |  |  |
|  |  |  |  |  |  |  |  |  |  |  |  |  |  |  |  |  |  |  |  |  |  |  | 6MWT: ICC = 0.928 [CI 0.791; 0.976] |  |  |
|  |  |  |  |  |  |  |  |  |  |  |  |  |  |  |  |  |  |  |  |  |  |  | Circuit: ICC = 0.991 [CI 0.971; 0.997] |  |  |
| ActivPAL3 | | Accelerometer | Steps | | MS | | | Coulter et al. (2017) | | CV | | DO | Thight | | Prop | | | N.R. | | 20 Hz | | Bland-Altman LoA | -4.7 [-22.88; 13.47] (ue) |  |  |
|  | |  |  | | Rheumatoid arthritis | | | O'Brien et al. (2020) | | CV | | DO | Thight | | Prop | | |  | |  | | Bland-Altman LoA | -30 [-116; 57] (ue) |  |  |
|  | |  | Activity time | | MS | | | Coulter et al. (2017) | | CV | | DO | Thight | | Prop | | | N.R. | | 20 Hz | | Bland-Altman LoA | Walking: -4.55 [-26.06; 16.96] sec (ue) |  |  |
|  |  |  |  |  |  |  |  |  |  |  |  |  |  |  |  |  |  |  |  |  |  |  | Upright position: 1.11 [-1.12; 3.34] sec (oe) |  |  |
|  | |  |  | | Rheumatoid arthritis | | | O'Brien et al. (2020) | | CV | | DO | Thight | | Prop | | | N.R. | | N.R. | | Bland-Altman LoA | Sed: 0.1 [-0.1; 0.2] min (oe) |  |  |
|  |  |  |  |  |  |  |  |  |  |  |  |  |  |  |  |  |  |  |  |  |  |  | Standing: 0.2 [-0.7; 1.1] min (oe) |  |  |
|  |  |  |  |  |  |  |  |  |  |  |  |  |  |  |  |  |  |  |  |  |  |  | Walking: -0.3 [-1.2; 0.6] min (ue) |  |  |
| **Modus Health** | | | | | | | | | | | | | | | | | | | | | | | | |  |
| StepWatch 3 | | Accelerometer | Steps | | Amputation | | | Arch et al. (2018) | | CV | | DO | Ankle (a) | | N.R. | | | N.R. | | N.R. | | ICC | 6MWT: 0.99 [CI 0.98; 0.99] |  |  |
|  |  |  |  |  |  |  |  |  |  |  |  |  |  |  |  |  |  |  |  |  |  |  | FSST/F8WT: 0.90 [CI 0.75; 0.96] |  |  |
|  | |  |  | | Multiple (cardiovascular, musculoskeletal, neurological, cancer and others) | | | Treacy et al. (2017) | | CV | | DO | Ankle | | Prop | | | 3 sec | | N.R. | | ICC | ICC = 0.982 [CI 0.975; 0.986] |  |  |
|  | |  |  | |  | | | Webber & John (2016) | | CV | | DO | Ankle | | Prop | | | 3 sec | | N.R. | | ICC | ICC = 0.960 [CI 0.924; 0.979] |  |  |
|  | |  |  | | Stroke | | | Garcia Oliveira et al. (2021) | | CV | | DO | Ankle (la) | | Prop | | | N.R. | | N.R. | | Spearman's rho | Comfortable: rho = 0.963 |  |  |
|  |  |  |  |  |  |  |  |  |  |  |  |  |  |  |  |  |  |  |  |  |  |  | Fast: rho = 0.994 |  |  |
|  | |  |  | |  | | | Henderson et al. (2021) | | CV | | DO | Ankle (a) | | Prop | | | 60 sec | | N.R. | | ICC | PT session: ICC = 0.96 [CI 0.93; 0.98] |  |  |
|  |  |  |  |  |  |  |  |  |  |  |  |  |  |  |  |  |  |  |  |  |  |  | Walk training: ICC = 0.92 [CI 0.68; 0.96] |  |  |
|  | |  |  | |  | | |  | |  | |  | Ankle (ua) | | Prop | | | 60 sec | | N.R. | | ICC | PT session: ICC = 0.96 [CI 0.91; 0.99] |  |  |
|  |  |  |  |  |  |  |  |  |  |  |  |  |  |  |  |  |  |  |  |  |  |  | Walk training: ICC = 0.97 [CI 0.94; 0.98] |  |  |
|  | |  | Distance walked | | Peripheral artery disease | | | Taoum et al. (2020) | | CV | | GPS | Ankle | | Cust | | | 10 sec | | N.R. | | MAPE | 16.7 ± 10.7 |  |  |
| StepWatch 4 | | Accelerometer | Steps | | Amputation | | | Smith & Guerra (2021) | | CV | | DO | Ankle | | N.R. | | | N.R. | | N.R. | | ICC | ICC = 0.967 (CI 0.929 - 0.984) |  |  |
| **Body Media** | | | | | | | | | | | | | | | | | | | | | | | | |  |
| Sensewear armband | | Multisensor | EE | | Chronic lung disease | | | Dhillon et al. (2018) | | CV | | IC | U-arm | | Prop | | | N.R. | | N.R. | | Bland-Altman LoA | Flat walking: 0.36 [-2.36; 3.08] (oe) |  |  |
|  |  |  |  |  |  |  |  |  |  |  |  |  |  |  |  |  |  |  |  |  |  |  | Incline walking: 0.56 [-1.68; 2.80] (oe) |  |  |
|  |  |  |  |  |  |  |  |  |  |  |  |  |  |  |  |  |  |  |  |  |  |  | Sit to stand: --0.38 [-1.92; 1.16] (ue) |  |  |
|  |  |  |  |  |  |  |  |  |  |  |  |  |  |  |  |  |  |  |  |  |  |  | Lift bend: -0.39 [-3.25; 2.47] (ue) |  |  |
|  |  |  |  |  |  |  |  |  |  |  |  |  |  |  |  |  |  |  |  |  |  |  | Cycling (10%): -0.86 [-2.16; 0.44] (ue) |  |  |
|  |  |  |  |  |  |  |  |  |  |  |  |  |  |  |  |  |  |  |  |  |  |  | Cycling (25%): -0.70 [-2.75; 1.35] (ue) |  |  |
|  |  |  |  |  |  |  |  |  |  |  |  |  |  |  |  |  |  |  |  |  |  |  | Cycling (50%): -0.69 [-4.01; 2.63] (ue) |  |  |
|  |  |  |  |  |  |  |  |  |  |  |  |  |  |  |  |  |  |  |  |  |  |  | Cycling (60%): -1.26 [-4.71; 2.19] (ue) |  |  |
|  | |  |  | | MS | | | Stuart et al. (2020) | | Con v, conv | | Scale, functional | Arm | | Prop | | | N.R. | | N.R. | | Spearman's rho | Expanded Disability Status Scale: -0.411, p = .002 |  |  |
|  | |  |  | |  | | |  | |  | | Composite score, functional | Arm | | Prop | | | N.R. | | N.R. | | Spearman's rho | (MS functional Composite: 0.365, p = .006 |  |  |
|  | |  |  | |  | | |  | |  | | Test, functional ambulation | Arm | | Prop | | | N.R. | | N.R. | | Spearman's rho | Timed 25-foot walk: -0.412, p = .002 |  |  |
|  | |  |  | |  | | |  | |  | | Test, functional upperlimb | Arm | | Prop | | | N.R. | | N.R. | | Spearman's rho | 9-hole peg test: -0.254, p = .061 |  |  |
|  | |  |  | |  | | |  | |  | | SR, disease impact | Arm | | Prop | | | N.R. | | N.R. | | Spearman's rho | MS impact scale -phys: -0.287, p = .033 |  |  |
|  | |  |  | |  | | |  | | Con v, discr | | Test, cognitive | Arm | | Prop | | | N.R. | | N.R. | | Spearman's rho | Paced Auditory Serial Addition Test: 0.154, p = .261 |  |  |
|  | |  |  | |  | | |  | |  | | SR, disease impact | Arm | | Prop | | | N.R. | | N.R. | | Spearman's rho | MS impact scale - psych: -0.185, p = .177 |  |  |
|  | |  |  | |  | | |  | |  | | SR | Arm | | Prop | | | N.R. | | N.R. | | Spearman's rho | Fatigue score: -0.231, p = .090 |  |  |
|  | |  |  | |  | | |  | |  | | SR, congitive | Arm | | Prop | | | N.R. | | N.R. | | Spearman's rho | Beck depression invertory: 0.052, p = .707 |  |  |
|  | |  |  | |  | | |  | | Resp | | Scale, functional | Arm | | Prop | | | N.R. | | N.R. | | Spearman's rho | Expanded Disability Status Scale: -0.196, p = .187 (CS) |  |  |
|  | |  |  | |  | | |  | |  | | Composite score, functional | Arm | | Prop | | | N.R. | | N.R. | | Spearman's rho | (MS functional Composite: -0.09, p = .532 (CS) |  |  |
|  | |  |  | |  | | |  | |  | | Test, functional ambulation | Arm | | Prop | | | N.R. | | N.R. | | Spearman's rho | Timed 25-foot walk: 0.024, p = .867 (CS) |  |  |
|  | |  |  | |  | | |  | |  | | Test, functional upperlimb | Arm | | Prop | | | N.R. | | N.R. | | Spearman's rho | 9-hole peg test: 0.088, p = .543 (CS) |  |  |
|  | |  |  | |  | | |  | |  | | SR, disease impact | Arm | | Prop | | | N.R. | | N.R. | | Spearman's rho | MS impact scale -phys: 0.168, p = .243 (CS) |  |  |
|  | |  |  | |  | | |  | |  | | Test, cognitive | Arm | | Prop | | | N.R. | | N.R. | | Spearman's rho | Paced Auditory Serial Addition Test: 0.140, p = .332 (CS) |  |  |
|  | |  |  | |  | | |  | |  | | SR, disease impact | Arm | | Prop | | | N.R. | | N.R. | | Spearman's rho | MS impact scale - psych: 0.154, p = .284 (CS) |  |  |
|  | |  |  | |  | | |  | |  | | SR | Arm | | Prop | | | N.R. | | N.R. | | Spearman's rho | Fatigue score: 0.098, p = .498 (CS) |  |  |
|  | |  |  | |  | | |  | |  | | SR, congitive | Arm | | Prop | | | N.R. | | N.R. | | Spearman's rho | Beck depression invertory: 0.075, p = .608 (CS) |  |  |
|  | |  |  | | Stroke | | | Compagnat et al. (2019b) | | CV | | IC | Arm (h) | | Prop | | | 1 sec | | N.R. | | Pearson’s r | Circuit: r = 0.60 |  |  |
|  |  |  |  |  |  |  |  |  |  |  |  |  |  |  |  |  |  |  |  |  |  |  | Transfers: r = 0.52 |  |  |
|  |  |  |  |  |  |  |  |  |  |  |  |  |  |  |  |  |  |  |  |  |  |  | Manual tasks: r = 0.69 |  |  |
|  |  |  |  |  |  |  |  |  |  |  |  |  |  |  |  |  |  |  |  |  |  |  | Walking: r = 0.48 |  |  |
|  |  |  |  |  |  |  |  |  |  |  |  |  |  |  |  |  |  |  |  |  |  |  | Stairs: r = 0.81 |  |  |
|  | |  |  | |  | | | Mandigout et al. (2017) | |  | |  | Arm (a) | | N.R. | | | N.R. | | N.R. | | Spearman’s rho | r = 0.61 |  |  |
|  | |  |  | |  | | |  | |  | |  | Arm (h) | | N.R. | | | N.R. | | N.R. | | Spearman’s rho | r = 0.45 |  |  |
|  | |  | Steps | | MS | | | Stuart et al. (2020) | | Con v, conv | | Scale, functional | Arm | | Prop | | | N.R. | | N.R. | | Spearman's rho | Expanded Disability Status Scale: -0.325, p = .016 |  |  |
|  | |  |  | |  | | |  | |  | | Composite score, functional | Arm | | Prop | | | N.R. | | N.R. | | Spearman's rho | (MS functional Composite: 0.305, p = .024 |  |  |
|  | |  |  | |  | | |  | |  | | Test, functional ambulation | Arm | | Prop | | | N.R. | | N.R. | | Spearman's rho | Timed 25-foot walk: -0.317, p = .018 |  |  |
|  | |  |  | |  | | |  | |  | | Test, functional upperlimb | Arm | | Prop | | | N.R. | | N.R. | | Spearman's rho | 9-hole peg test: -0.220, p = .106 |  |  |
|  | |  |  | |  | | |  | |  | | SR, disease impact | Arm | | Prop | | | N.R. | | N.R. | | Spearman's rho | MS impact scale -phys: -0.278, p = .040 |  |  |
|  | |  |  | |  | | |  | | Con v, discr | | Test, cognitive | Arm | | Prop | | | N.R. | | N.R. | | Spearman's rho | Paced Auditory Serial Addition Test: 0.126, p = .359 |  |  |
|  | |  |  | |  | | |  | |  | | SR, disease impact | Arm | | Prop | | | N.R. | | N.R. | | Spearman's rho | MS impact scale - psych: -0.119, p = .359 |  |  |
|  | |  |  | |  | | |  | |  | | SR | Arm | | Prop | | | N.R. | | N.R. | | Spearman's rho | Fatigue score: -0.153, p = .265 |  |  |
|  | |  |  | |  | | |  | |  | | SR, congitive | Arm | | Prop | | | N.R. | | N.R. | | Spearman's rho | Beck depression invertory: -0.043, p = .758 |  |  |
|  | |  |  | |  | | |  | | Resp | | Scale, functional | Arm | | Prop | | | N.R. | | N.R. | | Spearman's rho | Expanded Disability Status Scale: 0.005, p = .975 (CS) |  |  |
|  | |  |  | |  | | |  | |  | | Composite score, functional | Arm | | Prop | | | N.R. | | N.R. | | Spearman's rho | (MS functional Composite: -0.17, p = .238 (CS) |  |  |
|  | |  |  | |  | | |  | |  | | Test, functional ambulation | Arm | | Prop | | | N.R. | | N.R. | | Spearman's rho | Timed 25-foot walk: 0.137, p = .343 (CS) |  |  |
|  | |  |  | |  | | |  | |  | | Test, functional upperlimb | Arm | | Prop | | | N.R. | | N.R. | | Spearman's rho | 9-hole peg test: 0.203, p = .157 (CS) |  |  |
|  | |  |  | |  | | |  | |  | | SR, disease impact | Arm | | Prop | | | N.R. | | N.R. | | Spearman's rho | MS impact scale -phys: 0.182, p = .207 (CS) |  |  |
|  | |  |  | |  | | |  | |  | | Test, cognitive | Arm | | Prop | | | N.R. | | N.R. | | Spearman's rho | Paced Auditory Serial Addition Test: 0.165, p = .253 (CS) |  |  |
|  | |  |  | |  | | |  | |  | | SR, disease impact | Arm | | Prop | | | N.R. | | N.R. | | Spearman's rho | MS impact scale - psych: 0.186, p = .195 (CS) |  |  |
|  | |  |  | |  | | |  | |  | | SR | Arm | | Prop | | | N.R. | | N.R. | | Spearman's rho | Fatigue score: 0.057, p = .693 (CS) |  |  |
|  | |  |  | |  | | |  | |  | | SR, congitive | Arm | | Prop | | | N.R. | | N.R. | | Spearman's rho | Beck depression invertory: 0.250, p = .083 (CS) |  |  |
|  | |  | Activity time | | MS | | | Stuart et al. (2020) | | Con v, conv | | Scale, functional | Arm | | Prop | | | N.R. | | N.R. | | Spearman's rho | Expanded Disability Status Scale: -0.589, p < .0001 |  |  |
|  | |  |  | |  | | |  | |  | | Composite score, functional | Arm | | Prop | | | N.R. | | N.R. | | Spearman's rho | (MS functional Composite: 0.493, p = .0001 |  |  |
|  | |  |  | |  | | |  | |  | | Test, functional ambulation | Arm | | Prop | | | N.R. | | N.R. | | Spearman's rho | Timed 25-foot walk: -0.640, p < .0001 |  |  |
|  | |  |  | |  | | |  | |  | | Test, functional upperlimb | Arm | | Prop | | | N.R. | | N.R. | | Spearman's rho | 9-hole peg test: -0.436, p = .001 |  |  |
|  | |  |  | |  | | |  | |  | | SR, disease impact | Arm | | Prop | | | N.R. | | N.R. | | Spearman's rho | MS impact scale -phys: -0.447, p = .001 |  |  |
|  | |  |  | |  | | |  | | Con v, discr | | Test, cognitive | Arm | | Prop | | | N.R. | | N.R. | | Spearman's rho | Paced Auditory Serial Addition Test: 0.134, p = .329 |  |  |
|  | |  |  | |  | | |  | |  | | SR, disease impact | Arm | | Prop | | | N.R. | | N.R. | | Spearman's rho | MS impact scale - psych: -0.234, p = .085 |  |  |
|  | |  |  | |  | | |  | |  | | SR | Arm | | Prop | | | N.R. | | N.R. | | Spearman's rho | Fatigue score: -0.203, p = .138 |  |  |
|  | |  |  | |  | | |  | |  | | SR, congitive | Arm | | Prop | | | N.R. | | N.R. | | Spearman's rho | Beck depression invertory: -0.179, p = .195 |  |  |
|  | |  |  | |  | | |  | | Resp | | Scale, functional | Arm | | Prop | | | N.R. | | N.R. | | Spearman's rho | Expanded Disability Status Scale: -0.128, p = .390 (CS) |  |  |
|  | |  |  | |  | | |  | |  | | Composite score, functional | Arm | | Prop | | | N.R. | | N.R. | | Spearman's rho | (MS functional Composite: -0.091, p = .531 (CS) |  |  |
|  | |  |  | |  | | |  | |  | | Test, functional ambulation | Arm | | Prop | | | N.R. | | N.R. | | Spearman's rho | Timed 25-foot walk: 0.076, p = .6 (CS) |  |  |
|  | |  |  | |  | | |  | |  | | Test, functional upperlimb | Arm | | Prop | | | N.R. | | N.R. | | Spearman's rho | 9-hole peg test: 0.093, p = .519 (CS) |  |  |
|  | |  |  | |  | | |  | |  | | SR, disease impact | Arm | | Prop | | | N.R. | | N.R. | | Spearman's rho | MS impact scale -phys: 0.014, p = .922 (CS) |  |  |
|  | |  |  | |  | | |  | |  | | Test, cognitive | Arm | | Prop | | | N.R. | | N.R. | | Spearman's rho | Paced Auditory Serial Addition Test: 0.178, p = .215 (CS) |  |  |
|  | |  |  | |  | | |  | |  | | SR, disease impact | Arm | | Prop | | | N.R. | | N.R. | | Spearman's rho | MS impact scale - psych: 0.272, p = .056 (CS) |  |  |
|  | |  |  | |  | | |  | |  | | SR | Arm | | Prop | | | N.R. | | N.R. | | Spearman's rho | Fatigue score: 0.013, p = .931 (CS) |  |  |
|  | |  |  | |  | | |  | |  | | SR, congitive | Arm | | Prop | | | N.R. | | N.R. | | Spearman's rho | Beck depression invertory: 0.246, p = .088 (CS) |  |  |
|  | |  | Distance walked | | Stroke | | | Compagnat et al. (2019a) | | CV | | DO | Arm (a) | | Prop | | | 1 sec | | N.R. | | Pearson’s r | r = 0.72 |  |  |
|  | |  |  | |  | | |  | |  | |  | Arm (h) | | Prop | | | 1 sec | | N.R. | | Pearson’s r | r = 0.68 |  |  |
|  | |  | Activity time | | MS | | | Stuart et al. (2020) | | Con v, conv | | Scale, functional | Arm | | Prop | | | N.R. | | N.R. | | Spearman's rho | Expanded Disability Status Scale: -0.589, p < .0001 |  |  |
|  | |  |  | |  | | |  | |  | | Composite score, functional | Arm | | Prop | | | N.R. | | N.R. | | Spearman's rho | (MS functional Composite: 0.493, p = .0001 |  |  |
|  | |  |  | |  | | |  | |  | | Test, functional ambulation | Arm | | Prop | | | N.R. | | N.R. | | Spearman's rho | Timed 25-foot walk: -0.640, p < .0001 |  |  |
|  | |  |  | |  | | |  | |  | | Test, functional upperlimb | Arm | | Prop | | | N.R. | | N.R. | | Spearman's rho | 9-hole peg test: -0.436, p = .001 |  |  |
|  | |  |  | |  | | |  | |  | | SR, disease impact | Arm | | Prop | | | N.R. | | N.R. | | Spearman's rho | MS impact scale -phys: -0.447, p = .001 |  |  |
|  | |  |  | |  | | |  | | Con v, discr | | Test, cognitive | Arm | | Prop | | | N.R. | | N.R. | | Spearman's rho | Paced Auditory Serial Addition Test: 0.134, p = .329 |  |  |
|  | |  |  | |  | | |  | |  | | SR, disease impact | Arm | | Prop | | | N.R. | | N.R. | | Spearman's rho | MS impact scale - psych: -0.234, p = .085 |  |  |
|  | |  |  | |  | | |  | |  | | SR | Arm | | Prop | | | N.R. | | N.R. | | Spearman's rho | Fatigue score: -0.203, p = .138 |  |  |
|  | |  |  | |  | | |  | |  | | SR, congitive | Arm | | Prop | | | N.R. | | N.R. | | Spearman's rho | Beck depression invertory: -0.179, p = .195 |  |  |
|  | |  |  | |  | | |  | | Resp | | Scale, functional | Arm | | Prop | | | N.R. | | N.R. | | Spearman's rho | Expanded Disability Status Scale: -0.128, p = .390 (CS) |  |  |
|  | |  |  | |  | | |  | |  | | Composite score, functional | Arm | | Prop | | | N.R. | | N.R. | | Spearman's rho | (MS functional Composite: -0.091, p = .531 (CS) |  |  |
|  | |  |  | |  | | |  | |  | | Test, functional ambulation | Arm | | Prop | | | N.R. | | N.R. | | Spearman's rho | Timed 25-foot walk: 0.076, p = .6 (CS) |  |  |
|  | |  |  | |  | | |  | |  | | Test, functional upperlimb | Arm | | Prop | | | N.R. | | N.R. | | Spearman's rho | 9-hole peg test: 0.093, p = .519 (CS) |  |  |
|  | |  |  | |  | | |  | |  | | SR, disease impact | Arm | | Prop | | | N.R. | | N.R. | | Spearman's rho | MS impact scale -phys: 0.014, p = .922 (CS) |  |  |
|  | |  |  | |  | | |  | |  | | Test, cognitive | Arm | | Prop | | | N.R. | | N.R. | | Spearman's rho | Paced Auditory Serial Addition Test: 0.178, p = .215 (CS) |  |  |
|  | |  |  | |  | | |  | |  | | SR, disease impact | Arm | | Prop | | | N.R. | | N.R. | | Spearman's rho | MS impact scale - psych: 0.272, p = .056 (CS) |  |  |
|  | |  |  | |  | | |  | |  | | SR | Arm | | Prop | | | N.R. | | N.R. | | Spearman's rho | Fatigue score: 0.013, p = .931 (CS) |  |  |
|  | |  |  | |  | | |  | |  | | SR, congitive | Arm | | Prop | | | N.R. | | N.R. | | Spearman's rho | Beck depression invertory: 0.246, p = .088 (CS) |  |  |
|  | |  | MET | | MS | | | Stuart et al. (2020) | | Con v, conv | | Scale, functional | Arm | | Prop | | | N.R. | | N.R. | | Spearman's rho | Expanded Disability Status Scale: -0.343, p = -.011 |  |  |
|  | |  |  | |  | | |  | |  | | Composite score, functional | Arm | | Prop | | | N.R. | | N.R. | | Spearman's rho | (MS functional Composite: 0.316, p = .019 |  |  |
|  | |  |  | |  | | |  | |  | | Test, functional ambulation | Arm | | Prop | | | N.R. | | N.R. | | Spearman's rho | Timed 25-foot walk: -0.331, p = .014 |  |  |
|  | |  |  | |  | | |  | |  | | Test, functional upperlimb | Arm | | Prop | | | N.R. | | N.R. | | Spearman's rho | 9-hole peg test: -0.212, p = .121 |  |  |
|  | |  |  | |  | | |  | |  | | SR, disease impact | Arm | | Prop | | | N.R. | | N.R. | | Spearman's rho | MS impact scale -phys: -0.299, p = .027 |  |  |
|  | |  |  | |  | | |  | | Con v, discr | | Test, cognitive | Arm | | Prop | | | N.R. | | N.R. | | Spearman's rho | Paced Auditory Serial Addition Test: 0.137, p = .318 |  |  |
|  | |  |  | |  | | |  | |  | | SR, disease impact | Arm | | Prop | | | N.R. | | N.R. | | Spearman's rho | MS impact scale - psych: -0.142, p = .303 |  |  |
|  | |  |  | |  | | |  | |  | | SR | Arm | | Prop | | | N.R. | | N.R. | | Spearman's rho | Fatigue score: -0.163 , p = .235 |  |  |
|  | |  |  | |  | | |  | |  | | SR, congitive | Arm | | Prop | | | N.R. | | N.R. | | Spearman's rho | Beck depression invertory: -0.108, p = .437 |  |  |
|  | |  |  | |  | | |  | | Resp | | Scale, functional | Arm | | Prop | | | N.R. | | N.R. | | Spearman's rho | Expanded Disability Status Scale: 0.065, p = .663 (CS) |  |  |
|  | |  |  | |  | | |  | |  | | Composite score, functional | Arm | | Prop | | | N.R. | | N.R. | | Spearman's rho | (MS functional Composite: -0.191, p = .184 (CS) |  |  |
|  | |  |  | |  | | |  | |  | | Test, functional ambulation | Arm | | Prop | | | N.R. | | N.R. | | Spearman's rho | Timed 25-foot walk: 0.155, p = .282 (CS) |  |  |
|  | |  |  | |  | | |  | |  | | Test, functional upperlimb | Arm | | Prop | | | N.R. | | N.R. | | Spearman's rho | 9-hole peg test: 0.178, p = .216 (CS) |  |  |
|  | |  |  | |  | | |  | |  | | SR, disease impact | Arm | | Prop | | | N.R. | | N.R. | | Spearman's rho | MS impact scale -phys: 0.197, p = .171 (CS) |  |  |
|  | |  |  | |  | | |  | |  | | Test, cognitive | Arm | | Prop | | | N.R. | | N.R. | | Spearman's rho | Paced Auditory Serial Addition Test: 0.140, p = .333 (CS) |  |  |
|  | |  |  | |  | | |  | |  | | SR, disease impact | Arm | | Prop | | | N.R. | | N.R. | | Spearman's rho | MS impact scale - psych: 0.226, p = .114 (CS) |  |  |
|  | |  |  | |  | | |  | |  | | SR | Arm | | Prop | | | N.R. | | N.R. | | Spearman's rho | Fatigue score: 0.046, p = .749 (CS) |  |  |
|  | |  |  | |  | | |  | |  | | SR, congitive | Arm | | Prop | | | N.R. | | N.R. | | Spearman's rho | Beck depression invertory: 0.295, p = .039 (CS) |  |  |
|  |  | PA composite score | | MS | | Stuart et al. (2020) | Con v, conv | | Scale, functional | | Arm | | | Prop | | N.R. | N.R. | | Spearman's rho | | Expanded Disability Status Scale: -0.419, p = .002 | | | | |
|  | |  |  | |  | | |  | |  | | Composite score, functional | Arm | | Prop | | | N.R. | | N.R. | | Spearman's rho | (MS functional Composite: 0.376, p = .005 |  |  |
|  | |  |  | |  | | |  | |  | | Test, functional ambulation | Arm | | Prop | | | N.R. | | N.R. | | Spearman's rho | Timed 25-foot walk: -0.444, p = .001 |  |  |
|  | |  |  | |  | | |  | |  | | Test, functional upperlimb | Arm | | Prop | | | N.R. | | N.R. | | Spearman's rho | 9-hole peg test: -0.321, p = .017 |  |  |
|  | |  |  | |  | | |  | |  | | SR, disease impact | Arm | | Prop | | | N.R. | | N.R. | | Spearman's rho | MS impact scale -phys: -0.356, p = .008 |  |  |
|  | |  |  | |  | | |  | | Con v, discr | | Test, cognitive | Arm | | Prop | | | N.R. | | N.R. | | Spearman's rho | Paced Auditory Serial Addition Test: 0.105, p = .444 |  |  |
|  | |  |  | |  | | |  | |  | | SR, disease impact | Arm | | Prop | | | N.R. | | N.R. | | Spearman's rho | MS impact scale - psych: -0.143, p = .297 |  |  |
|  | |  |  | |  | | |  | |  | | SR | Arm | | Prop | | | N.R. | | N.R. | | Spearman's rho | Fatigue score: -0.140, p = .307 |  |  |
|  | |  |  | |  | | |  | |  | | SR, congitive | Arm | | Prop | | | N.R. | | N.R. | | Spearman's rho | Beck depression invertory: -0.112, p = .422 |  |  |
|  | |  |  | |  | | |  | | Resp | | Scale, functional | Arm | | Prop | | | N.R. | | N.R. | | Spearman's rho | Expanded Disability Status Scale: -0.032, p = .833 (CS) |  |  |
|  | |  |  | |  | | |  | |  | | Composite score, functional | Arm | | Prop | | | N.R. | | N.R. | | Spearman's rho | (MS functional Composite: -0.11, p = .449 (CS) |  |  |
|  | |  |  | |  | | |  | |  | | Test, functional ambulation | Arm | | Prop | | | N.R. | | N.R. | | Spearman's rho | Timed 25-foot walk: 0.007, p = .964 (CS) |  |  |
|  | |  |  | |  | | |  | |  | | Test, functional upperlimb | Arm | | Prop | | | N.R. | | N.R. | | Spearman's rho | 9-hole peg test: 0.124, p = .391 (CS) |  |  |
|  | |  |  | |  | | |  | |  | | SR, disease impact | Arm | | Prop | | | N.R. | | N.R. | | Spearman's rho | MS impact scale -phys: 0.129, p = .374 (CS) |  |  |
|  | |  |  | |  | | |  | |  | | Test, cognitive | Arm | | Prop | | | N.R. | | N.R. | | Spearman's rho | Paced Auditory Serial Addition Test: 0.176, p = .221 (CS) |  |  |
|  | |  |  | |  | | |  | |  | | SR, disease impact | Arm | | Prop | | | N.R. | | N.R. | | Spearman's rho | MS impact scale - psych: 0.306, p = .031 (CS) |  |  |
|  | |  |  | |  | | |  | |  | | SR | Arm | | Prop | | | N.R. | | N.R. | | Spearman's rho | Fatigue score: 0.108, p = .456 (CS) |  |  |
|  | |  |  | |  | | |  | |  | | SR, congitive | Arm | | Prop | | | N.R. | | N.R. | | Spearman's rho | Beck depression invertory: 0.356, p = .012 (CS) |  |  |
| Sensewear Pro2 | | Multisensor | Steps | | Stroke | | | Mahendran et al. (2016) | | CV | | DO | U-arm (a) | | N.R. | | | 60 sec | | N.R. | | Absolute % error | T slow: 66.8% |  |  |
|  |  |  |  |  |  |  |  |  |  |  |  |  |  |  |  |  |  |  |  |  |  |  | T comf: 62.5% |  |  |
|  |  |  |  |  |  |  |  |  |  |  |  |  |  |  |  |  |  |  |  |  |  |  | T fast: 53.1% |  |  |
|  |  |  |  |  |  |  |  |  |  |  |  |  |  |  |  |  |  |  |  |  |  |  | 6MWT: 21.9% |  |  |
|  |  |  |  |  |  |  |  |  |  |  |  |  |  |  |  |  |  |  |  |  |  |  | Circuit: 40.1% |  |  |
|  | |  |  | |  | | |  | | TRT R | |  | U-arm (a) | | N.R. | | | 60 sec | | N.R. | | Absolute % error | T slow: 2.2% |  |  |
|  |  |  |  |  |  |  |  |  |  |  |  |  |  |  |  |  |  |  |  |  |  |  | T comf: 15.5% |  |  |
|  |  |  |  |  |  |  |  |  |  |  |  |  |  |  |  |  |  |  |  |  |  |  | T fast: 16.3% |  |  |
|  |  |  |  |  |  |  |  |  |  |  |  |  |  |  |  |  |  |  |  |  |  |  | 6MWT: 34.5% |  |  |
|  |  |  |  |  |  |  |  |  |  |  |  |  |  |  |  |  |  |  |  |  |  |  | Circuit: 38.5% |  |  |
|  | |  | MET | | Stroke | | | Mahendran et al. (2016) | | TRT R | |  | U-arm (a) | | N.R. | | | 60 sec | | N.R. | | Absolute % error | T slow: 17.8% |  |  |
|  |  |  |  |  |  |  |  |  |  |  |  |  |  |  |  |  |  |  |  |  |  |  | T comf: 24.1% |  |  |
|  |  |  |  |  |  |  |  |  |  |  |  |  |  |  |  |  |  |  |  |  |  |  | T fast: 26.8% |  |  |
|  |  |  |  |  |  |  |  |  |  |  |  |  |  |  |  |  |  |  |  |  |  |  | 6MWT: 23.1% |  |  |
|  |  |  |  |  |  |  |  |  |  |  |  |  |  |  |  |  |  |  |  |  |  |  | Circuit: 20.3% |  |  |
| **Activ8** | | | | | | | | | | | | | | | | | | | | | | | | |  |
| Activ8 | | Accelerometer | Activity time | | CP | | | Claridge et al. (2019) | | CV | | DO | Thigh (frontal) (la) | | N.R. | | | 5 sec | | 12.5 Hz | | Spearman’s rhoho | Sitting: ρ = 0.86 |  |  |
|  |  |  |  |  |  |  |  |  |  |  |  |  |  |  |  |  |  |  |  |  |  |  | Standing: ρ = -0.04 |  |  |
|  |  |  |  |  |  |  |  |  |  |  |  |  |  |  |  |  |  |  |  |  |  |  | Walking: ρ = 0.59 |  |  |
|  |  |  |  |  |  |  |  |  |  |  |  |  |  |  |  |  |  |  |  |  |  |  | Bicycling: ρ = 0.35 |  |  |
|  |  |  |  |  |  |  |  |  |  |  |  |  |  |  |  |  |  |  |  |  |  |  | Running: ρ = 0.77 |  |  |
|  |  |  |  |  |  |  |  |  |  |  |  |  |  |  |  |  |  |  |  |  |  |  | Upright activity: ρ = 0.72 |  |  |
|  | |  |  | |  | | |  | |  | |  | Thigh (lateral 2cm) (la) | | N.R. | | | 5 sec | | 12.5 Hz | | Spearman’s rhoho | Sitting: ρ = 0.98 |  |  |
|  |  |  |  |  |  |  |  |  |  |  |  |  |  |  |  |  |  |  |  |  |  |  | Standing: ρ = 0.93 |  |  |
|  |  |  |  |  |  |  |  |  |  |  |  |  |  |  |  |  |  |  |  |  |  |  | Walking: ρ = 0.94 |  |  |
|  |  |  |  |  |  |  |  |  |  |  |  |  |  |  |  |  |  |  |  |  |  |  | Bicycling: ρ = 0.49 |  |  |
|  |  |  |  |  |  |  |  |  |  |  |  |  |  |  |  |  |  |  |  |  |  |  | Running: ρ = 0.73 |  |  |
|  |  |  |  |  |  |  |  |  |  |  |  |  |  |  |  |  |  |  |  |  |  |  | Upright activity: ρ = 0.99 |  |  |
|  | |  |  | |  | | |  | |  | |  | Pocket | | N.R. | | | 5 sec | | 12.5 Hz | | Spearman’s rhoho | Sitting: ρ = 0.48 |  |  |
|  |  |  |  |  |  |  |  |  |  |  |  |  |  |  |  |  |  |  |  |  |  |  | Standing: ρ = 0.14 |  |  |
|  |  |  |  |  |  |  |  |  |  |  |  |  |  |  |  |  |  |  |  |  |  |  | Walking: ρ = 0.37 |  |  |
|  |  |  |  |  |  |  |  |  |  |  |  |  |  |  |  |  |  |  |  |  |  |  | Bicycling: ρ = 0.39 |  |  |
|  |  |  |  |  |  |  |  |  |  |  |  |  |  |  |  |  |  |  |  |  |  |  | Running: ρ = 0.74 |  |  |
|  |  |  |  |  |  |  |  |  |  |  |  |  |  |  |  |  |  |  |  |  |  |  | Upright activity: ρ = 0.79 |  |  |
|  | |  |  | | Stroke | | | Fanchamps et al. (2018) | | CV | | DO | Thigh (frontal) | | N.R. | | | 5 sec | | 12.5 Hz | | % time difference | Upright: -3.8% |  |  |
|  |  |  |  |  |  |  |  |  |  |  |  |  |  |  |  |  |  |  |  |  |  |  | Lying/sitting: 4.5% |  |  |
|  |  |  |  |  |  |  |  |  |  |  |  |  |  |  |  |  |  |  |  |  |  |  | Cycling: 6.5% |  |  |
|  | |  | Steps | | Multi (cardiovascular, cancer, respiratory, musculoskeletal, neurological) | | | Ummels et al. (2018) | | CV | | DO | Pocket | | N.R. | | | N.R. | | N.R. | | Pearson’s r | r = 0.24 |  |  |
| **Philips** | | | | | | | | | | | | | | | | | | | | | | | | |  |
| Actical | | Accelerometer | EE | | Chronic lung disease | | | Dhillon et al. (2018) | | CV | | IC | Wrist | | Prop | | | N.R. | | N.R. | | Bland-Altman LoA | Flat walking: -1.26 [-2.66; 0.14] (ue) |  |  |
|  |  |  |  |  |  |  |  |  |  |  |  |  |  |  |  |  |  |  |  |  |  |  | Incline walking: -1.80 [-4.16; 0.56] (ue) |  |  |
|  |  |  |  |  |  |  |  |  |  |  |  |  |  |  |  |  |  |  |  |  |  |  | Sit to stand: -1.05 [-2.72; 0.62] (ue) |  |  |
|  |  |  |  |  |  |  |  |  |  |  |  |  |  |  |  |  |  |  |  |  |  |  | Lift bend: -0.78 [-1.60; 0.04] (ue) |  |  |
|  |  |  |  |  |  |  |  |  |  |  |  |  |  |  |  |  |  |  |  |  |  |  | Cycling (10%): -1.01 [-2.27; 0.25] (ue) |  |  |
|  |  |  |  |  |  |  |  |  |  |  |  |  |  |  |  |  |  |  |  |  |  |  | Cycling (25%): -1.59 [-2.83; -0.35] (ue) |  |  |
|  |  |  |  |  |  |  |  |  |  |  |  |  |  |  |  |  |  |  |  |  |  |  | Cycling (50%): -2.51 [-4.54; -0.48] (ue) |  |  |
|  |  |  |  |  |  |  |  |  |  |  |  |  |  |  |  |  |  |  |  |  |  |  | Cycling (60%): -3.36 [-6.36; -0.36] (ue) |  |  |
|  | |  |  | | Stroke | | | Mandigout et al. (2017) | | CV | | IC | Ankle (a) | | N.R. | | | N.R. | | N.R. | | Spearman’s rho | r = 0.30 |  |  |
|  | |  |  | |  | | |  | |  | |  | Ankle (h) | | N.R. | | | N.R. | | N.R. | | Spearman’s rho | r = 0.20 |  |  |
|  | |  |  | |  | | |  | |  | |  | Waist | | N.R. | | | N.R. | | N.R. | | Spearman’s rho | r = -0.01 |  |  |
|  | |  |  | |  | | |  | |  | |  | Wrist (a) | | N.R. | | | N.R. | | N.R. | | Spearman’s rho | r = -0.19 |  |  |
|  | |  |  | |  | | |  | |  | |  | Wrist (h) | | N.R. | | | N.R. | | N.R. | | Spearman’s rho | r = -0.27 |  |  |
|  | |  | Activity kilocounts | | SCI | | | Zbogar et al. (2016) | | TRT R | |  | Wrist | | N.R. | | | 15 sec | | 32 Hz | | Pearson’s r | r = 0.74 [CI 0.54; 0.86] |  |  |
|  | |  | Steps | | SCI | | | Zbogar et al. (2016) | | TRT R | |  | Waist | | N.R. | | | 15 sec | | 32 Hz | | Pearson’s r | r = 0.84 [CI 0.70; 0.92] |  |  |
| **Axivity** | | | | | | | | | | | | | | | | | | | | | | | | |  |
| AX3/AX6 | | Accelerometer | Steps | | Cardiac rehab | | | Femiano et al. (2022) | | CV | | DO | Wrist | | Cust - windowed peak detection | | | N.R. | | 50 Hz | | MAPE | Walking: 4.1 ± 3.7 (UE) |  |  |
|  |  |  |  |  |  |  |  |  |  |  |  |  |  |  |  |  |  |  |  |  |  |  | Running: 11.2 ± 4.5 (UE) |  |  |
|  |  |  |  |  |  |  |  |  |  |  |  |  |  |  |  |  |  |  |  |  |  |  | Nordic: 10.9 ± 7.1 (UE) |  |  |
|  |  |  |  |  |  |  |  |  |  |  |  |  |  |  |  |  |  |  |  |  |  |  | Stairs: 7.5 ± 5.7 (UE) |  |  |
|  |  |  |  |  |  |  |  |  |  |  |  |  |  |  |  |  |  |  |  |  |  |  | AM+W: 17.0 ± 13.0 (UE) |  |  |
|  |  |  |  |  |  |  |  |  |  |  |  |  |  |  |  |  |  |  |  |  |  |  | AM-W: 81.7 ± 76.0 (OE) |  |  |
|  | |  |  | |  | | |  | |  | |  |  | | Cust- Autocorrelation | | | N.R. | | 50 Hz | | MAPE | Walking: 5.8 ± 4.2 (UE) |  |  |
|  |  |  |  |  |  |  |  |  |  |  |  |  |  |  |  |  |  |  |  |  |  |  | Running: 8.7 ± 3.1 (UE) |  |  |
|  |  |  |  |  |  |  |  |  |  |  |  |  |  |  |  |  |  |  |  |  |  |  | Nordic: 5.2 ± 4.9 (OE) |  |  |
|  |  |  |  |  |  |  |  |  |  |  |  |  |  |  |  |  |  |  |  |  |  |  | Stairs: 6.4 ± 7.2 (UE) |  |  |
|  |  |  |  |  |  |  |  |  |  |  |  |  |  |  |  |  |  |  |  |  |  |  | AM+W: 24.3 ± 24.7 (OE) |  |  |
|  |  |  |  |  |  |  |  |  |  |  |  |  |  |  |  |  |  |  |  |  |  |  | AM-W: 143 ± 128.4 (OE) |  |  |
|  | |  |  | | Lumbar spinal stenosis | | | Gustafsson et al. (2022) | | CV | | DO | Lower back | | Prop | | | N.R. | | 100 Hz | | ICC | SPWT: ICC = 0.99 (0.99; 1.00) |  |  |
|  |  |  |  |  |  |  |  |  |  |  |  |  |  |  |  |  |  |  |  |  |  |  | Rollator: ICC = -0.10 (-00.15; 0.18) |  |  |
|  |  |  |  |  |  |  |  |  |  |  |  |  |  |  |  |  |  |  |  |  |  |  | Crutch: ICC = 0.63 (0.35, 0.81) |  |  |
|  |  |  |  |  |  |  |  |  |  |  |  |  |  |  |  |  |  |  |  |  |  |  | Interval walking: ICC = 0.05 (-0.02, 0.23) |  |  |
|  | |  |  | |  | | |  | |  | |  | Thigh | | Prop | | | N.R. | | 100 Hz | | ICC | SPWT: ICC = 1.00 (1.00, 1.00) |  |  |
|  |  |  |  |  |  |  |  |  |  |  |  |  |  |  |  |  |  |  |  |  |  |  | Rollator: ICC = 0.66 (0.39, 0.83) |  |  |
|  |  |  |  |  |  |  |  |  |  |  |  |  |  |  |  |  |  |  |  |  |  |  | Crutch: ICC = 0.66 (0.38, 0.83) |  |  |
|  |  |  |  |  |  |  |  |  |  |  |  |  |  |  |  |  |  |  |  |  |  |  | Interval walking: ICC = -0.04 (-0.12, 0.12) |  |  |
|  | |  |  | |  | | |  | |  | |  | Waist | | Prop | | | N.R. | | 100 Hz | | ICC | SPWT: ICC = 1.00 (1.00, 1.00) |  |  |
|  |  |  |  |  |  |  |  |  |  |  |  |  |  |  |  |  |  |  |  |  |  |  | Rollator: ICC = 0.77 (0.57, 0.88) |  |  |
|  |  |  |  |  |  |  |  |  |  |  |  |  |  |  |  |  |  |  |  |  |  |  | Crutch: ICC = 0.77 (0.56, 0.88) |  |  |
|  |  |  |  |  |  |  |  |  |  |  |  |  |  |  |  |  |  |  |  |  |  |  | Interval walking: ICC = -0.10 (-0.22, 0.18) |  |  |
|  | |  |  | |  | | |  | |  | |  | Wrist | | Prop | | | N.R. | | 100 Hz | | ICC | SPWT: ICC = 1.00 (0.99, 1.00) |  |  |
|  |  |  |  |  |  |  |  |  |  |  |  |  |  |  |  |  |  |  |  |  |  |  | Rollator: ICC = 0.72 (0.49, 0.86) |  |  |
|  |  |  |  |  |  |  |  |  |  |  |  |  |  |  |  |  |  |  |  |  |  |  | Crutch: ICC = 0.59 (0.29, 0.78) |  |  |
|  |  |  |  |  |  |  |  |  |  |  |  |  |  |  |  |  |  |  |  |  |  |  | Interval walking: ICC = -0.16 (-0.29, 0.21) |  |  |
| **McRoberts** | | | | | | | | | | | | | | | | | | | | | | | | |  |
| Dynaport | | IMU | EE | | Stroke | | | Daniel et al. (2022) | | CV | | IC | Lower back | | Prop | | | N.R. | | 100 Hz | | ICC | TEE: ICC = 0.94 [CI 0.86; 0.97] |  |  |
|  |  |  |  |  |  |  |  |  |  |  |  |  |  |  |  |  |  |  |  |  |  |  | AEE: ICC = 0.77 [CI 0.50; 0.90] |  |  |
| Dynaport Hybrid | | IMU | Steps | | Parkinson's disease | | | Pham et al. (2017) | | CV | | DO | Lower back | | Cust. | | | N.R. | | 100 Hz | | Kappa | Turning episodes: k = 0.70 |  |  |
| **Vandrico Inc.** | | | | | | | | | | | | | | | | | | | | | | | | |  |
| Metria-IH1 | | Accelerometer | EE | | iSCI | | | Jayaraman et al. (2016) | | CV | | IC | U-arm | | Prop | | | N.R. | | N.R. | | ANOVA (one-way) | Sed: p > 0.05 |  |  |
|  |  |  |  |  |  |  |  |  |  |  |  |  |  |  |  |  |  |  |  |  |  |  | Low: p > 0.05 |  |  |
|  |  |  |  |  |  |  |  |  |  |  |  |  |  |  |  |  |  |  |  |  |  |  | High: p > 0.05 |  |  |
|  | |  |  | |  | | | Jayaraman et al. (2018) | | CV | | IC | U-arm | | Prop | | | 5000 points/min | | 32 Hz | | Kruskal wallis (Games-Howell post hoc) | Lying: mean diff: -0.27, p = .764 |  |  |
|  |  |  |  |  |  |  |  |  |  |  |  |  |  |  |  |  |  |  |  |  |  |  | Sitting: mean diff = -0.44, p = .302 |  |  |
|  |  |  |  |  |  |  |  |  |  |  |  |  |  |  |  |  |  |  |  |  |  |  | Standing: mean diff = -0.44, p = .800 |  |  |
|  |  |  |  |  |  |  |  |  |  |  |  |  |  |  |  |  |  |  |  |  |  |  | 50 SWT: mean diff = 0.81, p = .899 |  |  |
|  |  |  |  |  |  |  |  |  |  |  |  |  |  |  |  |  |  |  |  |  |  |  | 6 MWT: mean diff = -0.39, p = .997 |  |  |
|  |  |  |  |  |  |  |  |  |  |  |  |  |  |  |  |  |  |  |  |  |  |  | Sit-to-stand: mean diff = 0.49, p = .979 |  |  |
|  | |  |  | | Stroke | | | Jayaraman et al. (2018) | | CV | | IC | U-arm (a) | | Prop | | | 5000 points/min | | 32 Hz | | Kruskal wallis (Games-Howell post hoc) | Lying: mean diff: -0.06, p = .998 |  |  |
|  |  |  |  |  |  |  |  |  |  |  |  |  |  |  |  |  |  |  |  |  |  |  | Sitting: mean diff = -0.33, p = .296 |  |  |
|  |  |  |  |  |  |  |  |  |  |  |  |  |  |  |  |  |  |  |  |  |  |  | Standing: mean diff = -0.16, p = .950 |  |  |
|  |  |  |  |  |  |  |  |  |  |  |  |  |  |  |  |  |  |  |  |  |  |  | 50 SWT: mean diff = 1.01, p = .162 |  |  |
|  |  |  |  |  |  |  |  |  |  |  |  |  |  |  |  |  |  |  |  |  |  |  | 6 MWT: mean diff = 0.44, p = .999 |  |  |
|  |  |  |  |  |  |  |  |  |  |  |  |  |  |  |  |  |  |  |  |  |  |  | Sit-to-stand: mean diff = 2.92, p = .104 |  |  |
|  | |  |  | |  | | |  | |  | |  | U-arm (ua) | | Prop | | | 5000 points/min | | 32 Hz | | Kruskal wallis (Games-Howell post hoc) | Lying: mean diff = 0.06, p = .999 |  |  |
|  |  |  |  |  |  |  |  |  |  |  |  |  |  |  |  |  |  |  |  |  |  |  | Sitting: mean diff = -0.38, p = .227 |  |  |
|  |  |  |  |  |  |  |  |  |  |  |  |  |  |  |  |  |  |  |  |  |  |  | Standing: mean diff = -0.17, p = .940 |  |  |
|  |  |  |  |  |  |  |  |  |  |  |  |  |  |  |  |  |  |  |  |  |  |  | 50 SWT: mean diff = 0.84, p = .404 |  |  |
|  |  |  |  |  |  |  |  |  |  |  |  |  |  |  |  |  |  |  |  |  |  |  | 6 MWT: mean diff = 1.71, p = .299 |  |  |
|  |  |  |  |  |  |  |  |  |  |  |  |  |  |  |  |  |  |  |  |  |  |  | Sit-to-stand: mean diff = 2.65, p = .021 |  |  |
|  | |  | Steps | | iSCI | | | Jayaraman et al. (2016) | | CV | | DO | U-arm | | Prop | | | N.R. | | N.R. | | ANOVA (one-way) | p < 0.05 |  |  |
|  | |  | MET | | iSCI | | | Jayaraman et al. (2018) | | CV | | IC | U-arm | | Prop | | | 5000 points/min | | 32 Hz | | Kruskal wallis (Games-Howell post hoc) | Lying: mean diff: -0.06, p = .956 |  |  |
|  |  |  |  |  |  |  |  |  |  |  |  |  |  |  |  |  |  |  |  |  |  |  | Sitting: mean diff = -0.18, p = .281 |  |  |
|  |  |  |  |  |  |  |  |  |  |  |  |  |  |  |  |  |  |  |  |  |  |  | Standing: mean diff = -0.16, p = .891 |  |  |
|  |  |  |  |  |  |  |  |  |  |  |  |  |  |  |  |  |  |  |  |  |  |  | 50 SWT: mean diff = 0.72, p = .506 |  |  |
|  |  |  |  |  |  |  |  |  |  |  |  |  |  |  |  |  |  |  |  |  |  |  | 6 MWT: mean diff = -0.01, p = 1.000 |  |  |
|  |  |  |  |  |  |  |  |  |  |  |  |  |  |  |  |  |  |  |  |  |  |  | Sit-to-stand: mean diff = 0.76, p = .142 |  |  |
|  | |  |  | | Stroke | | | Jayaraman et al. (2018) | | CV | | IC | U-arm (a) | | Prop | | | 5000 points/min | | 32 Hz | | Kruskal wallis (Games-Howell post hoc) | Lying: mean diff: -0.06, p = .92 |  |  |
|  |  |  |  |  |  |  |  |  |  |  |  |  |  |  |  |  |  |  |  |  |  |  | Sitting: mean diff = -0.24, p = .08 |  |  |
|  |  |  |  |  |  |  |  |  |  |  |  |  |  |  |  |  |  |  |  |  |  |  | Standing: mean diff = -0.12, p = .73 |  |  |
|  |  |  |  |  |  |  |  |  |  |  |  |  |  |  |  |  |  |  |  |  |  |  | 50 SWT: mean diff = 0.65, p = ..004 |  |  |
|  |  |  |  |  |  |  |  |  |  |  |  |  |  |  |  |  |  |  |  |  |  |  | 6 MWT: mean diff = 0.03, p = 1.00 |  |  |
|  |  |  |  |  |  |  |  |  |  |  |  |  |  |  |  |  |  |  |  |  |  |  | Sit-to-stand: mean diff = 1.78, p = .02 |  |  |
|  | |  |  | |  | | |  | |  | |  | U-arm (ua) | | Prop | | | 5000 points/min | | 32 Hz | | Kruskal wallis (Games-Howell post hoc) | Lying: mean diff = 0.02, p = 1.00 |  |  |
|  |  |  |  |  |  |  |  |  |  |  |  |  |  |  |  |  |  |  |  |  |  |  | Sitting: mean diff = -0.28, p = .68 |  |  |
|  |  |  |  |  |  |  |  |  |  |  |  |  |  |  |  |  |  |  |  |  |  |  | Standing: mean diff = -0.14, p = .68 |  |  |
|  |  |  |  |  |  |  |  |  |  |  |  |  |  |  |  |  |  |  |  |  |  |  | 50 SWT: mean diff = 0.61, p = .32 |  |  |
|  |  |  |  |  |  |  |  |  |  |  |  |  |  |  |  |  |  |  |  |  |  |  | 6 MWT: mean diff = 0.95, p = .15 |  |  |
|  |  |  |  |  |  |  |  |  |  |  |  |  |  |  |  |  |  |  |  |  |  |  | Sit-to-stand: mean diff = 1.79, p = .001 |  |  |
| **Activinsight** | | | | | | | | | | | | | | | | | | | | | | | | |  |
| GENEactive | | Accelerometer | Raw acceleration (ENMO - mg) | | Myotonic dystrophy type 1 | | | Jimenez-Moreno et al (2019) | | TRT R | |  | Wrist & Ankle | | N.A. | | | 1 sec | | N.R. | | ICC | 10 mWT: ICC = 0.86 (CI 0.74 - 0.93) |  |  |
|  | |  |  | |  | | |  | |  | |  |  | |  | | |  | |  | |  | 10 mW/RT: ICC = 0.96 (CI 0.93-0.98) |  |  |
|  | |  |  | |  | | |  | |  | |  |  | |  | | |  | |  | |  | 6 MWT: ICC = 0.97 (CI 0.95-0.99) |  |  |
| **CamNtech** | | | | | | | | | | | | | | | | | | | | | | | | |  |
| Actiheart | | Multisensor | EE | | Amputation | | | Ladlow et al. (2019) | | CV | | IC | Chest | | Branched-Model eqation | | | 30 sec | | 32 Hz | | Pearson’s r | Unilateral: r = 0.86 |  |  |
|  |  |  |  |  |  |  |  |  |  |  |  |  |  |  |  |  |  |  |  |  |  |  | Bilateral: r = 0.81 |  |  |
| **Espruino** | |  |  | |  | | |  | |  | |  |  | |  | | |  | |  | |  |  |  |  |
| Bangle.js | | Smart watch (open source) | Steps | | DM | | | Van Laerhoven et al. (2022) | | CV | | Acc | Wrist | | Custom (open source) | | | N.R. | | N.R. | | LoA | Parameters all participants: -566.7 [-4111.5 - 2978.0] |  |  |
|  |  |  |  |  |  |  |  |  |  |  |  |  |  |  |  |  |  |  |  |  |  |  | Parameters per individual: 17.48 [-211.5 - 246.5] |  |  |
| **Garcia Oliveira et al.** | | | | | | | | | | | | | | | | | | | | | | | | |  |
| AMoR | | Gysroscopes + accelerometers | Steps | | Stroke | | | Garcia Oliveira et al. (2021) | | CV | | DO | Thigh | | N.R. | | | N.R. | | 80 fps | | ICC | Comfortable: ICC = 0.999 (CI 0.998 - 1.000) |  |  |
|  |  |  |  |  |  |  |  |  |  |  |  |  |  |  |  |  |  |  |  |  |  |  | Fast: ICC = 0.999 (CI 0.998 - 0.999) |  |  |
|  | |  |  | |  | | |  | |  | | Acc (SAM - ankle) | Thigh | | N.R. | | | N.R. | | 80 fps | | ICC | Comfortable: ICC = 0.985 (CI 0.973 - 0.991) |  |  |
|  |  |  |  |  |  |  |  |  |  |  |  |  |  |  |  |  |  |  |  |  |  |  | Fast: ICC = 0.981 (CI 0.967 - 0.989) |  |  |
|  | |  | Sedentary time | | Stroke | | | Garcia Oliveira et al. (2021) | | CV | | DO | Thigh | | N.R. | | | N.R. | | 80 fps | | ICC | ICC = 0.960 (CI 0.929 - 0.977) |  |  |
| **Maastricht Instruments BV** | | | | | | | | | | | | | | | | | | | | | | | | |  |
| MOX (1.01) | | Accelerometer | Counts | | Multi (COPD, Diabetes mellitus type 2) | | | Van der Weegen et al. (2015) | | CV | | Acc (AG GT3 - lower back) | Lower back | |  | | | 60 sec | | 25 Hz | | Pearson’s r | Lab: r = 0.98 (range 0.95 - 1.00) |  |  |
|  | |  |  | |  | | |  | |  | |  |  | |  | | |  | |  | | Spearman’s rho | Free living: r = 0.82 (range 0.60 - 0.94) |  |  |
|  | |  | Intensity time | | Multi (COPD, Diabetes mellitus type 2) | | | Van der Weegen et al. (2015) | | CV | | Acc (AG GT3 - lower back) | Lower back | |  | | | 60 sec | | 25 Hz | | Bland-Altman LoA | Mod: -2.3 [CI -27.6; 22.9] min |  |  |
|  |  |  |  |  |  |  |  |  |  |  |  |  |  |  |  |  |  |  |  |  |  |  | Vig: -0.5 [CI -9.5; 8.5] min |  |  |
|  |  |  |  |  |  |  |  |  |  |  |  |  |  |  |  |  |  |  |  |  |  |  | Non-turning episodes: k = 0.71 |  |  |
| **Medtronic** | | | | | | | | | | | | | | | | | | | | | | | | |  |
| ICD/CRT device | | Pacemaker (build-in accelerometer) | Activity time | | Heart failure | | | Shoemaker et al. (2017) | | CV | | Acc (AG GT3 - waist) | Chest (internal) | | Prop | | | N.R. | | N.R. | | Bland-Altman LoA | -0.77 [-2.71; 1.17] hours/day (ue) |  |  |
|  | |  |  | |  | | |  | | Resp | | Acc (AG GT3 - waist) | Chest (internal) | | Prop | | | N.R. | | N.R. | | Bland-Altman LoA | 0.19 [-0.79; 1.17] hours/day (oe) |  |  |
| **StepsCount** | | | | | | | | | | | | | | | | | | | | | | | | |  |
| PiezoRX | | Pedometer | Steps | | MS | | | Anens et al. (2023) | | CV | | DO | N.R. | | N.R. | | | N.R. | | N.R. | | Spearman's rho | Comfortable: rho = 0.82 |  |  |
|  |  |  |  |  |  |  |  |  |  |  |  |  |  |  |  |  |  |  |  |  |  |  | Fast: rho = 0.99 |  |  |
|  |  |  |  |  |  |  |  |  |  |  |  |  |  |  |  |  |  |  |  |  |  |  | Slow: rho = 0.91 |  |  |
|  |  |  |  |  |  |  |  |  |  |  |  |  |  |  |  |  |  |  |  |  |  |  | Total: rho = 0.92 |  |  |
| **Xsens** | | | | | | | | | | | | | | | | | | | | | | | | |  |
| MTw | | IMU | EE | | Diabetes mellitus type 2 | | | Caron et al. (2019) | | CV | | IC | Lower back | | Bouten's equation | | | 30 sec | | 75 Hz | | Bland-Altman LoA | -1.17 [-6.45; 4.14] W/kg (oe) |  |  |
| **ZurichMOVE** | | | | | | | | | | | | | | | | | | | | | | | | |  |
| JUMP | | Multisensor | EE | | iSCI | | | Popp et al (2019) | | CV | | IC | Multiple | | Cust (Multi-linear regression model with preceding activity classification and updated Harris-Benidict REE equation) | | | N.R. | | 200 Hz | | Pearson’s r | r = 0.92 |  |  |
|  |  |  |  |  |  |  |  |  |  |  |  |  | - Wrist (2) | |  |  |  |  |  |  |  |  |  |  |  |
|  |  |  |  |  |  |  |  |  |  |  |  |  | - Ankle (2) | |  |  |  |  |  |  |  |  |  |  |  |
|  |  |  |  |  |  |  |  |  |  |  |  |  | - Foot (2) | |  |  |  |  |  |  |  |  |  |  |  |
|  |  |  |  |  |  |  |  |  |  |  |  |  | - Chest (1) | |  |  |  |  |  |  |  |  |  |  |  |
|  |  |  |  |  |  |  |  |  |  |  |  |  | - Waist (1) | |  |  |  |  |  |  |  |  |  |  |  |

EE = energy expenditure, MET = metabolic equivalent, PAL = physical activity level

CAD = coronay artery disease, COPD = chronic obstructive pulmonary disease, DM = diabetes mellitus, iSCI = incomplete spinal cord injury, MS = multiple sclerosis, PAD = pulmonary artery disease, PD = Parkinson's disease, RA = rheumatoid arthritis, SCI = spinal cord injury

CV = Criterion validity, Con V = construct validity, Resp = responsiveness, TRT R = test-retest reliability

Acc = accelerometer, DLW = doubly labelled water, DO = direct observation, IC = indirect calorimetry, SR = self-report, Q = questionnaire, AG = ActiGraph

(a) = affected side, (b) = both affected and unaffected side, (la) = less affected side, (LRL) = longest residual limb, (SRL) = shortest residual limb, (ua) = unaffected side

Cust = custom algorithm, LFE = low frequency effect, N.R. = not reported, Prop = proprietary algorithm, TEE = total energy expenditure, BMR = basal metabolic rate

APE = absolute percentage error, CCC = concordance correlation coefficients, ICC = intra class correlation, LoA = limits of agreement, MAPE = mean absolute percentage error, MPE = mean percentage error

[CI] = 95% confidence intervals, (oe) = over estimation, (ue) = under estimation, MVPA = moderate to vigorous physical activity, MWT = minutes walking test, Sed = sedentary, STS = sit-to-stand test, SWT = steps walk test
