# Supplementary material for: Measurement properties of device-based physical activity instruments in ambulatory adults with physical disabilities and/or chronic diseases: a scoping review
Source: BMC Sports Sci Med Rehabil. 2023 Sep 21;15:115. doi: 10.1186/s13102-023-00717-0 (PMC10512652; doi:10.1186/s13102-023-00717-0)
Supplement: Supplementary file 6 — Additional file 6: Supplementary file 6. Expanded overview of consumer-grade devices evaluated on their measurement properties in 74 studies. An expanded overview of the consumer-grade devices evaluated on their measurement properties. Extra information on epoch length, sampling rate and results per condition. [file 13102_2023_717_MOESM6_ESM.docx]

# Supplementary file 6 – Expanded overview of consumer-grade devices evaluated on their measurement properties in 67 studies. Ordering on number of studies evaluating manufacturer.

|  |  |  |  |  |  |  |  |  |  |  | Result |  | |
| --- | --- | --- | --- | --- | --- | --- | --- | --- | --- | --- | --- | --- | --- |
| Type | Device type | PA outcome | Population | Study | Measurement property | Criterion | Placement | Algorithm | Epoch length | Sampling rate | Test | Outcome | |
| **Fitbit** | | | | | | | | | | | | |  |
| Alta | Activity tracker | Steps | Cancer | Rossi et al. (2018) | Con V | SR (Godin Leisure-Time Exercise) | N.R. | N.R. | N.R. | N.R. | CCC | CCC = 0.00005 [CI -0.22 - 0.22] | |
|  |  |  | COPD | Blondeel et al. (2020) | CV | Acc (Dynaport - Lower back) | Wrist | Prop | N.R. | N.R. | Bland-Altman LoA | 306 [-2068; 2680] (oe) | |
|  |  |  | MS | Lavelle et al. (2021) | CV | DO | Wrist | N.R. | N.R. | N.R. | Bland-Altman LoA | -302.8 [-1036.8, 431.1] (oe) | |
|  |  |  | Stroke | Holubova et al. (2022) | CV | DO | Upper limb (a) | Prop | N.R. | N.R. | MARD | No aid: 3.05 ± 4.12% | |
|  |  |  |  |  |  |  |  |  |  |  |  | Stick: 3.74 ± 6.17% | |
|  |  |  |  |  |  |  |  |  |  |  |  | Rollator: 85.07 ± 25.19% | |
|  |  |  |  |  |  |  | Upper limb (ua) | Prop | N.R. | N.R. | MARD | No aid: 5.25 ± 6.19 | |
|  |  |  |  |  |  |  |  |  |  |  |  | Stick: 9.84 ± 14.17 | |
|  |  |  |  |  |  |  |  |  |  |  |  | Rollator: 85.67 ± 33.18 | |
|  |  |  |  |  |  |  | Lower limb (a) | Prop | N.R. | N.R. | MARD | No aid: 6.25 ± 8.05 | |
|  |  |  |  |  |  |  |  |  |  |  |  | Stick: 11.08 ± 16.58 | |
|  |  |  |  |  |  |  |  |  |  |  |  | Rollator: 4.82 ± 14.08 | |
|  |  |  |  |  |  |  | Lower limb (ua) | Prop | N.R. | N.R. | MARD | No aid: 1.35 ± 1.34 | |
|  |  |  |  |  |  |  |  |  |  |  |  | Stick: 1.33 ± 0.86 | |
|  |  |  |  |  |  |  |  |  |  |  |  | Rollator: 10.82 ± 35.20 | |
|  |  |  |  |  |  |  | Waist | Prop | N.R. | N.R. | MARD | No aid: 0.47 ± 0.50 | |
|  |  |  |  |  |  |  |  |  |  |  |  | Stick: 3.66 ± 8.48 | |
|  |  |  |  |  |  |  |  |  |  |  |  | Rollator: 3.36 ± 576 | |
|  |  | Activity time | MS | Lavelle et al. (2021) | CV | Acc (AG GT3 - waist) |  | N.R. | N.R. | N.R. | % error | 100% [range -38.7 - 100] | |
| Charge | Activity tracker | Steps | Amputation | Smith et al. (2019) | CV | DO | Wrist | N.R. | N.R. | N.R. | ICC | ICC = 0.86 | |
|  |  |  | Multi (Musculoskeletal, neurological & other) | Treacy et al. (2017) | CV | DO | Wrist | N.R. | N.R. | N.R. | ICC | ICC = 0.399 [CI -0.026- 0.654] | |
|  |  |  | Parkinson's diseases | Lamont et al. (2018) | CV | Acc (ActivPal - Thigh) | Wrist | N.R. | N.R. | N.R. | ICC | Indoor self-selected pace: ICC = 0.88 [CI 0.76 - 0.94] | |
|  |  |  |  |  |  |  |  |  |  |  |  | Indoor 60 steps/min: ICC = 0.36 CI -0.29 - 0.69] | |
|  |  |  |  |  |  |  |  |  |  |  |  | Indoor 80 steps/min: ICC = 0.18 [CI -0.67 - 0.59] | |
|  |  |  |  |  |  |  |  |  |  |  |  | Indoor 100 steps/min: ICC = 0.17 [CI -0.72 - 0.60] | |
|  |  |  |  |  |  |  |  |  |  |  |  | Indoor 120 steps/min: ICC = 0.37 [CI-0.39 - 0.71] | |
|  |  |  |  |  |  |  |  |  |  |  |  | Indoor 140 steps/min: ICC = 0.42 [CI -0.18 - 0.72] | |
|  |  |  |  |  |  |  |  |  |  |  |  | Outdoor self-selected pace: 0.94 [CI 0.86 - 0.97] | |
| Charge 2 | Activity tracker | EE | Coronary artery disease | Herkert et al. (2019) | CV | IC | Wrist | Prop | N.R. | N.R. | ICC | ICC = 0.10 | |
|  |  |  | Heart failure | Herkert et al. (2019) | CV | IC | Wrist | Prop | N.R. | N.R. | ICC | ICC = 0.42 | |
|  |  | Steps | Heart failure | Vetrovsky et al. (2019) | CV | Acc (AG GT3 - waist) | Wrist | N.R. | N.R. | N.R. | CCC | CCC = 0.48 [CI 0.20 - 0.69] | |
|  |  |  | Osteoarthritis | Collins et al. (2019) | CV | Acc (AG GT3 - waist) | Wrist | Prop | 60 sec | N.R. | ICC | ICC = 0.602 | |
|  | | | Parkinson's diseases | Lai et al. (2020) | CV | DO | Wrist | N.R. | N.R. | N.R. | ICC | Overground: ICC = 0.47 [CI -0.57 - 0.74] | |
|  |  |  |  |  |  |  |  |  |  |  |  | Treadmill: ICC = 0.27 [CI -0.26 - 0.61] | |
|  |  |  | Progressive muscle diseases | Roberts-Lewis et al. (2022) | CV | DO | Wrist | N.R. | N.R. | N.R. | Spearman's rho | rho = 0.97 [CI 0.96 - 0.98] | |
|  |  | Intensity time | Osteoarthritis | Collins et al. (2019) | CV | Acc (AG GT3 - waist) | Wrist | Cust | N.R. | N.R. | % bias | Sed: 37% | |
|  |  |  |  |  |  |  |  | Cust | N.R. | N.R. | % bias | MVPA: -5% | |
|  |  | MET | Progressive muscle diseases | Roberts-Lewis et al. (2022) | CV | SR (RPE) | Wrist | N.R. | N.R. | N.R. | Kappa | k = 0.90 [CI 0.89 - 0.93] | |
| Flex | Activity tracker | Steps | Amputation | Smith et al. (2019) | CV | DO | Wrist | N.R. | N.R. | N.R. | ICC | ICC = 0.843 | |
|  |  |  | Coronary artery disease | Alharbi et al. (2016) | CV | Acc (AG GT3 - waist) | N.R. | Prop | 60 sec | N.R. | Pearson r | r = 0.947 | |
|  |  |  | Post heart operation | Daligadu et al. (2018) | CV | DO | Wrist | N.R. | N.R. | N.R. | CCC | CCC = 0.43 | |
|  |  |  | MS | Balto et al. (2016) | CV | DO | Wrist | N.R. | N.R. | N.R. | Mean percentage error | 1e trial: 13.8% | |
|  |  |  |  |  |  |  |  |  |  |  |  | 2e trial: 12.4% | |
|  |  |  |  | Block et al. (2017) | CV | DO + ACC (AG GT3 - N.R.) | Wrist | N.R. | N.R. | N.R. | ICC | 2MWT DO: ICC = 0.69 | |
|  |  |  |  |  |  |  |  |  |  |  |  | 2MWT ACC: ICC = 0.59 | |
|  |  |  |  | Block et al. (2017) | CV | ACC (AG GT3 - N.R.) | Wrist | N.R. | N.R. | N.R. | ICC | ICC = 0.74 | |
|  |  |  |  | Block et al. (2019) | CV | DO | Wrist | N.R. | N.R. | N.R. | ICC | ICC = 0.69 [CI 0.53 - 0.80] | |
|  |  |  |  | Block et al. (2019) | CV | ACC (AG GT3 - waist) | Wrist | N.R. | N.R. | N.R. | ICC | ICC = 0.98 [CI 0.97 - 0.98] | |
|  |  |  | Multi (cardiovascular, cancer, respiratory, musculoskeletal, neurological) | Ummels et al. (2018) | CV | DO | Wrist | N.R. | N.R. | N.R. | Pearson r | r = 0.31 | |
|  |  | Intensity time | Chronic knee symptoms | Semanik et al. (2020) | CV | Acc (AG GT3 - waist) | Wrist | Prop | N.R. | N.R. | Spearman r | Light: ICC = 0.60 [CI 0.34 - 0.78] | |
|  |  |  |  |  |  |  |  | - cut off light: 1.5-3.0 MET |  |  |  | Moderate: ICC = 0.52 [CI 0.22 - 0.73] | |
|  |  |  |  |  |  |  |  | - cut off moderate: 3.0-6.0 MET in 10min bouts |  |  |  | Vigorous: ICC = 0.25 [CI -0.09 - 0.54] | |
|  |  |  |  |  |  |  |  | - cut off vigorous: >6.0 MET in 10min bouts |  |  |  | MVPA: ICC = 0.73 [CI 0.52 - 0.85] | |
|  |  |  | Coronary artery disease | Alharbi et al. (2016) | CV | Acc (AG GT3 - waist) | N.R. | Prop | 60 sec | N.R. | Pearson r | Light: r = 0.722 | |
|  |  |  |  |  |  |  |  |  |  |  |  | Moderate: r = 0.670 | |
|  |  |  |  |  |  |  |  |  |  |  |  | Vigorous: r = 0.036 | |
|  |  |  |  |  |  |  |  |  |  |  |  | MVPA: r = 0.710 | |
|  |  |  | Stroke | Chow et al. (2023) | CV | Acc (GENEActive - wrist) | Wrist | Prop | N.R. | N.R. | ICC | Light PA: ICC = 0.884 (0.722 - 0.954) | |
|  |  |  |  |  |  |  |  |  |  |  |  | Sedentary time: ICC = -0.236 (-0.596 - 0.299) | |
|  |  | Distance walked | Post heart operation | Daligadu et al. (2018) | CV | DO | Wrist | N.R. | N.R. | N.R. | CCC | CCC = 0.37 | |
| Flex 2 | Activity tracker | Steps | MS | Block et al. (2019) | CV | Acc (AG GT3 - waist) | Wrist | N.R. | N.R. | N.R. | ICC | ICC = 0.98 [CI 0.97 - 0.99] | |
|  |  |  | Osteoarthritis | Yu et al. (2021) | CV | SR (IPAQ-SF) | Wrist | N.R. | N.R. | N.R. | Correlation | Baseline: 0.20 | |
|  |  |  |  |  |  |  |  |  |  |  |  | Follow-up: 0.28 | |
|  |  |  |  |  | Resp | SR (Global rating of change) | Wrist | N.R. | N.R. | N.R. | Correlation | 0.28 | |
|  |  |  |  |  |  | Test (6MWT) | Wrist | N.R. | N.R. | N.R. | Correlation | 0.21 | |
|  |  |  |  |  |  | Test (TUG) | Wrist | N.R. | N.R. | N.R. | Correlation | 0.06 | |
|  |  |  |  |  |  | SR (Intermittent and Constant Osteoarthritis Pain) | Wrist | N.R. | N.R. | N.R. | Correlation | Constant pain: -0.23 | |
|  |  |  |  |  |  |  |  |  |  |  |  | Intermittend pain: -0.18 | |
|  |  |  |  |  |  | SR (Knee Injury and Osteoarthritis Outcome Score) | Wrist | N.R. | N.R. | N.R. | Correlation | Pain: 0.08 | |
|  |  |  |  |  |  |  |  |  |  |  |  | ADL: 0.22 | |
|  |  |  |  |  |  |  |  |  |  |  |  | Symptoms: 0.11 | |
|  |  |  |  |  |  |  |  |  |  |  |  | QoL: 0.11 | |
|  |  |  |  |  |  |  |  |  |  |  |  | Sports: 0.13 | |
|  |  |  |  |  |  | SR (Western Ontario and McMaster Universities Osteoarthritis Index) | Wrist | N.R. | N.R. | N.R. | Correlation | Pain: -0.18 | |
|  |  |  |  |  |  |  |  |  |  |  |  | Function: -0.28 | |
|  |  |  |  |  |  |  |  |  |  |  |  | Stiffness: -0.12 | |
|  |  |  |  |  |  | SR (Knee Pain - 11 point NRS) | Wrist | N.R. | N.R. | N.R. | Correlation | Average pain: -0.11 | |
|  |  |  |  |  |  |  |  |  |  |  |  | Walking pain: -0.09 | |
| Inc. | Activity tracker | Steps | Parkinson's disease | de Carvalho Lana et al. (2021) | CV | DO | Waist | N.R. | N.R. | N.R. | Pearson r | r = 0.82 | |
| Inspire | Activity tracker | Steps | MS | Polhemus et al. (2023) | CV | DO | Wrist | Prop | N.R. | N.R. | CCC | CCC = 0.66 (CI 0.14 - 0.80) | |
|  |  |  |  |  |  | Acc (AG GT3 - waist) | Wrist | Prop | N.R. | N.R. | CCC | Scripted tasks: | |
|  |  |  |  |  |  |  |  |  |  |  |  | CCC = 0.55 (CI 0.08 - 0.72) AG prop | |
|  |  |  |  |  |  |  |  |  |  |  |  | CCC = 0.65 (CI 0.24 - 0.77) AG prop+LFE | |
|  |  |  |  |  |  |  |  |  |  |  |  | FL - daily level: | |
|  |  |  |  |  |  |  |  |  |  |  |  | CCC = 0.44 (CI 0.32 - 0.57) AG prop | |
|  |  |  |  |  |  |  |  |  |  |  |  | CCC = 0.33 (CI 0.22 - 0.43) AG prop+LFE | |
|  |  |  |  |  |  |  |  |  |  |  |  | FL - Acverage level: CCC = 0.65 (CI 0.47 - 0.77) AG prop CCC = 0.50 (CI 0.34 - 0.63) AG prop+LFE | |
|  |  |  | Progressive muscle diseases | Roberts-Lewis et al. (2022) | CV | Acc (GENEActive - wrist) | Wrist | N.R. | N.R. | N.R. | Spearman's rho | rho = 0.76 (CI 0.60 - 0.87) | |
|  |  |  |  |  | TRT R |  | Wrist | N.R. | N.R. | N.R. | ICC | ICC = 0.96 (CI 0.92 - 0.98) | |
|  |  |  |  |  | Resp | Acc (GENEActive - wrist) | Wrist | N.R. | N.R. | N.R. | AUC | AUC = 0.86 (CI 0.75 - 0.97) | |
|  |  | Activity time | MS | Polhemus et al. (2023) | CV | Acc (AG GT3 - waist) | Wrist | Prop | N.R. | N.R. | CCC | FL - daily level: | |
|  |  |  |  |  |  |  |  |  |  |  |  | CCC = 0.36 (CI 0.21 - 0.50) AG vertical>100cpm | |
|  |  |  |  |  |  |  |  |  |  |  |  | CCC = 0.18 (CI 0.22 - 0.43) AG VM >150cmp | |
|  |  |  |  |  |  |  |  |  |  |  |  | FL - Average level: | |
|  |  |  |  |  |  |  |  |  |  |  |  | CCC = 0.52 (CI 0.32 - 0.68) AG Vertical > 100cpm | |
|  |  |  |  |  |  |  |  |  |  |  |  | CCC = 0.35 (CI 0.20 - 0.49) AG VM > 150cpm | |
|  |  | Intensity time | MS | Polhemus et al. (2023) | CV | Acc (AG GT3 - waist) | Wrist | Prop | N.R. | N.R. | CCC | FL - daily level: | |
|  |  |  |  |  |  |  |  |  |  |  |  | CCC = 0.45 (CI 0.27 - 0.63) AG Uni | |
|  |  |  |  |  |  |  |  |  |  |  |  | CCC = 0.41 (CI 0.27 - 0.56) AG Severity | |
|  |  |  |  |  |  |  |  |  |  |  |  | CCC = 0.44 (CI 0.27 - 0.58) Saaki | |
|  |  |  |  |  |  |  |  |  |  |  |  | FL - Average level: | |
|  |  |  |  |  |  |  |  |  |  |  |  | CCC = 0.80 (CI 0.64 - 0.89) AG Uni | |
|  |  |  |  |  |  |  |  |  |  |  |  | CCC = 0.63 (CI 0.39 - 0.79) AG Severity | |
|  |  |  |  |  |  |  |  |  |  |  |  | CCC = 0.78 (CI 0.61 - 0.88) Saaki | |
|  |  |  | Progressive muscle diseases | Roberts-Lewis et al. (2022) | CV | Acc (GENEActive - wrist) | Wrist | N.R. | N.R. | N.R. | Spearman's rho | rho = 0.51 (CI 0.29 - 0.69) | |
|  |  |  |  |  | TRT R |  | Wrist | N.R. | N.R. | N.R. | ICC | ICC = 0.78 (CI 0.63 - 0.87) | |
|  |  |  |  |  | Resp | Acc (GENEActive - wrist) | Wrist | N.R. | N.R. | N.R. | AUC | AUC = 0.72 (CI 0.56 - 0.88) | |
|  |  | MET | Progressive muscle diseases | Roberts-Lewis et al. (2022) | CV | Acc (GENEActive - wrist) | Wrist | N.R. | N.R. | N.R. | Spearman's rho | rho = 0.63 (CI 0.47 - 0.74) | |
|  |  |  |  |  | TRT R |  | Wrist | N.R. | N.R. | N.R. | ICC | ICC = 0.94 (CI 0.89 - 0.97) | |
|  |  |  |  |  | Resp | Acc (GENEActive - wrist) | Wrist | N.R. | N.R. | N.R. | AUC | AUC = 0.90 (CI 0.81 - 0.98) | |
| One | Activity tracker | Steps | Amputation | Arch et al. (2018) | CV | DO | Ankle (a) | N.R. | N.R. | N.R. | ICC | 6MWT: ICC = 0.97 [CI 0.93 - 0.99] | |
|  |  |  |  |  |  |  |  |  |  |  |  | FSST/F8WT: ICC = 0.88 [CI 0.69-0.96] | |
|  |  |  | Cancer | Van Blarigan et al. (2017) | CV | Acc (AG GT3 - waist) | Waist | N.R. | N.R. | N.R. | Pearson r | r = 0.94 | |
|  |  |  |  |  |  | Acc (Omron - waist) | Waist | N.R. | N.R. | N.R. | Pearson r | r = 0.67 | |
|  |  |  | MS | Balto et al. (2016) | CV | DO | Waist | N.R. | N.R. | N.R. | Mean percentage error | 1e trial: 1.9% | |
|  |  |  |  |  |  |  |  |  |  |  |  | 2e trial: 1.9% | |
|  |  |  | Myositis | Saygin (2022) | CV | Acc (AG GT3 - waist) | Waist | Prop | N.R. | N.R. | ICC | ICC = 0.96 (CI 0.92 - 0.98) | |
|  |  |  |  |  | TRT R |  | Waist | Prop | N.R. | N.R. | ICC | ICC = 0.89 (CI 0.72 - 0.96) | |
|  |  |  |  |  | Resp | SR (total improvement score) | Waist | Prop | N.R. | N.R. | Spearman's rho | rho = 0.63 | |
|  |  |  | Multi (cardiovascular, cancer, respiratory, musculoskeletal, neurological, other) | Ummels et al. (2018) | CV | DO | Waist | N.R. | N.R. | N.R. | Pearson r | r = -0.15 | |
|  |  |  |  | Treacy et al. (2017) | CV | DO | Ankle | N.R. | N.R. | N.R. | ICC | ICC = 0.919 [CI 0.772 - 0.961] | |
|  |  |  |  |  |  |  | Waist | N.R. | N.R. | N.R. | ICC | ICC = 0.397 [CI -0.087 - 0.689] | |
|  |  |  | Parkinson's diseases | Lai et al. (2020) | CV | DO | Waist | N.R. |  |  | ICC | Overground: ICC = 0.98 [CI 0.93 - 0.99] | |
|  |  |  |  |  |  |  |  |  |  |  |  | Treadmill: ICC = 0.98 [CI 0.93 - 0.99] | |
|  |  |  | Stroke | Duclos et al. (2019) | CV | DO | Ankle | Prop | N.R. | N.R. | % error | 6MWT: 0.50 [CI 0.24 - 6.79] | |
|  |  |  |  |  |  |  |  |  |  |  |  | Circuit: 2.67 [CI 0.61 - 12.54] | |
|  |  |  |  | Henderson et al. (2021) | CV | DO | Ankle (a) | Prop | N.R. | N.R. | ICC | PT session: ICC = 0.71 [CI 0.24; 0.87] | |
|  |  |  |  |  |  |  |  |  |  |  |  | Walk training: ICC = 0.92 [CI 0.36; 0.98] | |
|  |  |  |  |  |  |  | Ankle (ua) | Prop | N.R. | N.R. | ICC | PT session: ICC = 0.78 [CI 0.39; 0.91] | |
|  |  |  |  |  |  |  |  |  |  |  |  | Walk training: ICC = 0.92 [CI 0.59, 0.97] | |
|  |  |  |  | Hui et al. (2018) | CV | Acc (Actical - ankle) | Ankle (h) | Prop | 60 sec | N.R. | Regressions r | Day 1: r = 0.99 [lower CI 0.97] | |
|  |  |  |  |  |  |  |  |  |  |  |  | Day 2: r = 0.99 [lower CI 0.96] | |
|  |  |  |  |  |  |  |  |  |  |  |  | Day 3: r = 0.97 [lower CI 0.92] | |
|  |  |  |  | Klassen et al. (2016) | CV | DO | Ankle (h) | Prop | N.R. | N.R. | Mean percentage error | 0.3 m/s: 15.8 [CI 9.1 - 22.7]% | |
|  |  |  |  |  |  |  |  |  |  |  |  | 0.4 m/s: 5.5 [CI 2.4 - 8.6]% | |
|  |  |  |  |  |  |  |  |  |  |  |  | 0.5 m/s: 4.5 [CI 2.4 - 6.6]% | |
|  |  |  |  |  |  |  |  |  |  |  |  | 0.6 m/s: 4.0 [CI 2.4 - 5.6]% | |
|  |  |  |  |  |  |  |  |  |  |  |  | 0.7 m/s: 4.9 [CI 2.1 - 7.7]% | |
|  |  |  |  |  |  |  |  |  |  |  |  | 0.8 m/s: 6.9 [CI 2.8 - 11.0]% | |
|  |  |  |  |  |  |  |  |  |  |  |  | 0.9 m/s: 4.9 [CI 1.8 - 8.0]% | |
|  |  |  |  |  |  |  | Waist | Prop | N.R. | N.R. | Mean percentage error | 0.3 m/s: 84.6 [CI 75.5 - 93.7]% | |
|  |  |  |  |  |  |  |  |  |  |  |  | 0.4 m/s: 59.1 [CI 47.1 - 71.1]% | |
|  |  |  |  |  |  |  |  |  |  |  |  | 0.5 m/s: 38.3 [CI 28.0 - 48.6]% | |
|  |  |  |  |  |  |  |  |  |  |  |  | 0.6 m/s: 16.6 [CI 10.8 - 22.4]% | |
|  |  |  |  |  |  |  |  |  |  |  |  | 0.7 m/s: 11.8 [CI 6.1 - 17.5]% | |
|  |  |  |  |  |  |  |  |  |  |  |  | 0.8 m/s: 10.1 [CI 5.4 - 14.8]% | |
|  |  |  |  |  |  |  |  |  |  |  |  | 0.9 m/s: 7.7 [CI 4.3 - 11.1]% | |
|  |  |  |  | Klassen et al. (2017) | CV | Acc (SAM - ankle) | Ankle (h) | Prop | N.R. | N.R. | Bland-Altman LoA | 156.1 [-239.6; 551.9] (u) | |
|  |  | Intensity time | Cancer | Van Blarigan et al. (2017) | CV | Acc (AG GT3 - waist) | Waist | Cut off: | N.R. | N.R. | Pearson r | Light: r = 0.72 | |
|  |  |  |  |  |  |  |  | - Light: <3.0 MET |  |  |  | Moderate: r = 0.70 | |
|  |  |  |  |  |  |  |  | - Moderate: 3 - 5.9 MET |  |  |  | Vigorous: r = 0.65 | |
|  |  |  |  |  |  |  |  | - Vigorous: ≥6.0 MET |  |  |  | MVPA: r = 0.85 | |
|  |  |  | Myositis | Saygin (2022) | CV | Acc (AG GT3 - waist) | Waist | Prop | N.R. | N.R. | ICC | Light: ICC = 0.96 (CI 0.91 - 0.98) | |
|  |  |  |  |  |  |  |  |  |  |  |  | Moderate: ICC = 0.73 (CI 0.35 - 0.89) | |
|  |  |  |  |  |  |  |  |  |  |  |  | Vigorous: ICC = 0.59 (CI 0.24 - 0.78) | |
|  |  |  | Stroke | Hui et al. (2018) | CV | Acc (Actival - ankle) | Ankle (h) | Prop | 60 sec | N.R. | Regressions r | Sed day 1: r = 0.94 [lower CI 0.83] | |
|  |  |  |  |  |  |  |  |  |  |  |  | Sed day 2: r = 0.78 [lower CI 0.45] | |
|  |  |  |  |  |  |  |  |  |  |  |  | Sed day 3: r = 0.41 [lower CI -0.11] | |
|  |  |  |  |  |  |  |  |  |  |  |  | Light day 1: r = 0.95 [lower CI 0.85] | |
|  |  |  |  |  |  |  |  |  |  |  |  | Light day 2: r = 0.91 [lower CI 0.92] | |
|  |  |  |  |  |  |  |  |  |  |  |  | Light day 3: r = 0.97 [lower CI 0.90] | |
|  |  |  |  |  |  |  |  |  |  |  |  | Moderate day 1: r = 0.90 [lower CI 0.70] | |
|  |  |  |  |  |  |  |  |  |  |  |  | Moderate day 2: r = 0.91 [lower CI 0.74] | |
|  |  |  |  |  |  |  |  |  |  |  |  | Moderate day 3: r = 0.83 [lower CI 0.56] | |
|  |  |  |  |  |  |  |  |  |  |  |  | Vigorous day 1: r = 0.86 [lower CI 0.63] | |
| Surge | Activity tracker | Steps | Parkinson's disease | Wendel et al. (2018) | CV | DO | Wrist (la) | Prop | N.R. | N.R. | ICC | Cont., comfortable: ICC = 0.38 [CI 0.06 - 0.64] | |
|  |  |  |  |  |  |  |  |  |  |  |  | Cont., fast: ICC = 0.13 [CI -0.21 - 0.44] | |
|  |  |  |  |  |  |  |  |  |  |  |  | Household sim: ICC = -0.003 [CI -0.10 - 0.14] | |
|  |  |  |  |  |  |  |  |  |  |  |  | Obstacle neg.: ICC = 0.41 [CI 0.03 - 0.67] | |
| Ultra | Activity tracker | Steps | Stroke | Costa et al. (2020) | CV | DO | Wrist (b) | N.R. | N.R. | N.R. | Pearson r | r = 0.67 | |
| Zip | Pedometer | Steps | COPD | Blondeel et al. (2020) | CV | Acc (Dynaport - Lower back) | Waist | Prop | N.R. | N.R. | Bland-Altman LoA | -1055 [-2820; 589] (ue) | |
|  |  |  |  | Prieto-Centurion et al. (2016) | CV | DO | Waist | N.R. | N.R. | N.R. | Bland-Altman LoA | 6 [-14; 25] (ue) | |
|  |  |  | Cardiac diseases | Thorup et al. (2017) | CV | Acc (Shimmer3 - Ankle) | Waist | Prop | N.R. | 50 Hz | ICC | Hospital 24h: ICC = 0.60 | |
|  |  |  |  |  |  |  |  |  |  |  |  | Hospital 3min of walking: ICC = 0.79 | |
|  |  |  |  |  |  |  |  |  |  |  |  | Home 24h: ICC = 0.87 | |
|  |  |  |  |  |  |  |  |  |  |  |  | Home 3min of walking: ICC = 0.96 | |
|  |  |  |  |  |  |  |  |  |  |  |  | Combined 24h: ICC = 0.86 | |
|  |  |  |  |  |  |  |  |  |  |  |  | Combined 3min of walking: ICC = 0.93 | |
|  |  |  | MS | Lavelle et al. (2021) | CV | DO | Waist | N.R. | N.R. | N.R. | Bland-Altman LoA | -6.2 [-717.4, 705.0] (oe) | |
|  |  |  | Multi | Farmer et al. (2022) | CV | DO | Foot | Prop | N.R. | N.R. | ICC | indoor, All speeds: ICC = 0.74 (CI 0.29–0.88) | |
|  |  |  |  |  |  |  |  |  |  |  |  | Indoor, slow (<.80m/s): ICC = 0.60 (CI 0.03–0.82) | |
|  |  |  |  |  |  |  |  |  |  |  |  | Indoor, fast (>.80m/s): ICC = 0.65 (CI 0.24–0.83) | |
|  |  |  |  |  |  |  |  |  |  |  |  | Outdoor, All speeds: ICC = 0.81 (CI 0.38–0.92) | |
|  |  |  |  |  |  |  |  |  |  |  |  | Outdoor, slow (<.80m/s): ICC = 0.69 (CI 0.08–0.87) | |
|  |  |  |  |  |  |  |  |  |  |  |  | Outdoor, fast (>.80m/s): ICC = 0.85 (CI 0.43–0.94) | |
|  |  |  | Parkinson's disease | Wendel et al. (2018) | CV | DO | Waist | Prop | N.R. | N.R. | ICC | Cont. Comfortable: ICC = 0.98 [CI 0.96; 0.99] | |
|  |  |  |  |  |  |  |  |  |  |  |  | Cont. Fast: ICC = 0.90 [CI 0.81; 0.95] | |
|  |  |  |  |  |  |  |  |  |  |  |  | Household sim: ICC = -0.03 [CI -0.16; 0.16] | |
|  |  |  |  |  |  |  |  |  |  |  |  | Obstacle neg.: ICC = 0.58 [CI 0.24; 0.78] | |
|  |  |  | Polymyalgia rheumatica | Chandrasekar et al. (2018) | CV | DO | Waist | N.R. | N.R. | N.R. | Bland-Altman LoA | 2mwt: 10 [-55; 74] (ue) | |
|  |  |  |  |  |  |  |  |  |  |  |  | Stairs: 1 [-8; 10] (ue) | |
|  |  |  |  |  |  |  | Shirt, midline | N.R. | N.R. | N.R. | Bland-Altman LoA | 2mwt: 12 [-58; 83] (ue) | |
|  |  |  |  |  |  |  |  |  |  |  |  | Stairs: -6 [-81; 68] (oe) | |
|  |  |  | Stroke | Clay et al. (2019) |  |  | Waist | N.R. | N.R. | N.R. | Kendall Tau-b | τ = 0.80 | |
|  |  |  |  | Schaffer et al. (2017) | CV | DO | Waist | N.R. | N.R. | N.R. | Mean absolute percentage error | All: -18.9 [CI -104.5; 66.7]% | |
|  |  |  |  |  |  |  |  |  |  |  |  | Slow walkers: -88.2 [CI -134.0; -42.4]% | |
|  |  |  |  |  |  |  |  |  |  |  |  | Fast walkers: 4.2 [CI -16.9; 25.4]% | |
|  |  |  |  |  | TRT R |  | Waist | N.R. | N.R. | N.R. | ICC | ICC = 0.974 | |
| **Garmin** | | | | | | | | | | | | |  |
| Forerunner 35 | Accelerometer | Steps | Stroke | Huber et al. (2022) | CV | Acc (SAM - ankle) | Wrist (ua) | N.R. | N.R. | N.R. | Bland-Altman LoA | All: 5.0 [-63.7; 2689.5] (ue) | |
|  |  |  |  |  |  |  |  |  |  |  |  | Speeds > 1m/s: -1.6 [-86.9; 83.5] (oe) | |
|  |  |  |  |  | TRT R |  | Wrist (ua) | N.R. | N.R. | N.R. | ICC | All: ICC = 0.996 [0.989, 0.998] | |
|  |  |  |  |  |  |  |  |  |  |  |  | Speeds > 1m/s: ICC = 0.989 [0.967, 0.997] | |
| Vivofit | Activity tracker | Steps | Amputation | Smith et al. (2019) | CV | DO | Wrist (b) | N.R. |  |  | ICC | ICC = 0.86 | |
|  |  |  | Heart failure | Vetrovsky et al. (2019) | CV | Acc (AG GT3 - Waist) | Wrist | N.R. |  |  | CCC | CCC = 0.89 [CI 0.75; 0.96] | |
|  |  |  | Multi (musculoskeletal, neurological, other) | Treacy et al. (2017) | CV | DO | Wrist | N.R. | N.R. | N.R. | ICC | ICC = 0.259 [CI -0.071; 0.556] | |
|  |  |  | Parkinson's disease | Lamont et al. (2018) |  | Acc (ActivPal - Thigh) | Wrist | N.R. | N.R. | N.R. | ICC | Indoor self-selected pace: ICC = 0.93 [CI 0.85; 0.97] | |
|  |  |  |  |  |  |  |  |  |  |  |  | Indoor 60 steps/min: ICC = 0.36 [CI -0.30; 0.68] | |
|  |  |  |  |  |  |  |  |  |  |  |  | Indoor 80 steps/min: ICC = 0.89 [CI 0.76; 0.95] | |
|  |  |  |  |  |  |  |  |  |  |  |  | Indoor 100 steps/min: ICC = 0.71 [CI 0.40; 0.86] | |
|  |  |  |  |  |  |  |  |  |  |  |  | Indoor 120 steps/min: ICC = 0.68 [CI 0.39; 0.85] | |
|  |  |  |  |  |  |  |  |  |  |  |  | Indoor 140 steps/min: ICC = 0.70 [CI 0.40; 0.85] | |
|  |  |  |  |  |  |  |  |  |  |  |  | Outdoor self-selected pace: 0.97 [CI 0.93; 0.99] | |
|  |  |  | Stroke | Schaffer et al. (2017) | CV |  | Wrist (h) | N.R. | N.R. | N.R. | Mean absolute percentage error | All: -40.7 [CI -217.1; 45.8]% | |
|  |  |  |  |  |  |  |  |  |  |  |  | Slow: -90.1.0 [CI -116.2; -64.0]% | |
|  |  |  |  |  |  |  |  |  |  |  |  | Fast: -16.0 [CI -74.0; 42.0]% | |
|  |  |  |  |  |  |  | Wrist (a) | N.R. | N.R. | N.R. | Mean absolute percentage error | All: -33.4 [CI -117.3; 50.5]% | |
|  |  |  |  |  |  |  |  |  |  |  |  | Slow: -68.2 [CI -141.5; 5.1]% | |
|  |  |  |  |  |  |  |  |  |  |  |  | Fast: -4.0 [CI-29.4; 21.4]% | |
|  |  |  |  |  | TRT R |  | Wrist (h) | N.R. | N.R. | N.R. | ICC | ICC = 0.964 [CI 0.916; 0.984] | |
|  |  |  |  |  |  |  | Wrist (a) | N.R. | N.R. | N.R. | ICC | ICC = 0.858 [CI 0.672; 0.939] | |
| Vivofit 3 | Activity tracker | Steps | Amputation | Smith & Guerra (2021) | CV | DO | Ankle | N.R. | N.R. | N.R. | ICC | ICC = 0.122 (CI -0.141 - 0.398) | |
|  |  |  |  |  |  |  | Wrist | N.R. | N.R. | N.R. | ICC | ICC = 0.895 (CI 0.802 - 0.945) | |
|  |  |  | Heart failure | Vetrovsky et al. (2019) | CV | Acc (AG GT3 - Waist) | Wrist | N.R. | N.R. | N.R. | CCC | CCC = 0.92 [CI 0.78; 0.97] | |
| Vivofit 4 | Activity tracker | Steps | MS | Lavelle et al. (2021) | CV | DO | Wrist | N.R. | N.R. | N.R. | Bland-Altman LoA | -251.05 [-717.4; 253.6] (oe) | |
|  |  | Activity time | MS | Lavelle et al. (2021) | CV | DO | Wrist | N.R. | N.R. | N.R. | % error | 100% [range 100 - 100] | |
| Vivosmart 3 | Activity tracker | Steps | Parkinson's disease | Lai et al. (2020) | CV | DO | Wrist (la) | N.R. | N.R. | N.R. | ICC | Overground: ICC = 0.97 [CI 0.96; 0.99] | |
|  |  |  |  |  |  |  |  |  |  |  |  | Treadmill: ICC = 0.67 [CI 0.27; 0.85] | |
| Vivosmart 4 | Activity tracker | Steps | Parkinson's disease | Bianchini (2022) | CV | DO | Wrist (b) | Prop | N.R. | N.R. | ICC | Both sides: ICC = 0.66 (CI 0.31 - 0.83) | |
|  |  |  |  |  |  |  |  |  |  |  |  | Affected side: ICC = 0.64 (CI 0.25 - 0.82) | |
|  |  |  |  |  |  |  |  |  |  |  |  | Less affected side: ICC = 0.63 (CI 0.34 - 0.80) | |
| **Omron** | | | | | | | | | | | | |  |
| Active Style Pro HJA-350 | Accelerometer | MET | Stroke | Shimizu et al. (2018) | CV | ACSM MET compendium | Waist | Prop | 10 sec | N.R. | T-test (1-sample) | Lying: 0.5 ± 0.5 vs 1.0, sign lower | |
|  |  |  |  |  |  |  |  |  |  |  |  | Sitting: 0.8 ± 0.4 vs 1.3, sign lower | |
|  |  |  |  |  |  |  |  |  |  |  |  | Standing: 1.1 ± 0.2 vs 1.3, sign lower | |
|  |  |  |  |  |  |  |  |  |  |  |  | Walking: 3.8 ± 0.9 vs 5.0, sign higher | |
| Active Style Pro HJA-750c | Accelerometer | EE | DM | Nishida et al. (2020) | CV | DLW | Waist | TEE = BMR (Ganpule's equation) * PAL | 60 sec | N.R. | Pearson r | TEE: r = 0.87 | |
|  |  | PAL | DM | Nishida et al. (2020) | CV | DLW | Waist | PAL = ([BMR (Ganpule's equation) + AEE (prop)]*10/9)*BMR | 60 sec | N.R. | Pearson r | r = 0.71 | |
|  |  | Intensity time | COPD | Miyamoto et al. (2018) |  | Acc (DynaPort MoveMonitor - Waist) | Waist | Cust | 10 sec | 32 Hz | Pearson r | ≥2.0 MET: r = 0.808 | |
|  |  |  |  |  |  |  |  |  |  |  |  | ≥3.0 MET: r = 0.801 | |
|  |  |  |  |  |  |  |  |  |  |  |  | ≥4.0 MET: r = 0.376 | |
|  |  |  |  |  |  | Acc (Actimark - Waist) | Waist | Cust | 10 sec | 32 Hz | Pearson r | ≥2.0 MET: r = 0.832 | |
|  |  |  |  |  |  |  |  |  |  |  |  | ≥3.0 MET: r = 0.718 | |
|  |  |  |  |  |  |  |  |  |  |  |  | ≥4.0 MET: r = -0.045 | |
| HJ-113 | Pedometer | Steps | Amputation | Smith et al. (2019) | CV | DO | Waist | N.R. | N.R. | N.R. | ICC | ICC = 0.928 | |
| HJ-322U-E | Pedometer | Steps | Heart failure | Vetrovsky et al. (2019) | CV | Acc (AG GT3 - Waist) | Waist | N.R. | N.R. | N.R. | CCC | CCC = 0.82 [CI 0.56; 0.93] | |
| HJ-720ITC | Pedometer | Steps | COPD | Danilack et al. (2015) | CV | DO | Waist | N.R. | 4 sec | N.R. | Bland-Altman LoA | 34 [-186; 253] | |
| Walking Style x | Pedometer | Steps | Multi (cardiovascular, cancer, respiratory, musculoskeletal, neurological) | Ummels et al. (2018) | CV | DO | Waist | N.R. | N.R. | N.R. | Pearson r | r = 0.25 | |
| **Yamax** | | | | | | | | | | | | |  |
| Digiwalker CW-700 | Pedometer | Steps | Bronchiectasis | O'Neill et al. (2017) | CV | Acc (AG GT3 - Waist) | Waist | N.R. | N.R. | N.R. | Bland-Altman LoA | -167 [-3078; 2745] (oe) | |
|  |  |  | Multi (Cancer, Respirotory, Musculoskeletal, neurological) | Ummels et al. (2018) | CV | DO | Wrist | N.R. | N.R. | N.R. | Pearson r | r = -0.33 | |
|  |  | Activity time | Bronchiectasis | O'Neill et al. (2017) | CV | Acc (AG GT3 - Waist) | Waist | N.R. | N.R. | N.R. | Bland-Altman LoA | Daily activity time: 165 [62; 269] min | |
| Digiwalker SW-200 | Pedometer | Steps | MS | Anens et al. (2023) | CV | DO | N.R. | N.R. | N.R. | N.R. | Spearman's rho | Comfortable: rho = 0.84 | |
|  |  |  |  |  |  |  |  |  |  |  |  | Fast: rho = 0.97 | |
|  |  |  |  |  |  |  |  |  |  |  |  | Slow: rho = 0.64 | |
|  |  |  |  |  |  |  |  |  |  |  |  | Total: rho = 0.82 | |
|  |  |  |  | Balto et al. (2016) | CV | DO | Waist | N.R. | N.R. | N.R. | Mean % error | 1e trial: 8.5% | |
|  |  |  |  |  |  |  |  |  |  |  |  | 2e trial: 9.7% | |
|  |  |  |  | Lavelle et al. (2021) | CV | DO | Waist | N.R. | N.R. | N.R. | Bland-Altman LoA | 119.4 [-498.0; 736.8] (ue) | |
|  |  | EE | COPD | Farooqi et al. (2015) | CV | DLW | Waist | Harris-Benedict (RMR prediction equation) | N.R. | N.R. | ICC | ICC = 0.70 [CI 0.23; 0.89] | |
|  |  |  |  |  |  |  |  | Schofield (RMR prediction equation) | N.R. | N.R. | ICC | ICC = 0.71 [CI 0.21; 0.89] | |
|  |  |  |  |  |  |  |  | WHO (RMR prediction equation) | N.R. | N.R. | ICC | ICC = 0.74 [CI 0.33; 0.90] | |
|  |  |  |  |  |  |  |  | Moore (RMR prediction eqaution) | N.R. | N.R. | ICC | ICC = 0.69 [CI 0.21; 0.88] | |
|  |  |  |  |  |  |  |  | Nordic Nutrtion Recommendation (RMR prediction equation) | N.R. | N.R. | ICC | ICC = 0.70 [CI 0.17; 0.89] | |
|  |  |  |  |  |  |  |  | Nordenson (RMR prediction equation) | N.R. | N.R. | ICC | ICC = 0.40 [CI -0.16; 0.77] | |
|  |  | PAL | COPD | Farooqi et al. (2015) | CV | DLW + IC | Waist | PAL: | N.R. | N.R. | ICC | ICC = 0.34 | |
|  |  |  |  |  |  |  |  | - <5000 steps/day "Sedentary" = 1.4 |  |  |  |  |  |
|  |  |  |  |  |  |  |  | - 5000-7499 steps/day "Low active" = 1.6 |  |  |  |  |  |
|  |  |  |  |  |  |  |  | - 7.500-9999 steps/day "somewhat active" = 1.8 |  |  |  |  |  |
| **Google** | | | | | | | | | | | | |  |
| Fit | App | Steps | Parkinson's disease | de Carvalho Lana et al. (2021) | CV | DO | Waist | N.R. | N.R. | N.R. | Pearson r | r = 0.92 | |
|  |  |  | Stroke | Costa et al. (2020) | CV | DO | Waist | N.R. | N.R. | N.R. | Pearson r | r = 0.66 | |
|  |  |  |  |  | TRT R |  | Waist | N.R. | N.R. | N.R. | ICC | ICC = 0.76 | |
|  |  |  |  | Polese et al. (2019) | CV | DO | Front pocket (a) | N.R. | N.R. | N.R. | ICC | ICC = 0.93 [CI 0.86; 0.96] | |
|  |  | EE | Stroke | Faria et al. (2019) | CV | IC | Front pocket (a) | N.R. | N.R. | N.R. | Pearson r | r = 0.30 | |
| Android stepcounter | App | Steps | Rheumatoid arthritis | Wagner et al. (2022) | CV | DO | Waist | Prop | N.R. | N.R. | MAPE | Samsung A02: | |
|  |  |  |  |  |  |  |  |  |  |  |  | 2.5 km/h: 19.3 [min max = 0.0 - 74.0] | |
|  |  |  |  |  |  |  |  |  |  |  |  | 3 km/h: 7.3 [min max = 0.0 - 23.0] | |
|  |  |  |  |  |  |  |  |  |  |  |  | 3.5 km/h: 5.3 [min max = 0 - 46.0] | |
|  |  |  |  |  |  |  |  |  |  |  |  | 4 km/h: 4.1 [min max = 0.0 - 29.0] | |
|  |  |  |  |  |  |  |  |  |  |  |  | 4.5 km/h: 2.9 [min max = 0.0 - 20.0] | |
|  |  |  |  |  |  |  |  |  |  |  |  | 5 km/h: 1.5 [min max = 0.0 - 5.0] | |
|  |  |  |  |  |  |  |  |  |  |  |  | Google pixel 4 | |
|  |  |  |  |  |  |  |  |  |  |  |  | 2.5 km/h: 17.7 [min max = 0.0 - 68.0] | |
|  |  |  |  |  |  |  |  |  |  |  |  | 3 km/h: 2.6 [min max = 0.0 - 34.0] | |
|  |  |  |  |  |  |  |  |  |  |  |  | 3.5 km/h: 2.4 [min max = 0 - 39.0] | |
|  |  |  |  |  |  |  |  |  |  |  |  | 4 km/h: 1.0 [min max = 0.0 - 7.0] | |
|  |  |  |  |  |  |  |  |  |  |  |  | 4.5 km/h: 1.1 [min max = 0.0 - 16.0] | |
|  |  |  |  |  |  |  |  |  |  |  |  | 5 km/h: 2.5 [min max = 0.0 - 45.0] | |
| **Apple** | | | | | | | | | | | | |  |
| Watch Sport | Activity tracker | EE | Multi (cardiovascular, diabetes mellitus) | Falter et al. (2019) | CV | IC | Wrist | Prop | 2-3 sec | N.R. | ICC | ICC = 0.797 | |
| Health | App | Steps | MS | Balto et al. (2016) | CV | DO | Front pocket | N.R. | N.R. | N.R. | Mean % error | 1e trial: 2.7% | |
|  |  |  |  |  |  |  |  |  | N.R. | N.R. |  | 2e trial: 2.9% | |
| Iphone CMPedometer | Algorithm | Steps | Peripheral arterial disease | Ata et al. (2018) | CV | DO | Hand or front pocket | Prop | N.R. | N.R. | % error | -7.2 ± 13.8% | |
| Iphone SE | mobile phone | Steps | Cancer | Douma et al (2018) | CV | Acc (AG GT3 - wrist) | Waist | N.R. | N.R. | N.R. | ICC | ICC = 0.97 [CI 0.95; 0.98] | |
|  |  | Distance walked | Cancer | Douma et al (2018) | CV | Acc (AG GT3 - wrist) | Waist | N.R. | N.R. | N.R. | ICC | ICC = 0.47 [CI 0.21; 0.67] | |
| **Geonaute** | | | | | | | | | | | | |  |
| Onstep 400 | Pedometer | EE | Stroke | Compagnat et al. (2020) | CV | IC | Waist | Prop | N.R. | N.R. | Pearson r | TEE: r = 0.66 | |
|  |  |  |  |  |  |  |  | Cust | N.R. | N.R. | Pearson r | TEE: r = 0.87 | |
|  |  |  |  | Mandigout et al. (2017) | CV | IC | Neck | N.R. | N.R. | N.R. | Spearman r | r = -0.16 | |
|  |  |  |  |  |  |  | Waist | N.R. | N.R. | N.R. | Spearman r | r = -0.07 | |
|  |  | Distance walked | Stroke | Compagnat et al. (2019a) | CV | DO | Neck | Prop | N.R. | N.R. | Pearson r | r = 0.91 | |
|  |  |  |  |  |  |  | Waist | Prop | N.R. | N.R. | Pearson r | r = 0.98 | |
| **JawBone** | | | | | | | | | | | | |  |
| Up2 | Activity tracker | Steps | MS | Balto et al. (2016) | CV | DO | Wrist | N.R. | N.R. | N.R. | Mean % error | 1e trial: 3.9% | |
|  |  |  |  |  |  |  |  |  |  |  |  | 2e trial: 1.9% | |
|  |  |  | Parkinson's disease | Wendel et al. (2018) | CV | DO | Wrist | Prop | N.R. | N.R. | ICC | Cont., comfortable: ICC = 0.10 [CI -0.26; 0.43] | |
|  |  |  |  |  |  |  |  |  |  |  |  | Cont., fast: ICC = -0.02 [CI -0.35; 0.32] | |
|  |  |  |  |  |  |  |  |  |  |  |  | Household sim: ICC = 0.17 [CI -0.07; 0.49] | |
|  |  |  |  |  |  |  |  |  |  |  |  | Obstacle neg.: ICC = 0.05 [CI -0.28; 0.38] | |
| Up24 | Activity tracker | Steps | Multi (cardiovascular, cancer, respiratory, musculoskeletal, neurological) | Ummels et al. (2018) | CV | DO | Wrist | N.R. | N.R. | N.R. | Pearson r | r = 0.09 | |
| Up Move | Activity tracker | Steps | MS | Balto et al. (2016) | CV | DO | Waist | N.R. | N.R. | N.R. | Mean % error | 1e trial: 8.4% | |
|  |  |  |  |  |  |  |  |  |  |  |  | 2e trial: 8.9% | |
|  |  |  | Parkinson's disease | Wendel et al. (2018) | CV | DO | Waist | Prop | N.R. | N.R. | ICC | Cont., comfortable: ICC = 0.85 [CI 0.63; 0.94] | |
|  |  |  |  |  |  |  |  |  |  |  |  | Cont., fast: ICC = 0.55 [CI 0.26; 0.75] | |
|  |  |  |  |  |  |  |  |  |  |  |  | Household sim: ICC = -0.03 [CI -0.08; 0.09] | |
|  |  |  |  |  |  |  |  |  |  |  |  | Obstacle neg.: ICC = 0.05 [CI -0.13; 0.29] | |
| **Polar** | | | | | | | | | | | | |  |
| A300 | Accelerometer | Steps | COPD | Boeselt et al. (2016) | CV | Acc (SenseWear - Arm) | Wrist | Prop | N.R. | N.R. | ICC | ICC = 0.986 | |
|  |  | Activity time | COPD | Boeselt et al. (2016) | CV | Acc (SenseWear - Arm) | Wrist | Prop | N.R. | N.R. | ICC | Daily activity: ICC = 0.335 | |
|  |  | MET | COPD | Boeselt et al. (2016) | CV | Acc (SenseWear - Arm) | Wrist | Prop: | N.R. | N.R. | ICC | ICC = 0.066 | |
|  |  |  |  |  |  |  |  | Light = 1.1 - 2.9 MET |  |  |  |  |  |
|  |  |  |  |  |  |  |  | Moderate = 3.0 - 5.9 MET |  |  |  |  |  |
|  |  |  |  |  |  |  |  | Vigorous = ≥6.0 MET |  |  |  |  |  |
|  |  | Calories | COPD | Boeselt et al. (2016) | CV | Acc (SenseWear - Arm) | Wrist | Prop | N.R. | N.R. | ICC | ICC = 0.829 | |
| Loop | Activity tracker | Steps | Amputation | Smith et al. (2019) | CV | DO | Wrist | N.R. | N.R. | N.R. | ICC | ICC = 0.723 | |
| T131 | Heart rate monitor | EE | Chronic long disease | Dhillon et al. (2018) | CV | IC | N.R. | Flex Heart Rate Method | N.R. | N.R. | Bland-Altman LoA | Flat walking: -0.48 [-1.61; 0.65] (ue) | |
|  |  |  |  |  |  |  |  |  |  |  |  | Incline walking: -0.37 [-1.66; 0.92] (ue) | |
|  |  |  |  |  |  |  |  |  |  |  |  | Sit to stand: -0.06 [-0.97; 0.85] (ue) | |
|  |  |  |  |  |  |  |  |  |  |  |  | Lift bend: 0.01 [-0.89; 0.91] (oe) | |
|  |  |  |  |  |  |  |  |  |  |  |  | Cycling (10%): -0.11 [-1.57; 1.35] (ue) | |
|  |  |  |  |  |  |  |  |  |  |  |  | Cycling (25%): 0.21 [-0.87; 1.29] (oe) | |
|  |  |  |  |  |  |  |  |  |  |  |  | Cycling (50%): 0.39 [-0.34; 1.12] (oe) | |
|  |  |  |  |  |  |  |  |  |  |  |  | Cycling (60%): 0.42 [-0.32; 1.16] (oe) | |
| **Samsung** | | | | | | | | | | | | |  |
| Galaxy S4 mini | Mobile phone | Mean vector magnitude | MS | Zhai et al. (2020) | CV | Acc (AG GT3 - wrist) | Habitual phone pos. | N.R. | 60 sec | 2 Hz | Spearman r | vs AG daily MVPA: r = 0.327 | |
|  |  |  |  |  |  |  |  |  |  |  |  | vs AG meanVM: r = 0.173 | |
|  |  |  |  |  |  |  |  |  |  |  |  | vs AG steps/min: r = 0.058 | |
|  |  | Variance vector magnitude | MS | Zhai et al. (2020) | CV | Acc (AG GT3 - wrist) | Habitual phone pos. | N.R. | 60 sec | 2 Hz | Spearman r | vs AG daily MVPA: r = -0.128 | |
|  |  |  |  |  |  |  |  |  |  |  |  | vs AG meanVM: r = 0.201 | |
|  |  |  |  |  |  |  |  |  |  |  |  | vs AG steps/min: r = 0.288 | |
| Health | App | Steps | Parkinson's disease | de Carvalho Lana et al. (2021) | CV | DO | Waist | N.R. | N.R. | N.R. | Pearson r | r = 0.54 | |
|  |  | Steps | Stroke | Costa et al. (2020) | CV | DO | Waist | N.R. | N.R. | N.R. | Pearson r | Android: r = 0.19 | |
|  |  |  |  |  |  |  |  |  |  |  |  | Iphone: r = 0.18 | |
|  |  |  |  |  | TRT R |  | Waist | N.R. | N.R. | N.R. | ICC | Android: ICC = 0.10 | |
|  |  |  |  |  |  |  |  |  |  |  |  | Iphone: ICC = -0.70 | |
| **Lumo** | | | | | | | | | | | | |  |
| Lumoback | Accelerometer | Steps | Multi (cardiovascular, cancer, respiratory, musculoskeletal, neurological) | Ummels et al. (2018) | CV | DO | Lower back | N.R. | N.R. | N.R. | Pearson r | r = 0.19 | |
|  |  | Intensity time | Lower back pain | Takasaki et al. (2017) | TRT R |  | Lower back | Prop | N.R. | N.R. | ICC | Sed.: ICC = 0.75 [CI 0.26; 0.91] | |
| **Pacer Health** | | | | | | | | | | | | |  |
| Pacer Pedometer | App | Steps | Parkinson's disease | de Carvalho Lana et al. (2021) | CV | DO | Waist | N.R. | N.R. | N.R. | Pearson r | r = 0.77 | |
|  | |  | Stroke | Costa et al. (2020) | CV | DO | Waist | N.R. | N.R. | N.R. | Pearson r | Android: r = 0.68 | |
|  |  |  |  |  |  |  |  |  |  |  |  | Iphone: r = 0.80 | |
|  |  |  |  |  | TRT R |  | Waist | N.R. | N.R. | N.R. | ICC | Android: ICC = 0.68 | |
|  |  |  |  |  |  |  |  |  |  |  |  | Iphone: ICC = 0.80 | |
| **Withings** | | | | | | | | | | | | |  |
| Go | Activity tracker | Steps | Heart failure | Vetrovsky et al. (2019) | CV | Acc (AG GT3 - waist) | Wrist | N.R. | N.R. | N.R. | CCC | CCC = 0.90 [CI 0.77-0.96] | |
| Health Mate | App | Steps | MS | Balto et al. (2016) | CV | DO | Front pocket | N.R. | N.R. | N.R. | Mean % error | 1e trial: 3.5% | |
|  |  |  |  |  |  |  |  |  |  |  |  | 2e trial: 1.5% | |
| **Alexander et al.** | | | | | | | | | | | | |  |
| mSteps | App | Distance walked | MS | Alexander et al. (2022) | CV | DO | Arm | N.R. | N.R. | N.R. | Bland-Altman LoA | 0.262 [-1.496; 2.020] m (oe) | |
| **Corussen LLC** | | | | | | | | | | | | |  |
| Accupedo | App | Steps | Multi (cardiovascular, cancer, respiratory, musculoskeletal, neurological) | Ummels et al. (2018) | CV | DO | Waist | N.R. | N.R. | N.R. | Pearson r | r = 0.32 | |
| **DHS group** | | | | | | | | | | | | |  |
| MOVEBAND | Accelerometer | Steps | Amputation | Smith et al. (2019) | CV | DO | Wrist (b) | N.R. | N.R. | N.R. | ICC | ICC = 0.897 | |
| **Juen** | | | | | | | | | | | | |  |
| MoveSense | App | Distance walked | Pulmonary disease | Juen et al. (2015) | CV | DO | Lower back | Cust | N.R. | N.R. | Bland-Altman LoA | -7.7 [CI -33.0; 17.6] meter (oe) | |
| **Leap Fitness Group** | | | | | | | | | | | | |  |
| Pedometro | App | Steps | Chronic pain | Ferreira et al. (2020) | CV | DO | Arm & waist | N.R. | N.R. | N.R. | Pearson r | For all tasks and placements: p ≥ 0.99 | |
| **Letscom** | | | | | | | | | | | | |  |
| Letscom smart watch | Activity tracker | Steps | MS | Lavelle et al. (2021) | CV | DO | Wrist | N.R. | N.R. | N.R. | Bland-Altman LoA | -390.0 [-1006.7; 226.7] (oe) | |
|  |  | Activity time | MS | Lavelle et al. (2021) | CV | Acc (AG GT3 - waist) | Wrist | N.R. | N.R. | N.R. | % error | 52.9% [range 5.6 - 65.1] | |
| **Mario Herzberg** | | | | | | | | | | | | |  |
| EasyFit pedometer | App | Steps | Chronic pain | Ferreira et al. (2020) | CV | DO | Arm & waist | N.R. | N.R. | N.R. | Pearson r | For all tasks and placements: p between -0.32 and 0.24 | |
| **Mio** | | | | | | | | | | | | |  |
| Slice | Activity tracker | EE | Coronary artery disease | Herkert et al. (2019) | CV | IC | Wrist | Prop | N.R. | N.R. | ICC | ICC = 0.12 | |
|  |  |  | Heart failure | Herkert et al. (2019) | CV | IC | Wrist | Prop | N.R. | N.R. | ICC | ICC = 0.11 | |
| **Nakosite** | | | | | | | | | | | | |  |
| 3D walking | Accelerometer | Steps | Stroke | Negrini et al. (2020) | CV | DO | Ankle (a) | Prop | N.R. | N.R. | ICC | 10MWT: -0.20 [CI -0.47; 0.10] | |
|  |  |  |  |  |  |  |  |  |  |  |  | 50MWT: 0.06 [CI -0.24; 0.36] | |
|  |  |  |  |  |  |  |  |  |  |  |  | 6MWT: 0.70 [CI 0.51; 0.83] | |
|  |  |  |  |  |  |  |  |  |  |  |  | TUG: 0.05 [95% CI -0.25; 0.34] | |
|  |  |  |  |  |  |  | Ankle (ua) | Prop | N.R. | N.R. | ICC | 10MWT: -0.19 [CI -0.46; 0.11] | |
|  |  |  |  |  |  |  |  |  |  |  |  | 50MWT: 0.37 [CI 0.08; 0.60] | |
|  |  |  |  |  |  |  |  |  |  |  |  | 6MWT: 0.69 [CI 0.50; 0.82] | |
|  |  |  |  |  |  |  |  |  |  |  |  | TUG: -0.28 [CI -0.54; 0.01] | |
|  |  |  |  |  |  |  | Waist | Prop | N.R. | N.R. | ICC | 10MWT: -0.42 [CI -0.64; 0.14] | |
|  |  |  |  |  |  |  |  |  |  |  |  | 50MWT: -0.40 [CI -0.61; 0.10] | |
|  |  |  |  |  |  |  |  |  |  |  |  | 6MWT: 0.57 [CI 0.33; 0.74] | |
|  |  |  |  |  |  |  |  |  |  |  |  | TUG: -0.26 [CI -0.52; 0.04] | |
|  |  |  |  |  |  |  | Wrist (a) | Prop | N.R. | N.R. | ICC | 10MWT: -0.50 [CI -0.34; 0.26] | |
|  |  |  |  |  |  |  |  |  |  |  |  | 50MWT: 0.07 [CI -0.24; 0.36] | |
|  |  |  |  |  |  |  |  |  |  |  |  | 6MWT: 0.45 [CI 0.17; 0.66] | |
|  |  |  |  |  |  |  |  |  |  |  |  | TUG: -0.17 [CI -0.44; 0.14] | |
|  |  |  |  |  |  |  | Wrist (ua) | Prop | N.R. | N.R. | ICC | 10MWT: -0.41 [CI -0.63; 0.13] | |
|  |  |  |  |  |  |  |  |  |  |  |  | 50MWT: -0.32 [CI -0.57; 0.02] | |
|  |  |  |  |  |  |  |  |  |  |  |  | 6MWT: 0.45 [CI 0.17; 0.66] | |
|  |  |  |  |  |  |  |  |  |  |  |  | TUG: -0.23 [CI -0.49; 0.08] | |
| **Pedometer Australia** | | | | | | | | | | | | |  |
| G-Sensor 2026 | Pedometer | Steps | Multi (musculoskeletal, neurological, other) | Treacy et al. (2017) | CV | DO | Waist | N.R. | N.R. | N.R. | ICC | ICC = 0.308 [CI -0.094; 0.604] | |
| **ProtoGeo Oy** | | | | | | | | | | | | |  |
| Moves | App | Steps | MS | Balto et al. (2016) | CV | DO | Front pocket | N.R. | N.R. | N.R. | Mean % error | 1e trial: 14.2% | |
|  |  |  |  |  |  |  |  |  |  |  |  | 2e trial: 12.5% | |
| **Technogym** | | | | | | | | | | | | |  |
| MyWellnes Key | Accelerometer | Intensity time | Diabetes mellitus type 2 | McGinley et al. (2015) | CV | SR | Waist | Prop | N.R. | 16 Hz | Spearman r | r = 0.81 [CI 0.76; 0.85] | |
|  |  |  |  |  |  |  |  | - light: <3.0 MET |  |  |  |  |  |
|  |  |  |  |  |  |  |  | - moderate: 3.0-5.9 MET |  |  |  |  |  |
|  |  |  |  |  |  |  |  | - vigorous: ≥6.0 MET |  |  |  |  |  |

EE = energy expenditure, MET = metabolic equivalent, PAL = physical activity level

CAD = coronay artery disease, COPD = chronic obstructive pulmonary disease, DM = diabetes mellitus, iSCI = incomplete spinal cord injury, MS = multiple sclerosis, PAD = pulmonary artery disease, PD = Parkinson's disease, RA = rheumatoid arthritis, SCI = spinal cord injury

CV = Criterion validity, Con V = construct validity, Resp = responsiveness, TRT R = test-retest reliability

Acc = accelerometer, DLW = doubly labelled water, DO = direct observation, IC = indirect calorimetry, SR = self-report, Q = questionnaire, AG = ActiGraph

(a) = affected side, (b) = both affected and unaffected side, (la) = less affected side, (LRL) = longest residual limb, (SRL) = shortest residual limb, (ua) = unaffected side

Cust = custom algorithm, LFE = low frequency effect, N.R. = not reported, Prop = proprietary algorithm, TEE = total energy expenditure, BMR = basal metabolic rate

APE = absolute percentage error, CCC = concordance correlation coefficients, ICC = intra class correlation, LoA = limits of agreement, MAPE = mean absolute percentage error, MARD = mean absolute relative difference, MPE = mean percentage error

[CI] = 95% confidence intervals, (oe) = over estimation, (ue) = under estimation, MVPA = moderate to vigorous physical activity, MWT = minutes walking test, Sed = sedentary, STS = sit-to-stand test, SWT = steps walk test
